# Advances and Challenges in Wearable Sensors for Health Monitoring

**DOI:** 10.1021/acsami.6c04520

**Published:** 2026-07-16

**Authors:** Laís Canniatti Brazaca, Alvaro Moreno Lozano, Beatriz Mayol, Desiree Tamara Scheidt, Domenico De Fazio, Enji Kim, Gabriel Maroli, Giulio Rosati, Hatice Ceren Ates, Hui Wang, Hye Jin Kim, Ji-Hwan Ha, Jin Yang, Jisoo Jeon, Jörg Goldhahn, Keval K. Sonigara, La Li, Mingyu Zhou, Seokjoo Cho, Shicheng Fan, Shuang Wu, Vanessa Matos Gonçalves, Vitor H. B. Dei Santi, Weixin Zhou, Wenzheng Heng, Won Gi Chung, Xinming Li, Yang Zou, Yunxia Jin, Zixuan Wu, Amay J. Bandodkar, Arben Merkoçi, Can Dincer, Chwee Teck Lim, Dae-Hyeong Kim, Emanuel Carrilho, Giovanni Antonio Salvatore, Guozhen Shen, Inkyu Park, Itthipon Jeerapan, Jang-Ung Park, Jin Wu, John S. Ho, John Rogers, Joseph Wang, Kevin W. Plaxco, Maria Cristina Ferreira de Oliveira, Martin Pumera, Noé Brasier, Omid Veiseh, Pooi See Lee, Vladimir V. Tsukruk, Wei Gao, Yong Zhu, Zhong Lin Wang, Zhou Li, Juliane Sempionatto, Osvaldo N. Oliveira

**Affiliations:** † São Carlos Institute of Chemistry, University of São Paulo, São Carlos, SP 13566-590, Brazil; ‡ National Institute of Science and Technology in Bioanalytics Lauro Kubota - INCTBio-LK, State University of Campinas (Unicamp), Campinas, SP 13083-970, Brazil; § Department of Bioengineering, 3990 Rice University, Houston, Texas 77030, United States; ∥ Department of Electrical and Computer Engineering, Rice University, Houston, Texas 77005, United States; ⊥ Department of Molecular Science and Nanosystems, 19047 Ca’ Foscari University of Venice, Via Torino 155, 30172 Venezia, Italy; # Department of Materials Science and Engineering, 26721 Yonsei University, Seoul 03722, Republic of Korea; ∇ Center for Nanomedicine, Institute for Basic Science (IBS), Seoul 03722, Republic of Korea; ○ 231882 Catalan Institute of Nanoscience and Nanotechnology (ICN2), CSIC and BIST, Campus UAB, Bellaterra, Barcelona 08193, Spain; ◆ EURECAT - Technology Research Center, Av. de, Parc Científic i de la Innovació TecnoCampus, Carrer d’Ernest Lluch, 36, 08302 Mataró, Barcelona, Spain; ¶ Institute of Biomedical Engineering (MIBE), Department of Electrical Engineering, TUM School of Computation, Information and Technology, 9184 Technical University of Munich, 85748 Garching n. Munich, Germany; †† School of Materials Science and Engineering, Nanyang Technological University, Singapore 639798, Singapore; ‡‡ Department of Biomedical Engineering, 65448 Yonsei University, Wonju 26493, Republic of Korea; §§ Center for Nanoparticle Research, Institute for Basic Science (IBS), Seoul 08826, Republic of Korea; ∥∥ Department of Mechanical Engineering, 34968 Korea Advanced Institute of Science and Technology. Daejeon 34141, Republic of Korea; ⊥⊥ Department of Optoelectronic Engineering, Key Laboratory of Optoelectronic Technology and Systems Ministry of Education, 47913 Chongqing University, Chongqing 400044, China; ## School of Materials Science and Engineering, 1372 Georgia Institute of Technology, Atlanta, Georgia 30332, United States; ∇∇ Institute for Molecular and Translational Medicine, Department of Health Sciences and Technology, 27219 ETH Zurich, Zurich 8092, Switzerland; ○○ Future Energy and Innovation Laboratory, Central European Institute of Technology, Brno University of Technology, Purky̌nova 123, Brno 61200, Czech Republic; ◆◆ School of Integrated Circuits and Electronics, 47833 Beijing Institute of Technology, Beijing 100081, China; ¶¶ Mechanobiology Institute, 37580 National University of Singapore, Singapore 117411, Singapore; ††† Institute for Health Innovation and Technology (iHealthtech), National University of Singapore, Singapore 117599, Singapore; ‡‡‡ Department of Mechanical and Aerospace Engineering, 6798 North Carolina State University, Raleigh, North Carolina 27695-7001, United States; §§§ 5401 Florida Institute of Technology, Melbourne, Florida 32901-6982, United States; ∥∥∥ São Carlos Institute of Physics, 117186 University of São Paulo, São Carlos, SP 13566-590, Brazil; ⊥⊥⊥ Andrew and Peggy Cherng Department of Medical Engineering, Division of Engineering and Applied Science, 172429 California Institute of Technology, Pasadena, California 91125, United States; ### Querrey Simpson Institute for Bioelectronics, 3270 Northwestern University, Evanston, Illinois 60208, United States; ∇∇∇ Center for Bio-Integrated Electronics, Northwestern University, Evanston, Illinois 60208, United States; ○○○ Department of Biomedical Engineering, Northwestern University, Evanston, Illinois 60208, United States; ◆◆◆ Beijing Institute of Nanoenergy and Nanosystems, Chinese Academy of Sciences, Beijing 101400, China; ¶¶¶ School of Medical Technology, Beijing Institute of Technology, Beijing 100081, China; †††† Department of Biomedical Engineering, National University of Singapore, Singapore 117599, Singapore; ‡‡‡‡ Research Center of Flexible Sensing Materials and Devices, School of Applied Physics and Materials, Wuyi University, Jiangmen 529020, China; §§§§ Department of Electrical and Computer Engineering, North Carolina State University, Raleigh, North Carolina 27606, United States; ∥∥∥∥ Center for Advanced Self-Powered Systems of Integrated Sensors and Technologies (ASSIST), North Carolina State University, Raleigh, North Carolina 27606, United States; ⊥⊥⊥⊥ Lampe Joint Department of Biomedical Engineering, North Carolina State University and University of North Carolina at Chapel Hill, Chapel Hill, North Carolina 27695, United States; #### Catalan Institution for Research and Advanced Studies (ICREA), Passeig de Lluís Companys, 23, Barcelona 08010, Spain; ∇∇∇∇ School of Chemical and Biological Engineering, Institute of Chemical Processes, Seoul National University (SNU), Seoul 08826, Republic of Korea; ○○○○ Division of Physical Science, Faculty of Science, 26686 Prince of Songkla University, Hat Yai, Songkhla 90110, Thailand; ◆◆◆◆ Center of Excellence for Trace Analysis and Biosensor, Center of Excellence for Innovation in Chemistry, Prince of Songkla University, Hat Yai, Songkhla 90110, Thailand; ¶¶¶¶ The ijE Electrochemistry for All Laboratory, Hat Yai, Songkhla 90110, Thailand; ††††† Graduate Program of Nano Biomedical Engineering (NanoBME), Advanced Science Institute, Yonsei University, Seoul 03722, Republic of Korea; ‡‡‡‡‡ Department of Neurosurgery, Yonsei University College of Medicine, Seoul 03722, Republic of Korea; §§§§§ State Key Laboratory of Optoelectronic Materials and Technologies, Guangdong Province Key Laboratory of Display Material and Technology, School of Electronics and Information Technology, 529582 Sun Yat-sen University, Guangzhou 510275, China; ∥∥∥∥∥ Department of Electrical and Computer Engineering, National University of Singapore, Singapore 117599, Singapore; ⊥⊥⊥⊥⊥ Department of Mechanical Engineering, Northwestern University, Evanston, Illinois 60208, United States; ##### Rhaeos Inc., Evanston, Illinois 60201, United States; ∇∇∇∇∇ Department of Materials Science and Engineering, Northwestern University, Evanston, Illinois 60208, United States; ○○○○○ Department of Neurological Surgery, Feinberg School of Medicine, Northwestern University, Chicago, Illinois 60611, United States; ◆◆◆◆◆ Aiiso Yufeng Li Family Department of Chemical and Nano Engineering, 8784 University of California San Diego, La Jolla, San Diego, California 92093, United States; ¶¶¶¶¶ Department of Chemistry and Biochemistry, 8786 University of California, Santa Barbara, California 93106, United States; †††††† BioEngineering Department, University of California Santa Barbara, Santa Barbara, California 93106, United States; ‡‡‡‡‡‡ Institute of Mathematical Sciences and Computing, 28133 University of Sao Paulo, Sao Carlos, SP 13566-590, Brazil; §§§§§§ Advanced Nanorobots and Multiscale Robotics Laboratory, Faculty of Electrical Engineering and Computer Science, VSB—Technical University of Ostrava, 17. Listopadu 2172/15, Ostrava 70800, Czech Republic; ∥∥∥∥∥∥ Department of Chemical and Biomolecular Engineering, Yonsei University, 50 Yonseiro, Seodaemun-gu, Seoul 03722, Republic of Korea; ⊥⊥⊥⊥⊥⊥ School of Nanoscience and Technology, University of Chinese Academy of Sciences, Beijing 100049, China; 56 Department of Mechanical Engineering, Hanbat National University, Daejeon 34158 Republic of Korea

**Keywords:** wearable devices, sensors, biosensors, health monitoring, perspectives

## Abstract

Analytical tools may revolutionize healthcare by enabling accessible, rapid, and decentralized testing. Wearable (bio)­sensors, in particular, provide frequent or continuous patient monitoring through non- to minimally invasive measurements. This approach yields unprecedented amounts of health-related information, leading to more informed clinical decision-making and closer patient follow-up. In this mega-review article, we bring together leading researchers in the field to discuss the state of the art in wearable devices for health monitoring. We begin by providing a broad overview of the field through citation network analysis. We then review the application of chemical (bio)­sensors in biofluids (e.g., sweat, saliva, tears, interstitial fluid, and cerebrospinal fluid), highlighting the challenges and advantages associated with each. Subsequently, we discuss the construction of wearable devices and their main formats (e.g., smart contact lenses, textiles, mouthguards, watches/wristbands, and implantable systems). Physical sensors are addressed in a dedicated section focusing on the assessment of heart rate, blood pressure, and body temperature. The role of soft electronics in wearable devices is also examined, as these technologies are essential for enhancing user comfort and sensor reliability, which demands advances in materials science. Furthermore, we present strategies for signal acquisition and transmission, as well as approaches for on-body energy harvesting and device self-powering. The use of artificial intelligence and machine learning is then discussed as a means of enhancing analytical performance and managing the large volumes of data generated by wearable devices. Finally, business, regulatory, and ethical considerations are examined. We expect that this review will provide an overview of sensing and biosensing technologies for health-related applications, identify promising research directions, and inspire future developments.

## Graphical abstract

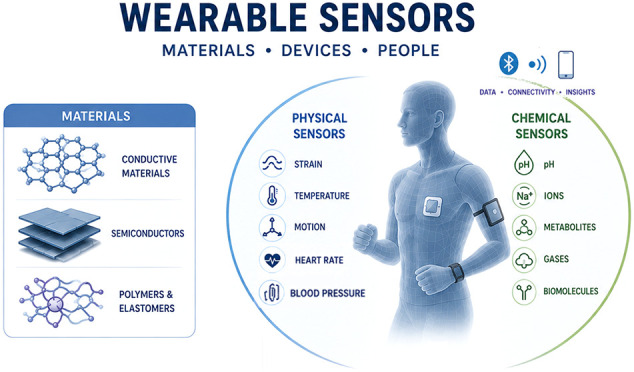

## 1 Introduction

Wearable (bio)­sensors are commonly designed for the continuous monitoring of an individual’s health status. These devices can provide real-time tracking of physical, physiological, and biochemical parameters, offering insights into clinical information.^1–3^ Sensors designed to monitor physical parameters are classified as physical wearable sensors.^4^ They include: (1) inertial sensors, to monitor body motion such as acceleration and angular velocity; (2) optical sensors, which rely on light’s transmission or reflection properties to monitor heart rate and oxygen saturation; and (3) electrical sensors, to detect biopotentials such as the electrical signals generated by the body, allowing for the measurement of cardiac activity (electrocardiogram) or muscle function (electromyography).^5–7^ This type of monitoring using smartphones, smartwatches, and patches outside healthcare institutions is transforming medical care. Representative examples include cardiac diseases, shifting away from bulky, time-limited classical Holter electrocardiograms (ECGs) toward more seamless solutions for detecting^8–10^ and managing^11^ atrial fibrillation in real-life settings. Additionally, physically assessed parameters can guide managing health conditions, including (i) respiratory diseases such as chronic obstructive pulmonary disease (COPD)^12^ and interstitial lung disease,^13^ (ii) neurological disorders like multiple sclerosis,^14^ Parkinson disease,^15^ and Alzheimer’s disease,^16^ (iii) autoimmune diseases such as inflammatory arthritis^17^ and Raynaud’s phenomenon in systemic sclerosis,^18^ (iv) dermatological conditions like atopic dermatitis,^19^ and (v) mental health disorders such as depression^20^ and schizophrenia.^21^ While physical measurements have supported the management of health conditions, including COVID-19,^22^ they mostly still lack disease-specific molecular biomarkers, such as those obtained from blood analysis.

The next generation of wearable devices should enable analysis of body fluids, including sweat, saliva, and interstitial fluid (ISF), among others. This approach represents a paradigm shift in healthcare, enabling continuous, lab-independent collection of molecular health information.^23^ There are pros and cons attributable to the use of each body fluid and device designs, which can follow the needs along the life cycle (Figure 1). It is of utmost importance, however, to understand biomarker partitioning mechanisms^24^ as well as body fluids’ (patho-)­physiology as, for example, sweat rate is influenced by a variety of internal and external factors.^25,26^ Latest developments in the field have demonstrated sweat’s potential to manage hydration,^27^ heart failure,^28,29^ disorders of substance abuse,^30^ and biological age.^31^ For breath, smart masks provide straightforward analysis of exhaled breath condensate to manage asthma or COPD^32^ and detect SARS-CoV-2.^33^ In terms of validated clinical use, continuous glucose monitoring through ISF analysis has transformed care for diabetic patients with a positive impact on Hb1Ac in patients with Type II Diabetes^34^ among others.^35^ A body fluid sensor, for example, could be selected based on clinical indication to personalize therapeutic drug monitoring for antibiotic treatment.^36^ Patients with pneumonia might undergo breath analysis, while those with soft tissue infections could use wearable devices for sweat analysis to monitor tissue penetration, drug concentrations, and ensure compliance with narrow therapeutic windows.

**Figure 1:**
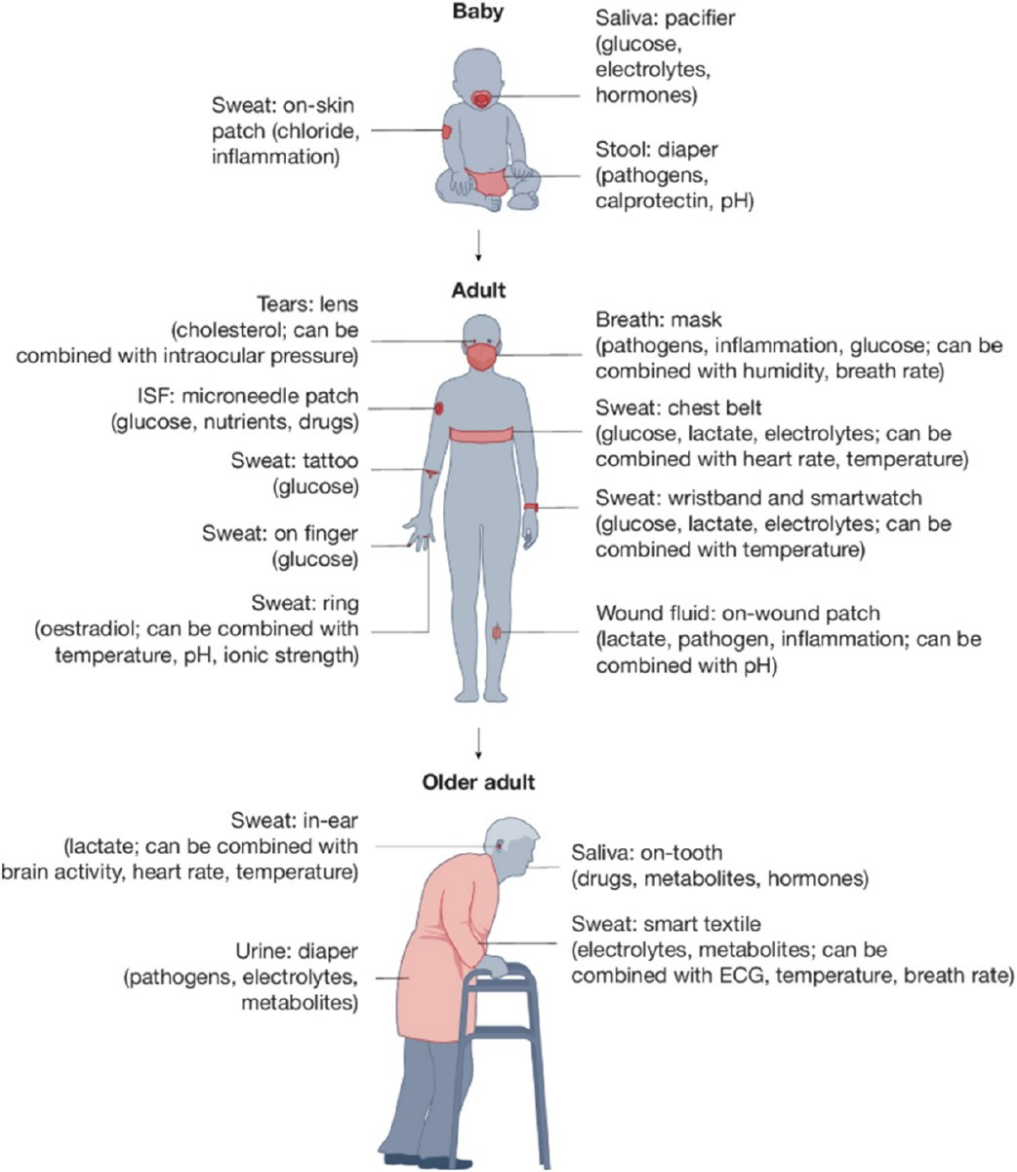
Accessing various body fluids to serve the various needs along the life cycle, ranging from saliva analysis through pacifier to in-ear sweat analysis in advanced age. Reproduced with permission from Brasier et al.,^23^ © 2024 Springer Nature.

Wearable sensors drive person-centered health management, having the potential to support overcoming population-based health challenges, such as antimicrobial resistance (AMR), climate change, and aging. For AMR, for example, incentives to develop novel antibiotics remain scarce.^37^ Therefore, optimizing drug therapy is crucial in fighting the silent pandemic of antimicrobials,^38^ with therapeutic drug monitoring being able to reduce antibiotic resistance and improve efficiency.^39^ While blood-based therapeutic drug monitoring is indicated during reserve antibiotic therapy, wearable devices for body fluid analysis may allow for understanding drug tissue penetration.^36^ Climate change leads to increased weather extremes, including heat waves.^40^ It is predicted that by the end of the 21st century, more than 95% of countries will be exposed to heat stress,^41,42^ which can lead to health issues and a decline in work performance.^43,44^ Wearable devices allow for monitoring hydration status in workers exposed to heat, ensuring safety, and enhancing productivity through haptic feedback,^27^ facilitating personalized heat strain management. Recent investigations have further demonstrated the potential to monitor metabolic changes related to heat stress, going beyond hydration monitoring.^45^ Aging societies increasingly challenge medical care systems as the elderly population grows, and their needs become more complex, requiring high-quality, equitable care. Wearable sensors have significant potential to support a livable and autonomous life at home and beyond.^46^

One of the most important tasks of medical doctors is to understand the derived health data in its broader context. Thus, efficient wearable sensing platforms can be coupled to artificial intelligence (AI) for automated data processing for interpretation, detection, prediction, and clinical outcome visualization.^23,47^ Once fully validated, these sensors can be integrated into closed-loop management approaches, enabling automated and person-centered therapies. Therefore, sensors and actuators are coupled and coordinated through communication protocols.^48^ Various applications have been identified in areas such as diabetes mellitus,^49^ wound care,^50^ and Parkinson’s disease,^15^ and in just-in-time adaptive interventions in behavioral health^51^ when combined with digital therapeutics.

In this Mega Review, we describe and discuss the state of the art of wearable (bio)­sensors, including their diverse applications and formats, the methods for fabricating wearable devices, the basis for energy harvesting and self-powering, soft electronics, the use of machine learning (ML) and AI for data analysis, and the main challenges and prospects of the area. We expect this Review to serve as a reference for both early-career and experienced researchers in wearable devices, providing insightful information for future research.

## 2 Landscape of Wearable Sensors for Health Monitoring

### 2.1 Citation Network

Landscapes of scientific fields can be generated using computational methods based on network analysis and natural language processing (NLP),^52^ particularly with large-language models. For instance, citation networks and NLP have been employed to identify the most relevant areas in journals covering chemistry and materials sciences^53^ and in providing a historical account of the journal.^54^ This approach provides a bird’s eye view of a particular field and estimates its size in terms of the number of publications. We have employed it here by generating citation networks for papers retrieved with searches using various queries to the OpenAlex database.^55^ For that, we applied the following queries: “*wearable sensors or health monitoring*”; “*wearable sensors*”; “*wearable health monitoring*”; “*wearable health technology*“; “*wearable sensors and health monitoring*“; “*wearable medical devices*”; “*materials for wearable sensors*”; “*wearable health monitoring technology*”; “*wearable sensors for healthcare*”; “*wearable sensors and human health monitoring*”. The searches were conducted with a title and abstract filter (“title and abstract”). The broadest term in scope was the first (“*wearable sensors or health monitoring”*), which retrieved a considerable number of papers related to the monitoring of the “health” of constructions, bridges, machines, and batteries, not being associated with monitoring the health of humans and other animals. Judging from the communities (i.e., clusters representative of topics), the network that best represented the topic of our interest was obtained with the query *“wearable sensors”*. Though “*health monitoring*” was not included in the query, most of the papers are related to it, as it will be discussed next. The networks generated with the other queries were smaller and could be considered as being contained in the larger network for “*wearable sensors*”. The search using *“wearable sensors”* led to 68,310 papers, most of which were published in the last 20 years. Figure 2 shows updated numbers of papers published until the end of 2025, featuring a trend of increasing attention given to wearable sensors.

**Figure 2:**
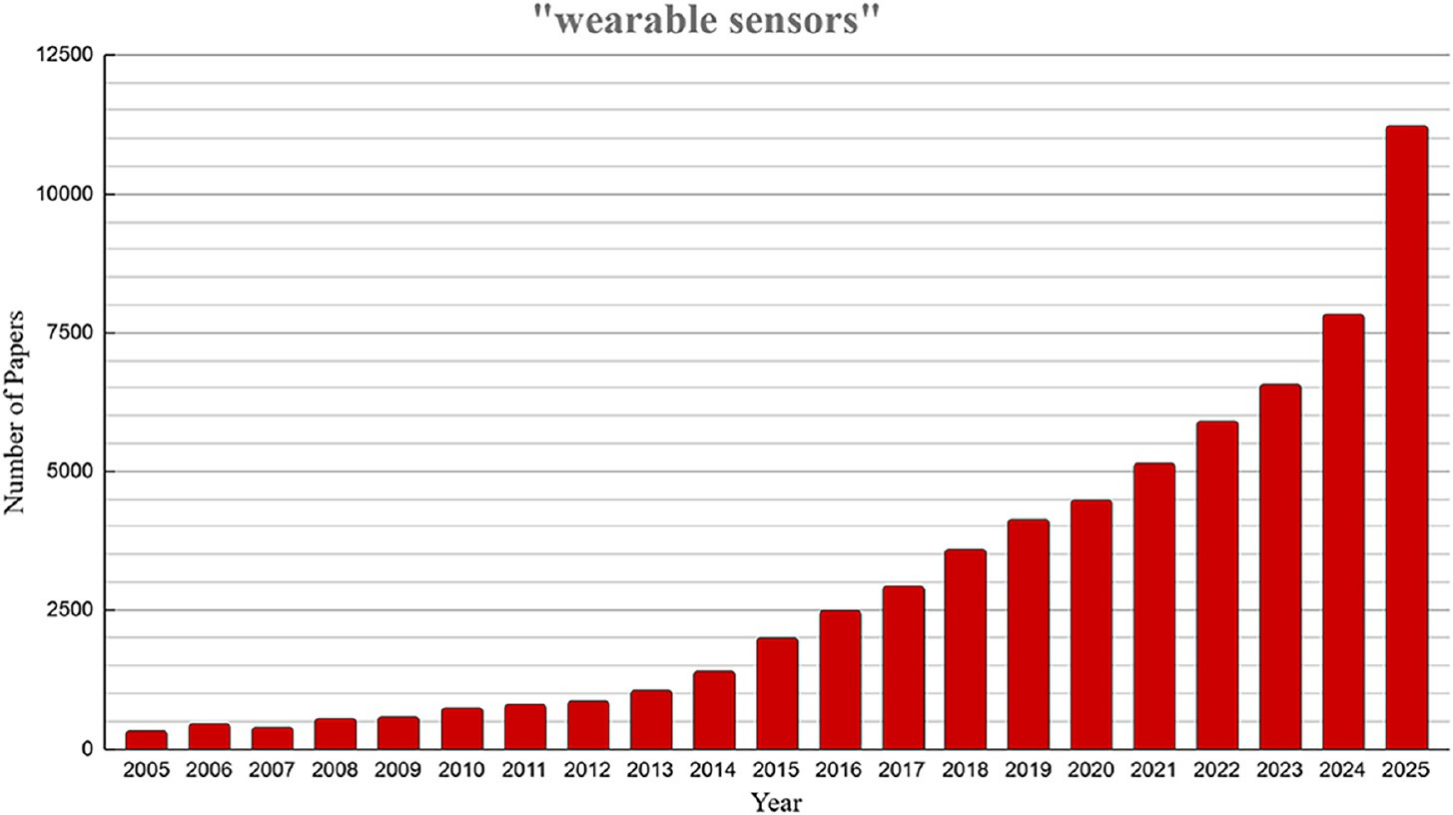
Number of papers published per year since 2005 for the search “wearable sensors” in the OpenAlex database in April, 2026.

The largest connected component of the citation network, obtained with the query “wearable sensors”, contains 35,935 papers (Figure 3). It is worth mentioning that the citation network includes only papers that cited others in the set of papers retrieved. Besides, this citation network may be formed by several subnetworks that are disconnected among themselves (the so-called “connected components”), and the subnetwork considered and shown in the figure corresponds to the largest of these connected components, with the smaller ones being ignored. Nevertheless, owing to the large number of papers, the network should be representative of the field. A community detection algorithm was applied to the network to identify clusters of densely connected papers and the major topics addressed by the papers in each cluster, following the methodology described elsewhere.^52–54^ These clusters are shown in different colors in the figure, and also their most relevant keywords characterizing the topics. The largest clusters, from A to F, can all be associated with sensors used for human health monitoring.

**Figure 3:**
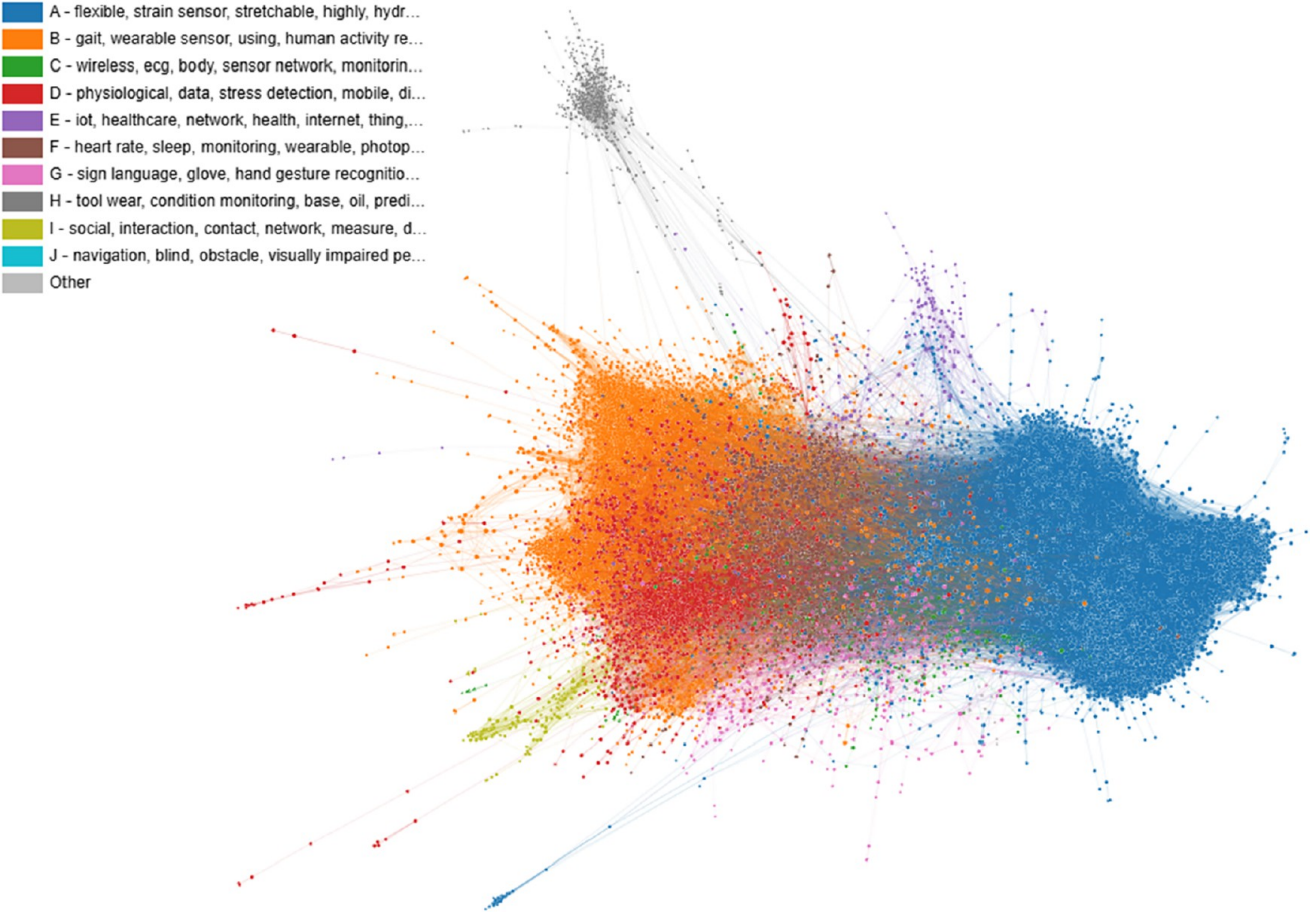
Citation network with 35,935 articles obtained from the search “wearable sensors”.

The largest cluster, A (in blue, with 13,962 articles), focuses on flexible and stretchable pressure and strain sensors. The second largest cluster, B (in orange, with 11,418 articles), relates to sensors used for monitoring human activities, such as gait and physiological conditions. Cluster C (in green, with 2,580 articles) is related to wearable physical sensors monitoring physiological conditions and is located near cluster B.[Fn fn1] Cluster D (in red, with 1,945 articles) explores the monitoring of emotional states and physiological stress using mobile sensors and learning algorithms. Cluster E (purple, with 1,871 articles) focuses on IoT infrastructure applied to healthcare, indicating attention to connectivity and intelligent systems for remote health surveillance. Cluster F (in brown, with 1,667 articles) brings together studies of heart rate and sleep monitoring, particularly using photoplethysmography (PPG).

The additional clusters covered more specific applications. Cluster G (in pink, with 967 articles) is dedicated to gesture-based interfaces, especially for *sign language* and *hand gesture recognition*, using glove-based sensors to translate finger and hand movements into digital commands. Cluster I (in light yellow, with 195 articles) addresses social interaction and group behavior. It includes studies that use wearable sensors to track interpersonal dynamics and proximity patterns. The small cluster J (in light blue, with 159 articles) focuses on assistive navigation for visually impaired individuals. This cluster highlights the use of wearable systems to enhance spatial awareness and autonomy for people with visual impairments. There is a cluster significantly distant from the others: cluster H (in dark gray, with 937 articles), which relates to tool monitoring, including for the oil industry. This cluster is obviously very different as it does not pertain to human or animal health.

Considering the clusters unequivocally related to health monitoring with wearable sensors, we estimate the field’s size at approximately 36,000 articles in the citation network, equivalent to about 60,000 articles in total.

Though the other, more specific searches led to networks that could be considered subsets of that of Figure 3, they are useful to reveal additional topics that could have been hidden in the broader search. Indeed, clusters emerged from the other networks that could be associated with machine learning and artificial intelligence methods in health monitoring, wireless communication to transfer data from the sensors, specific monitoring of cardiovascular conditions, and the use of textiles in wearable devices. The main conclusion from the analysis of the network in Figure 3 is that the main topics of the field of “wearable sensors for health monitoring” are flexible, stretchable pressure and strain sensors, followed by various technologies to monitor health using wearable sensors, mostly physical sensors, which include data science methods, machine learning, and artificial intelligence. Physiological conditions monitored include heartbeat, blood pressure, and other vital signs, sleep, stress, gait, while the most monitored disease is diabetes.

### 2.2 Recent Review Papers

The large growth in the number of papers on wearable sensors for health monitoring over the past few years is reflected by a significant increase in review papers. A search conducted on the Web of Science using the query “health monitoring AND wearable sensor*” across all fields (i.e., without restricting it to topic or title) retrieved 1,357 review papers. To provide a flavor of what is being covered in review papers, we made a manual selection of comprehensive review articles, with 22 being chosen, published from 2020 to 2026. Overall, the articles emphasize two major dimensions in the development of wearable sensor technology. On one side, there is a strong focus on the advancement of fabrication techniques and novel materials—such as 3D printing of polymer nanocomposites, conductive polymer-based hydrogels, graphene-based composites, and gold nanomaterials—to create sensors that are flexible, stretchable, and biocompatible. These methods aim to overcome the limitations of traditional rigid sensors, improving wearability and enabling continuous, noninvasive health monitoring. On the other side, a significant number of reviews concentrate on the integration of AI, particularly ML and deep learning (DL), to enhance data processing, pattern recognition, and predictive analytics in real time. We will cover the specific reviews on the use of AI for wearable sensors and health monitoring at the end of this subsection.

Most reviews examine physical sensors, such as motion sensors, inertial measurement units, ECG, PPG, and electromyography (EMG) devices along with their applications. These sensors are the most researched, serving today as the foundation of wearable technology. Chemical sensors, which are crucial for detecting biochemical markers in body fluids, are less explored and present significant challenges related to stability, specificity, and integration into wearable formats. Many of these challenges will be discussed throughout the different sections in this review paper.

Discrepancies arise mainly in the debate over optimal fabrication strategies and material selection. While some studies highlight the promise of novel materials like polymer nanocomposites and hydrogels for their superior flexibility and biocompatibility, others point out issues such as mechanical durability and signal stability. Additionally, although the integration of energy-harvesting solutions is recognized as important, there is less consensus on the best approaches for power management in long-term autonomous sensor operation. Overall, the review papers highlight required improvements in several areas: enhancing sensor durability and reliability; refining energy harvesting and storage solutions to support continuous operation; advancing AI algorithms for more robust and efficient on-device analysis; and increasing research on chemical sensors to achieve multianalyte detection with high specificity. Addressing these challenges is important for transitioning wearable sensors from the research stage to practical and scalable solutions in personalized healthcare.

The use of AI, particularly ML, in conjunction with wearable sensors has been reviewed by many authors from varied perspectives. A systematic review indicated a preference for standard ML over DL methods, primarily due to ML’s superior interpretability in classification tasks.^56^ The ability to detect complex patterns in large, high-dimensional data sets was highlighted concerning data from wearable sensors.^57^ Some of the reviews focused on specific areas of wearable sensors. For instance, AI-assisted cardiovascular health monitoring was examined by analyzing health indicators and noninvasive methods.^58^ Meanwhile, the literature on AI and smart devices for tracking the physical activity of people with diabetes examined the relationship between diabetes management and physical activity.^59^ A thorough analysis was conducted on the use of ML and DL for processing data from hydrogel sensors, particularly pressure and strain sensors.^60^ The role of AI in advancing electrochemical sensors for healthcare was discussed, with a focus on developing affordable point-of-care (POC) and point-of-use (POU) devices to improve diagnostic accuracy and health monitoring efficiency.^61^ AI and ML methods have primarily been used for data processing and prediction, relying entirely on software-based ML. However, there is growing research in the opposite direction, where wearable sensors are integrated with other devices to enable ML via hardware. Applications in both approaches have been reviewed, covering flexible sensors and the use of artificial synapses.^62^

As expected, all of these reviews express optimism about the transformative potential of AI in health monitoring. However, they also emphasize the challenges of fully harnessing AI. These challenges include obtaining consistent experimental data for model training, adapting algorithms to the evolving properties of flexible electronics, transferring trained models between devices, and enhancing the computational power for real-time updates. Additionally, difficulties in model generalization and the need for robust, interpretable ML methods remain significant obstacles. There is also concern about the societal implications of the use of AI.

## 3 Wearable (Bio)sensors for the Detection of Molecular Biomarkers

Wearable sensors can detect biochemical markers in various biofluids such as sweat, saliva, tears, wound fluid, and urine. These biomarkers typically originate through partitioning from blood plasma due to diffusion or active transport across biological membranes.^63,64^ Blood acts as the central transport system, carrying a range of molecules, such as hormones, proteins, metabolites, drugs, and inflammatory mediators, either in a free form or bound to carrier proteins. For partitioning to occur, these biomarkers must first pass through the vascular endothelium and into the surrounding tissue or fluid compartments. Size, charge, solubility, transport mechanism, metabolism, and degradation are some of the factors that influence the time lag, type, and concentration of biomarkers detectable in each biofluid, with the molecule size being an important limiting factor. Smaller molecules tend to partition easily into sweat, making it suitable primarily for detecting electrolytes, small metabolites, and certain hormones.^65^ Conversely, tears and saliva can contain relatively larger molecules, including hormones and specific proteins, due to the selective permeability of gland membranes.^64,66^ Interstitial and wound fluids usually contain biomarkers of a larger size due to the structure, permeability, and proximity to capillaries from the tissue.^67,68^ Figure 4 summarizes the size, production rates, and time required to find biomarkers in each of the biofluids compared to blood.^69^

**Figure 4:**
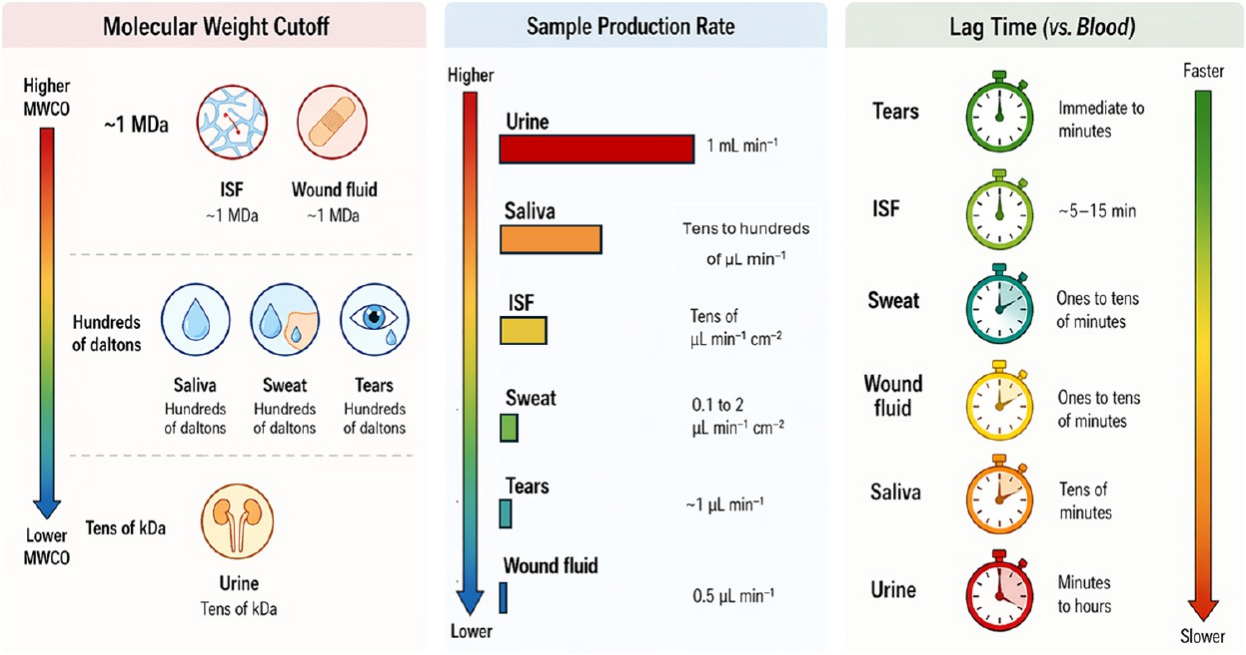
Comparison of molecular weight cutoff, production rates, and lag time (vs blood) for biomarkers in different biofluids.

The selection of a biofluid for wearable biosensing platforms involves careful consideration of several factors, including fluid accessibility, biomarker concentration, sampling invasiveness, stability, and correlation with physiological or pathological conditions. Sweat and saliva offer minimally invasive or noninvasive collection and continuous accessibility, making them attractive for real-time monitoring. However, biomarker concentrations in these fluids can vary widely due to factors such as hydration, diet, circadian rhythms, and physical activity, posing challenges in establishing consistent baseline levels.^70^ Saliva biomarkers generally show good correlation with systemic conditions, particularly hormones and stress-related markers,^71^ while sweat biomarkers often correlate well with electrolyte balance, metabolic activities, and hydration status.^65^ Tears and wound fluid contain specialized but highly relevant biomarker profiles for some health conditions. However, their sampling requires specialized sensors that are suited to limited fluid volumes and specific anatomical regions. Tears provide biomarkers relevant to ocular diseases, inflammation, and certain metabolic disorders,^72^ but their collection is challenging because of low volumes, rapid evaporation, and concentration instability. Wound fluid, rich in inflammatory markers, proteins, and indicators of healing progress, varies significantly depending on the wound type, infection status, and individual physiological responses, thereby complicating standardized biomarker detection.^73^ Urine, easily collectible and abundant, facilitates the analysis of metabolic waste biomarkers, although its intermittent availability limits continuous monitoring applications.

Various challenges must be addressed to ensure reliable biomarker detection in wearable systems. First, biofluid composition can vary among individuals depending on their physiological status, hydration levels, diet, and external factors such as environmental temperature or physical activity. This biological variability can complicate the establishment of baseline biomarker concentrations and the diagnostic thresholds. Second, biofouling, the nonspecific adsorption of proteins or biomolecules on sensor surfaces, can reduce sensor performance, demanding antifouling strategies and rigorous calibration protocols.^74^ Despite these limitations, the advantages of wearable biomarker detection are substantial. Real-time continuous monitoring of biofluids enables early detection of disease onset, proactive health management, timely intervention, and personalized therapeutic decisions, enhancing the overall efficiency of healthcare systems by potentially reducing the number of hospital visits and costs.

The following sections will explore each biofluid in detail, highlighting its attributes, challenges, and opportunities in wearable sensor applications.

### 3.1 Sweat

Sweat is a vital physiological fluid with significant implications for maintaining thermal homeostasis and for monitoring health status.^75–77^ The autonomic nervous system controls the secretion of sweat through activation of the sweat glands, primarily driven by sympathetic cholinergic stimulation.^78,79^ Eccrine glands are distributed throughout the human body, with the highest density in areas such as the palm and forehead.^80^ Sweat produced by these glands is primarily responsible for thermoregulation at elevated body temperatures, assisting the body to maintain an optimal temperature through evaporative cooling processes.^76,81,82^ Sweat also maintains skin hydration and establishes a slightly acidic environment as a protective barrier against external pathogens.^83–85^ Additionally, sweat helps to regulate electrolyte concentration, and it assists in the excretion of metabolic waste products to maintain fluid and biochemical balance within the body.^25,86–88^

In terms of its use in health monitoring, sweat functions as a rich and noninvasively accessible biofluid, best understood through three primary categories of application: exposures to exogenous species, loss of substances from the body, and biochemical assessments of physiological health. In the first, sweat provides a means to monitor contact with hazardous substances in the environment, such as heavy metals and toxic volatile organic compounds.^89–92^ As sweat glands reside in the dermis, they carry information due to direct physical exposure to the environment. They also provide indications of ingestion or inhalation, resulting from diffusive transport from the surrounding ISF and blood. This possibility also has implications in monitoring for the use of illicit drugs, either recreationally or for sports performance.^93,94^ In the second category, sweat serves as a channel for the loss of essential substances that require replenishment. The excretion of water and electrolytes such as sodium (Na^+^), potassium (K^+^), and chloride (Cl^–^) during physical exercise or heat exposure represents an obvious example, but other species, such as amino acids, are also important to consider.^95,96^ In the context of tracking parameters related to physiological health, sweat contains biomolecules that mirror internal body processes. Metabolites like glucose, lactate, and urea,^97–99^ along with hormones such as cortisol and estradiol,^100–102^ can pass from blood and ISF into sweat via transcellular and paracellular transport mechanisms. Peptides and amino acids are also present in sweat, as biomarkers of muscle activity and systemic health.^45,103^ Unlike the first two categories of application, for such types of medical purposes, reliable correlations between sweat and blood chemistry are necessary.

Traditional methods for generating eccrine sweat rely on natural mechanisms that follow from elevation in body temperature due to physical exercise or psychological stress, or from exposure to warm, humid environments such as those in saunas or baths/showers. While these natural processes are effective, they require standardization and control over sweat rate and composition. Moreover, these mechanisms for sweat generation are not effective for diseased, pediatric, and elderly subjects. For collection, absorbent pads represent one of the earliest and simplest methods. Weighing the pads before and after a period of sweating yields information on local sweat loss. Scrapping, squeezing, or spinning these pads allows for the removal of sweat for subsequent laboratory analysis. These methods do not, however, support capabilities in real-time data collection, and they require laboratory processing steps performed in specialized facilities by trained personnel. Additionally, the potential for contamination and evaporation during collection can compromise the accuracy of the analysis.^104–106^

These limitations for the induction and collection of sweat motivate the development of alternative methods. A good example is the approach used as the main clinical diagnostic for cystic fibrosis (CF), which relies on an analysis of the concentration of chloride in sweat.^107^ The system (the Macroduct Sweat Collection System, developed by ELITechGroup Inc.), introduced in the late 20th century, employs transdermal iontophoretic delivery of a pharmacological agent, pilocarpine, to stimulate the action of eccrine sweat glands. The collection of sweat that subsequently appears on the surface of the stimulated region of the skin occurs via a coiled plastic tube in a manner that prevents contamination. Transfer of sweat from this tube into a laboratory instrument, known as a chloridometer, allows for analysis of chloride concentration, which is the critical marker in CF diagnosis.^108^ Although this system represents a well-established clinical standard, failure rates due to insufficient sample collection can be high.^109,110^ Furthermore, its use is confined to clinical environments with clinical staff, and real-time monitoring is not possible.

A first attempt to overcome these limitations involved a skin-interfaced, textile-based patch with a pair of light-emitting diodes for optical measurements of pH and sodium using colorimetric indicators for each.^111,112^ Pairing this platform with a separate, external data acquisition system allows for sweat analysis in a system that we refer to as partially wearable. A few years later, a transferable tattoo paper was developed, which was suitable for mounting on the skin and designed for subsequent potentiometric readout with an external unit.^113,114^ The first fully wearable device for on-skin, quantitative analysis of sweat appeared in 2014. This platform used a porous elastomeric slab of PDMS with integrated colorimetric assays and a passive LC oscillator to allow options in optical and radio frequency measurements of sweat rate, loss, and pH, with additional capabilities in determining the concentrations of copper and iron in sweat.^115^ Later in the same year, an active radio frequency identification (RFID) tag incorporating a sweat sensing module was introduced for measuring electrolytes, mainly sodium, with power and wireless links provided by smartphones for user engagement.^116^ These early papers catalyzed a broad range of activities in this area, including an impressive, advanced version of this type of device, but with integrated electronics, batteries, and multimodal electrochemical sensors. Also in 2016, Ahyeon Koh et al. introduced a fully integrated microfluidic platform, as a “lab on the skin” technology, with collections of channels, valves, and reservoirs for capture, storage, and biomarker analysis of sweat. Examples included chloride, pH, glucose, and lactate sensing via colorimetric and options in wireless electronics for data communication.^117^

Microfluidic approaches quickly became an integral aspect of design in wearable sweat sensors (see Section 5.1 for an extensive account of microfluidics). These systems leverage capillary bursting valves and precisely patterned channels to direct small volumes of sweat with high spatial and temporal control, and with straightforward mechanisms for quantifying sweat rate and sweat loss for monitoring and interpreting biomarker concentrations. By engineering valves with varied bursting pressures, these platforms enable chrono-sampling (i.e., time sequential collection of separate small volumes of sweat into different reservoirs without mixing late sweat with early sweat), thereby linking biomarker concentrations to collection time to track dynamic physiological changes. Polydimethylsiloxane (PDMS) is a common material for these microfluidic systems, though its intrinsic hydrophobicity can hinder fluid collection and transport in certain cases. Introducing hydrophilic modifiers or integrating porous, wicking materials within the microchannels leads to enhanced transport efficiency and improved sensing fidelity.^118,119^ PDMS-based devices can also be susceptible to mechanical deformation and high-water vapor permeability.^120–122^ To mitigate mechanical deformation, researchers developed hard/soft composite structures that maintain device integrity under deformation.^123,124^ These systems employ printing materials that combine flexibility and robustness, allowing them to endure mechanical strain during wear while maintaining reliable sweat uptake, storage, and analytical performance. Reeder et al. addressed water vapor transport concerns by integrating block copolymers into the device design, improving barrier properties and overall performance.^125^ Laser engraving methods offer an alternative to molding approaches for forming the microfluidic constructs. Examples include laser-engraved polyimide (PI)-based microfluidic channels, with electrochemical sensors for monitoring electrolytes and metabolites.^126,127^ Techniques in 3D printing are also of interest due to their ability to support complex microfluidic architectures, enabling the hard/soft structures mentioned above. When implemented with optical-grade polymers, fabrication of microcuvettes for high-precision spectroscopic, colorimetric, and fluorometric assays is possible.^123,124,128^

Traditional laboratory-based analysis techniques based on spectroscopic methods, enzyme-linked immunosorbent assays (ELISA), and liquid chromatography–mass spectrometry (LC-MS), exhibit high sensitivity and specificity in sweat analysis.^100,129–131^ Requirements in complex workflows, expensive instrumentation, dedicated facilities, and trained personnel are not, however, suitable for continuous monitoring in compact devices for wearable platforms. Optical sensing modalities, including colorimetric and fluorometric methods, suitably calibrated against these traditional laboratory approaches, are attractive for sensors that can be implemented in thin, flexible formats on the skin. This is partly due to their simplicity, their ability to operate without supporting electronics and capacity for wireless readout using analysis of images captured with digital cameras in smartphones. Colorimetric sensors use enzymatic or chemically responsive assay components that produce quantifiable color changes upon reacting with target analytes, thereby allowing accurate measurements based on relating color information extracted from images to analyte concentration. Successful examples for sweat analysis include electrolytes,^118,132,133^ pH,^134,135^ lactate,^134,136–138^ metal ions,^139,140^ ketones,^141,142^ creatinine,^143^ urea,^144–146^ uric acid,^147,148^ glucose,^149–151^ cortisol,^152,153^ ammonia,^154^ and ethanol.^154,155^ The sensitivity of colorimetric assays may, however, be insufficient for detecting sweat biomarkers that are present only at low abundance, such as cytokines, which exist at picogram-per-milliliter levels.^156,157^ For these targets and others, fluorometric sensors offer superior sensitivity and specificity, as demonstrated in the detection of ions,^158^ amino acids,^159^ and other biomolecules in sweat. Lateral flow assays (LFAs), long established in POC diagnostics for their straightforward visual interpretation, represent additional options for use in wearable sweat sensing. A microfluidic platform integrating paper-based LFAs was used for immunoassays of cortisol in sweat.^160^ Other examples exploit LFA formats in a similar manner for aptamer-based cortisol sensing^152^ and potassium^161^ monitoring.

Electrochemical sensors are also important, particularly for real-time detection and application to chemical species where no colorimetric or fluorometric assays exist. Potentiometric sensors can be used to measure electrolyte concentrations (e.g., Na^+^, K^+^, Cl^–^),^162–164^ while amperometric sensors, often functionalized with enzymes or molecularly imprinted polymers (MIPs), enable detection of metabolites such as glucose, lactate, and ethanol through current responses to redox reactions.^165–168^ Impedimetric sensors allow label-free, highly specific, and sensitive detection of low-concentration biomarkers like cortisol.^169–172^ Conductometric sensors can capture ionic strength and sweat volume via shifts in conductivity.^173^ These electrochemical platforms, when integrated with wireless electronics, offer high specificity, fast response times, and continuous monitoring capabilities. Recent developments in multiplexed sensor arrays allow simultaneous tracking of multiple biomarkers in one wearable device, including pH, electrolytes, metabolites, and inflammation markers like C-reactive protein (CRP), providing a comprehensive view of physiological health.^174^

The technologies above can support a broad range of applications, such as the detection of hazardous heavy metals (e.g., Zn, Cd, Pb, Cu, and Hg), using electrochemical square-wave anodic stripping voltammetry (SWASV).^175–177^ Other sensors can monitor exposure to addictive substances, such as caffeine,^178^ nicotine,^179^ alcohol,^180^ and 2-fluoro-methamphetamine (2-FMA).^181^ As examples in the second area of application, reports describe sensors that can measure loss of water, electrolytes, amino acids, and other metabolites through eccrine sweat.^27,182,183^ The results are important for monitoring hydration status and maintaining homeostasis during physical activities associated with sports and manual labor. In one specific case, a microfluidic wearable band with channels for measuring sweat rate and total loss, containing colorimetric assays for sweat pH and lactate, permitted monitoring of indicators of muscle metabolism and fatigue.^138^

The third application area aims to establish sweat and noninvasive measurements of its chemistry as an alternative to traditional blood and urine assays for health monitoring and disease management. A key challenge in such uses is in establishing quantitative connections between sweat and blood, a task complicated by factors such as secretion dynamics and variable gland responses.^24,104,184^ Early studies were often hindered by inconsistencies in sweat induction and collection protocols, leading to unreliable data across many analytes.^185–187^ Even today, commonly targeted species such as sodium, chloride,^188^ glucose,^189–191^ and lactate^192^ continue to pose challenges due to their high sensitivity to factors like sweat rate and local metabolic activity. However, recent advances in wearable platforms, particularly those integrating microfluidic sampling with standardized iontophoretic stimulation, have begun to establish robust connections between sweat and blood chemistries. Under such controlled conditions, strong and reproducible correlations have been demonstrated for certain analytes, including potassium^188^ and ethanol.^193,194^ Urea^195^ and cortisol^196^ have also shown promising alignment with serum concentrations, offering potential pathways for noninvasive management of chronic kidney disease and stress. While challenges remain, these advances reaffirm the growing utility of sweat sensors in tracking dynamic physiological trends with increasing precision and clinical relevance.

Integration of capabilities for iontophoretic transdermal delivery of cholinergic agents (e.g., pilocarpine, carbachol) into wearable sweat analysis platforms provides a means for triggering localized sweat gland activation. This scheme builds on well-established methods for capturing samples of sweat for assessments of chloride concentration in CF diagnosis. Typically, the stimulation uses hydrogels containing pilocarpine/carbachol with a 0.1–0.25 mA current, followed by microfluidic collection and multiplexed ion, metabolites, and nutrients analysis.^197,198^ These combined stimulation and sensing devices support monitoring of daily health parameters, including diet, supplement intake, and stress levels. Some studies suggest an ability for continuous sweat glucose monitoring in perspiration, as an alternative to blood glucose assessment.^199^ Additional published data demonstrate the ability to track the metabolism of nutrients and vitamins in sweat after 24 h of supplement intake.^197^ Also, by analyzing hormones such as cortisol,^200,201^ sweat sensors can provide insights into stress response and overall well-being. In clinical contexts, such technologies can help to diagnose and manage various diseases. For example, CF wearable devices can quantify chloride and sodium concentrations using either colorimetric or electrochemical approaches.^197,202–204^ Tracking glucose and uric acid levels in sweat is relevant for diabetes and gout.^126,205^ Additional possibilities are in monitoring for cardiac conditions, where the combined tracking of sweat loss biomolecule concentrations can complement measurements of other vital signs (blood pressure, heart rate, etc.) for a comprehensive view of physiological state.^28^

It is worth recalling the challenges in using sweat as a matrix for quantitative and real-time detection. First, sweat availability is related to the patient’s transpiration, which can be passive or induced. The passive one requires specific environmental conditions (high temperature and humidity), which are not always possible to obtain. The active transpiration typically relies on drugs and direct or reverse iontophoresis, which may be difficult to implement. A second issue is sweat rate normalization, as the rate of sweating of a human changes over time. This influences the concentration of any analyte in sweat, creating an artifact in the measurements. For example, the concentration of a metabolite such as lactic acid, passively generated during physical exercise, is initially extremely high due to the reduced amount of sweat produced at the beginning of physical activity. A few minutes afterward, the volume of sweat drastically increases; thus, the lactic acid concentration measured results are much lower.^206^

Wearability is also crucial for sensors applied to sweat. Most skin-conformable sensors are fabricated using synthetic polymers due to their wide range of compositions and ease of functionalization. However, it may be advantageous to explore natural, skin-substitute polymers such as microbial nanocellulose (MNC), which is also used in wound dressings. Advantages of MNC include its strong adhesion to skin without the need for additional adhesives and its potential use in implantable devices, as it resists degradation in the human body while maintaining structural stability. Silva et al.^177^ demonstrated the use of MNC as a substrate for screen-printed carbon electrodes (SPCEs). This concept is schematically illustrated in Figure 5, which highlights the suitability of self-adherent MNC for the noninvasive detection of biomarkers in sweat. The zoom of Figure 5 also shows a photograph of a SPCE-based electrochemical sensor deposited on an MNC substrate adhered to the human skin. A significant enhancement in analytical performance for the detection of cadmium (Cd^2+^), lead (Pb^2+^), uric acid, and 17β-estradiol was obtained by pretreating the surface of the SPCEs in diluted sulfuric acid, leading to faster electron transfer rates and lower limits of detection (LODs). The reason for such an enhancement was related to the creation of oxygen groups on the carbon surfaces, which increased wettability and hydrophilicity.

**Figure 5:**
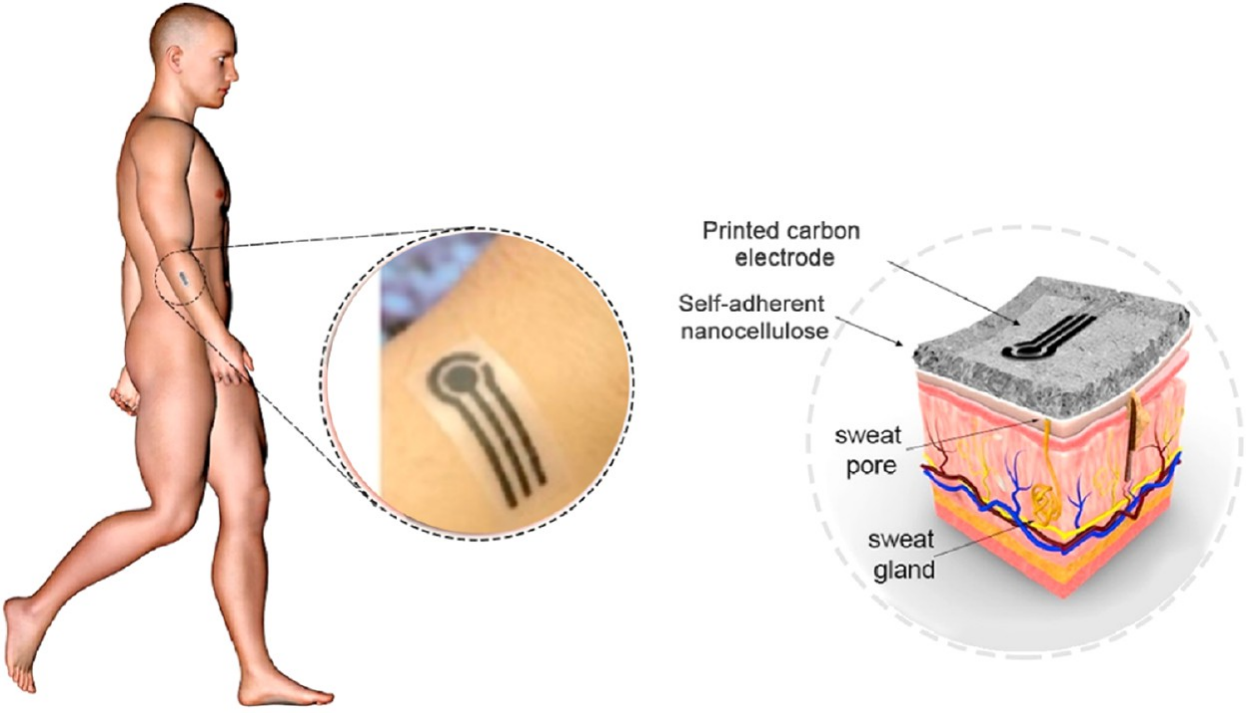
(Left) Schematic diagram illustrating a skin-adhered sensor fabricated with a screen-printed carbon electrode (SPCE) on a microbial nanocellulose (MNC) substrate. The zoom displays an actual picture of the device adhered to the human skin. The pictorial representation on the right demonstrates that the self-adherent MNC is suitable for a wearable skin sensor capable of detecting biomarkers in sweat. Adapted from Silva et al.^177^ © 2020 Elsevier.

Detection of the protein metabolite urea, a biomarker of renal function, is relevant for patients at risk of heart failure. Ideally, urea levels should be monitored continuously, which can be achieved using wearable sensors to detect urea in sweat.^207^ Urea levels in sweat can be correlated with those in blood, but their electrochemical detection is influenced by sweat pH, which varies among individuals. This limitation was addressed by Ibáñez-Redín et al.,^207^ who developed wearable potentiometric biosensors on SPCEs capable of quantifying both urea concentration and pH. The biosensor architecture and fabrication procedure are illustrated schematically in Figure 6Ai, showing a reference electrode and two working electrodes (one for pH and one for urea). The dual sensor included polyaniline ink, urease bioink, and a polyvinyl chloride (PVC) membrane. The working electrode for urea featured a polyaniline (PANI)-based ink that detected pH increases resulting from urea hydrolysis by urease. The enzyme was immobilized using a chitosan-containing ink, which helped preserve its activity. Data from the potentiometric measurements are presented in Figure 6Aii-iv, where a change in potential occurs as urea concentration increases. The biosensor presented a rapid response, with an incubation time of only 5 min.

**Figure 6:**
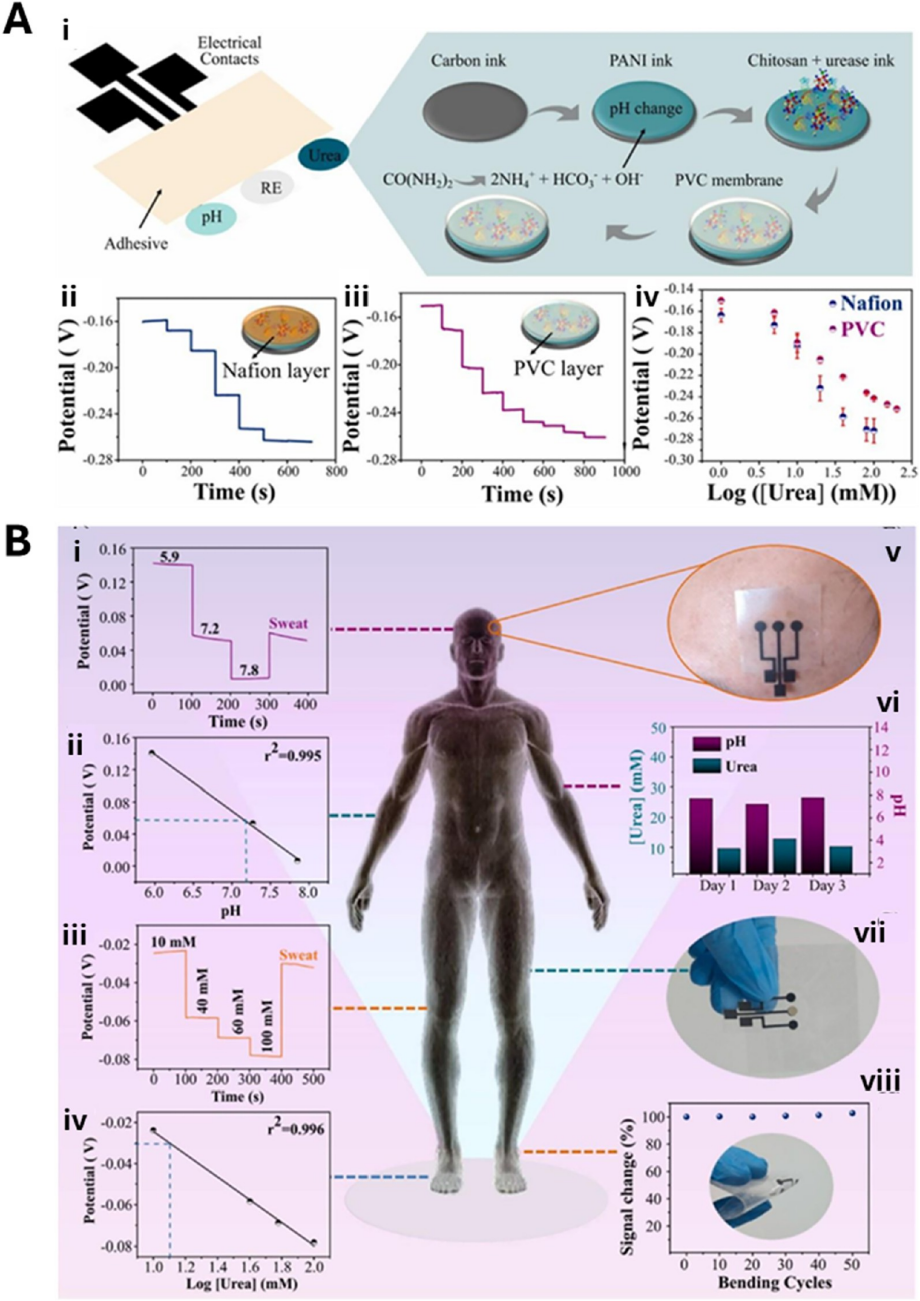
(A) (i) Fabrication procedure for potentiometric sensors that could detect urea and measure pH in sweat. (ii) Change in potential as increasing concentrations of urea were added for the sensor with the PVC membrane. (iii) Same as in (B) with a sensor containing a Nafion membrane. (iv) Calibration curves for urea detection using the two types of sensor (with Nafion and PVC membranes). (B) (i) Potentiometric response of the sensor at distinct pHs, which led to the calibration plot in (ii). (iii) Sensor response at different urea concentrations, from which the calibration plot was obtained (iv). (v) Photo of the sensor attached to the forehead of a volunteer. (vi) Urea and pH measurements at 3 different days with sweat of the same volunteer. (vii) Photo of the wearable device. (viii) demonstration that the wearable device could be bent multiple times without compromising the potentiometric response. Reproduced with permission from Ibáñez-Redín et al.^207^ © 2023 Elsevier.

The wearable device’s ability to detect urea in a real-world scenario was demonstrated by attaching the sensor to a volunteer’s forehead. The results and corresponding photos are presented in Figure 6B. The potentiometric response and calibration plots for urea and pH in the volunteer’s sweat sample are shown in Figure 6Bi-iv, while a photo of the sensor on the forehead is shown in Figure 6Bv. Having determined the pH of the sweat sample as 7.2, the urea concentration could be precisely determined using the corresponding pH value in the calibration curve. A urea concentration of 10.9 mM, typical of healthy individuals, was obtained and remained stable over 3 days, as shown in Figure 6Bvi. The epidermal device, depicted in Figure 6Bvii, includes an opening to enable direct contact between the electrodes and the skin. It demonstrated flexibility, as it could be bent multiple times without affecting the potentiometric response, as evidenced by Figure 6Bviii.

A plastic glove used to determine therapeutic drugs and biomarkers in human sweat samples was developed by Raymundo-Pereira et al.^208^ The electrochemical sensor array was screen-printed onto four fingers of the glove, as in the picture of Figure 7A, each of which was designed for a specific analyte. The sensor on the index finger was functionalized with carbon black to quantify uric acid to a LOD of 1.37 × 10^–6^ mol L^–1^. The middle and ring finger sensors were coated with Printex Carbon to detect paracetamol and paroxetine with LODs of 2.47 × 10^–7^ and 4.93 × 10^–7^ mol L^–1^, respectively. Detection of the ethinylestradiol hormone was carried out with a pretreated screen-printed carbon electrode on the little finger, with an LOD of 9.35 × 10^–7^ mol L^–1^. Besides the choice of sensing materials, measuring conditions in differential pulse voltammetry (DPV) were carefully chosen to yield the high sensitivity and selectivity achieved with the glove-embedded sensors.

**Figure 7:**
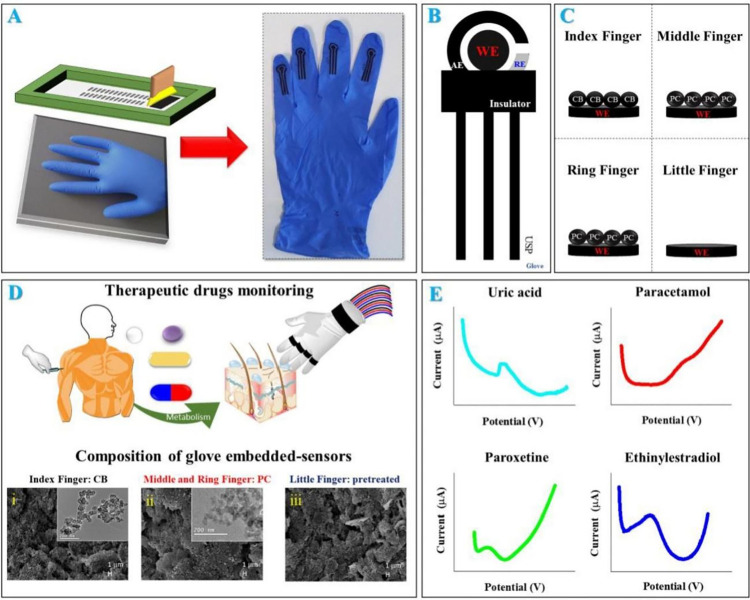
(A) Fabrication process of the screen-printed sensors on a plastic glove. (B) Schematic representation of the 3-electrode sensor where the auxiliary (AE), reference (RE), and working (WE) electrodes are marked. (C) The working electrodes varied depending on the analyte to be detected. (D) Schematic representation of the concept of therapeutic drug monitoring as quantification in sweat may indicate how much of a given drug has been metabolized by the patient. Also shown in this part are scanning electron microscopy images of the working electrodes on the different fingers. (E) Representative signals in DPV for the 4 analytes detected upon touching human sweat on the skin. Reproduced with permission from Raymundo-Pereira et al.^208^ © 2022 Elsevier.

Adequate signal acquisition and transmission are essential for the development of lightweight and compact wearable devices that do not hinder the natural movements and daily activities of the patient (see Section 9 for further details). In sweat-based sensing platforms, wireless communication technologies such as near-field communication (NFC) and Bluetooth are commonly employed to enable data transmission to external readers or smartphones. Integrating wireless communication and signal-conditioning functionalities into compact electronic modules allows a significant reduction in system size and system complexity compared to traditional wired readout architectures. Indeed, a fully printed wearable system was developed for the detection of copper ions in sweat, correlated to the diagnosis of Wilson’s disease. The printed microfluidic devices were connected to an ad hoc Bluetooth system for the wireless readout with a customized smartphone app. The readout device was also designed with the possibility of performing reverse iontophoresis to get a sweat sample in adverse environmental conditions. A multilayer microfluidic passive system (capillary-based) was designed to (i) maximize the area of collection of the sweat, (ii) take the sample to the screen-printed carbon device for the copper measurement by SWASV, (iii) let the sample flow through interdigitated electrodes to measure its conductivity, and (iv) let the sample flow along a channel surrounded by two parallel long electrodes to measure its volume by the change of impedance among them and taking advantage of the measured conductivity. Then, the sweat rate would be defined and used for the copper concentration normalization. The microfluidic circuit, which was built using patterned biadhesive plastic tape, ended in a sponge, so that the drying of the sweat in the channel after the measurements was granted, allowing for new tests and keeping the capillary effect moving the sample onto the electrodes. The system showed a LOD of 396 ppb, a linear range up to 2500 ppb, and a sensitivity of 2.3 nA/ppb in artificial sweat.

With regard to commercially available devices, Eccrine Systems was one of the earliest companies focused on wearable sweat sensors for diagnostic purposes via electrochemical approaches. They established important technical and clinical benchmarks but ultimately ceased operations. Another company, Epicore Biosystems, launched shortly after Eccrine Systems, found commercial success in comparatively simple devices that use microfluidics and colorimetry for monitoring sweat rate, total sweat loss, and electrolyte concentration. The Gx Sweat Patch, launched in the spring of 2021, was the first such product, through a partnership with Gatorade. This thin, flexible microfluidic device pairs with a graphical user interface that operates on a smartphone to capture images that allow for quantitative measurements of local sweat loss and electrolyte concentration, the latter through colorimetric measurements of the concentration of chloride. The result is a personalized recommendation for rehydration following physical exertion in sports training or competition, with specific guidance on replenishment of lost water and electrolytes.^209^ Advanced versions of this technology support wireless electronics for continuous measurements without the need for visual access to the device, designed for applications in worker safety associated with the oil/gas, construction, manufacturing, mining, and agricultural industries. Specifically, data is passed on to a mobile app and cloud dashboard, allowing safety managers to intervene before heat stress occurs, thereby improving workforce safety and performance.^27^ Another key player in this space, Nix Biosensors, reports a reusable hydration biosensor designed primarily for endurance athletes and military applications, providing hydration recommendations with historical and predictive data. Other emerging efforts in commercialization are at the company Persperion, where the focus is on sweat for inferring glucose concentrations in blood by electrochemical detection of glucose levels through sweat collected from the fingertips.^210^ Persperity Health is also advancing wearable sweat-sensing technology focused on women’s health. Their platform uses iontophoresis-based on-demand sweat induction to enable real-time assessment of key reproductive hormones like estradiol and progesterone.^102^

As these examples illustrate, the commercial landscape of sweat collection and analysis technologies has evolved rapidly over the last several years, transitioning from simple absorbent pads for collection and separate analysis using conventional techniques to integrated microfluidic systems capable of real-time monitoring in a wearable format. The potential is to transform noninvasive, continuous health monitoring practices and disease diagnosis from wearable devices that track biophysical parameters such as heart rate, respiration rate, physical activity, and temperature to those that include diverse biochemical markers. The result may lead to proactive health management strategies, ultimately improving overall well-being in a cost-efficient manner, applicable across various populations and in resource-constrained areas of the world.

### 3.2 Saliva

Saliva is a complex biofluid that can provide valuable physiological and biochemical information about both local and systemic health. It is secreted primarily by the parotid, submandibular, and sublingual glands and contains a wide range of biomarkers, including electrolytes (Na^+^, Cl^–^, Ca^2+^), small molecules (glucose, lactate, uric acid, cortisol, and urea), proteins (enzymes, immunoglobulins, mucins), nucleic acids, and microbial metabolites.^211,212^ As saliva originates partly from the filtration, diffusion, or active transport of blood plasma, it carries molecular signatures that reflect not only oral but also systemic physiological states, making it useful for tracking health status in real time.^213^ Unlike blood, saliva can be collected repeatedly without pain or specialized equipment, to monitor hormone regulation, immune activity, metabolic function, and stress responses.^214^ Salivary biomarkers have been linked to numerous conditions such as diabetes, oral cancer, cardiovascular diseases, stress, and infectious diseases.^215–219^

Metabolism “begins in the mouth,” and saliva plays an active biochemical role in early digestion. Enzymes such as α-amylase, which initiates starch breakdown, and lingual lipase, which begins lipid digestion, transform the oral cavity into a dynamic metabolic environment immediately after food enters the mouth.^214^ The salivary flow rate, electrolyte content, and enzyme activity are influenced by dietary intake, stress, circadian rhythms, and autonomic nervous system activation, factors that make saliva a rich medium for monitoring metabolic physiology. Saliva also provides clinically useful information about oral hygiene and dental health. Its buffering capacity, antimicrobial proteins, and mineral composition directly regulate enamel demineralization and plaque formation. Simple measurements such as salivary pH can offer rapid insights: low pH is associated with a higher risk of caries and enamel erosion, whereas high pH and increased buffering may indicate calculus formation or altered microbiome composition.^220^ Because pH strips and miniaturized pH electrodes are easy to integrate into sensors, salivary pH remains one of the most accessible and informative biomarkers for everyday oral-health monitoring.

Engineering wearable or intraoral biosensors for saliva presents significant challenges. Saliva contains abundant proteases, amylases, and lipases that can degrade analytes or foul sensor surfaces, reducing sensor stability.^221^ The mouth is also chemically unpredictable because eating, drinking, toothpaste, and mouthwash introduce rapid fluctuations in pH, ionic strength, and viscosity. Moreover, the oral cavity is mechanically harsh, exposing devices to constant moisture, shear forces, and temperature changes. Electronics must be encapsulated in biocompatible, waterproof materials while still enabling wireless communication and stable sensing performance. Additionally, a stable sample acquisition should be ensured, accounting for variations in salivary flow rate and minimizing interference from food residues and microbial contamination.^222^ The flow and viscosity of saliva can affect analyte concentration, requiring integrated microfluidic systems for controlled sampling and normalization. Comfort and user acceptance are additional obstacles. Oral devices must not interfere with speech, swallowing, or breathing. Nevertheless, several platforms have shown strong promise. Smart mouthguards, originally developed for athletes, have been adapted to measure analytes such as glucose, lactate, and uric acid during real-world activities.^223,224^ Orthodontic retainers offer discrete, long-term placement and can host microfluidics and electrodes with minimal discomfort.^221^ Additional information on mouthguards as saliva-based biosensors can be found in Section 5.4. Ultrathin tooth-mounted “tattoo” sensors, including graphene or RF-based enamel patches, demonstrate the potential for invisible, continuous monitoring of oral chemistry and bacteria.^225^ For infants and pediatric care, pacifier-based biosensors can continuously track electrolytes and metabolites, providing stress-free sampling.^226,227^ Lollipop-based microfluidic systems (“lab-on-a-lollipop”) encourage natural saliva production and enable easy point-of-care testing, especially in children.^228^

The composition and characteristics of saliva depend strongly on the mode of secretion, which can be classified as unstimulated (basal) or stimulated. Both types have distinct biochemical profiles, flow rates, and implications for biomarker detection, which are relevant for the design and interpretation of wearable biosensors.^229^ Unstimulated saliva is continuously produced mainly by the submandibular and sublingual glands, with a flow rate of ∼0.3–0.5 mL/min.^212^ It reflects the body’s resting physiological state and contains higher concentrations of proteins, hormones, and small molecules such as cortisol, IL-6, and oxidative stress markers. Because it is less affected by transient *stimuli*, unstimulated saliva provides a reproducible biofluid for longitudinal monitoring and is preferred for chronic or systemic biomarker detection. However, low flow and high viscosity may complicate sample handling and limit the volume available for analysis, particularly in patients with dry mouth. A collection period of at least 5 min is generally recommended to ensure representative sampling.^229^

The analysis of unstimulated saliva can be achieved through intraoral wearable sensors that maintain direct contact with the oral cavity. One example of an intraoral device using basal saliva is the biosensor reported by García-Carmona et al.^227^ The system was designed for infant glucose monitoring, integrating a Prussian Blue-modified carbon electrode functionalized with glucose oxidase (GOx) in a wireless, baby-safe platform. The design uses the infant’s natural sucking motion to draw saliva through a rectifying channel toward an external electrochemical cell, isolating all electronic components from the mouth. The unidirectional saliva flow enabled reproducible measurements without backflow contamination, while the external cell configuration avoided direct contact of electrode materials with oral tissue. The biosensor demonstrated a strong correlation between salivary and blood glucose in diabetic adults, confirming saliva’s diagnostic relevance and illustrating how unstimulated saliva can be harnessed for continuous, noninvasive biochemical monitoring in sensitive populations such as neonates. Similarly, Ichikawa et al. developed a mouthguard-type optical sensor for monitoring salivary turbidity as an indicator of oral hygiene.^230^ The system integrates a 405 nm LED and a phototransistor within a double-layer mouthguard, sealed to protect electronics from moisture and powered by a wireless Bluetooth module. By tracking light transmittance through the thin salivary film formed naturally in the mouth, the device could continuously quantify turbidity values across 1–4000 FTU, showing strong agreement with benchtop spectrophotometric measurements. This design demonstrates the potential of optical readouts for continuous oral monitoring using naturally secreted saliva without any stimulation or external intervention.

Stimulated saliva is secreted upon mechanical or gustatory stimulation (e.g., chewing, citric acid), predominantly from the parotid glands. The flow rate increases up to 1–3 mL/min, while protein content decreases and pH and electrolyte levels (Na^+^, Cl^–^, HCO_3_^–^) rise.^212^ This makes stimulated saliva less viscous and easier to collect, facilitating faster analyses and better reproducibility in subjects with low basal secretion. However, its composition varies with the stimulation method, which can influence biomarker concentration and sensor response. Standardizing the stimulation type and collection time is thus essential for accurate comparison and calibration across studies. Wearable systems that sample stimulated saliva offer additional design flexibility and safety advantages, such as reducing risks associated with prolonged intraoral contact. However, as the analyte concentrations in stimulated saliva are typically more dilute, higher sensor sensitivity and calibration strategies are required. A notable example of this approach is the paper-based microfluidic mouthguard sensor reported by de Castro et al.^231^ In this semiwearable device, stimulated saliva obtained after a brief mechanical activation was introduced into a microfluidic paper analytical device (μPAD) integrated into a 3D-printed mouthguard. The μPAD incorporated colorimetric detection zones for glucose and nitrite, achieving detection limits of 27 and 7 μM, respectively. This work highlighted the practicality of low-cost, disposable colorimetric assays embedded in oral accessories, representing a bridge between conventional sampling and fully integrated wearables.

More recently, saliva-sensing dental floss has been introduced as an on-demand platform for stress assessment. During flossing, mechanical action stimulates saliva secretion and simultaneously guides it through the floss’s thread-based microfluidic pathway toward a flexible electrochemical sensor. The device employs a MIP deposited onto porous laser-engraved graphene, enabling highly selective cortisol recognition with ultralow detection limits and strong correlation to ELISA.^232^ Some of such saliva (bio)­sensor formats are illustrated in Figure 8.

**Figure 8:**
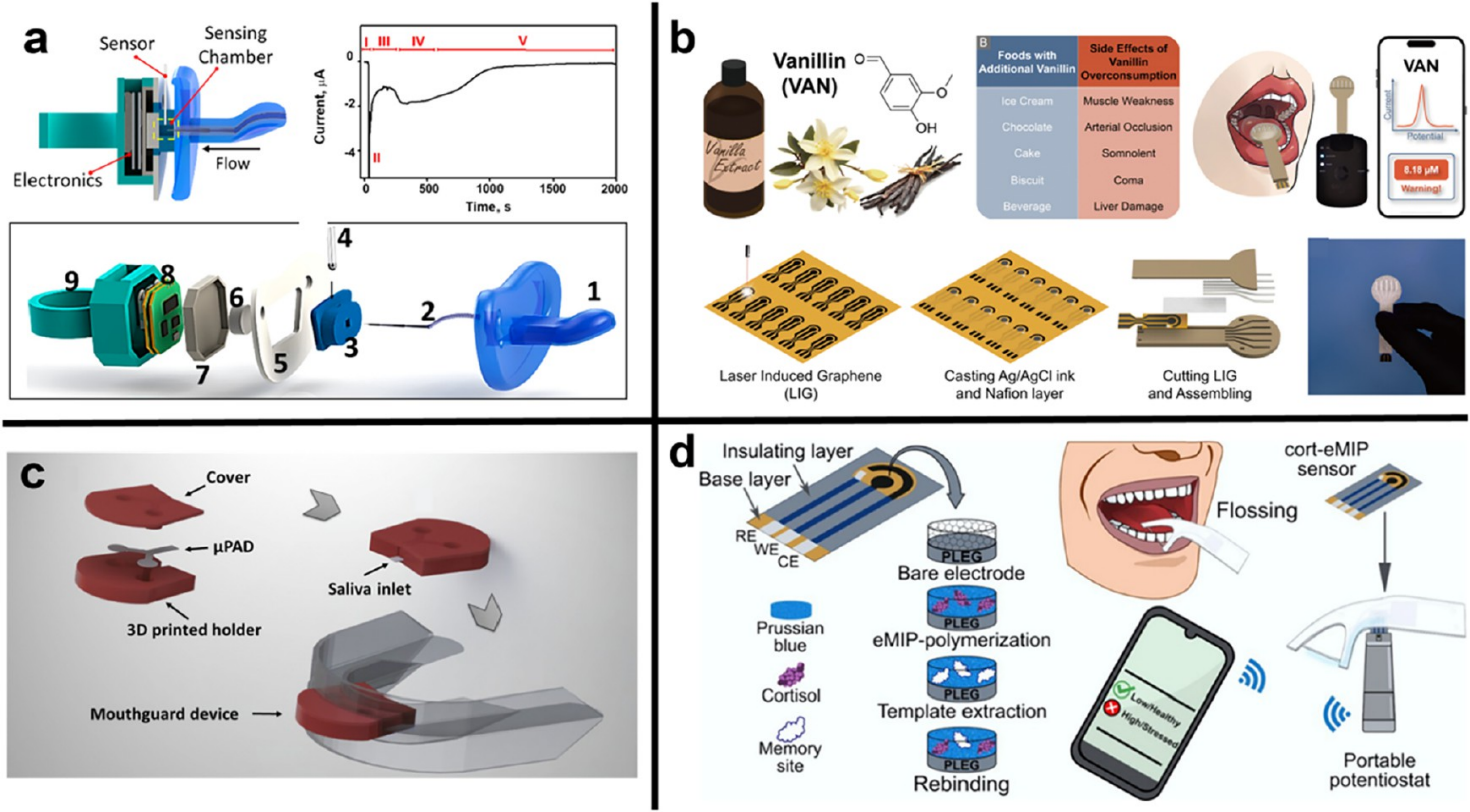
Wearable biosensing platforms for salivary analysis. (a) Pacifier-based biosensor for unstimulated salivary glucose monitoring. Top: On-body demonstration and representative amperometric response. Bottom: Schematic of the fluidic pathway, showing passive saliva pumping during sucking and enzymatic glucose detection via GOx/Prussian Blue in an external sensing chamber. Exploded views illustrate the integration of the sensor, chamber, and electronics into a baby-safe pacifier platform. Reproduced from García-Carmona et al.^227^ © 2019 American Chemical Society. (b) Wearable electrochemical biosensing based on laser-induced graphene (LIG) electrodes for molecular detection in saliva. Top: motivation for detecting vanillin as a food additive. Bottom: fabrication steps and the hand-held reader for on-body measurement. Reproduced with permission from Ma et al.^228^ © 2024 Royal Society of Chemistry. (c) μPAD-integrated mouthguard device for colorimetric detection in saliva. The assembly consists of a 3D-printed holder, a removable microfluidic paper analytical device (μPAD), and a top cover. Reproduced with permission from de Castro et al.^231^ © 2019 Springer Nature. (d) Thread-based microfluidic dental floss sensor for stimulated salivary cortisol detection. Left: fabrication of the porous laser-engraved graphene (PLEG) electrode, electropolymerized molecularly imprinted polymer (eMIP), and template extraction steps forming cortisol-selective binding sites. Right: on-demand cortisol sensing during flossing, with wireless readout to a portable potentiostat and smartphone interface for stress assessment. Reproduced from Sharma et al.^232^ © 2025 American Chemical Society.

Future efforts are focused on improving signal stability, antifouling performance, and real-time calibration. The oral cavity presents unique challenges, including variable pH and the presence of enzymes and bacteria that may degrade the sensing elements. Strategies such as applying coatings, implementing ratiometric sensing, and monitoring the salivary flow rate in situ are being developed to address these issues. The transition from single-analyte enzymatic devices to multiplexed and integrated intraoral systems holds great promise for real-time, personalized health monitoring. As wireless, biocompatible microelectronics, materials, flexible sensing technologies, and microfluidic control continue to evolve, next-generation saliva-based wearable biosensors are poised to play a key role in continuous biochemical monitoring and early disease detection.

### 3.3 Tears

The eyes can provide important physiological signals, including intraocular pressure and temperature, besides allowing a minimally invasive analysis of diverse biomarkers in tears. These include electrolytes (K^+^, Na^+^, Cl^–^, HCO^–^, Mg^2+^, Ca^2+^), proteins, lipids, and small molecules (e.g., glucose, urea, lactate). These biomarkers carry information not only from the eyes but from the entire body, as tears are created from the filtration of blood plasma. The main challenge, in this case, is to detect biomarkers precisely in tears and correlate their contents with those in blood.^233^ Biomarkers in tears have already been associated with different types of cancer, Parkinson’s disease, multiple sclerosis, diabetes, ocular allergies, glaucoma, and others.^234^

The analysis of basal tears (i.e., the protective layer of tears that lubricate, nourish, and clean the eyes) is of interest due to the higher analyte content and greater sample reproducibility when compared to stimulated tears. This can be performed, for example, through direct contact of the analytical device and the eyes, including contact lenses, which will be further discussed in Section 5.3. Park’s research group^235^ developed a wireless, soft, smart contact lens for noninvasive detection of glucose in tears. The wearable sensor comprised functional devices (rectifier, light-emitting diode (LED), glucose sensor) that collected tears and performed a colorimetric reaction. A stretchable, transparent conductor was used for data transmission, interpretation, and display. This platform for personalized eye health management was demonstrated with *in vivo* tests in a rabbit wearing the contact lens, including monitoring of temperature on the rabbit’s eye. A smart contact lens was also developed to correlate glucose in tears and blood, illustrated in Figure 18.^236^ The key components of the lens include a glucose biosensor, an NFC chip, and an antenna to transmit the tear glucose data via an app. This work is also relevant because it strictly tracks the effects of wearing the prepared contact lens without interference from other causes, such as pH value. The tears were collected by an external mechanical stimulation to the conjunctiva while wearing the devices. In addition, the designed glucose biosensor ensures long-duration continuous measurements of tear glucose. This enabled the precise identification of the lag time in all of the tested subjects (rabbit, dog, and healthy and diabetic humans) for the in-depth correlation analysis between tear glucose and blood glucose.

Wearable devices that analyze stimulated tears are also an interesting option because of the lower chances of promoting infections or the leakage of metals or other harmful substances into the eye. In this case, however, the use of more sensitive devices is important due to the dilution of the analytes as well as the monitoring of the generated tear volume. Sempionatto et al.,^237^ for example, developed modified eyeglasses for the real-time and noninvasive detection of glucose, ethanol, and vitamins in tears (Figure 9). In this case, tears were stimulated using a menthol stick and captured through a superhydrophilic microfluidic device attached to the nose bridge pad of the eyeglasses. Carbon electrodes modified with Prussian blue and containing GOx or alcohol oxidase (AOx) were applied for the detection of glucose and ethanol, respectively. Bare carbon electrodes were used for the simultaneous detection of vitamins B2, C, and B6 through square-wave voltammetry (SWV). The devices were applied for multiple rounds of analyte monitoring, showing great reproducibility and responsiveness throughout the full day.

**Figure 9:**
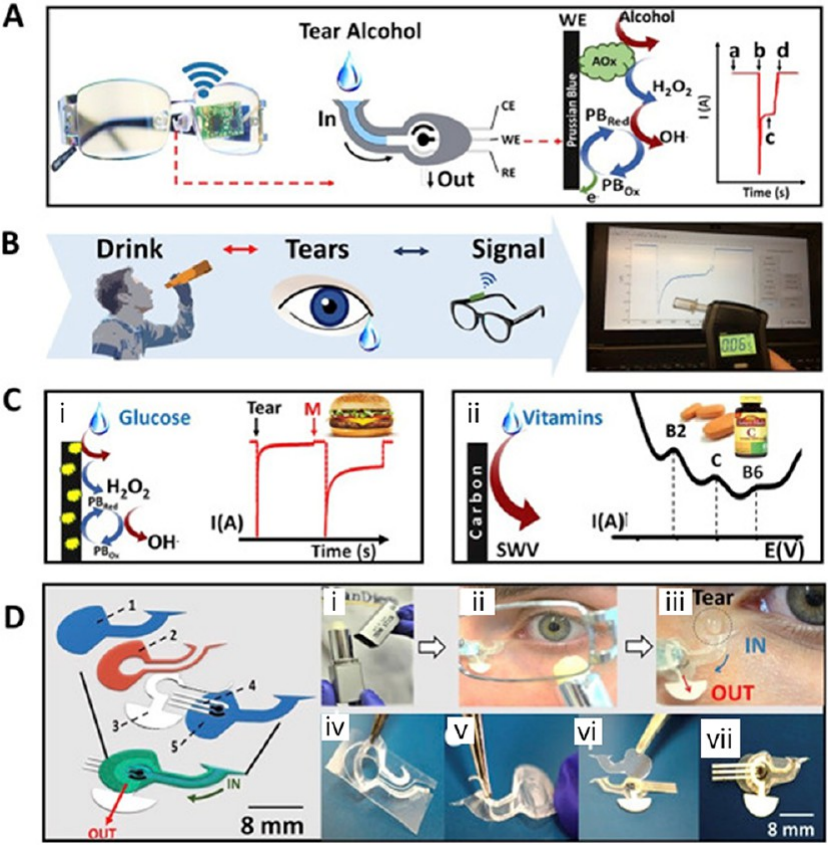
Wearable device based on modified eyeglasses for the detection of glucose, ethanol, and vitamins in stimulated tears. (A) Pictures of the analytical device attached to the nose-bridge pad of the eyeglasses, and the portable potentiostat linked to the side of the wearable. Scheme showing the tears flow on the device, the enzymatic detection mechanism for ethanol, and the overall form of the obtained signal. (a) Dry device; (b) tears enter the device; (c) stable signal related to the analyte concentration; (d) tears leave the device, making it ready for the next measurement. (B) When performing ethanol detection, the signals were recorded after the volunteer had a drink. The device was calibrated through the simultaneous usage of a breathalizer. (C) Detection mechanism of: (i) glucose using GOx and a PB-modified carbon electrode; (ii) Vitamins using SWV and a bare carbon electrode. (D) Assembly of the microfluidic device. 1—hydrophilic membrane; 2—double-sided tape; 3—filter paper; 4—screen-printed electrode; 5—hydrophilic membrane. The stimulation of tears using the menthol stick is shown in i–iii, while pictures of the assembly of the wearable device are shown from iv-vii. Reproduced with permission from Sempionatto et al.^237^ © 2019 Elsevier.

Physical parameters can also be an important source of health information for the eyes. For example, an elevated intraocular pressure may be an indication of glaucoma, an irreversible ocular disease affecting 80 million people worldwide.^238,239^ Many glaucoma patients miss the optimal time for treatment because of the lack of frequent intraocular pressure monitoring. Continuous monitoring was made possible with a graphene-based smart contact lens.^240^ The lens had a high sensitivity of 1.0164 mV mm Hg^–1^ on a silicone eye, and 3.166 mV mm Hg^–1^ *in vitro* on porcine eyes, displaying remarkable linearity. In a later work, a smart closed-loop system that combines a Ti_3_C_2_T_*x*_ MXene-based soft contact lens sensor, wireless data transmission units, display, and warning components to realize continuous and nondestructive pressure monitoring was developed.^241^ The Ti_3_C_2_T_*x*_ contact lens had a high sensitivity of 7.483 mV mm Hg^–1^ with superior linearity on silicone eyeballs, and excellent biocompatibility when worn onto the rabbit eyes. The intraocular pressure monitoring/real-time display in the smartphone was also realized via the integration of the smart contact lens with Bluetooth modules. An SCL can also function as a theranostic device by delivering a drug to decrease the intraocular pressure when a threshold value is reached. Kim et al.^242^ reported on a theranostic SCL (Figure 18, Physical signal) consisting of a sensitive gold hollow nanowire sensor, a flexible drug delivery system, wireless power and communication systems, and an integrated circuit chip for pressure monitoring and control. These functionalities were demonstrated in *in vivo* experiments on glaucoma-induced rabbits, thus highlighting SCLs for POC ophthalmic diagnosis and disease management.

The main challenges for sensing biomarkers in tears are the small sample volumes combined with their rapid evaporation. The stimulation of tears can be difficult, and the obtained biofluid volume is commonly inconsistent as well as the concentration of analytes. However, these remain valuable for containing biomarkers for important diseases and exogenous compounds. Also, their characteristics facilitate analytical processing, such as transparency, lower cellular content, and fewer interfering components (compared to blood).

### 3.4 Wound Fluid

Wound biofluids, such as exudate and ISF, are important in the wound healing process and are rich in biomarkers.^243^ Chronic wounds, such as those caused by diabetes or prolonged pressure, pose significant challenges for traditional management due to their intricate healing processes and heightened vulnerability to infection.^244,245^ The composition of the wound fluid depends on the wound type (acute or chronic) and the phase of healing. For example, pH levels serve as key indicators, since chronic wounds typically exhibit an alkaline pH (7.15–8.93), indicating inflammation or infection, while healing wounds tend to be more acidic.^246–249^ Similarly, inflammatory cytokines such as TNF-α, IL-6, and IL-8 are elevated during the inflammatory phase of chronic wounds, signifying heightened immune activity and delayed healing.^243,250–252^ Elevated glucose levels in wound exudate may indicate poor metabolic control in diabetic wounds, while reduced levels may suggest bacterial consumption, pointing to a potential infection.^253–257^ Lactate, a byproduct of anaerobic metabolism, serves as a marker for tissue hypoxia and angiogenesis, providing insights into oxygenation and metabolic activity.^258^ Other biomarkers, such as reactive oxygen species (ROS) and uric acid, are associated with oxidative stress and inflammation; elevated levels may indicate excessive immune responses or impaired tissue repair.^259–262^ Additionally, monitoring the bacterial load, particularly the presence and concentration of specific pathogens, helps assess the infection severity and guide appropriate treatment strategies.^263,264^

Figure 10 provides an overview of wearable sensor technologies for wound monitoring. These sensors commonly rely on noninvasive or minimally invasive sampling strategies, focusing on direct biofluid collection from the wound site.^265–267^ The multiplex sensing depicted in Figure 10B enables simultaneous monitoring of various biomarkers, permitting the assessment of wound healing and infection status.^268^ Wound monitoring can be performed with electrochemical and optical transduction techniques^50,243,263,268–273^ to quantify biomarkers with high sensitivity and specificity. Electrochemical sensors are used due to their sensitivity, compact size, and versatility,^265^ mostly being amperometric or potentiometric, as illustrated in Figure 10C. Cytokines and bacterial loads have been detected with aptamer-functionalized electrochemical sensors (Figure 10D).^243^ These sensors used graphene-gold nanoparticle composites to enhance electron transfer and signal fidelity and have been applied to monitor conditions associated with venous ulcers. Figure 10E shows the measured pH related to inflammation and infection, cytokine concentration related to immune activity, and bacterial load. Using such a multiplex sensor system permits identifying infections early, thus enabling timely intervention.

**Figure 10:**
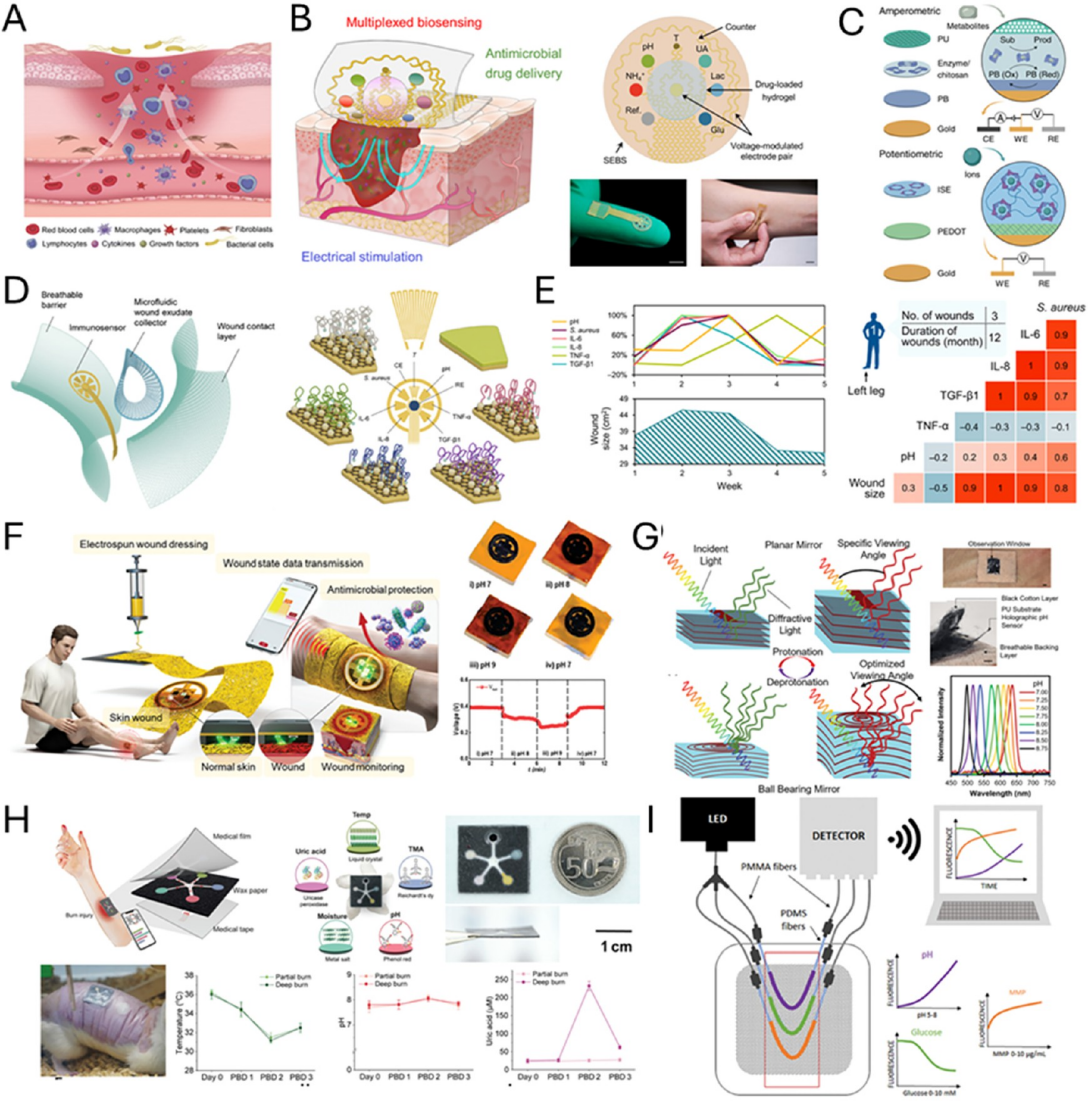
Wearable sensor technologies for wound monitoring. (A) Microenvironments and biomarkers in wound biofluids for diagnosing and monitoring the wound healing status. Reproduced from Gao et al.^243^ Available under a CC BY 4.0 license. ©2021 American Association for the Advancement of Science. (B) Multiplexed biosensor for simultaneous monitoring of multiple wound biomarkers. Reproduced from Sani et al.^268^ Available under a CC BY 4.0 license. © 2023 American Association for the Advancement of Science. (C) Mechanisms of electrochemical sensors: amperometric enzymatic electrode sensors and potentiometric ion-selective sensors utilizing metal electrodes. Reproduced from Sani et al.^268^ Available under a CC BY 4.0 license. © 2023 American Association for the Advancement of Science. (D) Aptamer-functionalized electrochemical sensors with microfluidic wound collectors. Reproduced from Gao et al.^243^ Available under a CC BY 4.0 license. © 2021 American Association for the Advancement of Science. (E) Clinical validation of electrochemical sensor for wound monitoring. Reproduced from Gao et al.^243^ Available under a CC BY 4.0 license. © 2021 American Association for the Advancement of Science. (F) Colorimetric pH wound dressing integrating an optoelectronic sensor with green LED and PD for rapid detection and NFC wireless communication of wound status. Reproduced with permission from Cho et al.^249^ © 2024 John Wiley & Sons. (G) High-resolution pH colorimetric wound dressing leveraging fluorescent expression changes for precise diagnostics. Reproduced from Zhang et al.^281^ Available under a CC BY 4.0 license. © 2024 John Wiley & Sons. (H) Wax paper-based dressing sensor enabling colorimetric diagnostics for five key biomarkers—uric acid, TMA, pH, temperature, and moisture—designed for advanced burn care management. Reproduced from Zheng et al.^259^ Available under a CC BY-NC license. © 2023 American Association for the Advancement of Science. (I) Colorimetric polymeric fiber-based wound fluid sensor integrating biomarkers for pH, glucose, and MMP, offering a multifunctional design for simultaneous wound condition diagnosis. Reproduced with permission from Giovannini et al.^285^ © 2024 John Wiley & Sons.

Wound monitoring can also be done with optical sensors, particularly colorimetric ones. Recent advancements include multifunctional wound dressings incorporating pH-sensitive dyes or natural extracts such as turmeric,^135,274^ cabbage,^275,276^ and blueberries.^277^ Traditional methods included pH sensors embedded in mammary glands or composite pH sensors fabricated with flexible polymers.^278,279^ However, these approaches often constrain patient mobility and require the involvement of medical professionals with specialized expertise, rendering them unsuitable for wearable diagnostic devices. Colorimetric sensors have been reported for lactic acid and glucose, which, as previously mentioned, are very relevant to monitor in wound exudates.^280,281^ Furthermore, fluorescent materials have been integrated into devices, aiding in the monitoring of the progression and pathological states of infected wounds.^257^ These materials show responses to glucose^282^ and bacterial infection,^283^ for example, providing a visual indication of wound deterioration or healing. Diagnostic and therapeutic functions can be combined by incorporating drug layers into wound dressings.^274,284^

Figure 10F illustrates a wound dressing with curcumin, a natural extract from turmeric, as a colorimetric indicator.^249^ The absorbance and reflectance of this multifunctional dressing are affected by structural changes in curcumin under different pH conditions of the wound site. For nonmedically literate patients, the visible color changes provide an intuitive means to monitor wound healing or deterioration. These optoelectronic sensors utilize colored LEDs and photodiodes to measure changes in absorbance and reflection associated with the dressing’s color. A POC strategy for pH determination of wound biofluids using holographic technology has been devised, leading to an adaptive holographic pH-sensing bandage.^243,285^ This dressing operates by detecting subtle shifts in optical peaks, enabling precise monitoring of pH variations. Figure 10G shows the wound dressing integrated with holograms to detect pH changes down to 0.25 intervals across a range from 6.50 to 8.75 in the wound environment.^281^

The use of colorimetric sensors has been expanded to a wider range of biomarkers, as in the case of the wax paper in Figure 10H.^259^ This colorimetric wax paper can detect skin temperature, TMA (trimethylamine), uric acid, moisture, and pH. A breakthrough in polymeric fiber optics for chronic wound diagnosis and management, illustrated in Figure 10I, was achieved with simultaneous measurements of pH, concentration of glucose, and of metalloproteinases (MMPs) in the exudate.^285^ This multibiomarker sensor utilizes a fluorescent sensing approach by coating PDMS fibers with fluorescein isothiocyanate (FITC) and glucose oxidase-functionalized nanoparticles. This configuration enables precise detection of pH (range: 5–7), glucose (range: 0–10 mmol), and MMP concentrations (range: 0–10 μg/mL) in the wound exudate.

Research is expanding to include therapeutic functions, such as drug delivery,^269,270^ functional textiles,^249^ and electrical stimulation^50^ into wearable sensors. By integrating such functionalities, these devices dynamically adapt to the wound environment to expedite healing.^245,286–288^ Wireless closed-loop smart wound care technologies may improve wound monitoring and treatment outcomes. For instance, a battery-free, wireless smart wound dressing was developed, which is capable of monitoring wound infection and delivering drugs on-demand using flexible electronics and NFC.^270^ This system includes sensors for temperature, pH, and uric acid, establishing a closed-loop mechanism that integrates real-time monitoring with therapeutic functions. Its effectiveness was validated through both *in vitro* and *in vivo* studies. Another study introduced a wireless, closed-loop smart bandage incorporating sensors and stimulators.^50^ The bandage features a flexible bioelectronic system with skin-interfacing hydrogel electrodes for monitoring skin impedance and temperature, as well as delivering electrical stimulation in response to wound conditions. This approach led to accelerated wound healing and enhanced dermal recovery in preclinical models. Multifunctional platforms equipped with cloud connectivity and AI-driven analytics further enhance diagnostic precision, offering predictive insights and optimized care.^289^ Another interesting example is the bandages designed for diabetic wounds that adapt by monitoring glucose levels and releasing therapeutic agents as needed.^286,290^ In this case, hydrogels or porous membranes^263,268^ are employed for passive exudate absorption and integrated into wound dressings for continuous biofluid collection. Hydrogel-based dressings further contribute by mimicking the extracellular matrix, fostering a moist healing environment suitable for tissue regeneration.^286,291^ Water-powered bandages have also been fabricated to deliver electrical stimulation at low cost, which is essential for resource-limited settings.^292^ Furthermore, integrated electrical stimulation and photodynamic therapy enhance healing by promoting cell proliferation, angiogenesis, and antimicrobial activity. Electrical stimulation for wound healing has also been done with triboelectric nanogenerator (TENG)-based wearables.^293,294^

Several challenges persist for the use of wearable sensors in wound monitoring. The heterogeneity of the wound biofluid composition across different wound types complicates sensor calibration and sensitivity optimization. Biofluid volume variability, such as excessive exudate in chronic wounds versus minimal fluid in healing wounds, impacts the sensor performance. Environmental factors such as temperature, humidity, and movement can also affect sensor stability, necessitating robust material designs. Furthermore, biocompatibility is a significant concern; prolonged sensor contact must not impede healing or provoke an adverse immune response.

Despite the current challenges, a few wearable technologies for wound healing have reached the market. Tools such as Woundchek Protease Status monitor protease activity in chronic wounds, delivering critical biochemical insights to guide treatment strategies. Devices like Natrox, which deliver oxygen to wounds, improve metabolism and tissue oxygenation, while SNaP (Smart Negative Pressure) provides a portable method for managing exudate and reducing bacterial loads. Grapheal’s smart patch offers continuous, noninvasive monitoring of chronic wounds. This wearable patch uses a graphene core to detect and record bioparameters with high sensitivity, enabling caregivers to track healing progress remotely and receive early warnings of potential infections. Moreover, the patch can stimulate wound healing while conforming flexibly to various wound shapes. These systems are a demonstration of the seamless integration of monitoring and therapeutic functions, allowing real-time care adjustments.

### 3.5 Urine

Urine is an indispensable diagnostic medium due to its unique ability to reflect a spectrum of physiological and pathological conditions.^295,296^ It contains a variety of metabolites, electrolytes, proteins, and other analytes that diffuse from the bloodstream, providing a window into metabolic and systemic health. Biomarkers indicative of conditions, such as diabetes, kidney disorders, urinary tract infections, and metabolic imbalances, can be detected. However, the intrinsic dilution of analytes during urine production means that only those with sufficiently high plasma concentrations are readily detectable, unless supported by advanced, highly sensitive detection methods. The state of the art in wearable urine diagnostics integrates real-time, noninvasive analysis with user-friendly technologies.^297,298^ Biosensors embedded in diapers (see Section 5.8), for instance, enable detection of key biomarkers like glucose, bilirubin, and proteins. Using diapers is particularly beneficial for neonates, the elderly, and individuals requiring long-term care, where traditional sampling methods are impractical. Many of these devices now incorporate self-powered technologies, such as biofuel cells that generate electricity from urine itself.^297^ In addition to single-use wearable devices, smart urine analysis systems are evolving into sophisticated, multifunctional platforms, often integrating microfluidic channels for precise biomarker quantification alongside wireless communication modules for data transmission.

AI-based systems can analyze trends in urinary biomarkers, predict potential health risks, and provide tailored health insights. Examples are smart toilets equipped with sensors and cameras that monitor urine flow, color, and volume while analyzing multiple biomarkers *in situ*.^295,296,299–301^ These devices can also collect stool samples and integrate their analysis with smartphone apps, offering a comprehensive approach to health tracking. Commercial diagnostic tools have expanded to include fluorescence-based sensors and immunochromatographic assays that address longstanding issues like interpretation variability and sensitivity. For example, optical readers integrated with immunoassay platforms have improved the reliability in detecting acute kidney injury biomarkers.^302^ Systems such as nanoparticle-based CRISPR-Cas-amplified platforms^303^ or biosensors with volatile organic compound-based detection^304^ provide enhanced sensitivity, permitting the early detection of critical conditions like sepsis and cancer.^295^ Wearable devices with hybrid capabilities are also gaining traction; these systems not only monitor biomarkers but also deliver feedback to adjust therapeutic interventions in real time.^295^

The episodic nature of urination limits the temporal resolution of data collection, often restricting wearable devices to single-use designs. Variability in urine composition due to hydration, dietary intake, and circadian rhythms complicates the standardization of diagnostic thresholds.^305–307^ Additionally, embedding sensors into flexible and durable wearables requires overcoming significant engineering hurdles, including ensuring biocompatibility and user comfort.

### 3.6 Breath

Diseases of the respiratory system represent a major global health challenge, with increasing incidence rates of COPD, asthma, lung cancer, and other conditions placing significant burdens on healthcare systems.^308,309^ Exhaled breath analysis permits monitoring health systemically, including metabolic disorders, daily metabolic fluctuations, and overall physiological well-being. For instance, volatile organic compounds (VOCs) in exhaled breath, such as acetone and ammonia, provide insights into lipid metabolism, renal function, and liver health, making breath analysis useful for managing diabetes, kidney dysfunction, and cirrhosis.^310–313^ Traditional breath monitoring methods, such as pulmonary function tests and imaging studies, offer crucial diagnostic information, but they often require complex equipment and professional operation. In contrast, health monitoring can be done using exhaled breath aerosols (EBA) and exhaled breath condensate (EBC), which contain abundant biomarkers for both respiratory and systemic health conditions.^314,315^ Laboratory analysis of EBA involves particle collection and subsequent biochemical analysis. Particle collection typically utilizes filter membranes or electrostatic deposition techniques, followed by solvent extraction for mass spectrometry analysis or PCR-based determinations. For instance, an early COVID-19 diagnosis can be achieved by detecting SARS-CoV-2 nucleic acids in EBA. These laboratory analytical techniques provide high-sensitivity and high-resolution detection methods, permitting data validation and calibration of wearable devices.^4,316^

EBA analysis has evolved from laboratory settings to real-time portable monitoring. Smart masks (see Section 5.7) serve as ideal platforms for wearable EBA analysis, incorporating features such as particle collection through integrated filter membranes or electrostatic deposition devices for real-time sample analysis via microfluidic technology. These devices can integrate immunosensors^317^ or CRISPR sensing technology^33^ for real-time pathogen detection, such as SARS-CoV-2 protein detection through immunoelectrochemical sensors, enabling rapid COVID-19 screening within minutes (Figure 11A).^318^ Alternatively, a mask can incorporate a series of microfluidic chambers designed to sequentially extract, amplify, and detect viral DNA using CRISPR-based technology, providing an efficient approach for viral diagnostics (Figure 11B).^33^ Moreover, wearable devices can monitor respiratory infection levels and treatment efficacy^319,320^ by measuring inflammation biomarkers in EBA (Figure 11C).

**Figure 11:**
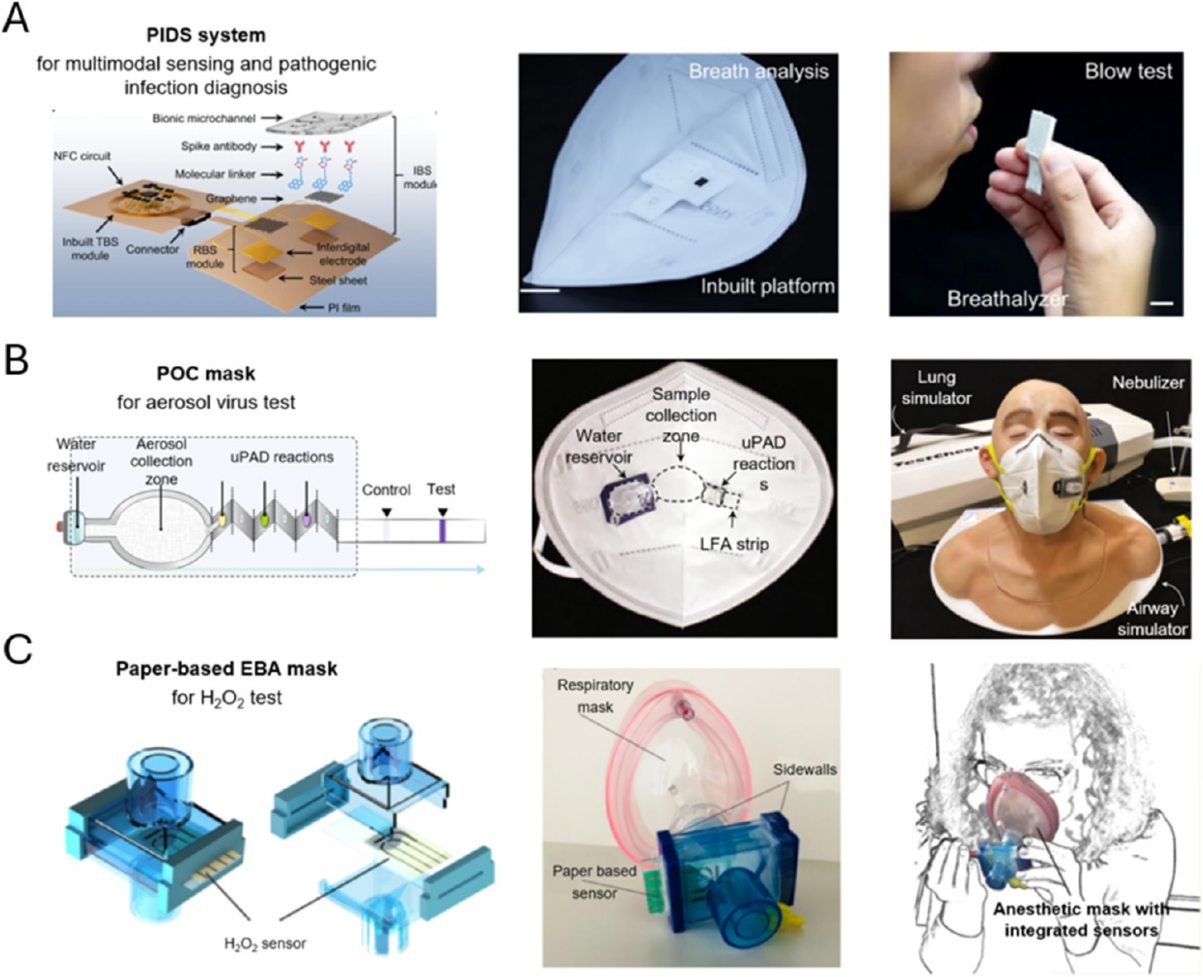
Wearable smart mask system development for in situ breath aerosol and condensate analysis. (A) A wireless, battery-free, multifunctional pathogenic infection diagnosis system. Reproduced from Li et al.^318^ Available under a CC BY 4.0 license. © 2023 Springer Nature. (B) A POC mask with microfluidics for COVID-19 test-based CRISPR technology. Reproduced with permission from Nguyen et al.^33^ © 2021 Springer Nature. (C) A paper-based electrochemical sensor in a mask for H_2_O_2_ detection in exhaled breath aerosol. Reproduced from Maier et al.^319^ Available under a CC-BY 4.0 license. © 2019 American Chemical Society.

EBC is normally analyzed using LC-MS, nuclear magnetic resonance (NMR), and immunological detection methods (e.g., ELISA). Wearable devices for EBC analysis typically utilize passive cooling technologies, such as hydrogel evaporation and radiative cooling, to achieve efficient condensation under low power consumption (Figure 11C).^32^ These devices can integrate electrochemical, optical, and immunosensors for real-time detection of biomarkers, such as hydrogen peroxide, nitrite/nitrate, and ammonium. The collected data can be transmitted to smartphones or cloud platforms for real-time analysis and remote monitoring, with machine-learning algorithms enhancing analytical accuracy and efficiency. Besides particle collection, there are other innovations based on materials able to sample and analyze targets in breath, vapor, and/or gas.

#### 3.6.1 Gas-Sampling and Sensing Mechanisms

We have already mentioned that exhaled gases contain biomarkers with clinical significance, including nitrogen oxides (NO_2_, NO), hydrogen sulfide (H_2_S), ammonia (NH_3_), and VOCs like ethanol, acetone, and alkanes.^32,321^ These biomarkers are often related to specific diseases, including lung cancer, asthma, COPD, and tuberculosis.^32^ Current clinical breath analyzers are typically large and unable to provide continuous, real-time monitoring, which has led to growing interest in miniaturized, wearable “e-noses”. Electrochemical gas sensors that operate at room temperature are more suitable, with recent advances focusing on replacing the liquid electrolyte with flexible hydrogels. In the next few subsections, we discuss different hydrogel-based strategies to detect NO_2_, H_2_S, and O_2_.

Figure 12A illustrates that hydrogels, as ion-conductive electrolytes, can be utilized to form a symmetrical electrode-hydrogel-electrode structure in electrochemical gas sensors when combined with metal electrodes. Under a direct current voltage, an anode-hydrogel interface and a cathode-hydrogel interface are established. Oxidizing gases such as NO_2_ and O_2_ are reduced at the cathode under the influence of the electric field, and the electrons transferred between the gas and the electrode generate a current response.^322–327^ Another transducing principle depicted in Figure 12B does not rely on constructing a special electrode-hydrogel interface, but requires a small hydrogel volume and low initial conductivity, as well as strong adsorption and solubility of specific gases, such as NH_3_.^328,329^ The hydrolyzed gas produces ions, thereby reducing the sensor’s impedance. Depending on the electrodes’ potential difference and on the interaction between the gas and the interface, self-powered electrochemical cell-type sensors can also be built. The reactive metals on one side of the hydrogel act as anodes, while the target gas (e.g., NO_2_) spontaneously reduces at the cathode to generate current, as shown in Figure 12C.^330^ Another type of self-powered potentiometric gas sensor relies on the adsorption of gas onto electrodes. For example, H_2_S can be adsorbed onto the surface of an Ag electrode, altering its potential and enabling self-powered gas detection with minimized power consumption (Figure 12D).^331^

**Figure 12:**
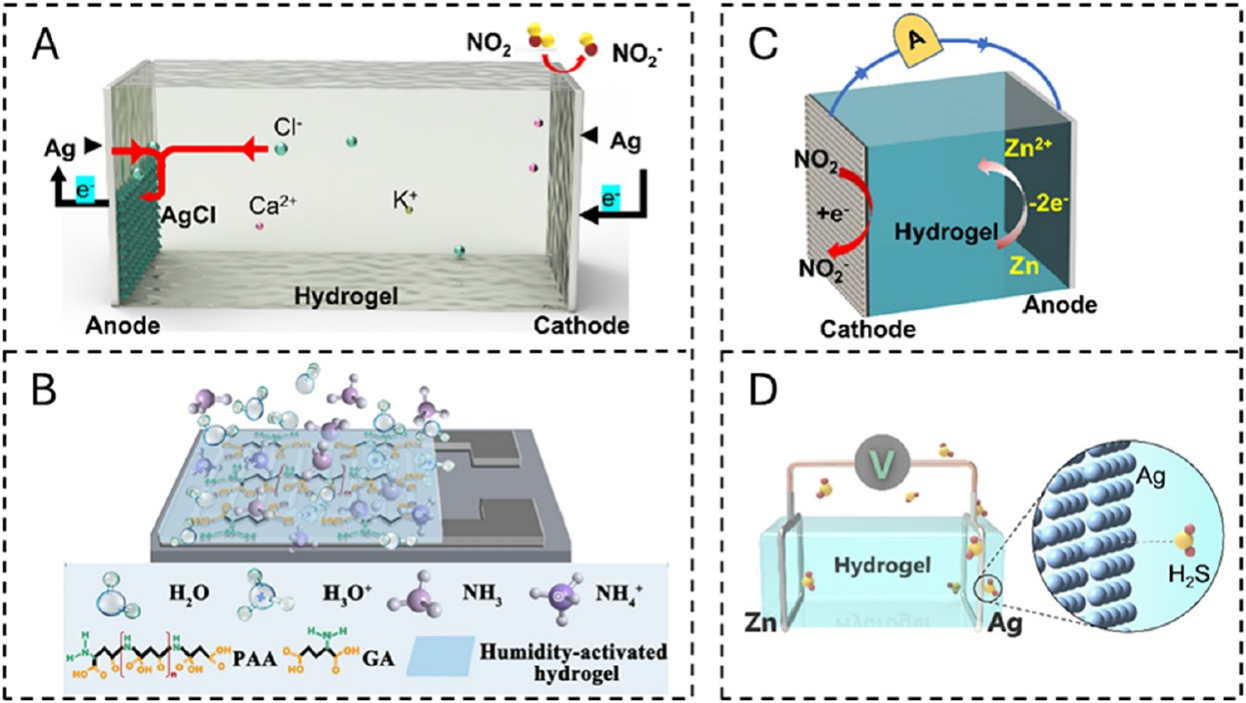
Gas-sensing mechanisms of hydrogel-based gas sensors. (A) Schematic illustrating that the NO_2_ gas is reduced at the cathode-hydrogel interface of the electrochemical-type gas sensor under a DC external electric field. Reproduced with permission from Wu et al.^322^ © 2021 John Wiley & Sons. (B) Schematic showing that soluble gas (NH_3_) is absorbed by hydrogel and then hydrolyzed to generate movable ions, thereby reducing the resistance of hydrogel electrolyte. Reproduced with permission from Liu et al.^328^ © 2021 Elsevier. (C) Schematic of the self-powered electrochemical cell-type NO_2_ sensor with two different electrodes. Reproduced with permission from Wu et al.^330^ © 2023 John Wiley & Sons. (D) Schematic diagram illustrating the device structure and H_2_S-sensing mechanism of self-powered potentiometric gas sensor. Reproduced from Huang et al.^331^ Available under a CC-BY 4.0 license. © 2023 Springer Nature.

#### 3.6.2 Detection of NO_2_

NO_*x*_ is an important biomarker for lung infections and bowel diseases.^332^ The increasing recreational use of NO_2_ also makes the development of rapid and simple techniques for its determination in breath relevant.^333^ Wu and co-workers found that ion-conductive polymer hydrogels could respond to NO_2_.^325^ A CaCl_2_-polyacrylamide (PAM) hydrogel enwound by two silver coils^322^ showed high sensitivity (119.9%/ppm) at room temperature, in addition to high selectivity, short response and recovery times (29.8 and 41.0 s, respectively), good linearity, and a low LOD of 80 ppb. Because the electrical response mainly comes from the gas reaction at the hydrogel-electrode interface, mechanical deformation of the hydrogel, such as stretching and bending, has little impact on its sensing performance. In contrast, the electrolyte composition of the hydrogel affected sensor performance, and the addition of specific ionic salts to increase conductivity can promote electrochemical reactions at the electrode-hydrogel interface, thereby enhancing sensitivity. The high conductivity also suppresses electrical signal interference caused by ionization of dissolved gases due to hydrolysis, enhancing the selectivity. Increasing the number of silver wire coils was found to improve the sensor’s response due to the larger reaction area (Figure 13Ai-ii).

**Figure 13:**
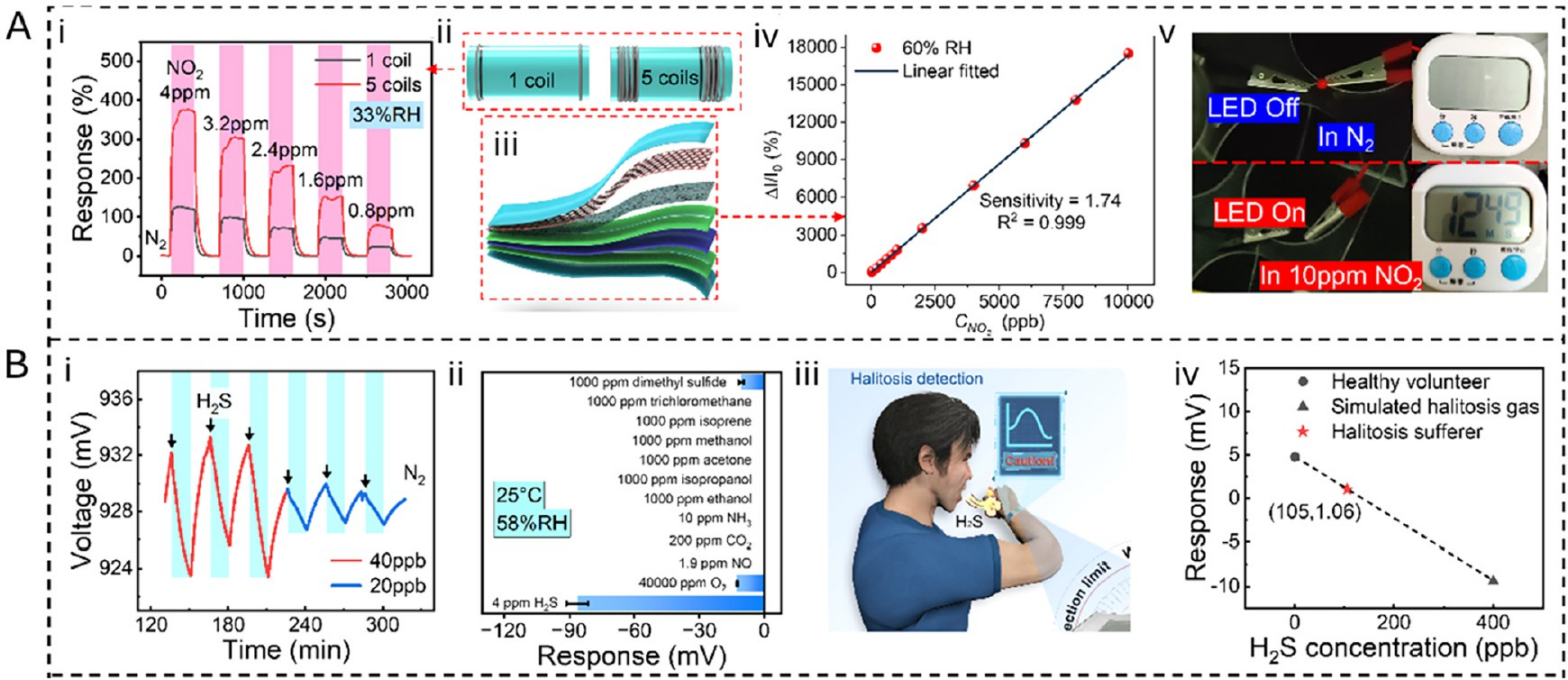
Room-temperature gas sensing performance. (A) NO_2_ sensor. (i, ii) Dynamic responses of the CaCl_2_-infiltrated hydrogel sensor with one and five turns of Ag coil electrode to 0.8–4 ppm of NO_2_ gas at 33% RH. Reproduced with permission from Wu et al.^322^ © 2021 John Wiley & Sons. (iii) Structure of the self-powered NO_2_ sensor. (iv) Sensor response versus NO_2_ concentration (C_NO2_) in a wide concentration range (200–10,000 ppb), showing its excellent linearity and high sensitivity. (v) Photographs presenting that the self-powered sensor in 10 ppm of NO_2_ can generate sufficient current to turn on the LED and electronic timer. Reproduced with permission from Wu et al.^330^ © 2023 John Wiley & Sons. (B) H_2_S sensor. (i) Dynamic open-circuit voltage change of Zn/Ag/hydrogel sensor to 40 and 20 ppb H_2_S. (ii) Comparison of the responses of the Zn/Ag/hydrogel H_2_S sensors to various gaseous chemicals, reflecting the high selectivity. (iii, iv) Response versus H_2_S concentration curve of the Zn/Ag/hydrogel sensor to the exhaled breath of a healthy volunteer/halitosis sufferer and simulated halitosis gas. Reproduced from Huang et al.^331^ Available under a CC-BY 4.0 license. © 2023 Springer Nature.

To address long-term energy consumption, electrode corrosion, and humidity interference issues, an electrochemical self-powered sensor was designed, which featured heterogeneous anode/cathode materials, a recoverable electrode, and a hydrophobic hydrogel-electrode interface.^11^ Compared with the silver coil structure mentioned above, the self-powered sensor configured as a flat-stacked Zn-hydrogel-carbon structure significantly increased the gas reaction surface area (Figure 13Aiii). The oxidized Zn electrode can be recovered through the charging process (reduced by applying a reversed DC voltage), ensuring long-term stability and a prolonged lifespan. With the addition of amphiphilic Zn­(OTf)_2_ to the hydrogel electrolyte, the hydrophobic electrode-gel interface created reduced humidity interference by repelling the adsorption of environmental moisture. With these optimizations, the self-powered sensor exhibited outstanding performance, including ultrahigh sensitivity (1.92%/ppb), linearity (R^2^ = 0.999), an ultralow theoretical LOD (0.1 ppb), and humidity immunity (Figure 13Aiv). Notably, the energy generated from a 10 ppm of NO_2_ reaction could even turn on an LED and electronic timer (Figure 13Av).

#### 3.6.3 H_2_S Sensors

H_2_S is a typical biomarker produced by the metabolism of organic matter by accumulated bacteria, which can indicate oral diseases such as periodontal and mucosal diseases. A clinical study revealed that the average H_2_S concentration exhaled from patients with oral halitosis is 20.64 ppb, much higher than the value of healthy volunteers (3.36 ppb). Wu and co-workers reported a self-driven and stretchable potentiometric H_2_S sensor consisting of two different metal electrodes and a PAM/calcium alginate (CA) double-network hydrogel electrolyte.^331^ Because adsorption and chemical reaction between H_2_S and different metal electrodes differ, the open-circuit voltage (OCV) would change during exposure to different concentrations of H_2_S. The chemical adsorption of H_2_S on Ag electrodes was the strongest, with the open-circuit voltage being reversible when H_2_S was removed. This gas sensor exhibited high sensitivity, repeatability, and selectivity at room temperature (25 °C), being suitable for working under severe mechanical deformations or in aerobic/anaerobic environments. Furthermore, an organo-hydrogel obtained by replacing pure water with a water-glycerol binary solvent had an increased performance in terms of stability and environmental tolerance. Figure 13Bi shows that the self-driven sensor could detect ultralow concentrations of H_2_S (20 ppb in the experiment and 0.79 ppb in theory), which meets the demand of detecting H_2_S biomarkers released by bacteria. The sensor also had good selectivity, as indicated in Figure 13Bii, and could be used to diagnose halitosis (Figure 13Biii-iv).

#### 3.6.4 O_2_ Sensors

Detection of O_2_ is important for health monitoring because the respiratory oxygen content or transcutaneous oxygen partial pressure (tcPO_2_) is an indicator of cellular metabolism. Wearable O_2_ sensors can be used in noninvasive diagnosis and rehabilitation of some chronic diseases, including respiratory diseases, diabetic foot, wound healing, and infection. Wearable electrochemical sensors have been developed by incorporating a hydrogel and a soft elastomer. For example, Wu and co-workers designed a waterproof and breathable Ecoflex-encapsulated hydrogel film, which can detect O_2_ within a wide range of concentrations (from 5 ppm to 90% O_2_).^324^ Notably, the waterproof and breathable elastomer endows good sweat and moisture interference. Therefore, not only could the sensor be assembled in a mask for breath detection, but it could also be adhered to the skin for monitoring tcPO_2_.

Remaining challenges in wearable EBA and EBC analysis involve the standardization of sampling and analytical methods to ensure comparability and reliability. Sensors must achieve higher sensitivity and selectivity for accurate detection of low-concentration biomarkers, probably employing nanomaterials and novel sensing technologies. ML and big data methodologies may solve the problem of managing and analyzing the vast, complex data sets generated by these devices.^4,334^

## 4 Implantable and Ingestible Biosensors

When monitoring is required for extended periods or when the desired biofluids are not accessible from the outside of the body, implantable and ingestible biosensors can be used. These biosensors can be applied for the detection of metabolites from different body fluids, such as the cerebrospinal fluid (CSF), gastric fluid, interstitial fluid, or blood, in real time.^335^ In the following, each biofluid will be discussed in further detail regarding its nature and bioanalytical application strategies.

### 4.1 Cerebrospinal Fluid

CSF is a clear liquid primarily produced in the choroid plexus of the ventricular system, playing a key role in cerebral blood regulation and in transporting molecules such as neurotransmitters and toxins.^336^ It flows constantly through the brain and spinal cord, providing mechanical support and acting as a shock absorber.^337^ In addition to monitoring physical parameters such as dielectric properties^338^ or flow dynamics,^339^ a primary area of advancement lies in the *in situ* quantification of specific chemical and biochemical species within CSF. Detecting biomarkers in CSF is important for diagnosing neurodegenerative diseases, cancers, and immune disorders.^340–343^ The collection of CSF has historically been done by performing either a needle biopsy or lumbar puncture (LP), which is extremely invasive and painful. In addition, LP leads to multiple health-related complications for patients, including long-lasting headaches after the procedure.^344^ Furthermore, successive sampling can influence the concentration of some biomarkers, hampering the reliability of the sample.^345^ These reasons, combined with the possibility of real-time monitoring, are some of the most relevant for promoting the development of implantable devices for the analysis of CSF.

The practical application of implantable sensors is intricately linked to the anatomy of the central nervous system and the inherent challenges of accessing CSF-containing spaces. CSF resides primarily within the brain’s ventricles, the subarachnoid space around the brain and spinal cord, and the central channel of the spinal cord.^346,347^ Accessing these locations typically requires neurosurgical procedures. Ventricular access often involves drilling a burr hole in the skull and inserting a catheter, while spinal access may involve procedures similar to lumbar puncture but utilizing a device intended for implantation.^348,349^ Each access route presents significant risks, including infection (meningitis, ventriculitis), hemorrhage, and potential damage to neural tissue. Therefore, sensor design must prioritize minimizing invasiveness. This translates into demands for small device size, flexibility to conform to surrounding anatomical structures, and the use of functional materials with established long-term biocompatibility to minimize tissue reaction and inflammation. Furthermore, the mechanical properties of the implant must be robust to withstand physiological movements and CSF pressure fluctuations without fracturing or becoming dislodged.

Electrochemical methods are well-suited for such sensing devices due to their inherent potential for miniaturization, high sensitivity, selectivity, low power consumption, and suitability for digital integration. However, translating these methods into reliable *in vivo* sensors requires significant innovations in materials science and device engineering. Reliable molecular modification of electrode surfaces is essential for developing stable electrochemical sensors. Traditional gold–thiol (Au–S) linkages,^350,351^ such as those formed with cysteine or mercaptopropionic acid, are prone to displacement by endogenous thiols like glutathione (GSH), leading to signal drift or loss.^352^ Stability can be enhanced by employing bidentate thiol linkers, which form dual coordination bonds with gold surfaces.^353^ This configuration demonstrated significantly lowered signal loss (5.3% after 2 h in GSH solution, compared to a 23.4% decrease observed with monodentate Au–S linkages). Further advancements include the use of gold–alkyne (Au–C≡C) bonds. Sensors utilizing Au–C≡C linkages exhibited superior stability, maintaining signal integrity even after 4 h in GSH solution.^354^ This improved performance is attributed to the higher bond energy and lower Gibbs free energy associated with Au–C≡C bonds, resulting in more robust surface assemblies. Additionally, conductive polymer coatings, such as poly­(3,4-ethylenedioxythiophene) (PEDOT) integrated with polydopamine melanin, have been explored to improve electrode stability and biocompatibility.^355^ These coatings not only enhanced electrochemical performance and mechanical stability but also supported cell proliferation and differentiation, making them suitable for long-term implantable applications.

A sonogel-carbon (SNGC) electrode modified with l-leucine was used for the electrochemical detection of homovanillic acid (HVA) in CSF.^356^ HVA, a key metabolite of dopamine and norepinephrine, serves as a biomarker for conditions like neuroblastoma.^357^ The fabrication of this SNGC-leucine sensor utilized a sol–gel process with methyltrimethoxysilane (MTMOS) and hydrochloric acid, followed by the incorporation of graphite powder and l-leucine. The resulting composite was packed into glass capillary tubes and polished to create the working electrode. The presence of l-leucine on the electrode surface enhanced the electrochemical detection of HVA, with the optimal voltammetric response being pH-dependent. DPV analysis demonstrated high analytical sensitivity, achieving a low LOD of 0.4 μM for HVA within a linear concentration range of 0.5 to 50 μM. The sensor’s analytical performance was evaluated in artificial CSF samples.

Another example targeting neurotransmitter monitoring employed a miniaturized, wireless, and battery-free implantable platform that integrates electrochemical sensing (Figure 14).^358^ This device had carbon nanotube (CNT)-based sensors, taking advantage of its exceptional electrical conductivity and high surface area to detect neurotransmitters such as dopamine. A μ-LED was integrated on the same probe, enabling precise optogenetic control of nearby neuronal activity and allowing real-time investigation of *stimulus*-response dynamics. Wireless power transfer technology, combined with the absence of a battery, resulted in a compact (12 mm × 8.5 mm × 3.2 mm) and lightweight (<49 mg) device that minimized invasiveness and eliminated the need for battery replacement. A flexible serpentine interconnect ensured mechanical stability and electrical connectivity during implantation in moving subjects, including small animal models such as mice. Both *in vitro* and *in vivo* experiments demonstrated the platform’s sensitivity and selectivity, highlighting its ability to monitor dopamine concentration changes in response to optogenetic stimulation and to track real-time dopamine levels following drug exposure (e.g., opioids and naloxone).

**Figure 14:**
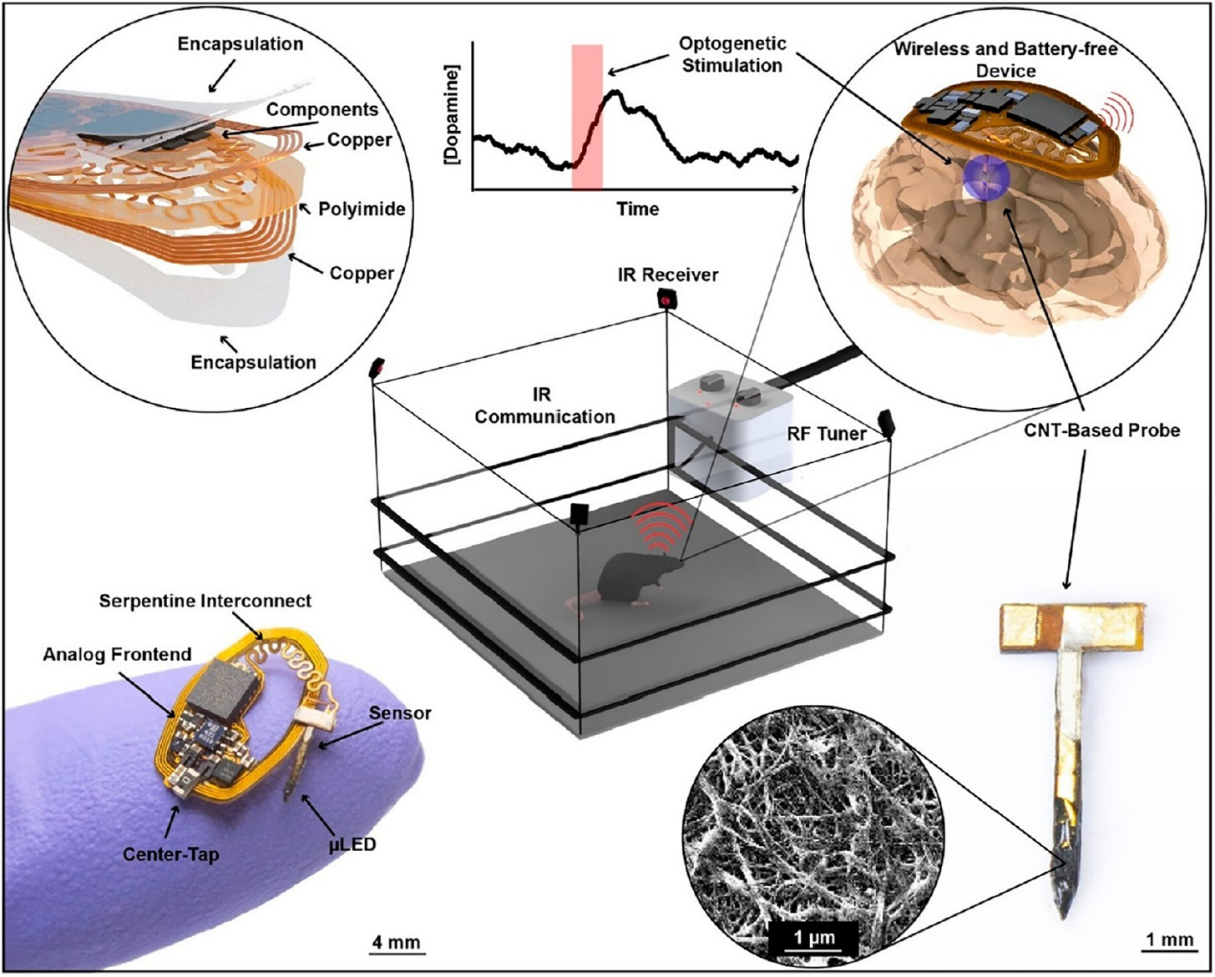
Overview of the implantable electrochemical device for the wireless and battery-free monitoring of catecholamines in CSF developed by Stuart et al. The image shows the device and its layers, as well as its operating principles and typical signals. Reproduced from Stuart et al.^358^ © 2022 American Chemical Society.

In addition to organic molecules, the monitoring of inorganic ions like calcium (Ca^2+^) in CSF permits understanding neuronal signaling and pathological states.^359,360^ An all-solid-state potentiometric microelectrode, fabricated on the tip of an acupuncture needle (diameter <80 μm), was developed for *in vivo* Ca^2+^ monitoring in rat CSF.^361^ This design, incorporating functional materials, circumvents the drawbacks associated with conventional liquid-filled reference electrodes. The calcium ion-selective electrode (ISE) was constructed by coating the acupuncture needle tip premodified with a PEDOT/poly­(styrenesulfonate) (PSS) contact with a calcium ion-selective membrane. The resulting Ca^2+^-ISE exhibited a Nernstian response over a wide concentration range (μM to mM levels) with a near-ideal slope (around 31 mV/decade) and a low LOD (0.12 μM Ca^2+^). *In vivo* application of this microelectrode in rats following spinal cord transection revealed a sharp and significant increase in CSF calcium concentration from a baseline of approximately 21 to 133 μM, highlighting its capability for real-time monitoring of dynamic ionic changes in the CNS with high temporal resolution. The versatility of this platform allows for the potential measurement of other ions by simply changing the ion-selective membrane, permitting investigation of various physiological and pathological conditions.

The detection of low-abundance protein biomarkers in CSF requires highly sensitive and specific recognition strategies. A sensor utilizing electrografted laser-induced graphene (E-LIG) electrodes was developed for the label-free detection of the poly­(GP) dipeptide repeat, a biomarker for *C9orf72*-associated amyotrophic lateral sclerosis (ALS), directly in unprocessed CSF.^362^ ALS is often linked to a hexanucleotide repeat expansion in the *C9orf72* gene, leading to the production of toxic dipeptide repeat proteins, including poly­(GP). Early detection of these biomarkers may allow for a timely intervention. The fabrication of the E-LIG electrodes involved a cost-effective laser-based conversion of a PI substrate to porous, conductive graphene. The graphene surface was then functionalized via electrografting and coated with antipoly­(GP) antibodies. Label-free detection of poly­(GP) was achieved using electrochemical impedance spectroscopy (EIS), where the change in the charge transfer resistance (R_ct_) upon antigen binding served as the detection signal. The E-LIG immunosensor demonstrated high sensitivity (LOD of 0.19 ng/mL in buffer and 0.27 ng/mL in CSF) and selectivity for poly­(GP). The sensor quantified poly­(GP) in CSF samples from ALS patients and distinguished between *C9orf72*-associated and non-*C9orf72*-associated ALS, thus confirming its potential for clinical diagnostics and POC applications.

In another approach for protein biomarker detection, a label-free electrochemical immunosensor for glial fibrillary acidic protein (GFAP),^363^ a biomarker for neurological conditions, was developed by using SPCEs modified with a nanocomposite.^364^ This sensor was applied to the GFAP determination in human CSF. The nanocomposite leverages the high conductivity of reduced graphene oxide (rGO), the peroxidase-like activity of molybdenum disulfide (MoS_2_), and the electrocatalytic effect of silver nanoparticles (AgNPs) to enhance the amperometric detection of hydrogen peroxide, indirectly quantifying the antigen–antibody binding. The immunosensor was fabricated by covalently immobilizing a specific capture antibody for GFAP onto a nanocomposite-modified SPCE. The biorecognition event was detected by measuring the electrocatalytic reduction current of hydrogen peroxide. The immunosensor exhibited a linear response to GFAP concentrations from 0.6 to 100 ng/mL with a low LOD of 0.16 ng/mL. Its practical utility was confirmed by successfully determining the endogenous GFAP levels in CSF samples from patients diagnosed with encephalitis.

For Parkinson’s disease diagnostics, a screen-printed electrochemical sensor modified with palladium nanoparticles (PdNPs) was developed for the detection of epinephrine and α-synuclein, two key biomarkers.^365^ A conductive ink based on carbon black in a poly­(vinyl alcohol) (PVA) matrix was used to fabricate the three-electrode system. The deposition of PdNPs onto the electrode surface enhanced both electrical conductivity and reaction kinetics, with deposition parameters optimized using chemometric methods. The resulting sensor demonstrated a response to epinephrine in the range of 0.75 to 100 μM, with a LOD of 0.051 μM, showing promising results in synthetic CSF. Additionally, these disposable SPCEs were modified with specific antibodies for the EIS-based detection of α-synuclein. In phosphate buffer, this immunosensor exhibited a linear response to α-synuclein concentrations from 1.5 to 15 μg mL^–1^, with an LOD of 0.13 μg mL^–1^.

An implantable sensor for measuring CSF dielectric properties as a potential biomarker for Alzheimer’s disease contained a voltage-controlled oscillator and an implantable antenna designed for wideband operation with a low-quality factor (Q-factor) to maintain stability despite variations in the CSF.^366^ The implantable antenna transmitted signals that interact with CSF, and the received power level was measured externally. With this sensor, an increase in the loss tangent was correlated with the progression of Alzheimer’s disease. Zarrin et al. developed an implantable sensor for monitoring CSF flow in patients with ventriculoperitoneal shunts.^367^ These shunts can divert CSF in conditions like hydrocephalus, but they often face high failure rates, and current clinical practices lack effective solutions for continuous, long-term monitoring of CSF flow rates. The sensor consisted of electrodes arranged in a cylindrical configuration surrounding the flow path. These electrodes generated an electric field across the CSF, inducing spatial variations in charge density within the CSF as it flowed through the shunts. The variations correlated with the flow rate, allowing the sensor to generate an electrical signal that corresponded to fluid movement. The recorded signals were then converted into readable flow data, enabling real-time monitoring of CSF dynamics. The ability to monitor CSF flow with high sensitivity was demonstrated, with an average error of 4.2% and only consuming 37.5 microjoules per flow measurement.^367^

A challenge hindering the long-term performance of implantable CSF sensors is biofouling, the nonspecific adsorption of biomolecules (primarily proteins like albumin, even at CSF’s relatively low concentrations) followed by cellular adhesion (including microglia and astrocytes) onto the sensor.^368,369^ This process initiates a foreign body response, potentially leading to glial scar formation, which physically obstructs analyte diffusion to the sensing interface, consequently degrading signal sensitivity, accuracy, and overall device lifespan. To mitigate this, functional materials and surface engineering strategies are crucial. Passive approaches involve modifying the sensor interface with antifouling coatings, such as hydrophilic polymers like polyethylene glycol (PEG)^370^ or highly effective zwitterionic materials^371^ (e.g., poly­(sulfobetaine methacrylate)),^372^ which creates a hydration layer to repel protein adsorption. Another avenue utilizes nanomaterials and fabrication techniques to create specific micro/nanotopographies on the sensor surface, bioinspired by structures like shark skin,^373^ to physically deter cellular attachment. Furthermore, active antifouling methods are being explored with electrical potentials applied to repel charged biomolecules (electrophoresis/dielectrophoresis).^374,375^ Achieving robust, long-term bioinertness in the complex CSF environment likely requires combinatorial approaches integrating these materials and surface modification techniques.

In addition to the challenges related to biointeractions, another hurdle lies in the disparity in mechanical properties between conventional rigid sensor materials (often with Young’s moduli in the GPa range) and the extremely soft neural tissue surrounding CSF pathways (typically <1 kPa).^376,377^ This mechanical mismatch can cause chronic inflammation, tissue damage due to micromotion between the device and tissue, and impede seamless integration. Consequently, developing flexible and stretchable sensors using functional materials is essential for long-term *in vivo* stability and biocompatibility within the CNS. Materials such as elastomeric polymers (e.g., PDMS),^378,379^ flexible polyimides,^380^ or parylene coatings serve as substrates or encapsulation layers.^381,382^ Integrating nanomaterials like carbon nanotubes,^383^ graphene,^384^ or metallic nanowires^385^ into these polymers can create conductive composites for electrodes and interconnects that retain functionality under strain. Device architectures incorporating thin-film deposition techniques and stretchable designs, such as serpentine interconnects, allow the sensor to conform intimately to the brain or spinal cord contours, minimizing stress concentration.^386,387^ However, fabricating complex electrochemical or optical sensing elements onto these nontraditional platforms while ensuring robust electrical connections and long-term hermetic sealing against the corrosive CSF environment remains a significant engineering challenge requiring interdisciplinary innovations in materials science, microfabrication, and analytical device design.

Another challenge is providing a sustainable power source for these sensors. Batteries have limited lifespans, which require replacement surgeries. While wireless power transfer is promising, its efficiency can decrease with the implant depth, often necessitating continuous external power sources. Biofuel cells (BFCs) present an alternative by harvesting energy directly from the biological environment (see Section 10.3).^388^ These devices utilize biological catalysts (enzymes or microbes) and endogenous fuels present in body fluids, such as lactate found in CSF.^389^ A typical enzymatic BFC consists of an anode and a cathode within a miniaturized housing, where the potential difference drives electrical current.^390^ Implantable BFCs may enable self-powered, autonomous sensors operating continuously for extended periods. However, achieving sufficient power density to operate the sensor and associated wireless communication circuitry remains a bottleneck. Current research focuses on developing more stable enzymes, improving electron transfer efficiency, designing BFC structures that optimize fuel/oxidant access while minimizing fouling, and enhancing overall biocompatibility. Hybrid systems combining BFCs with small rechargeable batteries or capacitors could also help to buffer power fluctuations and ensure reliable operation. Overcoming these challenges is crucial for realizing truly autonomous, long-term implantable diagnostic, and monitoring systems for neurological disorders.

### 4.2 Gastric Fluid

Gastric fluid and gastrointestinal (GI) microbiota are essential for maintaining human health, with deviations from the regular levels of homeostasis being associated with multiple diseases. The gut microbiota is formed by trillions of microbial cells that produce harmful and beneficial metabolites contributing to the development of diseases or providing protection from them.^391,392^ Beneficial metabolites, such as short-chain fatty acids (SCFAs) and γ-aminobutyric acid (GABA), aid digestion and gut-brain communication.^393,394^ On the other hand, metabolites such as p-cresol-sulfate, indoxyl sulfate, deoxycholic acid, lithocholic acid, tyramine, trimethylamine-N-oxide, trimethylaminuria, and *N*-phenylacetylglutamine can be associated with several diseases (e.g., neurodegenerative and cardiovascular diseases).^395,396^ Therefore, close and constant monitoring of the gut microbiota (GM) and their metabolites can provide important health information and indicate possible predispositions to different disorders.^397^

The gastric fluid represents a uniquely hostile milieu for sensor development. Its extreme acidity (typically pH 1–3), driven by hydrochloric acid secreted by parietal cells, coupled with the presence of potent proteolytic enzymes like pepsin, creates a highly corrosive chemical environment.^398,399^ Furthermore, the constant mechanical agitation from peristalsis imposes significant physical stress.^400^ Consequently, designing sensors—whether implantable for applications like postoperative monitoring or ingestible for diagnostic screening—capable of withstanding these conditions while delivering accurate, reliable measurements constitutes a major materials science and engineering challenge. To be effective, these sensors must ideally function reliably while either free-floating or anchored or adhered near the gastric mucosa—the tissue lining the stomach that is the source of key chemical constituents. This necessitates robust materials, stable sensing mechanisms, sophisticated encapsulation strategies, and intelligent device designs.

Current methods to detect and measure metabolites in the gastric fluid include, for example, high-performance liquid chromatography and gas chromatography, which are not suitable for real-time analysis.^401,402^ Electrochemical biosensors used for gastrointestinal fluids include those made with food-grade materials, for example, olive oil and activated charcoal, which can serve as a binder and a conductor, respectively.^403^ In the latter work, glucose was monitored within the GI tract using a biosensor containing the enzyme GOx within the olive oil matrix and activated charcoal (Figure 15). The biosensors had a stable performance in strongly acidic conditions (pH 1.5) over 90 min, with a linear response to glucose concentration from 2 to 10 mM with precise control over sensor activation.^403^ Another ingestible biosensor made with a probiotic *Escherichia coli* (*E. coli*) was used to characterize gut metabolites related to major depressive disorder.^26^ Upon exposure of gut metabolites associated with depression, such as indole, butyrate, tetrahydrofolate, hydrogen peroxide, and tetrathionate, the biosensor activates a CRISPR-DNA writing system, which records the detection event at the molecular level. Genetic recording allows for the analysis of metabolite exposure by sequencing bacterial DNA after it has been excreted. The biosensor was administered orally in capsule form, ensuring safe delivery of the engineered bacteria to the gastrointestinal tract. To achieve spatial specificity, the system utilized riboflavin (vitamin B2) as a marker as its concentration increases in the large intestine. The *E. coli* biosensor was equipped with a riboflavin-responsive element that activates the detection mechanism specifically in the large intestine, thereby providing both temporal and spatial control over metabolite monitoring. With this biosensor, metabolite concentrations could be determined in a time- and dose-dependent manner.^26^

**Figure 15:**
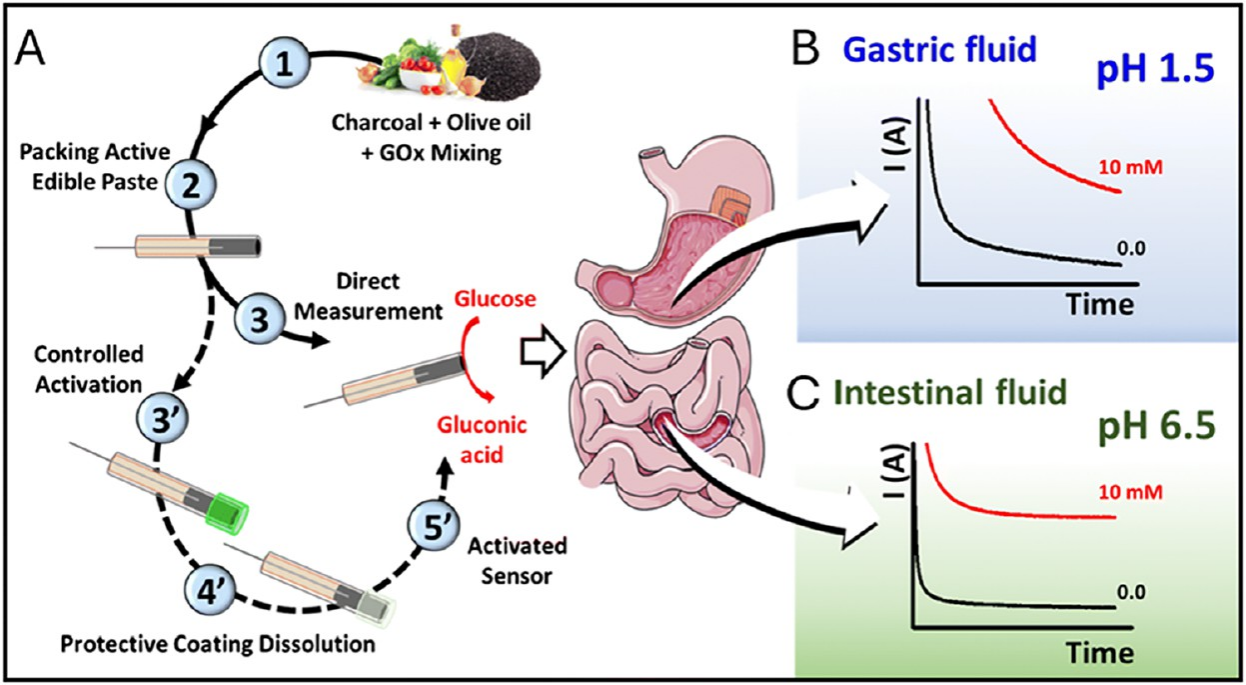
Edible electrochemical biosensor for the determination of glucose in gastric and intestinal fluids. (A) Schematics showing the preparation and application of the proposed device. (B) Examples of chronoamperograms in gastric fluid. (C) The same curve in the intestinal fluid. Reproduced with permission from Montiel et al.^403^ © 2019 Springer Nature.

Engineered bacteria are promising for sensing markers of stomach and bowel diseases, as they can be encapsulated within ingestible devices. This was done, for example, to detect colonic inflammation through luminescence and fluorescence outputs.^335^ Ingestible biosensors have also been developed to monitor gastrointestinal health. For instance, an ingestible microbioelectronic device was used for biomolecular detection *in situ* through luminescence readout electronics that communicate wirelessly with an external device. The device implanted in a porcine model could detect gastric bleeding by sensing levels of heme in real time.^404^

Gastric fluid pH, on the other hand, is also an important indicator of physiological status.^405^ The stomach’s characteristic acidity is essential for digestion and pathogen defense. Significant deviations from this acidic range can signal important clinical events. For instance, a rise in pH following gastric surgery might indicate anastomotic leakage, where alkaline fluids from the intestine infiltrate the surgical site.^354,406^ Conversely, abnormally high pH could suggest impaired acid secretion (achlorhydria), linked to conditions like pernicious anemia or chronic gastritis.^407,408^ For temporary monitoring, such as during postoperative recovery periods (typically weeks), bioresorbable, wireless, passive sensors offer a particularly compelling approach. These devices eliminate the need for onboard batteries and avoid the requirement for subsequent surgical retrieval, degrading harmlessly over a predetermined time frame.

A prominent strategy for passive pH sensing utilizes inductor-capacitor (LC) resonant circuits.^409^ In this design, the sensor itself contains no power source; instead, it relies on inductive coupling with an external reader device. The external reader generates a time-varying magnetic field that energizes the sensor’s LC circuit, causing it to resonate at a specific frequency. By sweeping the reader’s frequency and detecting the corresponding change in impedance reflected by the sensor, this resonant frequency can be determined from outside the body. Implementations often engineer the inductive component (L) to be pH-sensitive, as in a patterned zinc (Zn) foil structured into a serpentine geometry to accommodate deformation. This inductor was then encapsulated within a pH-responsive hydrogel. For example, a copolymer hydrogel like poly­[2-(diisopropylamino)­ethyl methacrylate]-*co*-poly­(ethylene glycol)­diacrylate (PDPAEMA-*co*-PEGDA) served this purpose well since it contained tertiary amine groups with a p*K*_a_ around 6.3. In the highly acidic environment (pH < p*K*_a_), these amines become protonated, acquiring positive charges. The resulting electrostatic repulsion between these charges within the polymer network, combined with increased osmotic pressure, causes the hydrogel to swell. This swelling mechanically deforms the embedded Zn inductor, increasing its physical dimensions and, consequently, its inductance (L). Since the resonant frequency of an LC circuit can be affected by changes in inductance and capacitance, in general, the increase in L leads to a decrease in the resonant frequency. Therefore, a measurable change in resonant frequency provides a wireless, passive signal indicating a shift toward less acidic (or more alkaline) conditions. The selection of materials is paramount: zinc provides conductivity for the inductor and exhibits well-characterized bioresorption kinetics, while bioresorbable polymers like poly­(lactic-*co*-glycolic acid) (PLGA) are used as dielectric materials for the capacitor (C). Meticulous encapsulation, often using biocompatible waxes or other polymers, is crucial to protect sensitive components, especially the capacitor, from premature degradation or signal drift due to biofluid permeation. This ensures that frequency shifts during the intended operational lifespan reliably reflect pH-induced inductance changes. Similar challenges related to material stability and response specificity in harsh biological fluids are encountered in sensor development for other environments, such as the urinary tract, where mineral encrustation poses significant problems.

Another important application is the simultaneous detection of iron species (Fe­(II)/Fe­(III)) in gastric fluid. While the small intestine is the primary site for iron absorption, understanding iron’s chemical state (speciation) within the stomach is vital for assessing the bioavailability of dietary iron and the efficacy of oral iron supplements, as Fe­(II) is generally more readily absorbed than Fe­(III). Electrochemical sensors using nanocomposite-modified electrodes can achieve this simultaneous quantification. For instance, platforms incorporating nitrogen-doped carbon quantum dots (N-CQDs), AgNPs, and β-cyclodextrin (β-CD) demonstrated this potential.^410^ In such systems, N-CQDs provide high electrical conductivity and electrocatalytic surfaces, enhancing signal transduction; AgNPs further improve electron transfer kinetics and increase the active surface area available for reaction; and β-cyclodextrin acts as a molecular recognition element, utilizing its hydrophobic inner cavity to selectively bind iron ions, thereby enhancing sensitivity and selectivity against potential interferents in the gastric matrix. Voltammetric techniques like DPV or SWV are typically employed because they can resolve the distinct electrochemical signals (peak potentials) corresponding to the oxidation or reduction of Fe­(II) and Fe­(III). The ability to differentiate these species offers a more nuanced understanding of gastric iron chemistry, informing diagnostics for iron-related disorders and guiding the design of more effective supplements.

A critical area is real-time gas monitoring, particularly for hydrogen sulfide (H_2_S), which is relevant in gastrointestinal homeostasis, including modulating mucosal defense mechanisms, regulating inflammation, and influencing gut motility. Aberrant H_2_S levels have been implicated in gastritis and inflammatory bowel disease. Miniaturized, ingestible electrochemical sensors integrated into swallowable capsules allow for real-time H_2_S monitoring directly within the GI tract.^411^ These sensors often employ working electrodes modified with materials selected for high selectivity and catalytic activity toward H_2_S oxidation or reduction. For example, a gold (Au) electrode modified with a Nafion solid-polymer electrolyte layer was selective for H_2_S. Integrated within a wirelessly communicating capsule, such sensors demonstrated linear responses to H_2_S concentrations relevant to both physiological and pathological states (e.g., 0.21–4.5 ppm). Significant engineering challenges include ensuring efficient diffusion of the target gas to the sensing surface within the confined space of an encapsulated device, maintaining long-term sensor stability and calibration *in vivo*, and adequately addressing the power budget and data transmission requirements for ingestible electronic systems.

The adaptability of electrochemical strategies extends to other relevant analytes. Examples include detecting metabisulfite,^412^ a potentially sensitizing food additive whose presence and concentration in the stomach could be clinically relevant, or monitoring key ions like sodium (Na^+^), potassium (K^+^), and chloride (Cl^–^) that influence osmotic balance and gastric secretions.^413^ Furthermore, research is exploring alternative sensor form factors, such as flexible, thread-based electrochemical sensors.^414^ These formats offer potential advantages in conforming intimately to the gastric mucosa, enabling more localized measurements at the tissue interface.

It has already been mentioned that any implantable or ingestible gastric sensor must possess exceptional robustness to survive in the highly aggressive gastric environment. Devices must reliably function despite exposure to extreme pH levels, which can plummet to as low as 1.5 during fasting and rise toward neutral after meals, creating large, rapid fluctuations. Furthermore, they must withstand potent enzymatic activity, particularly from pepsin, which actively digests proteins, potentially degrading sensor components or protective layers. Continuous mechanical stresses from gastric motility, including grinding and mixing motions (peristalsis), also impose significant physical demands. Consequently, robust protective coatings or encapsulation layers (e.g., using materials such as medical-grade silicones, parylene, or specialized polymers) are indispensable. These protective barriers must be impermeable enough to prevent damage while still allowing for controlled and predictable diffusion of the target analyte (like glucose, pH ions, or specific biomarkers) to the active sensing element.

Gastric sensors must also be compatible with biological tissues. All materials intended for contact with the gastric mucosa or internal environment must meet compatibility standards (e.g., ISO 10993)^415^ to be nontoxic, nonimmunogenic, and elicit only a minimal inflammatory response. Failure to do so can lead to tissue damage, chronic inflammation, or rejection. Also, a closely related and persistent challenge is biofouling. This process involves the rapid adsorption of biomolecules, primarily proteins and mucus components, onto the sensor surface immediately upon exposure to gastric fluid. This initial layer is often followed by the adhesion of cellular debris and bacteria, forming a complex biofilm. This biofouling layer presents a major hurdle because it can physically obstruct the path of the target analyte to the sensor, passivate reactive electrode surfaces, thereby reducing sensitivity, and alter the sensor’s calibration and overall performance, ultimately limiting its functional lifespan. Therefore, effective antifouling strategies are required. This often involves surface modifications, such as grafting hydrophilic polymers like PEG^416^ or zwitterionic materials,^417^ applying specialized hydrogel coatings,^418^ or designing micro/nanostructured surfaces that discourage adhesion,^419,420^ all aimed at maintaining a clean and accessible sensor interface for reliable long-term operation.

Further insights can be drawn from advancements like an ingestible robotic interface device designed for chronic electrostimulation.^421^ This ingestible robotic interface device is released when its gelatin capsule dissolves in the stomach. Once deployed, a built-in magnet enables precise navigation to the target location using an external magnetic field. After reaching the desired site, its thin, flexible structure—made of circuits embedded within a soft elastomer—conforms closely to the stomach lining. A hydrogel adhesive activates upon contact with moisture, securing a strong and long-lasting attachment for several days. Finally, the device operates without a battery, receiving power and programmable stimulation commands wirelessly through near-field inductive coupling at a frequency of approximately 13.56 MHz. Adapting such principles—specifically, flexible conformal designs, robust bioadhesion for stable positioning and consistent fluid access, guided navigation, and wireless battery-free powering—offers a pathway to gastric fluid sensors capable of reliable, long-term *in situ* monitoring of crucial biomarkers within the challenging gastric lumen.

Sustained operation and data retrieval indeed pose engineering challenges for gastric fluid sensors (see Section 10 for more details on energy harvesting). Passive sensors, which require no internal battery and are powered wirelessly by an external reader (often via inductive coupling or NFC), offer simplicity but are typically limited by a very short communication range (millimeters to centimeters) and high sensitivity to the precise alignment between the sensor and reader. Active sensors, incorporating miniaturized batteries, possess greater operational flexibility and potentially longer communication range. However, they necessitate highly reliable, energy-dense, and safe power sources encased to prevent the leakage of potentially toxic contents into the body. Reliable wireless data transmission through the intervening biological tissues is also crucial. This requires power-efficient antenna designs and robust communication protocols optimized for low-power consumption and tissue penetration, such as the Medical Implant Communication Service (MICS) band,^422^ Bluetooth Low Energy (BLE),^423^ or NFC,^424^ each with different trade-offs regarding range, data rate, and power usage (see Section 9 for more details on signal acquisition). An emerging, though still largely experimental, requirement being explored is the potential for energy harvesting directly from the gastric environment, for instance, using tiny generators harnessing mechanical energy from stomach movements, which could potentially eliminate battery limitations in the future.^425^

### 4.3 Interstitial Fluid

Interstitial fluid (ISF) is a major biological fluid that contains a rich and diverse variety of biomarkers and analytes. ISF is also one of the most epidermally accessible biofluids with a similar composition to blood, making it an ideal candidate for the detection of biomarkers.^426–428^ While blood remains the primary biofluid for the measurement of numerous biomarkers, its collection requires the use of invasive hypodermic needles, necessitating professional expertise for safe retrieval.^429,430^ ISF shows distinct advantages including greater volume, lower biointerference, and high clinical relevance. To fully utilize ISF sensors, researchers have developed biosensors capable of extracting biomarker data in a minimally invasive manner.^431^

One important application is targeted to diabetes patients,^432,433^ to avoid needle injections or finger-pricking.^434,435^ Kim et al. developed a subcutaneously implantable electrochemical biosensor that detects minute changes in the dielectric permittivity, which are then correlated to changes in glucose levels.^436^ The device consists of a circuit that responds to glucose-induced dielectric changes by altering its resonant frequency, which is then wirelessly transmitted to an external reader. *In vitro* results demonstrated the ability to measure glucose concentrations from 50 to 500 mg/dL with a sensitivity of 2.3 kHz per mg/dL.^436^ Another subcutaneous implantable biosensor utilized electrical impedance spectroscopy to analyze the electrical properties of ISF, which depend on the glucose level.^431^ The data from this implantable bioimpedance spectroscope are transmitted to an external reader for analysis. *In vivo* experiments were conducted on pigs to evaluate the sensor’s performance. After a 14-day postsurgical recovery period, intravenous glucose tolerance tests were performed, during which 236 subcutaneous bioimpedance measurements were collected. Blood glucose levels ranged from 77.4 to 523.8 mg/dL during these tests. The study found a strong correlation between impedance measurements and blood glucose levels.^431^

Continuous glucose monitoring with wearable devices via measurements collected in the subcutaneous ISF is revolutionizing diabetes management.^437^ Expanding such technologies beyond glucose to a broader range of clinically important molecules, such as biomarkers (e.g., creatinine for renal function or interleukin-6 for the detection of sepsis) and narrow-therapeutic-index drugs (e.g., toxic, “last-line-of-defense” antibiotics), could transform the personalization of clinical care. For example, the ability to measure drug concentrations *in situ* in the blood or ISF could enhance the accuracy with which drug doses are personalized to each patient. A problem with extending the technology underlying traditional glucose monitoring to other molecular analytes is that it relies on the transformation of glucose into an easily detectable, redox-active product via the action of a redox-active enzyme. Thus, while this approach has been expanded to the *in vivo* measurement of a limited number of other molecules, including lactate (using lactate dehydrogenase),^438^ acetyl choline (choline oxidase),^439^ and glutamate (glutamate oxidase),^440^ the absence of suitable redox transforming enzymes renders the vast majority of clinically relevant molecular species impossible to capture using such sensors. For example, while one can broaden enzymatic electrochemical sensors to a much wider range of molecular analytes by using potentiometric approaches (i.e., “ion-selective electrodes”) to detect the protons or other ionic species produced when hydrolytic enzymes degrade their targets, this approach has seen almost no successful application to *in vivo* measurements. Specifically, the first such potentiometric, enzymatic sensor, which used urease to target urea, was reported more than 55 years ago,^441^ but only a single potentiometric enzymatic sensor has ever been reported to work *in vivo*—a penicillinase-based penicillin sensor^442^—in work that has not been expanded upon in the 5 years since it was originally published.

Given the difficulty of expanding *in vivo* enzymatic sensors to any more than a handful of analytes, in recent decades, the biosensor field has expended significant effort on affinity-based sensors. That is, to say, sensors that rely on the recognition by a receptor rather than employing the chemical transformation of their analytes. Affinity-based sensors, however, face signal transduction challenges, as most biomolecular receptors (e.g., antibodies) do not produce a measurable signal upon binding their targets. Hence, the development of affinity-based sensors that support measurements in blood or ISF *in vitro*, much less *in vivo* (where, for example, exogenous reagents cannot be easily added), has proven a significant hurdle. For example, despite an enormous volume of literature describing sensors that detect binding to surface-attached receptors via changes in the optical properties of the surface (e.g., surface plasmon resonance), the mass or steric bulk loaded on the surface (e.g., microcantilevers and quartz crystal microbalances), and the electric field at the surface (e.g., field-effect transistors), only a vanishingly small number of such sensors have been reported to work in unprocessed bodily fluids *in vitro*, much less *in vivo* ^443–445^ (for an exceedingly rare counter example, see ref 446). The main problem is, as previously mentioned in different sections of this review article, biofouling: the nonspecific adsorption that inevitably occurs when an artificial surface is placed in unprocessed bodily fluids also changes the optical properties, mass, and electric field on the sensor surface, producing a signal that is indistinguishable from that caused by analyte binding.

How, then, do we solve the “signal transduction problem” that has prevented affinity-based sensor approaches from deployment in blood and ISF? A glance at nature suggests an alternative to this quandary. Specifically, naturally occurring bioreceptors designed by evolution such that they undergo a significant physical change, such as a conformational change or a change in oligomerization state, upon target binding, which is used to transduce the binding event into an easily measurable output that is not “spoofed” by nonspecific binding.^447^ The question, then, is how to “re-engineer” normally nonresponsive biomolecules into receptors that undergo such a binding-induced physical change. To date, two approaches to this end have been shown to work *in vivo*.

The most well-explored means of re-engineering biomolecules to undergo a binding-induced conformational change is to destabilize them, so that they are unfolded in the absence of their target analyte but then fold upon binding.^448^ This couples recognition to an enormous change in polymer physics which, in turn, can be used to produce optical or electrochemical outputs. Because of the relative paucity of electroactive species (at any reasonable potential) in biological fluids, the latter approach is particularly amenable to measurements performed in the living body. The most successful application of binding-induced conformational changes in *in vivo* biosensors so far has been the electrochemical aptamer-based (EAB) sensor platform. First introduced some 20 years ago,^449^ EAB sensors uniquely blend the broad applicability of affinity-based sensors with the selectivity required to perform useful *in vivo* measurements. At their core, these sensors utilize aptamers that are re-engineered to undergo a significant binding-induced conformational change.^450^ They are modified with an electrochemically active “redox reporter,” and anchored onto the surface of an interrogating electrode. The structural shift they undergo upon target binding alters the rate of electron transfer from the redox reporter, producing an easily detectable electrochemical signal. Compatible with various electrochemical analysis techniques—including SWV, chronoamperometry, and impedance spectroscopy,^451–454^ EAB sensors allow for a continuous, real-time molecular monitoring *in situ* within the living body.

Since first adapted to *in vivo* deployments in 2017,^452^ a half-dozen research groups have described EAB sensors supporting the real-time, *in vivo* measurement of about a dozen different analytes.^455–465^ These have been shown to support real-time, multihour measurements *in situ* in the body, with the large majority focusing on intravenous and intracranial sensors for measuring plasma drug and metabolite concentrations. This includes seconds and even subseconds resolution measurements of a number of drugs.^455–458,464,466^ and metabolites^465^ *in situ* in the jugular veins and antibiotics and psychoactive drugs in the brains^463,467^ of live rats. The real-time concentration information provided by these sensors also provides unique opportunities for the high-precision control over *in vivo* drug and metabolite concentrations. Specifically, EAB sensors have been shown to support closed-loop feedback control over *in vivo* molecular concentrations in the blood,^452,455^ brain,^467^ and solid peripheral tissues.^468^ This provides an unprecedented ability to maintain constant *in vivo* drug concentrations, or to follow predefined, time-varying concentration profiles.

Of course, intravenous (much less intracranial) sensors do not easily translate into convenient, easily wearable technologies. Thus, recent years have seen increasing focus on the adaptation of EAB sensors to measurements performed in the dermal or subcutaneous ISF. These measurements have been performed using submillimeter microneedles,^460^ hollow, fluid-^469^ or hydrogel-filled microneedles,^470^ and millimeter-scale wire sensors,^468,471^ with the hollow-needle approach even having been deployed on human subjects. A problem with the measurement of molecular analytes in the ISF (or, indeed, most bodily fluids) is that it is plasma drug and biomarker concentrations, and not their concentrations in the ISF, that are the basis of clinical decision-making. Hence, it is essential that we learn how best to estimate plasma concentrations using minimally invasive measurements performed in the dermal ISF.^472^ Fortunately, preliminary efforts to this end suggest that, at the very least, the estimation of systemic (i.e., plasma) drug exposure (as “area under the curve” on time–concentration plots) from measurements collected in the ISF will prove straightforward. Specifically, for the two drugs investigated to date, tobramycin and doxorubicin, the correlation between exposure in the ISF and plasma exposure is predictively strong (*R* ^2^ > 0.95).^460,471^

As a scientific tool, EAB sensors also provide opportunities to measure drug transport between bodily compartments with unprecedented ease and time resolution, with recent examples including the transport of drugs between the plasma and both the brain^463^ and the subcutaneous ISF.^468^ Likewise, by providing researchers with a tool to manipulate molecular concentrations in the body with high temporal and concentration resolution, the use of EAB sensor-informed feedback control over *in vivo* molecular concentrations could likewise significantly impact biomedical research. For example, using EAB sensors to perform feedback control, researchers have eliminated subject-to-subject pharmacokinetic variability as a confounding variable and have even reproduced human pharmacokinetic profiles in rat animal studies.^466^

A second, recently developed alternative to EAB sensors, “molecular pendulum” sensors, may provide a simple yet powerful method to translate the binding of larger molecular analytes (i.e., proteins) into an electrochemical signal via changes in hydrodynamic drag.^473^ This approach builds on somewhat similar optical^474^ and electrochemical^475^ approaches. It employs a short, electrode-bound, double-stranded DNA element to which a receptor, such as an antibody, nanobody, or aptamer, and a redox reporter have been covalently attached at the distal end. When a positive potential is applied to the electrode, the negatively charged DNA is attracted to its surface, with the rate of the resulting motion depending monotonically on the hydrodynamics of the pendulum. That is, signal transduction in this sensor architecture relies on the increase in drag that occurs when target binding increases the hydrodynamic radius of the pendulum. This converts binding into a change in electron transfer rate that is easily measurable using chronoamperometry, at least for higher molecular weight analytes. The approach has not been applied to lower molecular weight analytes, as these do not significantly alter the hydrodynamic radius of the pendulum. Still, by swapping the target-recognizing receptor, the approach has been used for the detection of 10 different proteins spanning a wide range of sizes and charges. Preliminary results suggest that this sensor architecture supports measurements *in situ* in the body. Specifically, the approach has been shown to work for the detection of the cytokines interleukin-6 and tumor necrosis factor α and in the subcutaneous interstitial fluid of a live rat with 40 min time resolution.^476^

## 5 Wearable Device Formats

The production of wearable sensors and devices depends on a variety of engineering aspects, such as materials, design, and fabrication processes.^477,478^ Herein, we discuss the fabrication of various wearable devices to adjust to body parts, where they can acquire biosignals and monitor health. We focus on the fabrication methods to avoid being overly repetitive as several examples of the use of the different formats are given in other sections. Figure 16 illustrates some of the main fabrication methods and devices that will be discussed in the following subsections.

**Figure 16:**
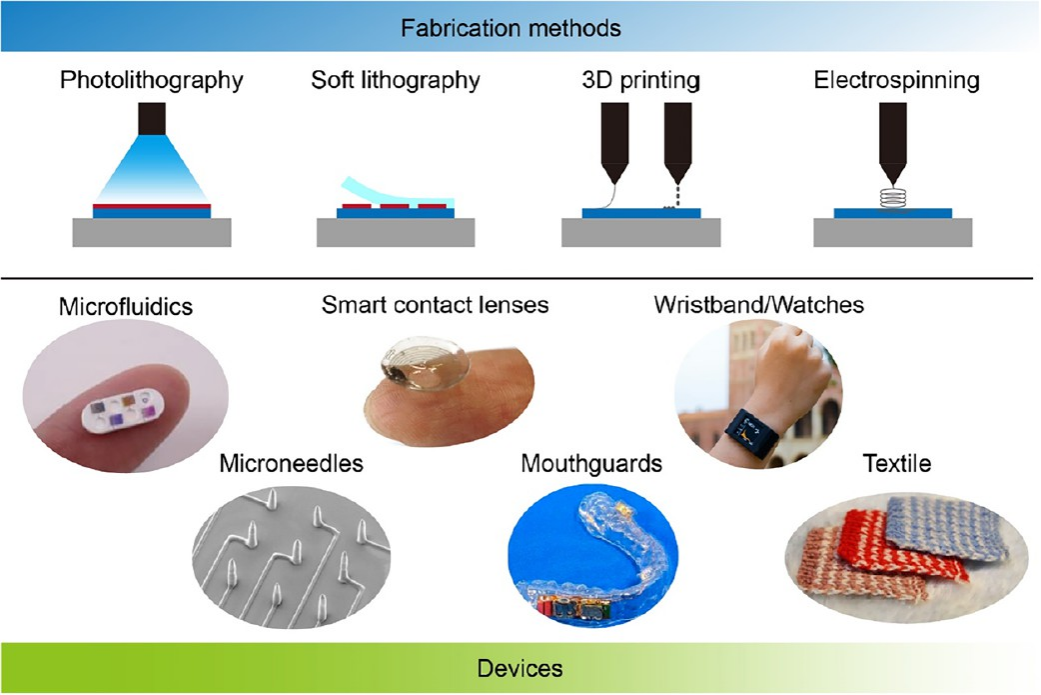
Fabrication methods for wearable devices. Microfluidics, Reproduced from Kim et al.^139^ Available under a CC-BY 4.0 license. © 2022 John Wiley & Sons; Microneedles, Reproduced from Kim et al.^479^ © 2024 American Chemical Society; Smart contact lenses, Reprinted with permission from Kim et al.^238^ © 2021 Springer Nature; Mouthguards, Reprinted from Ichikawa et al.^230^ Available under a CC-BY 4.0 license. © 2024 MDPI; Wristband/Watches, Reprinted with permission from Zhao et al.^480^ © 2020 American Association for the Advancement of Science; Textile, Reprinted with permission from Fan et al.^481^ © 2020 American Association for the Advancement of Science.

### 5.1 Microfluidics

Microfluidics is the science of precisely manipulating very small fluid volumes (nL to pL) using microscale systems. In wearable bioanalysis, microfluidic platforms serve for collecting fluid-type samples (e.g., sweat, blood, tears, saliva) using capillary forces to analyze biochemical markers in real time. In this section, swear-based devices will be used as an example of different microfluidic applications due to the numerous reports and their variations. They are mostly produced with soft lithography utilizing polymeric substrates, such as PDMS, due to their relatively low cost, rapid prototyping, and compatibility with soft and flexible substrates.^482–484^ This method involves the patterning of a master mold, followed by polymer casting and bonding to create microchannels.^139,485,486^ Song et al. developed a microfluidic sweat sensor patch on a flexible printed circuit board platform that can monitor sweat biomarkers (e.g., pH and Na^+^).^487^ To improve scalability, laser engraving of patterns onto flexible substrates has been employed in ambient conditions.^126,488,489^ These manufacturing techniques enable the formation of microfluidic channels directly, thereby reducing fabrication complexity. Additionally, 3D printing has been exploited to fabricate complex and customized microfluidic designs, offering precise control over geometry and feature size.^488,489^

Microfluidics can be used, for example, to address the challenge of low sweat rates coupled with the low density of sweat glands in developing wearable sweat sensors. The human skin has the highest density of sweat glands in palms and soles (∼600 glands/cm^2^), but the average sweat gland density is only ∼150 sweat glands/cm^2^.^24^ Moreover, each sweat gland produces anywhere between 1 and 20 nL/min sweat with a secretory pressure between 3 and 70 kPa, depending on an individual’s age, diet, environment, and physiological state.^490,491^ Microfluidics also enables fluid handling via active/passive valves and pumps, stabilizes the naturally occurring pulsating sweat flow to a more laminar flow for reliable sensor signals, and supports inclusion of sample pretreatment capabilities. It can also be used to filter out undesired constituents that can negatively affect sensor response. For example, naturally produced sweat contains a small amount of sebum, an oily substance produced by the sebaceous glands present in the hair follicles. The oily constituents of the sebum can deposit onto sensor surfaces and cause signal drift and biofouling. Microfluidic devices have been developed to address this issue which incorporate porous polypropylene-based filters that absorb sebum before the sweat is introduced to the sensors.^492^

Key challenges for wearable microfluidics include ensuring mechanical durability under continuous wear and achieving reliable fluid flow control in ultrathin devices. A range of materials has been utilized in wearable microfluidics, including silicones,^493–498^ paper,^499–502^ fabrics,^503,504^ and flexible plastics^505^ fabricated using techniques such as soft lithography,^493^ laser cutting,^505–507^ and 3D printing.^128,508–510^ Wearable microfluidics have been employed to house a rich variety of electrochemical,^508,509,511–514^ colorimetric,^27,132,135,146,515,516^ fluorometric,^123,158–160^ and surface-enhanced Raman spectroscopic^517–522^ based transducers to detect biomarkers such as metabolites,^494,520,523,524^ electrolytes,^118,492,500,525,526^ proteins,^102,527–529^ and minerals.^176,507,530,531^ The simplest version of wearable microfluidics involves an inlet for the sweat to enter, an outlet to expel old sweat, a sensing chamber where the sensor is embedded, and a connective channel that links all the above three regions of the device. While simple in design, care must be taken to ensure that the dimensions and surface properties of the microfluidics allow rapid capture and transport of the sweat from the skin to the sensor and then ultimately out of the device. Here, advanced computer simulation models provide researchers with insights into designing the architecture and selecting the right materials for developing microfluidics that meet their needs.^532^

Simple microfluidic designs are mostly suitable for applications that involve high sweat rates, for example, in the case of active users. However, for the majority of applications, users will have low sweat rates. A strategy to solve this involves the use of locally administered sweat-inducing drugs^197,533,534^ or raising the local skin temperature via Joule heating to induce sweat synthesis.^525^ While such approaches facilitate a higher sweat rate, the reliance on external factors to induce sweating artificially and their effect on modulating the natural sweat composition remain issues to investigate. The efficient capture of naturally produced sweat may be done with multiple inlets^494,524,525,527,535^ and materials with capillary action (e.g., paper). One such embodiment involves a paper microfluidic array with 96 inlets distributed across 8 interconnected input channels around the collection zone for efficient collection of sweat.^501^ The multiple circular and semicircular inlets, inlet density, number and length of inlet channels, and dimensions of the harvesting zone were rationally optimized based on probability analysis to capture 100% of the sweat generated in the skin region where the device is applied.

Researchers are also taking inspiration from nature to reach an efficient sweat harvesting.^511^ For example, a device with open microfluidic structures inspired on tree frog toe pads was used for rapid collection of sweat at low flow rates (∼0.1 μL/min·cm^2^).^536^ Taking cues from the hexagonal epithelial cells on the toe pads of the tree frog *Rhacophorus pardalis*, the team designed microfluidics with rigid hexagonal walls covered with soft elastomeric materials. The rigid walls helped maintain the open microfluidics structure, while the soft elastomeric covering enabled conformal attachment of the device to the human skin. The strong capillary force exerted by the microfluidic design allowed the device to capture sweat and perform sweat analysis with >75 nL of sweat collection in 45 s. Another nature-inspired microfluidic device was based on Ginkgo biloba veins to include microfluidic channels with a 5° wedge angle that facilitates 40% higher flow rate than traditional channels with a 0° wedge angle.^523^ The unique Ginkgo biloba mimicking microchannels achieve higher fluid transport due to the elevated capillary forces created by the geometry of the channels. Rapid and directional collection of sweat was also obtained with Janus-based microfluidic membranes.^517,537–539^ A typical approach for developing Janus-based membranes involves creating a mesh-like hydrophobic layer in direct contact with a highly mesh-like hydrophilic layer. The hydrophobic layer is mounted on the skin, while the sensor is interfaced with the hydrophilic side. The two layers are usually made via electrospinning, which yields large sheets with reproducible pore size and density. Computer simulations provide a valuable understanding of the working principle of these hybrid membranes.^517^ Sweat droplet contacting the hydrophobic layer experiences opposing hydrostatic pressure (*P*_H_) and Laplace pressure (*P*_L_). When *P*_H_ exceeds *P*_L_, the sweat droplet penetrates through the hydrophobic layer and comes in contact with the hydrophilic layer, where capillary pressure (*P*_C_) dominates and serves as the driving force to rapidly pull the remainder of the fluid into the hydrophilic region and enable lateral spreading of the liquid within the hydrophilic layer. Once the sweat spreads across the hydrophilic layer, *P*_H_ decreases drastically, thus disincentivizing sweat from re-entering the hydrophobic layer. Janus-type architectures can also be fashioned in the form of yarns for rapid, pumpless capture and transport of sweat.^540^ Using osmotic pumps for capturing sweat represents yet another path for uses wherein the sweat rate is low.^541,542^

In addition to enabling efficient sweat collection, wearable microfluidics also aid in advanced fluid handling, which is necessary for various sensing applications. In this context, innovations in microfluidic valves and pumps come into play. Capillary burst valves are among the most explored valve designs.^494,543–546^ The burst pressure (BP) for valves located within rectangular channels is based on the Young–Laplace equation

$$\mathrm{BP}=-2\sigma \left[\frac{\cos\theta_{I}^{*}}{b}+\frac{\cos\theta_{A}}{h}\right]$$

where σ is the surface tension of the liquid, θ_A_ is the contact angle of the channel, θ*_I_ is the min­[θ_A_ + β; 180°], β is the diverging angle of the channel, and *b* and *h* are the width and height of the diverging section, respectively. The sweat can open these valves and travel forward only when the sweat pressure is higher than BP. Such valves thus allow a simple geometry-guided passive valving methodology for controlling the direction of the sweat flow within the microfluidics. Selectively patterned hydrophobic and hydrophilic regions within the microfluidics can also be implemented as valves.^506,547,548^ For example, in one embodiment, hydrophobically patterned channels route incoming sweat toward specific sensing regions within the device.^547^ When these regions are filled, the sweat is directed toward a superabsorbent polymer-based gate located at the entrance of the sensing region, which rapidly expands and closes the sensing region from accepting more sweat. The sweat is then forced to travel to subsequent sensing regions where the entire process of sensing region filling and thereafter superabsorbent polymer-based gate closing occurs, thus allowing a time-sequenced analysis of sweat. Along similar lines, time-sequenced sweat routing can also be achieved by strategically depositing a water-soluble polymer within the microfluidic channels that initially stop the sweat from flowing past it, but over time, the polymer dissolves, thus allowing sweat to flow across and travel to sensing regions that were previously inaccessible to it.^549^

While the above-mentioned approaches of passive valves serve in developing simple, low-cost devices with fluid routing capabilities, a major challenge involves their inability to completely isolate the liquids across the valves, which can result in sample intermixing and contamination. A recent work attempts to address this issue by demonstrating liquid-bridge-cutting valves.^550^ It builds on the concept of capillary burst valves and leverages the liquid bridge breakup principle that allows the device to separate liquids and fill the resulting gap with air. Each sensing region is loaded with filter paper placed at a small offset from a capillary burst valve, creating an air gap between the paper and the valve. When sweat arrives at this valve, the front-end bulges as pressure builds up until the liquid front touches the tip of the filter paper, forming a liquid bridge. At this point, the capillary forces within the paper draw in the sample until the entire sensing chamber is filled. Upon complete filling of the chamber, the liquid bridge breaks and recreates the small air pocket between the paper and the valve, which stops the flow of any new fluid into the filled chamber. Such air pocket-based designs can also be used to control the flow rate, which can be helpful in obtaining more reliable data.^550^ Tesla valves represent another type of passive valve design for fluid flow control.^551,552^ Variations of Tesla valves that include single-stage, multistage, and derivatives of the original design can be employed to achieve unidirectional flow of liquid.^553^

Active valves embedded in wearable microfluidics that are activated by heat,^554,555^ electrical,^556^ and electromagnetic^557^ triggers have also been developed for more stringent control of sweat flow within the device. One example of a heat-triggered valve involves the use of proprietary thermoplastic microspheres encapsulating a gas (Expancel).^555^ The encapsulated gas reversibly expands when heated. The device had affixed microspheres to a thin layer of silicone and embedded at specific regions within the microfluidic channels. Thin-film heaters, powered by a wearable flexible electronic module and located beneath the microsphere-functionalized silicone layer, provide the heat necessary to expand the microspheres, which ultimately block the channel and stop the fluid flow. Electrical trigger-based electrowetting valves can also be employed for controlling sweat flow.^556^ A device exemplifying this approach was made with inject-printed silver electrodes coated with a self-assembled monolayer of perfluorodecanethiol embedded at the floor of the microfluidic channels. This monolayer renders the electrodes hydrophobic, which hampers sweat from flowing over it, thus acting as a valve. However, applying 4 V for ∼17 s reversibly transforms the hydrophobic coating into a hydrophilic layer, thus enabling the liquid to cross. Such valves can be used for retaining fluids for at least 9 h.^556^

Strategies for incorporating micropumps within wearable microfluidics have also been investigated. Passive capillary pumps may contain microposts within the microfluidic chambers.^494^ They can be integrated with inlet channels that possess continuously tapering cross-sectional areas to augment the sweat collection process.^558^ Other strategies include active mechanisms, as with finger-actuated pumps in which paper or silicone-based pull tabs attached to the soft microfluidics allow the user to exert suction forces to drive the collected sweat sample to specific regions.^183,559,560^ Piezoelectric materials have also been used in wearable pumps^561^ by incorporating aluminum oxide/carbon nanotubes core–shell nanofillers within a ferroelectric polymer. The polymer composite was sandwiched between two electrodes that were biased at 160 V and were largely deformed (bending angle: 74°), thus enabling the system to behave like a piezoelectric pump. Another strategy involves a portable electrohydrodynamic pump (10 × 2 × 1.05 cm).^562^ It includes three main components: an interdigitated electrode system, flow channels, and an insulating fluid. The electrical field applied across the electrodes drives the fluid forward. The device utilizes the insulating fluid due to its low conductivity (10^–9^ S/m), high dielectric withstand voltage (5–6 kV at a gap: 0.5 mm), and a dielectric constant of 6.1. Moreover, it has a boiling point of 76 °C, high thermal conductivity, ultralow toxicity, zero ozone depletion potential, zero flash point, and nonflammability. To reduce the size of the electrohydrodynamic pumping system, triboelectric self-powered devices can be used.^563^ The device includes a rotatory triboelectric nanogenerator that collects ambient energy and converts it into a high-voltage power source for powering an electrohydrodynamic pump that can generate a maximum pressure of 4.49 kPa and a maximum flow rate of 502 mL/min.

Most chemical sensors, especially those relying on enzymes, aptamers, and antibodies, require specific incubation periods, washing steps, and multistep detection protocols. Wearable microfluidics are not designed to achieve these capabilities. To address this issue, a new class of wearable microfluidics that offers on-demand sensing has been reported.^564,565^ These devices employ the above-mentioned pumps and valves to introduce captured sweat to sensors at user-defined time points, thus allowing complete control over the incubation period. One of the earliest examples had a series of finger-actuated pumps and capillary burst valves strategically located within the microfluidic architecture. This allowed the user to initiate sensor reactions on-demand, thus opening the possibilities toward true lab-on-skin devices. Additional capabilities were introduced by embedding user-activated 3D-printed rotational routers that can direct collected sweat to different regions within the microfluidic patch for fluid handling and sensing applications.^565^

The many advances made in the field of wearable microfluidics as reported here are a testament to the collaborative efforts put together by a diverse set of researchers, including mechanical, chemical, and biomedical engineers working closely with material scientists and clinicians. As articulated in this section, the field of wearable microfluidics has come a long way from simple designs reported less than a decade ago to advanced systems that can perform complex fluid handling in a miniaturized and low-power form factor, which is amenable to the unique needs of wearable sensing applications.

### 5.2 Microneedles

Microneedles (MNs) are needle-like vertical structures typically in the hundreds to thousands of micrometers range in height, with the aim to pierce tissues at defined depths. One of their biggest advantages when used in humans is that MNs application can be pain-free if their height is not enough to reach the nerves under the epidermis. Also, they provide a minimally invasive alternative for creating microchannels in the skin for sample collection or for the direct acquisition of electrical and chemical signals.^566^ Microneedles can be solid, coated, hollow, or dissolvable, depending on the desired application, being typically built using silicon, metals, ceramics, or polymers.^567^ Multiple construction techniques can be applied. For example, silicon (Si)-based microneedles are normally fabricated with photolithography and reactive ion etching (RIE), ensuring high mechanical strength and precise geometries.^568^ For flexible and biodegradable microneedles, polymers are processed using molding or 3D printing techniques.^569–571^ After defining 3D polymeric structures, thin metal layers can be deposited selectively on the microneedle tips through electroplating or laser micromachining, creating precise recording sites.^572^

A variety of applications exist for MNs. Solid ones can be used for drug administration if they are properly coated, whereas hollow MNs are applied for minimally invasive ISF low-volume sampling. Conductive MNs can be used for ISF sensing and biosensing inside skin. Biodegradable MNs can be designed to naturally dissolve and release chemical agents in the skin. Significant advancements have been made in ISF glucose sensing, particularly with commercial, needle-based continuous glucose monitoring devices that provide accurate, calibration-free, and real-time glucose measurements in subcutaneous tissue.^573^

Concerns regarding microneedles often include minimizing immune responses caused by their penetrating structures, which can lead to the inflammation or scarring of biological tissues. To mitigate these, strategies are used to reduce the elastic modulus of microneedles to levels comparable to those of biological tissues by using extremely soft materials.^574,575^ For example, Kim et al. utilized liquid metals (LMs) as microneedle materials sufficiently stiff to penetrate the skin when frozen and become softer upon penetration because of their low melting point, causing no significant immune responses on the skin after implantation.^479^

Despite their variety of possible uses, MNs are still not ubiquitous in medicine and other fields such as agriculture because of their cumbersome fabrication routes. When using photolithography, for example, clean rooms and multiple steps are required, resulting in high costs and low scalability. As previously mentioned, alternative methods, as additive manufacturing, have been implemented for MNs fabrication, but they are limited in terms of resolution, scalability, flexibility, and materials portfolio. Conductive MNs are challenging to fabricate with these techniques in a single step. Rosati et al.^569^ introduced a method to produce MNs in a single step on a flexible plastic substrate. They used inkjet printing of AgNPs with a research-grade printer by controlling the kinetics of the curing of the AgNPs ink when in contact with the substrate. The MNs presented conductivity immediately after the fabrication process, which consisted of the layer-by-layer, precise deposition of picoliter drops of the AgNPs ink. Different shapes and sizes were tested, identifying the limits of the method (e.g., verticality of the structures, minimum base width, maximum height). The mechanical properties of the inkjet-printed real-time cured AgNP ink have been characterized, obtaining a Young’s modulus and a hardness of 20 and 76% of those of bulk silver, respectively. The inkjet-printed MNs were tested as a proof-of-concept on plants, performing EIS measurements to characterize the plant hydration level. An increased signal-to-noise ratio and much lower impedance values were obtained in comparison with the same measurement performed by flat electrodes of the same material/size. This fabrication method is being further developed for use in patients, extending the portfolio of printing materials to gold nanoparticles and other nanomaterials, and implementing improvements to increase scalability and decrease the fabrication time per microneedle. A similar and even more scalable approach for the fabrication of fully-printed AgNP microneedles has been proposed by Baraniecki et al.,^1249^ studying the use of aerosol jet printing, and validating them for the monitoring of total ion conductivity in plants.

The fundamental sensing mechanism integrated into microneedle tips often involves electrochemical detection, particularly enzymatic or affinity-based electrochemical biosensing methods. For enzymatic sensors, enzymes are immobilized on the microneedle surface, catalyzing biochemical reactions that produce electroactive intermediates measured through chronoamperometric or voltammetric techniques. In affinity-based biosensors, aptamers or antibodies anchored to microneedle facilitate selective binding of target molecules, generating measurable changes in electrical properties that correlate with analyte concentrations. This direct microneedle–ISF interface eliminates external fluid extraction or dilution steps, significantly enhancing sensing accuracy, reliability, and response time. Optical transduction is an alternative due to the possibilities of label-free and real-time detection along with its high sensitivity, specificity, affordability, and small size.^576^

Microneedle sensors have been applied in numerous health and clinical contexts, illustrating their versatility and potential. Enzyme-based microneedle glucose sensors integrate GOx onto microneedle tips, typically using drop-casting, electrodeposition or entrapping methods to immobilize the enzyme, catalyzing the oxidation of glucose from ISF and generating measurable current signals proportional to the biomarker concentration.^577–579^ Continuous glucose monitoring is one of the most advanced clinical applications, delivering precise and minimally invasive measurements, reducing patient discomfort compared to traditional finger-stick methods. Such devices are used in diabetes management, enabling real-time glycemic control.^580^ Xiao et al. have expanded microneedle applications to monitor uric acid levels, using surface-enhanced Raman spectroscopy (SERS) integrated into MN arrays, with noninvasive assessment of health disorders. The 3D-printed microneedles are connected via a microfluidic channel to the SERS sensing chip. This sensor is based on nanogold arrays, which enhance the SERS signals, on a silicon wafer.^581^

The versatility of microneedle arrays further enables multiplexing by simultaneously monitoring different biomarkers. For instance, Tehrani et al. demonstrated integrated microneedle sensor systems capable of concurrently detecting glucose, lactate, or alcohol. These microneedles perform as electrodes and were fabricated using CNC milling and poly­(methyl methacrylate) (PMMA) as a base material. They functionalized the electrodes by electrodepositing a conductive polymer (o-phenylenediamine) and drop-casting enzyme solutions (GOx, lactate oxidase (LOx), and AOx) in a chitosan matrix to enhance enzyme stability and sensor performance. The combination of a nonionic surfactant and PVC was used as a diffusion-controlled film in a microneedle system for real-time analysis in freely moving human subjects, highlighting practical clinical potential.^582^ Other multiplexed microneedle arrays have been developed by Windmiller et al. for simultaneous monitoring of neurotransmitters like glutamate alongside glucose, offering valuable insights for neurological applications. They combine 3D-printed solid and hollow microneedles and employ electropolymeric entrapment of enzymes (glutamate oxidase and GOx) in poly­(o-phenylenediamine) thin films within the space created between both microneedle structures to ensure enzyme stability and selective analyte detection.^583^ A noteworthy example is the work presented by Huang et al., who developed a wearable microneedle-based sensor system for continuous transdermal monitoring of critical physiological ions, such as calcium (Ca^2+^), potassium (K^+^), and sodium (Na^+^). This system integrates ion-selective microneedle electrodes into a three-dimensional array, employing a fabrication strategy involving the assembly of planar microneedle sheets produced through laser micromachining of stainless steel, followed by electrochemical deposition of a gold layer and a conductive polymer (PEDOT:PSS). Ion-selective membranes were then applied via dip-coating, creating ion-selective electrodes capable of potentiometric detection of the aforementioned ions (Figure 17).^584^

**Figure 17:**
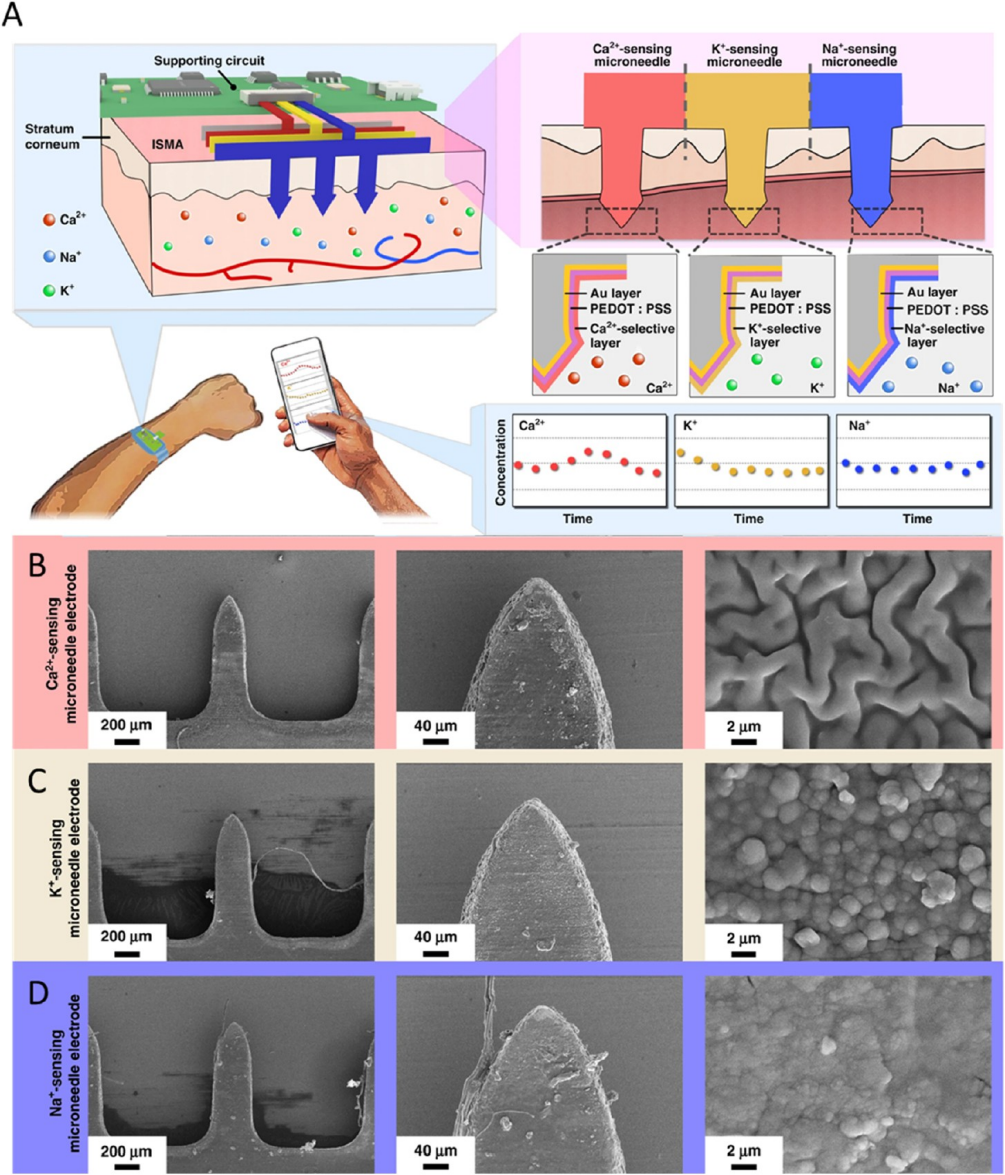
Ion-sensing microneedles. (A) Overview of the device, its wrist positioning, electrode modifications, and typical measurements. Optical and scanning electron microscopies from (B) Ca^2+^-, (C) K^+^-, and (D) Na^+^-sensing electrodes in different magnitudes. Reproduced from Huang et al.^585^ Available under a CC-BY 4.0 license. © 2023 Springer Nature.

Therapeutic drug monitoring (TDM) is another area where microneedle sensors have been applied. Goud et al. illustrate the substantial clinical potential of wearable electrochemical microneedle sensors for continuous levodopa monitoring in Parkinson’s disease management. The 3D-printed microneedle arrays were functionalized with enzymatic coatings via electrodeposition and incorporated two different electrochemical detection mechanisms (enzymatic-amperometric and nonenzymatic voltammetric detection), enabling continuous, real-time tracking of drug pharmacokinetics in ISF.^586^ Similar applications include microneedle-based sensors for real-time continuous monitoring of apomorphine in Parkinson’s treatment, demonstrating high sensitivity, selectivity, and good operational stability. Hollow microneedles were applied as working electrodes, being packed with carbon paste modified by rhodium nanoparticles, and apomorphine was detected by measuring its electrochemical oxidation peak.^587^ In this same context, Lin et al. developed a wearable microneedle-based electrochemical aptamer biosensor patch. The microneedles were fabricated from clinical-grade acupuncture needles, modified through electrodeposition of gold nanoparticles to enhance aptamer immobilization and electrochemical sensitivity. This biosensor permitted continuous and real-time monitoring of antibiotics, such as tobramycin and vancomycin.^588^

A wearable 3D-printed microneedle sensor was developed by Mishra et al.^589^ to detect organophosphate nerve agents. The hollow microneedles were packed with the corresponding electrode paste (carbon or Ag/AgCl ink) and functionalized with enzymes that generated an electrochemical signal. The same research group prepared similar microneedles but employed a computer numerical control (CNC) micromachining method. It facilitates continuous monitoring of opioids and nerve agents simultaneously, providing capabilities for emergency medical services and military personnel exposed to hazardous substances.^590^ Dermatological and oncological monitoring further expands the microneedle applications. Using a similar protocol for microneedle preparation from the previous work, microneedle sensors developed by Ciui et al. were used for melanoma screening by detecting the tyrosinase enzyme directly from the skin and drop-casting its substrate (catechol) onto the surface. This method offers a minimally invasive, real-time diagnostic capability.^591^

Theranostic systems have also been developed with microneedle wearable sensors, combining real-time biomarker monitoring with precise drug delivery. For instance, Razzaghi et al. introduced a wearable theranostic microneedle system featuring 3D-printed hollow microneedles. The system integrates colorimetric sensors to monitor physiological parameters such as pH, glucose, and lactate, alongside an ultrasonic atomizer, enabling remote-controlled, on-demand drug delivery. The colorimetric sensors were integrated by drop-casting enzymes (GOx, lactate oxidase, and HRP) or chromogenic dye solution (for pH sensor) onto filter papers interfacing with microneedles. This combination demonstrated rapid, minimally invasive sampling and precise medication administration, which is promising for chronic disease management and personalized remote healthcare.^592^

Several challenges must be addressed in microneedle-based wearable sensors, including ensuring consistent skin penetration, biofouling management, maintaining sensor accuracy under varying physiological conditions, and long-term biosensor stability. This requires integration of functionalized materials such as antifouling coatings and nanostructured surfaces and comprehensive clinical validation to establish accuracy and safety in diverse patient populations. Future developments will likely focus on enhancing sensor longevity and performance through improved material biocompatibility, incorporating self-powered systems, and integrating advanced wireless electronics. Such improvements will facilitate real-time remote health monitoring and fully autonomous operation, positioning microneedle-based wearable sensors at the forefront of personalized medicine, continuous health monitoring, and therapeutic management technologies.

### 5.3 Smart Contact Lenses (SCLs)

Smart contact lenses (SCLs) are wearable devices designed for varied functions, including the monitoring of physicochemical parameters (e.g., eye pressure) and biochemical species on tears. They have been fabricated with various methods that can integrate functional components with soft lens platforms.^236,593,594^ Photolithography, for example, is used for precise electrode patterning on the lens surface. However, its planar nature limits the lens functionality to 2D structures. Soft lithography with elastomeric stamps is an alternative to transfer functional components onto soft lens surfaces^593^ (e.g., for fabricating microfluidic components and sensor integration for real-time monitoring).^595^ For instance, Ku et al. integrated immunosensors into contact lenses with PDMS microfluidic channels to detect cortisol levels in tear fluid.^596^ Direct printing and inkjet printing are also used in SCL manufacturing.^597^ Kim et al. introduced a high-resolution 3D printing of LM to form stretchable free-standing 3D patterns of interconnects essential to electrically connect individual device components for their integration.^238^ Park et al. utilized inkjet printing for fabricating wirelessly chargeable 3D solid-state supercapacitors that serve as a power source for SCLs.^598^ The flexibility and precision offered by these methods enable the creation of custom molds and components on different substrates including flexible and stretchable materials. Electrospinning methods to produce ultrafine fibers from nanoparticles, nanorods, nanowires, nanotubes, and nanosheets in a polymer solution or melt can also be used to fabricate electrodes on the soft lens platform.^599^ Metal-based nanowires, nanofibers, and hybrid structures, such as silver nanowires (AgNWs) and silver nanofibers (AgNFs), have been researched for their enhanced electrical, mechanical, and optical properties in SCLs.

Various applications of SCLs are described in Section 3.3 when the detection and monitoring of biomarkers in tears were discussed. SCLs have also been utilized in other health-monitoring approaches, as depicted in Figure 18. They encompass six main aspects: vision repair, detection of biomarkers in tears, determination of physiological signals, eye movement tracking, power management of smart contact lenses, and neuroprosthetic contact lenses.

**Figure 18:**
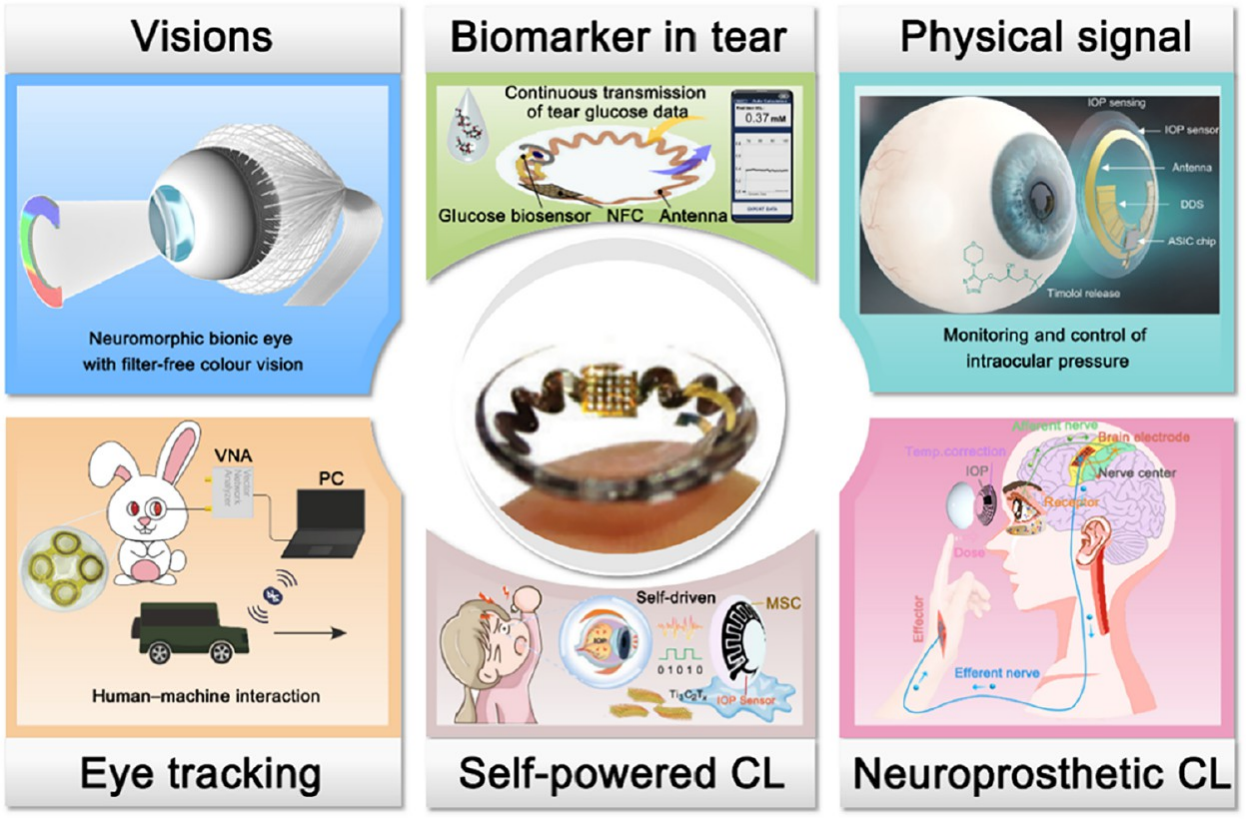
Overview of the smart contact lens for ophthalmic health monitoring. The digital image of the wireless SCL (in the center). Reproduced/adapted with permission from Song et al.^594^ © 2022 John Wiley & Sons. Visions, a neuromorphic bionic eye with filter-free color vision. Reproduced/adapted with permission from Long et al.^602^ © 2023 Springer Nature. Biomarker in tear. Reproduced/adapted with permission from Park et al.^236^ © 2024 Springer Nature. Physical signal. Reproduced/adapted with permission from Kim et al.^242^ © 2022 Springer Nature. Eye tracking. Reproduced/adapted with permission from Zhu et al.^603^ © 2024 Springer Nature. Self-powered CL. Reproduced with permission from Liu et al.^604^ © 2024 Royal Society of Chemistry. Neuroprosthetic CL. Reproduced/adapted with permission from Liu et al.^605^ © 2024 Springer Nature.

Human vision has basic features of a wide field of view, high resolution, and processing ability of visual information.^600^ Image sensors constructed by a flexible photodetector array with the capability of photon detection have promoted imaging technology, showing wide applications in the restoration of human visual function, medicine, optical communication, and the visual system of biorobotics. The photodetector can even achieve enhanced vision, like night vision, by adjusting the response spectral range from UV (ultraviolet, 10–300 nm), Vis (visible, 400–700 nm), IR (infrared, 700 nm–1 mm), to THz (Terahertz, 0.1 to 1 mm). The difficulty lies in the transformation of the planar photodetector to the hemispherical substrate with a diagonal visual field >100°, high resolution, in-device preprocessing signals, and optical adaptivity. Many attempts to find solutions have been made, as in Fan’s research,^601^ where a hemispherical electrochemical eye made with perovskite nanowires (NWs) served as an image sensor to mimic the photoreceptors on a human retina. A neuromorphic spherical bionic eye was endowed with color vision, in-device processing, and optical adaptivity.^602^ Figure 18 (“Vision”) shows the magnified structures of the neuromorphic bionic eye, which includes a convex lens, electronic iris, artificial crystalline lens, artificial retina based on perovskite NWs self-driven image sensor array with color imaging ability, and artificial optical nerves.

The potential of eye-machine/computer interaction via SCLs requires efficient and wireless communication. Mao et al.^603^ proposed a frequency encoding strategy for *in situ* eye tracing and wireless eye-machine interaction. Four passive copper radio frequency (RF) tags with different working frequency coils were embedded into biocompatible contact lenses. The eye movement information was encoded in RF signals for detecting the movement and closure of the eye. Then, a portable sweeping-frequency reader was fixed on the glasses and opposite to the user’s eyeball, collecting the tags’ signal without embedding chips and batteries. Since the RF signals depend on the eye movements or positions, a high angular accuracy of eye tracking was obtained. By detecting and analyzing these frequency changes, the system (or machine) can determine the direction and degree of eye movement in real time, which provides multiple eye-machine interaction modes.

Energy storage devices can be integrated into SCLs for driving the sensing units employed in health monitoring.^606^ For instance, Pourshaban et al.^607^ reported an eye-tear activated Mg-Air battery driven by eye blinking motion. It contains 4 parts, which were the substrate, an Mg anode, a Pt cathode, and an eye-tear fluid as the electrolyte. When the eyes periodically blinked, the battery was activated to deliver a power density of 1.3 mW cm^–2^ at a load of 740 Ω. However, the Mg-Air battery had low safety and poor stability. To meet the demands of wearable SCLs, supercapacitors are being considered owing to their high-power density, security, and stability. Park’s group^598^ developed rechargeable solid-state supercapacitors directly printed on soft substrates. When connected to an antenna, rectifier, and light-emitting diode, they provided continuous power for SCLs. An eye-wearable Ti_3_C_2_T_*x*_ MXene-based supercapacitor was used for powering a sensor to measure the intraocular pressure, as shown in Figure 18 (“Self-powered CL”).^604^ The supercapacitor delivers a high energy density of 10 mW h cm^–2^, which ensures the stable work of the integrated sensor. For more information on self-powered devices, see Section 10.

Neuroprosthetic devices that rebuild the connection between the nervous system and organs are promising for disease monitoring and health management. For instance, a neuroprosthetic contact lens (NCL) was used for POC monitoring and feedback of intraocular pressure, as shown schematically in Figure 18 (“Neuroprosthetic CL”).^605^ This NCL makes up for the lack of feedback on the abnormal intraocular pressure of the SCL and establishes the relationship between this abnormal pressure and the somatosensory cortex. The sensorimotor system consists of a pressure sensor, a temperature sensor, and a wireless transmission/display App. The NCL exhibited a high sensitivity of 12.52 mV mm Hg^–1^ and excellent stability. *In vivo* experiments in rats proved that NCL can convert temperature-corrected changes in intraocular pressure into a pulse signal, which is transmitted to the somatosensory cortex through electric current, triggering graded motor cortex feedback. The corresponding signals generated in the motor cortex further activate the leg twitch responses, finally forming a closed loop of intraocular pressure signal generation-nerve-perception-motor activities. These NCL devices represent a solution for the early prevention and precision diagnosis/treatment of ophthalmic diseases.

Regarding the determination of biochemical biomarkers, Song et al. developed smart contact lenses for the real-time quantification of cholesterol by using electrochemical biosensors.^608^ The device is intended to monitor hyperlipidemia patients and requires only a smartphone to be operated, not obstructing vision and presenting good wearability. The electrodes were built through the combination of metal thin-film deposition and screen printing, and the working electrode was modified with Prussian blue and the cholesterol oxidase enzyme. The bioreceptor catalyzes the oxidation of cholesterol into H_2_O_2_, which is in turn detected electrochemically. Stretchable antennas and integrated circuits for wireless communication were also integrated into the smart lenses, making it straightforward to use. *In vivo* experiments displayed a good correlation between cholesterol levels in tears and blood, showing their potential applicability in clinical settings.

### 5.4 Mouthguards

Since the 1960s, when Graf and Mühlemann developed a removable partial denture to monitor salivary fluoride ions, interest in intraoral wearable sensors has grown, driven by the potential of saliva as a noninvasive diagnostic medium.^609,610^ The emphasis on saliva arises from its capacity to transport molecules directly from the bloodstream via transcellular or paracellular pathways, effectively serving as a “mirror” of the body’s physiological state.^609,611,612^ These early devices integrated oral sensors, miniature transmitters, and onboard power sources into removable dentures, enabling *in vivo* measurements while the denture was worn.^610^ Despite the novelty of this approach, initial prototypes did not undergo comprehensive safety assessments, which constrained their development. Recent advances in wearable technology, including miniaturization, mechanical flexibility, and wireless communication, have enabled more sophisticated, noninvasive access to salivary biomarkers and strengthened the technological foundation for physiological monitoring, notably via mouthguard-based sensors.

A mouthguard wearable sensor is typically fabricated from medical-grade polymers such as polyethylene terephthalate glycol (PETG), ethylene-vinyl acetate (EVA), and natural rubber.^613^ To obtain a custom fit, the user’s oral anatomy is captured using a dental cast,^614^ and the support is formed by pressure forming, vacuum forming, or 3D printing.^613^ Commonly used in clinical settings and as protective gear against sports-related injuries, these devices seat securely over the teeth, are straightforward to wear and replace, and maintain continuous contact with relevant oral biofluids—features that are advantageous for real-time monitoring.^615^ The first mouthguard biosensor was reported in 2014 by Joseph Wang’s group, who screen-printed a three-electrode system onto a flexible, commercial polyethylene terephthalate (PET) mouthguard using Prussian blue/graphite inks for the working and auxiliary electrodes and Ag/AgCl ink for the reference (Figure 19). Lactate oxidase was immobilized on the working electrode by electropolymerization within poly­(o-phenylenediamine), which both entrapped the enzyme and mitigated interference from the complex salivary matrix.^612^ Although the sensor provided reliable amperometric detection of lactate in saliva samples, it was not tested *in vivo*. The authors emphasized key limitations, calling for further miniaturization, integration of wireless circuitry, and rigorous safety and biocompatibility assessments prior to practical use.^615^

**Figure 19:**
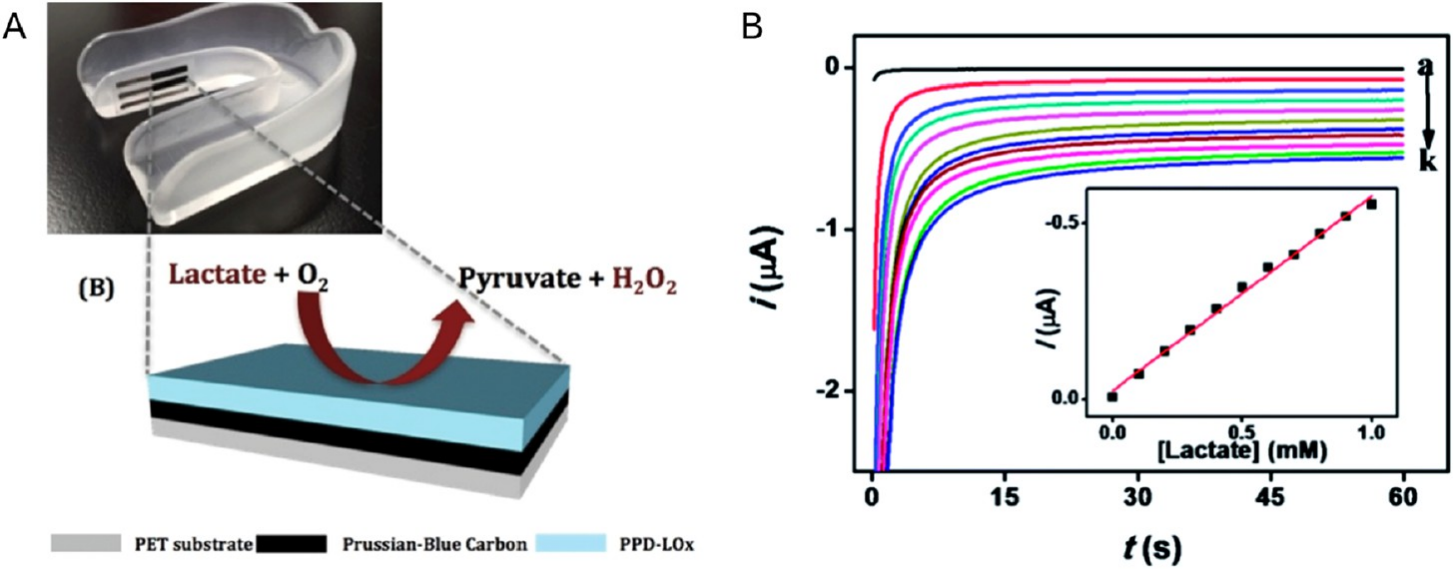
Electrochemical biosensor embedded in a mouthguard. (A) Picture of the modified mouthguard, containing an integrated electrode, as well as schematics showing the layers of the working electrode and the enzymatic detection principle. (B) Chronoamperograms obtained in different lactate concentrations. Reproduced with permission from Kim et al.^615^ © 2014 Royal Society of Chemistry.

Building on these advances, Arakawa and co-workers addressed several of these limitations.^224^ The mouthguard support was thermoformed from PETG by suction molding after being heat-softened. Platinum and silver electrodes, materials considered to be biocompatible and safe for human use, were deposited on PETG by parallel-plate sputtering and insulated with PDMS. A cellulose acetate membrane was applied as an interference-rejection layer, combining electrostatic repulsion and size-exclusion effects. GOx was immobilized on the Pt electrode using poly­(MPC-*co*-EHMA-*co*-MBP) (PMEHB), chosen for its biocompatibility and mechanical strength; a PMEHB overcoat further limited enzyme leakage and suppressed contaminants. A BLE wireless module and a battery were affixed near the sensing region by heat-welding with a heat gun. The mouthguard was then fitted to a fasting subject, and continuous signal changes were recorded wirelessly.

In addition to monitoring biomolecules in saliva for health purposes, mouthguard devices are also being used as physical sensors. Ichikawa and colleagues^616^ developed a mouthguard wearable sensor to measure salivary turbidity, an indicator of oral hygiene. Their optical sensor, integrated with an LED, phototransistor, wireless module, and coin cells, was sealed inside a double-layered PETG mouthguard. The optical path between the LED and phototransistor measured the turbidity in the sensing area. In *in vivo* experiments with diluted milk, the mouthguard sensor successfully quantified turbidity in the range of 100–1000 FTU, demonstrating high linearity and repeatability, sufficient for standard salivary turbidity measurements. The same group^617^ introduced a battery-free bite sensor for diagnosing intraoral and extraoral conditions. The device integrates an ultrathin sensor sheet, 82 μm thick, comprising a PET dielectric, an electret, and electrodes. It functions as an energy harvester, generating both power and signal from bite-induced deformation, thereby eliminating the need for a battery. The mouthguard is vacuum-formed on a dental model, and the sensor is embedded in the occlusal surface of a double-layered shield. *In vitro* tests demonstrated a dynamic range up to 2.5 MPa, corresponding to approximately 1 kN of bite force across the full dentition.

Mouthguard-based platforms have evolved from proof-of-concept biochemical sensors to multifunctional devices capable of both chemical and physical monitoring. Customizable, biocompatible materials and improved integration of sensing, sealing, and wireless modules now enable continuous data acquisition in the oral environment. Challenges in developing wearable mouthguard sensors include biofouling caused by saliva’s high protein content. Furthermore, biomarker concentrations are often far lower than in blood, and correlations between salivary and serum levels are not yet well-defined. Additional factors, including background noise, matrix interference, viscosity, salivary flow rate, and recent food intake, can compromise accuracy and stability.^609,612^ Important requirements include sensor integration, wireless modules, and power sources within the limited form factor of a mouthguard to avoid altering natural occlusion. In this context, one needs to consider safety concerns in using batteries owing to the risk of accidental ingestion.^618^

### 5.5 Wristband/Watches

Technological advances have transformed wrist-worn devices from simple timekeeping jewelry into networked computers. Modern devices now incorporate miniaturized batteries and processors, a variety of applications, and wireless communication modules, enabling continuous data acquisition and connectivity. Also, there is an expanding set of sensors for health-monitoring features. Commonly termed smartwatches, wristbands, or fitness trackers, these devices are currently among the most used wearable systems for health monitoring.^5,7,619^ The first generation of wrist-worn technologies was marked by the advent of Android Wear and Apple Watch in the early 2010s.^620^ These initial smartwatches were equipped mainly with motion sensors such as accelerometers, gyroscopes, and magnetometers, which detect changes in linear acceleration and rotational velocity.^5,621^ In practice, they were used predominantly for fitness monitoring, including step counting, estimation of energy expenditure, assessment of exercise intensity, and inference of sleep patterns.^622^ To this day, motion sensors remain among the most widely implemented technologies in wrist-worn wearable devices.

Over time, health-monitoring smartwatches have evolved from simple activity trackers into complex multisensor devices. Current models can monitor blood pressure and heart rate, sleep–wake cycles, skin temperature, and other vital signals. A central technology in this context is photoplethysmography (PPG), a fiber-free optical sensing method that probes physiological parameters through light–tissue interactions.^6^ On the wrist, PPG is used to track blood volume changes in the microvasculature, which are converted into electrical signals; the interval between successive pulsations (interbeat interval) can then be processed by algorithms for atrial fibrillation detection.^623^ Devices such as the Apple Watch combine PPG with single-lead electrocardiography. Beyond cardiac monitoring, PPG signals are also used to derive respiratory waveforms and to distinguish sleep–wake states, supporting the assessment of sleep quality and the detection of conditions such as sleep apnea.^624^ Skin temperature is another relevant physiological parameter, as it is associated with stress levels^625^ and reflects systemic status, including the presence of fever or infection. In smartwatches, this vital sign is typically monitored using thermistors, thermoelectric-based sensors, or optical approaches.^626^

In the literature, the terms *smartwatch* and *fitness tracker* usually refer to commercial wrist-worn devices designed to monitor physical health parameters. In contrast, *wristband* is often used for custom or prototype wrist-worn sensors developed in research settings that can measure both physical and biochemical markers. For biochemical monitoring, sweat is the most common biological matrix,^202,627–629^ owing to its noninvasive accessibility and the possibility of continuous sampling. Wristbands themselves can be constructed from diverse structural (flexible or rigid) and sensing materials, using both standard microfabrication and low-cost solution processing routes. Emaminejad and co-workers,^202^ for example, reported a flexible wristband for sweat analysis of Na^+^, Cl^–^, and glucose. In their design, the electronics are integrated on a flexible printed circuit board within a plastic housing, while the sensing and iontophoresis electrodes are microfabricated on an ultrathin PET film by photolithography and thermal evaporation. A key feature of this platform is the fully integrated, on-wrist sweat stimulation and analysis: iontophoretic electrodes contact the skin through a drug-loaded agarose hydrogel that locally induces sweating, and a thin rayon pad wicks sweat directly to the sensing membranes, enabling autonomous, exercise-independent, real-time measurements.

Cai and co-workers^628^ pursued a complementary strategy, implementing an integrated sweat-sensing wristwatch built on a rigid FR-4 commercial printed circuit board, with all electronics embedded in a 3D-printed resin case and held against the skin by an elastic silicone wristband. In this architecture, the ion sensors are fabricated directly on the FR-4: during the standard RPCB manufacturing process, the sensing electrodes are defined as metallic pads on the board by photolithography. Sweat handling is achieved by a laser-cut PET microfluidic layer bonded on top of the sensor array with medical adhesive; this PET film defines inlet and outlet ports and a network of shallow microchannels. During use, sweat generated naturally during exercise is pressed into the inlet by contact between the underside of the watch and the skin. From there, it is wicked through the PET microchannels by capillary forces, forming a continuous thin film that flows across the membrane-coated electrodes and enables a stable, on-wrist, real-time potentiometric readout of electrolyte concentrations. In a different approach, Ma and collaborators^627^ built a sweat-sensing wristband directly in a single piece of textile instead of on a rigid PCB (Figure 20). A commercial PET cloth serves simultaneously as a mechanical support and electronic substrate. To construct the sensor array, the textile is first converted into a conductive Cu-patterned fabric via polymer-assisted metal deposition (PAMD) and then patterned by double-sided photolithography and wet etching to define all electrodes and interconnects monolithically on the cloth. In this case, sweat is collected by exercise-induced perspiration using an epidermal microfluidic module: a PDMS channel layer molded from a 3D-printed template is laminated onto double-sided medical tape patterned with multiple sweat inlets and a reservoir over the textile electrodes so that sweat from a larger skin area is continuously drawn into the channels and across the sensing cavity by hydraulic/osmotic pressure and capillary flow. The textile-first architecture of this platform preserves air and moisture permeability, offering long-term wearing comfort and breathability that conventional flexible electronics on plastics cannot match.

**Figure 20:**
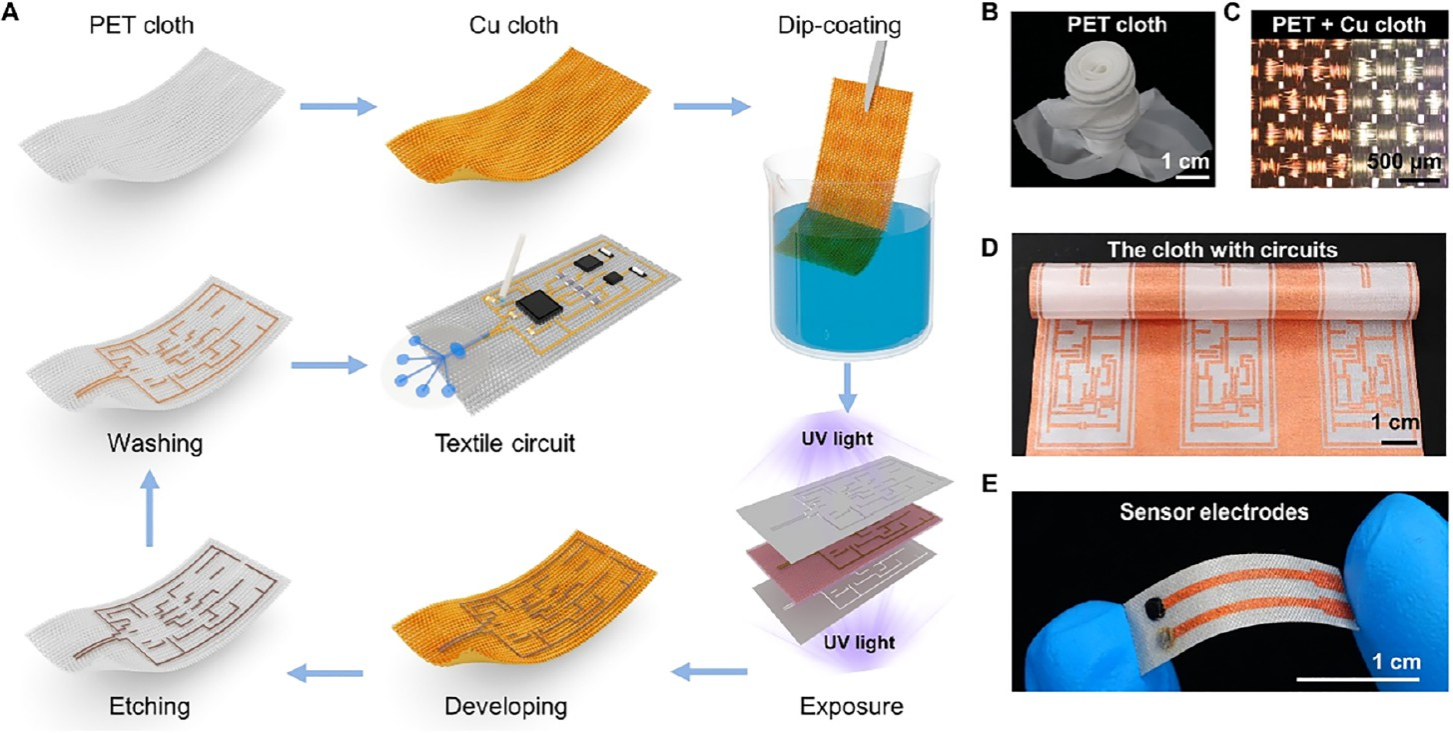
Sweat-sensing wristband developed by Ma et al. (A) Steps involved in the patterning of the device using double-sided photolithography; (B) flexible PET cloth used in the wristband; (C) sharp interface between PET and Cu cloth; (D) a picture of the cloth containing the patterned circuits; (E) electrodes in detail. Reproduced from Ma et al.^627^ Available under a CC-BY 4.0 license. © 2023 American Association for the Advancement of Science.

Taken together, these examples illustrate how wrist-worn devices for health monitoring have evolved from motion-centric smartwatches to sophisticated platforms capable of biochemical sensing, built on flexible films, rigid PCBs or textiles. At the same time, they highlight that the main construction-related limitations of wrist-worn systems still lie in the difficulty of monolithically integrating batteries and electronics with robust skin–sensor interfaces and reliable sweat collection/transport, while maintaining a form factor that is comfortable, manufacturable at scale and stable over time.

### 5.6 Textiles

Electronic textiles (e-textiles) represent a natural evolution of wearable technology, merging conventional fabrics with sensors or electronics. These smart textiles have social acceptability across sectors such as healthcare, fashion, sports, and personal defense by enabling real-time monitoring, adaptive functionality, and interactive communication between users and their environments.^630–635^ The intimate contact with large body areas makes them ideal for hosting distributed biosensors at different body locations to track the physiological, biochemical, and metabolic markers of the wearer more effectively than many conventional sensing methods. The all-day duration of wearability further makes e-textiles suitable for continuous monitoring of biomarkers for chronic conditions, rehabilitation, personal health, and lifestyle management.^636–638^ The widespread adoption of e-textiles is hindered by several challenges, especially in fabrication.^639–642^ For e-textiles to be adopted on a large scale, they must balance electronic performance with wearability, ensuring durability, washability, and long-term reliability.^643^ Scalable manufacturing must maintain high performance without excessive production complexity or cost. Because powering these textiles remains a significant hurdle, researchers are exploring solutions such as harvesting energy from body movement or fluid or using radio frequency waves to power wirelessly.^644,645^ This section explores the key fabrication techniques used in e-textile manufacturing, their associated challenges, and emerging wireless solutions that could pave the way for widespread adoption.

#### 5.6.1 Fabrication of e-Textiles

A typical e-textile requires a supporting matrix to maintain its everyday-wear feature, functional components to promote the capacity of sensing, computing, and communication, and a protection layer to endorse durability under daily use and washing (Figure 21A).^646–650^ The supporting matrix is usually the textile itself, which is made from yarns/threads by weaving, knitting, crocheting or bonding. The base yarns or threads are usually made from natural fibers like cotton, wool, and silk, which can offer comfort and breathability, or synthetic fibers such as polyester and nylon, which provide durability and moisture-wicking properties (Figure 21B).^641,651,652^ These base materials are not inherently conductive, but can be transformed into conductive materials for sensing through the incorporation of functional components. These functional components can be external attachments of classic electronic devices, such as LEDs, integrated circuits, and traditional batteries, that are attached to the surface of a traditional textile (Figure 21C).^653^ However, this approach tends to be less desirable as it can compromise the textile-like properties such as breathability, flexibility, and lightness. Thus, the primary thrust in e-textile research is to integrate or embed the electronic function into textiles, maintaining the desired characteristics while offering advanced functionalities. One strategy is to directly coat, electroplate, or print functional elements on the surface of textile using various inks, bypassing rigid electronic devices (Figure 21C).^654–659^ The typical function imparted to textiles is electrical conductivity for applications such as physical and electrochemical sensing, wireless power transfer, or communication. The conductive inks are typically based on materials including metallic nanoparticles or nanowires, MXenes, carbon-based materials (carbon nanotubes, graphene), and conjugated polymers (PEDOT:PSS, PPy, PANI).^4,660^ Metallic ink provides the best conductivity, and silver-based inks, including nanoparticles, nanowires, and flakes, are most used because of the good balance between cost and performance, offering better oxidation resistance than copper and significantly lower cost than gold.^656,661^ Carbon-based and conjugated polymer-based inks provide higher electrochemical activity for electrochemical or physiological sensing and larger resistance for physical sensing (strain, pressure, temperature) compared to metal-based ones.^662,663^ Other functionalities, such as energy harvesting (triboelectric generators, piezoelectric and thermoelectric generators), electroluminescence, and biosensing, are often implemented at the single-fiber level and then integrated into textiles,^640,651,664–668^ which will be introduced in the next section.^654–660^

**Figure 21:**
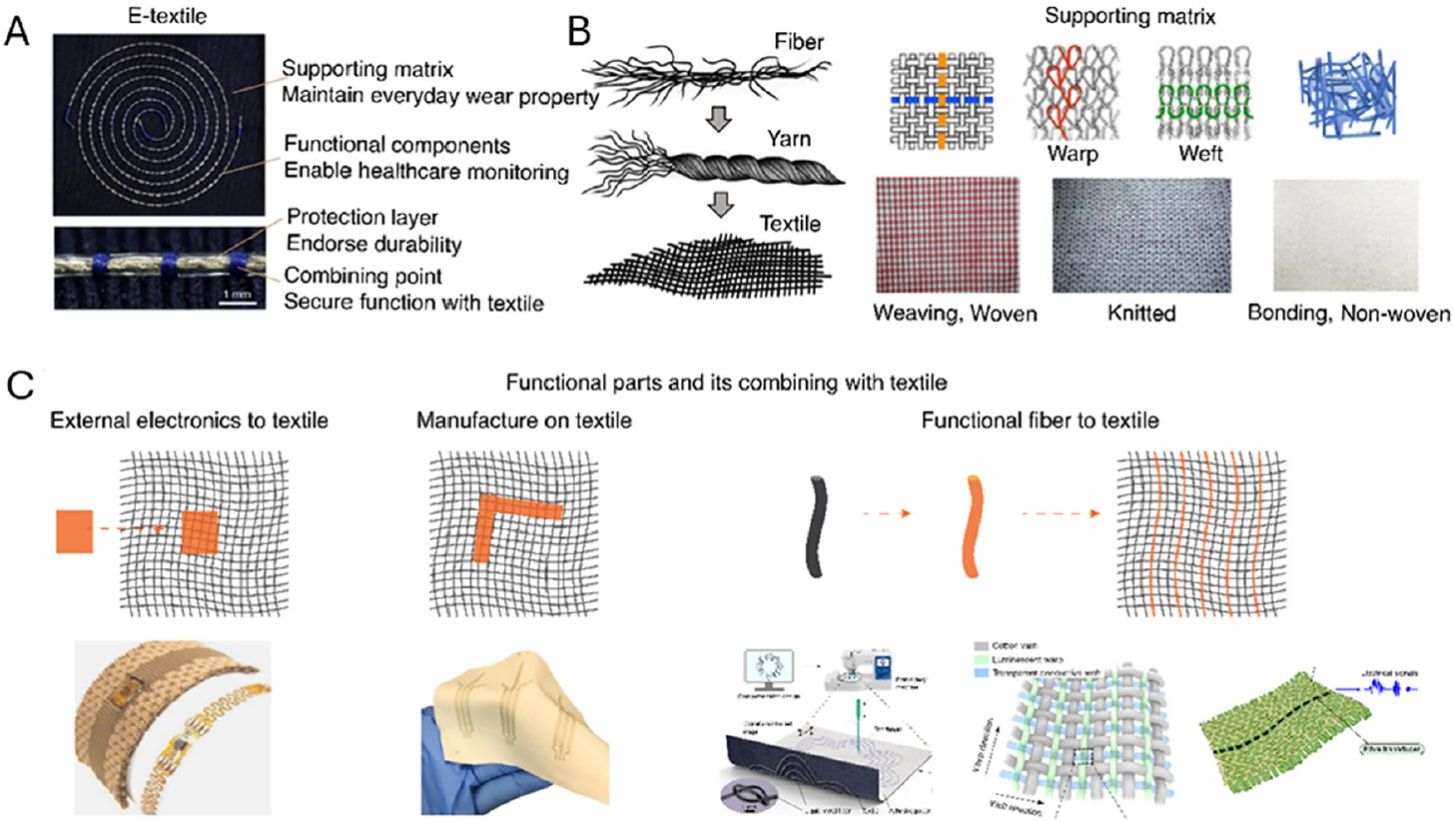
E-textile fabrication architecture. (A) Overview of e-textile structure and definition. Reproduced from Lin et al.^669^ Available under a CC BY 4.0 license. © 2020 Springer Nature. (B) Supporting matrix of e-textile formation. Reproduced from Latko-Duralek et al.^651^ Available under a CC BY 4.0 license. © 2023 MDPI and Wang et al.^652^ © 2019 John Wiley & Sons. (C) Functional parts and their integration into textile. It typically follows three main approaches. First, surface attachment. Attaching premanufactured electronic components directly onto textile surface. Reproduced from Wicaksono et al.^653^ Available under a CC BY 4.0 license. © 2020 Springer Nature. Second, in situ manufacturing. Directly fabricating functional elements onto textile surface using coating or printing technique. Reproduced with permission from La et al.^659^ © 2018 John Wiley & Sons. Third, fiber integration. Incorporating functional fibers, such as conductive fibers or fiber-based devices, into textile through methods like digital embroidery, conventional textile weaving, knitting, or bonding. Reproduced with permission from Shi et al.^670^ © 2021 Springer Nature, Yan et al.^671^ © 2022 Springer Nature, and Lin et al.© 2022 Springer Nature^672^ Lin et al. is available under a CC BY 4.0 license.

Most methods for coating, electroplating, and printing used for flexible polymer substrates can be adapted for textiles. However, an extra layer for protection and insulating additives for modifying the ink is required to improve adhesion, mechanical robustness, and durability. This inevitably alters the textile nature with reduced breathability and increased stiffness. Additionally, the insulator additives lower the electrical conductivity to around 10% of pure metal, and the conductive path degrades over time due to washing and daily wear. Despite these limitations, coating and printing methods are advantageous in fabrication speed and simplicity, especially when fine pattern resolution is not required (less than 100 μm). They eliminate the need for complex photolithography, and the setup can be deployed rapidly. Coating is mostly done with techniques such as dip-coating, spray coating, electroless plating, electroplating, and vapor deposition (Figure 22A).^673^ Dip-coating, in particular, is simple and scalable, involving immersion of fibers into functional solutions followed by solvent evaporation and thermal curing. For example, conductive cotton yarns coated with inks containing conductive carbon paste (CCP), PEDOT:PSS, and sodium alginate (cross-linked with calcium chloride) achieved a conductivity of 19.46 ± 0.240 S·cm^–1^, with minimal loss after washing.^673^ Similarly, MXene-coated Kevlar yarns reached a low linear resistance of 5.3 ± 0.7 Ω·cm^–1^ using only 0.12 mg·cm^–1^ of MXene.^674^ Electroluminescent fibers, promising for textile displays and wireless interaction, have also been fabricated by coating ZnS phosphors on silver-plated yarns and encapsulating them with silicone, maintaining a luminance of 122 cd·m^–2^ after 10,000 folding cycles.^670^

**Figure 22:**
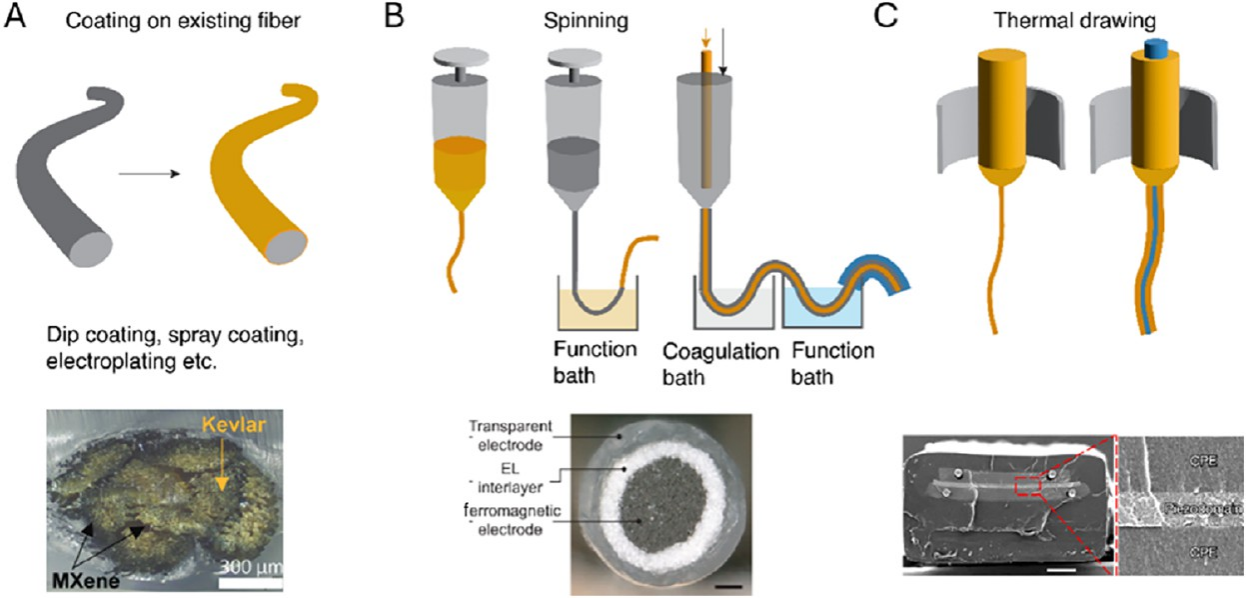
Fabrication of functional fibers and yarns in E-textile. (A) Coating-based methods by introducing functional materials on the surface of fiber or yarn. Reproduced with permission from Bi et al.^674^ © 2023 John Wiley & Sons. (B) Spinning-based methods by extruding liquid or molten polymer or polymer composite via a small hole to form a thin filament that solidifies into fiber mostly by coagulation bath, and can be further functionalized by functional materials bath coating. Reproduced from Fu et al.^682^ Available under a CC BY-NC license. © 2024 Springer Nature. (C) Thermal-drawing-based method by heating a prior prepared macroscopic preform to a viscous state and drawn into kilometer-long fiber. Reproduced with permission from Yan et al.^671^ © 2022 Springer Nature.

Electroplating enables highly conductive and precisely controlled metal or polymer coatings (e.g., Cu, Ag, Ni, Au, PEDOT:PSS) where high conductivity and flexibility are required, which cannot be provided by dip-coating or spray coating.^675^ The thickness of such a coating is usually less than 1 μm to lower the cost and minimize added stiffness. This method requires conductive surfaces or pretreatment, limiting direct use on insulating textiles. Electroless plating chemically produces uniform metal coatings on insulating fibers without external current, but has slower deposition rates and stringent control of plating bath chemistry.^647^ Spinning is an interesting alternative, including wet spinning, melt spinning, electrospinning, and microfluidic spinning (Figure 22B).^676,677^ It integrates functional materials into polymer fibers in a single step at scale, enabling precise control over uniformity, conductivity, and mechanical properties. Wet spinning is the most adopted because of its low processing temperatures and simple process, which is preferable for thermally sensitive or nonthermoplastic functional materials.^678–680^ Fibers produced with wet spinning include those containing ATO@TiO_2_ nanoparticles, which achieved a stable electrical conductivity of 2.5 S m^–1^ under strains of 50 and 100% for 1000 cycles.^681^ A self-healing electroluminescent fiber has also been developed using wet spinning, in which ZnS and Ni particles are dispersed within a high-k PVDF-HFP matrix to form a core–shell structure.^682^ A hydrogel layer served as the transparent conductive outer sheath. This fiber recovered over 98.6% of its initial luminance after complete severing and continued to operate reliably for more than 10 months.

While coating and spinning methods possess various advantages, they often require adhesion promoters or suffer from an overall conductivity reduction by insulating additives and limit architectural complexity. Thermal drawing presents an alternative for fabricating multifunctional fibers with integrated internal structures (Figure 22C). This process involves designing a macroscopic preform composed of polymers, metals, and semiconductors arranged in a precise geometry, which is then heated and drawn into fine microscale fibers. Thermal drawing preserves the geometric arrangement of materials across scales, allowing for the integration of diverse functions within a single fiber. For example, acoustic sensing fibers have been created using this method by embedding piezoelectric poly­(vinylidene fluoride-*co*-trifluoroethylene) (P­(VDF-TrFE)) and BaTiO_3_ nanoparticles within an elastomeric cladding with internal copper electrodes.^671^ These fibers have demonstrated applications in assistive technologies, including voice detection, acoustic communication, and real-time heart sound monitoring. Thermal drawing has also been used to develop fibers for computing,^683^ neural interfaces,^684^ and energy storage.^685^ Its compatibility with continuous manufacturing and scalability makes it highly attractive for commercial e-textile applications.

An alternative strategy for e-textile fabrication is to integrate premanufactured functional fibers or fiber-based devices directly into textiles (Figure 21C).^667,686–690^ This fiber-based approach offers advantages over on-site fabrication, particularly in its compatibility with existing textile manufacturing processes and improved durability, as the functional fibers are preinsulated before integration. It also maintains the intrinsic porosity of textiles, which is critical for comfort and long-term wearability. By functionalizing individual fibers with distinct sensing materials prior to assembly, multiple sensing modalities can be achieved within a single textile platform. A solid metal wire covered by textile yarns using a braiding machine provides robust conductivity to make e-textile where high conductivity is required, but faces limitations in comfort. Thus, composites made from conductive materials with yarns or synthetic fibers have become the preferred choice. Metallic ink, MXene, carbon-based materials (CNTs, graphene), or conductive polymer (e.g., PEDOT:PSS, PPy, PANI, etc.) have been adopted to integrate into textile fibers.^4,660^ Unlike film-based coatings that often fail under repeated deformation, these fiber-integrated textiles are better suited to conform to complex body movements without compromising performance. This method combines wearability, robustness, and scalability, making it highly suitable for healthcare applications that demand reliable, unobtrusive operation during daily use.

One common approach is weaving or knitting functional fibers directly into the textile. It is compatible with industrial-scale textile manufacturing and allows for integration at the yarn level and across large textile areas, preserving the textile nature and user comfort. Electrochemical textiles capable of real-time health monitoring have been developed by weaving sensing fibers into textiles to detect analytes such as glucose, Na^+^, K^+^, Ca^2+^, and pH.^670,691^ Fiber integration can also be made with digital embroidery, where conductive or functional fibers are stitched onto existing textiles using programmable embroidery machines. One distinctive advantage of embroidery is the ability to place stitches in any direction—forward, backward, and sideways—allowing for flexible and complex designs. Silver-plated polyamide yarn encapsulated in transparent thermoplastic polyurethane tubing has been stitched onto regular textiles through this method to form wireless coil networks.^669,672^ It can sense physiological signals, including spinal cord posture, temperature, gaits, etc., across multiple body locations. While embroidery offers excellent design flexibility and preserves the porosity of unstitched areas, it may introduce local stiffness or thickness where dense patterns are applied.

#### 5.6.2 Wireless e-Textiles

E-textile platforms have been produced that are capable of wirelessly receiving power, detecting physiological signals, and transmitting data. Especially for radio frequency network systems, they can detect signals from different locations on the body within one wearable format, eliminating bulky batteries or rigid circuits for an unobtrusive form. These networks distribute sensing and communication tasks across multiple nodes, typically sharing a common reader or power source, which reduces the need for each sensor to be fully self-contained. By attaching prepatterned conductive fabrics (such as copper/nickel-coated polyester) to textiles via adhesives, Tian et al. introduced a metamaterial network through radio surface plasmon propagation along the patterned fabrics (Figure 23A).^692^ It enables wireless signals in radio-wave propagation confined around the body rather than radiation to the outside. The power transmission efficiency was enhanced by 3 orders of magnitude compared to that of a traditional radiative network. Additionally, signal confinement within 10 cm of the body enhances the communication security by limiting the risk of external interception. Using computer-controlled embroidery of conductive threads (silver-plated polyamide yarn encapsulated in transparent thermoplastic polyurethane) onto regular textiles, Lin et al. developed near-field-enabled clothing with battery-free sensors to monitor physiological signals continuously (Figure 23B).^669^ Multiple electromagnetically responsive thread patterns were integrated to form a body network with one reader hub, extending the short working distance in a few centimeters of near-field technology to meter-scale networks. Thus, wireless signal collection can be done from different sensors located at different locations on the body.

**Figure 23:**
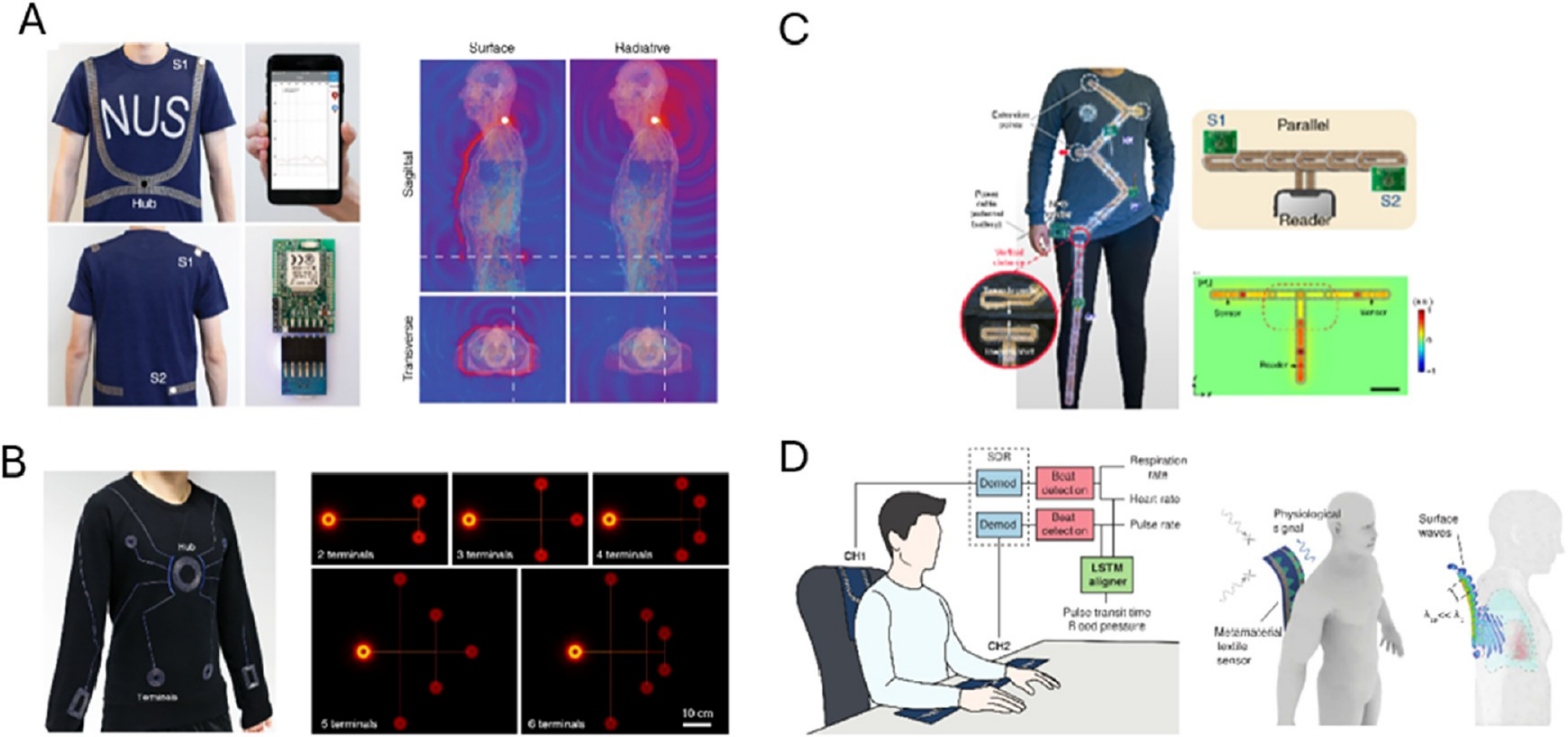
System-level wireless e-textile design and fabrication. (A) Metamaterial textile network. Reproduced with permission from Tian et al.^692^ © 2019 Springer Nature. (B) Digital embroidery near-field clothing network. Reproduced from Lin et al.^669^ Available under a CC BY 4.0 license. © 2020 Springer Nature. (C) Reconfigurable NFC textile network. Reproduced with permission from Hajiaghajani et al.^693^ © 2021 Springer Nature. (D) Contactless e-textile. Reproduced from Nguyen et al.^695^ Available under a CC BY-NC license. © 2024 American Association for the Advancement of Science.

Hajiaghajani et al. proposed a reconfigurable NFC textile network via magneto-inductive wave propagation along flexible arrays of discrete resonators constructed from laser-cut metal coils (Figure 23C).^693^ The networks allow battery-free sensors to be added or relocated (“drag-and-drop”) anywhere along the coil arrays. The authors demonstrated real-world use across multiple garments and during user motion, highlighting the practicality of this distributed sensing system approach. Another innovation comes from Takahashi et al., who developed a twin meander coil textile system for passive inductive telemetry (PIT).^694^ The large reader coil was fabricated using industrial knitting techniques, and interrogated spatially distributed sensor coils, each having a different resonant frequency. The meandered winding pattern alternates between clockwise and counterclockwise turns, which mitigates electromagnetic interference from the body. This enables accurate and spatially flexible sensing without requiring direct coupling or complex routing. Contactless sensing eliminates the need for physical contact between the textile and the skin, thus enhancing user comfort and long-term durability. Nguyen et al. developed a contactless metamaterial textile that was integrated into the furniture surface for ambient physiological monitoring.^695^ The textile comprised multilayer structures with patterned conductive top layers, intermediate nonconductive fabric layers, and conductive bottom isolation layers. The conductive layers were specifically patterned via laser cutting to support spoof surface plasmon (SSP) modes. Using the close-range interaction between SSP surface waves and the human body, it can monitor multisite physiological signals, including heartbeat, respiration, and blood pressure.

### 5.7 Facemasks

Respiratory monitoring masks have been used for personalized medicine and early disease diagnosis,^696,697^ with the integration of sensors and data processing technologies. These devices can be fabricated using several methods, including the direct integration of sensors into the facial region of the mask to ensure direct contact with the breath airflow. By embedding sensors such as airflow vibration sensors,^698^ pressure sensors,^699^ humidity sensors,^700^ and temperature sensors^701^ within the mask, researchers can collect multiple physical parameters of respiratory airflow, including the respiratory rate, airflow velocity, humidity, and temperature. A key challenge is reaching large-scale fabrication of facemasks incorporating wearable sensors. A possible solution is to employ standard computerized embroidery where conductive threads are produced via a scalable roll-to-roll process and optimized to maintain performance under mechanical stress, washing, and long-term use.^702^ Using these threads, sensors could be directly integrated into fabrics as demonstrated with a facemask for respiration monitoring by measuring moisture-induced conductivity changes in the textile. Indeed, smart facemasks may evolve into platforms for personalized, real-time health monitoring if sensors and electronic devices can be integrated. For instance, a facemask embedded with a near-field electrochemical sensor was used for continuous respiratory assessment, which contained a lightweight, flexible, and battery-free sensor chip that could detect breathing patterns through humidity-induced impedance changes.^703^

Once processed by algorithms, the data collected with facemasks as indicated above can extract valuable respiratory health information, providing support for clinical diagnosis and daily health management.^704,705^ In addition to monitoring physical parameters, the chemical composition of exhaled breath also serves as a health indicator (see Section 3.6). The incorporation of electrochemical sensors allows the mask to detect specific chemical components in breath gases, such as oxygen, carbon dioxide, and nitric oxide.^705–708^ These sensors operate by detecting redox reactions of gas molecules on electrode surfaces or changes in electrolyte pH, enabling real-time reflection of the chemical composition and concentration of breath gases. Monitoring masks have also benefited from microfluidics to detect a broader range of biomarkers.^32,709^ For instance, through microfluidics, researchers can extract nonvolatile substances such as inflammatory factors, proteins, alcohol, and lactate, providing new sample sources for studying metabolic conditions and other respiratory system diseases.^32^

By integrating ML algorithms, smart respiratory monitoring masks can extract valuable health insights from vast amounts of respiratory data, helping users identify potential health issues in a timely manner. For example, algorithms such as support vector machines (SVMs) and convolutional neural networks (CNNs) have been widely used for the classification and pattern recognition of breath data, laying the foundation for personalized and precision medicine.^318^ In a recent example, ML was used to determine glucose concentration through breath analysis with a facemask incorporating an organic electrochemical transistor (OECT).^710^

### 5.8 Diapers

Sensors embedded within diapers enable real-time analysis of urine biomarkers for diabetes management,^298^ neonatal jaundice monitoring,^711^ and long-term care.^712–715^ Additional discussion on urine biomarkers can be found in Section 3. The fabrication process consists of integrating flexible and functional materials into the diaper structure.^297,716^ Biosensors are typically embedded between the diversion and absorption layers, allowing direct contact with urine. Substrates such as PET or cellulose-based materials are chosen for their flexibility and compatibility with screen-printing technologies.^298,306,717^ Conductive inks, including silver and carbon composites, are printed to form circuitry and electrodes, while functionalized layers sensitive to specific biomolecules, such as GOx for glucose detection or bilirubin oxidase for bilirubin analysis, provide precise biochemical detection. Self-powered diaper sensors have been made with biofuel cells that harness enzymatic reactions to generate electricity from urine glucose (see Section 10.3).^297,718,719^ Paper-based glucose biofuel cells were fabricated by printing MgO-templated porous carbon electrodes and graft-polymerizing active enzyme layers, ensuring both stability and high sensitivity (Figure 24).^297^ Data transmission modules, equipped with wireless communication technologies such as Bluetooth or Wi-Fi, transmit real-time data to smartphones or cloud platforms, facilitating remote monitoring (see Section 9). Structural integration is achieved through modified diaper layers that optimize fluid diversion and interaction with the biosensor, using hydrophilic polymers for efficient urine flow and absorption layers to maintain user comfort and hygiene.

**Figure 24:**
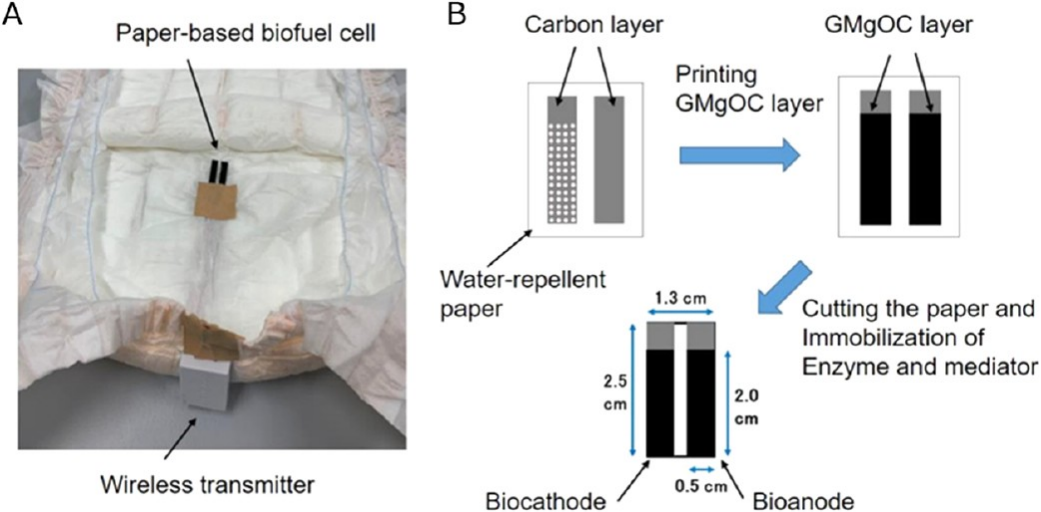
Paper-based diaper biofuel cell developed by Shitanda et al. (A) Picture of the device embedded in the diapers, containing the electrodes and the wireless data transmitter. (B) Preparation process of the device. Reproduced from Shitanda et al.^720^ © 2021 American Chemical Society.

Design principles for these sensors prioritize biocompatibility, ensuring that materials are safe for prolonged skin contact and hypoallergenic.^721^ Flexibility is essential to maintain comfort and functionality during movement, while miniaturization ensures that the sensors are lightweight and do not add bulk. Methods such as screen printing and roll-to-roll processing can be used to obtain durable diapers capable of withstanding a wet environment. These design considerations ensure that diaper sensors meet the requirements of diverse user populations from neonates to elderly patients.

## 6 Physical Sensors

Wearable physical sensors are used to monitor parameters such as heart rate, blood pressure, and body temperature and can be combined with chemical sensors to provide a comprehensive picture of the health of an individual. In the following sections, the recent advancements of the most relevant wearable physical sensors will be further explored.

### 6.1 Pressure and Strain Sensors

Pressure and strain sensors can be applied for monitoring multiple essential clinical parameters, including pulse and respiration, besides acting in fall detection, gait analysis, assessment of joint movements, and so on. The landscape of the literature on wearable sensors in Section 2 highlights the dominance of pressure and strain sensors due to the large number of publications (tens of thousands). An inspection of the clusters associated with these sensors indicated the relevance of advanced materials in their use, particularly nanomaterials and hydrogels. Because the topic contains such an extensive literature, we will not attempt a historical overview of material developments. Instead, we will present a few recent examples that we hope will illustrate these advancements.

The importance of hydrogen bonding and the incorporation of nanomaterials into hydrogels with tailored properties were highlighted by He et al.^722^ They developed a soft, conductive hydrogel composed of PEDOT:PSS, PAM, silk fibroin (SF), and graphene oxide (GO). Figure 25A depicts the possible structures of two hydrogels: PS (PAM/SF) and PSGP (PAM/SF/GO/PEDOT:PSS). The SF nanofibrils impart mechanical strength to the PS hydrogels, enabling them to function as elastic matrices. When GO sheets are incorporated into the PS matrix via hydrogen bonding and combined with PEDOT:PSS layers, the resulting PSGP hydrogels also exhibit conductivity. PSGP hydrogels were employed in strain and pressure sensors capable of monitoring physical signals from the human body, such as pulses, breathing, facial gestures, and joint movements. Figure 25B illustrates how the conductive components of PSGP sensors respond to stretching and compression.

**Figure 25:**
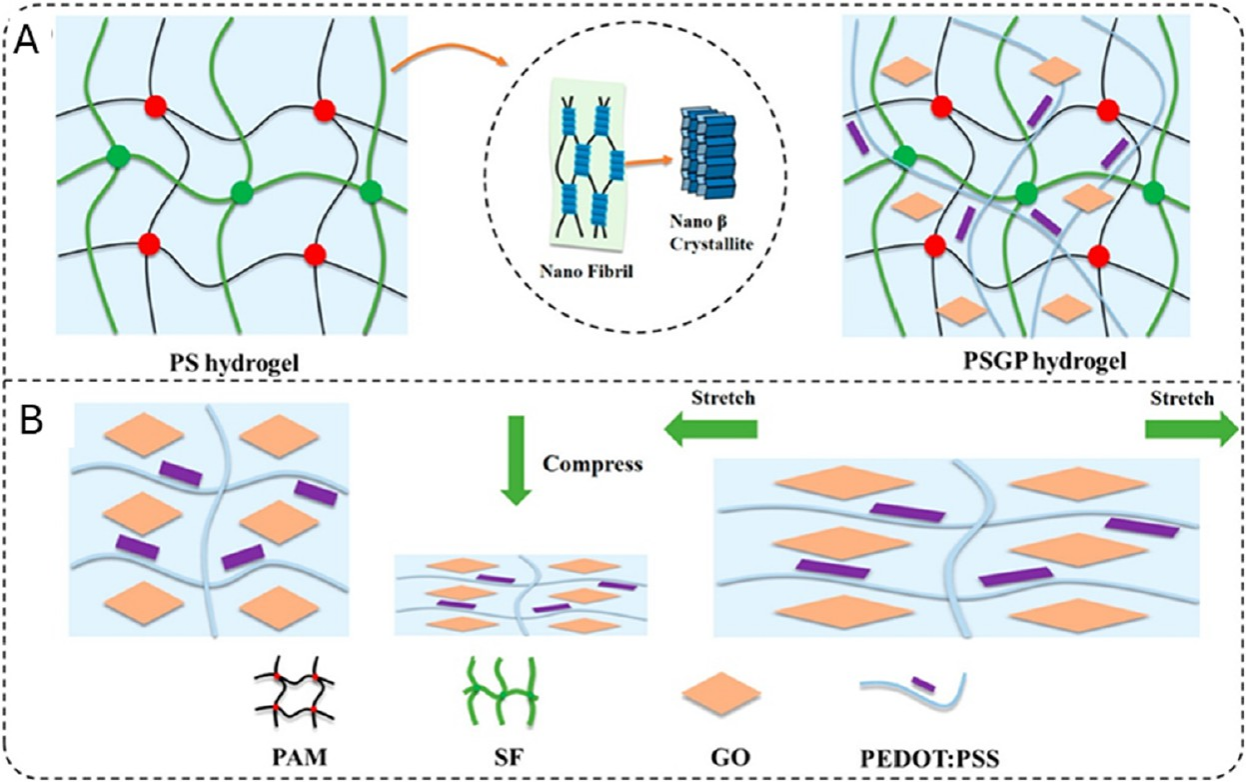
(A) Possible structures of PS (PAM/SF) and PSGP (PAM/SF/GO/PEDOT:PSS) hydrogels, highlighting the presence of SF nanofibrils and the intertwining of GO sheets through hydrogen bonding. (B) Diagram illustrating how the conductive components (GO and PEDOT:PSS) in PSGP sensors respond to compression and stretching, resulting in changes in pressure or strain. Reproduced from He et al.^722^ © 2020 American Chemical Society.

Besides hydrogen bonding (H-bonding), other noncovalent interactions, including dipole–dipole, ionic, and van der Waals interactions, can impart additional properties to hydrogels. Liu et al.^723^ reported hydrogels consisting of a soft, homogeneous polymer network combined with a hard, dynamic network of Fe^3+^-cross-linked cellulose nanocrystals (CNCrys–Fe^3+^). These hydrogels exhibited excellent mechanical properties due to the synergistic interaction between the two networks. The dynamic CNCrys–Fe^3+^ coordination bonds dissipated energy under stress, while the homogeneous polymer network facilitated smooth stress transfer. Furthermore, the reorganization of CNCrys and Fe^3+^ through ionic coordination allowed the hydrogels to self-heal within 5 min without requiring external healing agents or *stimuli*. The functional network hydrogels were explored as strain sensors, with their conductivity varying in response to strain. This is illustrated in Figure 26Ai, where the brightness of an LED decreases as the strain increases from 0 to 150%. Step-like resistance changes during loading–unloading cycles, which could be repeated more than 200 times, are shown in Figure 26Aii. The hydrogel sensor was also capable of monitoring finger joint motion, as depicted in Figure 26B. The monitoring of physiological signals, such as breathing and pulse, is demonstrated in Figure 26D–F. For pulse monitoring, in particular, reproducible signals corresponding to 78 beats/min at rest and 102 beats/min after exercise were obtained.

**Figure 26:**
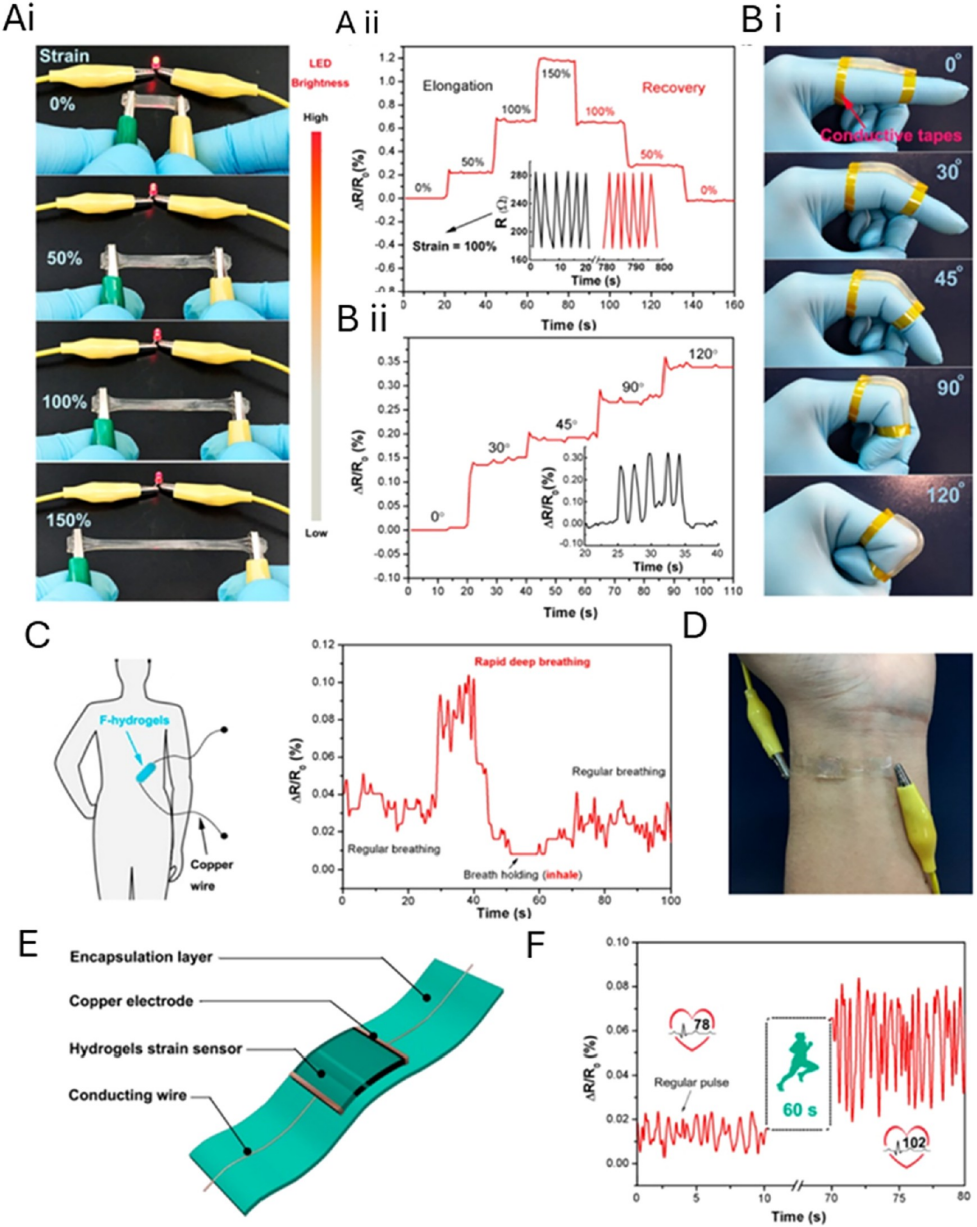
(A) (i) LED brightness decreases as the F-hydrogel is elongated, indicating a decrease in conductivity with strain. Step changes in resistance are shown in (Aii) and (Bii) for elongation and finger bending, respectively. (B) (i) Images showing the index finger bending at different angles. (C) Relative resistance changes during regular and rapid breathing, obtained using the wearable sensors shown schematically. (D) Image of a sensor placed on the wrist of a volunteer. (E) Schematic representation of the F-hydrogel sensors. (F) Response of the strain sensor before and after exercise, corresponding to a volunteer running. The measured heart rates were 78 beats/min at rest and 102 beats/min postexercise. Reproduced from Liu et al.^723^ © 2017 American Chemical Society.

Another example involving the combination of H-bonding and other noncovalent interactions was reported in multifunctional hydrogels suitable for various applications, including strain sensors.^724^ The hydrogel, referred to as DTPAM, was synthesized by incorporating polydopamine-coated talc (PDA–talc) nanoflakes into a PAM hydrogel. The key intermolecular interactions are illustrated in the scheme in Figure 27, which outlines the fabrication process of DTPAM. In particular, intercalating dopamine molecules ensured higher dispersion of talc and preservation of catechol groups within the hydrogel. The final properties were governed by π–π stacking of PDA chains and H-bonding in the hydrogel. DTPAM exhibited characteristics such as stretchability, biocompatibility, self-healing, and adhesiveness, which allowed its use in strain sensors to monitor human motion.

**Figure 27:**
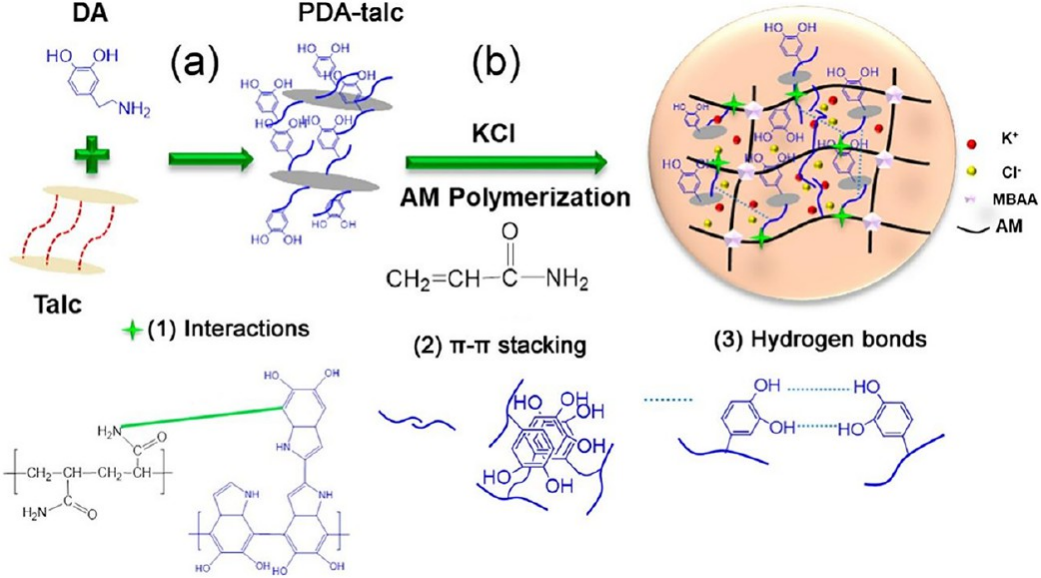
Fabrication process of the DTPAM hydrogel, showing how dopamine (DA) molecules intercalated within talc layers (a) are incorporated into a PAM hydrogel (b). The distinct interactions in DTPAM are highlighted: (1) Amide groups in PDA chains, formed in the presence of talc, enable linkage with PAM chains; (2) π–π stacking between PDA chains; (3) H-bonding within the hydrogel. Reproduced from Jing et al.^724^ © 2018 American Chemical Society.

The incorporation of polymer hydrogel microspheres into a matrix hydrogel enabled the exploitation of dynamic hydrogen bonds, hydrophobic interactions, and covalent bonds to produce a multiscale hydrogel.^725^ The design strategy, illustrated in Figure 28, involves immersing microspheres of one of three types of polymer into an aqueous solution of acrylamide/1-vinyl-3-butylimidazolium bromide (AM/[VBIm]­Br) and the cross-linking agent *N*,*N*′-methylene-bis-acrylamide (MBA). Polymerization in the solution and within the microspheres resulted in a double network comprising a chemical network from PAM-[VBIm]­Br and physical entanglements with the microspheres as nodes. Additionally, the hydrophilic groups on PAM and the microspheres formed dynamic hydrogen bonds, enhancing physical cross-linking strength. These microspheres improved the hydrogel structure, resulting in superior sensing performance.

**Figure 28:**
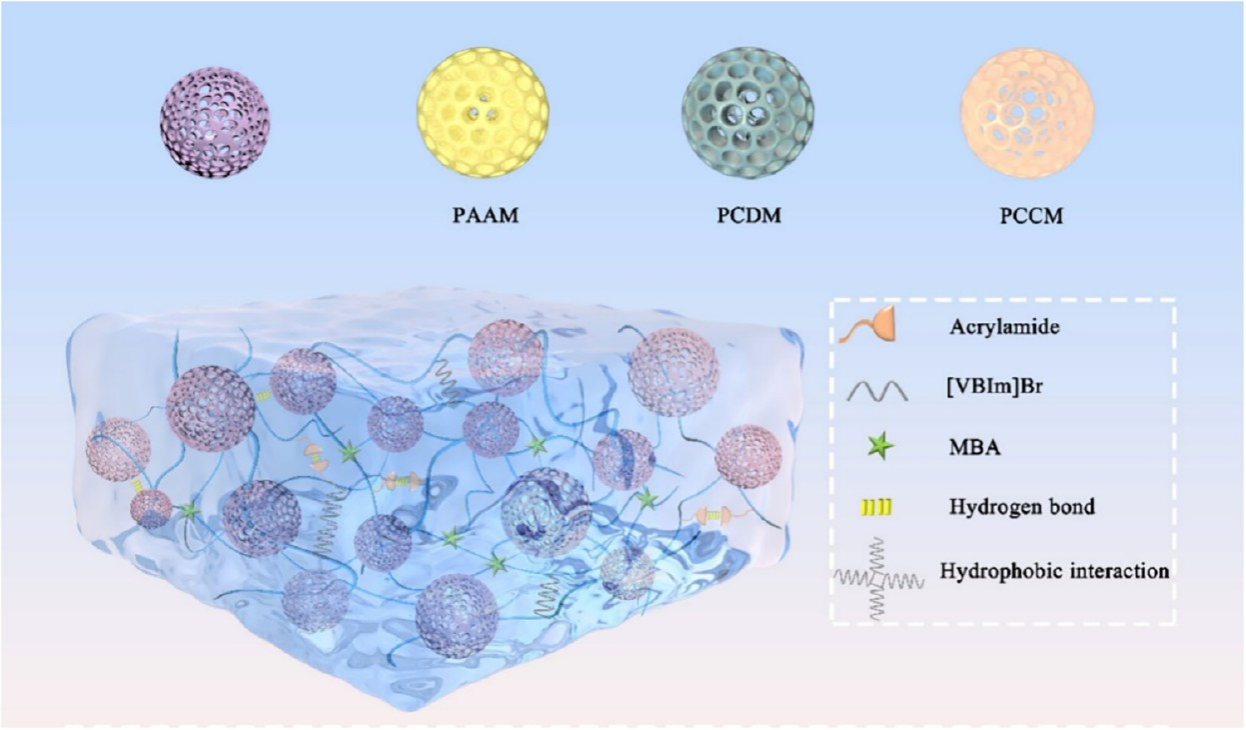
Illustration of the design strategy for producing the multiscale hydrogel. The image depicts microspheres that can be derived from one of three polymers: poly­(acrylic acid) hydrogel (PAAM), poly­(cyclodextrin) hydrogel (PCDM), and poly­(cyclodextrin)-chitosan hydrogel (PCCM). The inset highlights the groups involved in hydrogen bonding and hydrophobic interactions. Reproduced from Zhao et al.^725^ © 2024 American Chemical Society.

Dynamic cross-linking can also be essential for conductive hydrogels used in strain and pressure sensors, enabling them to withstand cold temperatures without freezing.^726^ A hydrogel consisted of a double network of chitosan-poly­(acrylamide-*co*-acrylic acid) [CS-P­(AM-*co*-AA)], with dynamic cross-links within the chitosan physical network and through ionic coordination with Fe ions. While the mechanical properties of the hydrogel, such as tensile strength, compressibility, and self-recovery, were enhanced by immobilizing ions in the dynamic cross-links, the free ions in the hydrogel imparted a high conductivity and freezing tolerance.

The examples above illustrate how hydrogels can be designed to exploit intermolecular interactions and achieve variable properties. Other strategies also exist to enhance material properties for wearable sensors. For instance, microporous dielectric elastomers with great piezocapacitive effects have been used in tactile pressure sensors.^727^ The enhancement in piezocapacitive effect was obtained using a three-dimensional (3D) microporous Ecoflex elastomer, which is highly deformable upon compression. Wearable sensors with piezocapacitive effects were employed in various applications, as illustrated in Figure 29. A sensor was attached to the fingertips of a robot to measure the pressure exerted on a target object—specifically, a lightbulb, as shown in Figure 29A. The seizing motion at different displacements was reflected in the relative changes in capacitance, as depicted in Figure 29B. This figure also highlights the full recovery of the capacitance signal upon the release of the object. Due to the elastomeric nature of the sensor, it serves as a cushion, enabling the robotic finger to handle the lightbulb without breaking it. In a health-monitoring application, the sensor was adhered to a volunteer’s wrist, as shown in Figure 29C, to measure the pulse signal presented in Figure 29D. This signal was confirmed to originate from the wrist pulse by comparing it with the sensor signal recorded on the palm. As demonstrated in Figure 29E, the palm signal was significantly lower, comparable to the noise level of the pulse signal. Due to its fast response, the sensor was also capable of measuring a single pulse signal. The result, shown in Figure 29F, represents a typical single pulse signal of a healthy adult male, characterized by two peaks (*P*_1_ and *P*_2_) that provide information on arterial stiffness. For example, the radial artery augmentation index (AIr) can be defined as AI_r_ = *P*_2_/*P*_1_. Furthermore, the pulse pressure *P*_1_ was approximately 3 Pa, consistent with measurements from other wearable devices.

**Figure 29:**
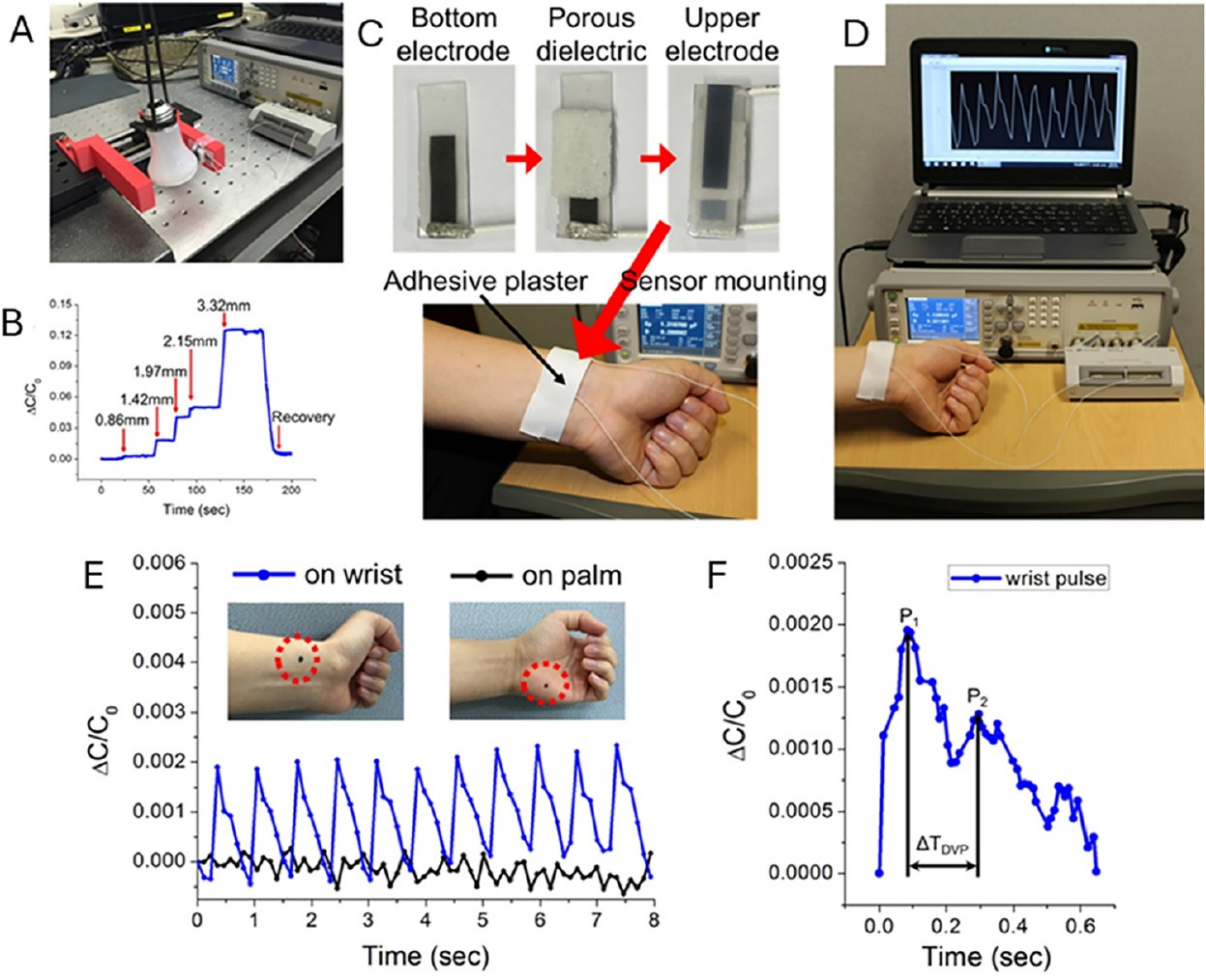
(A) Photograph of robotic fingers integrating the piezocapacitive sensor while handling a lightbulb. (B) Displacements of one finger and the corresponding capacitive responses, showing full recovery of the capacitance signal when no pressure is exerted. (C) Image of a sensor attached to the wrist of a volunteer. (D) Pulse signal recorded from the volunteer. (E) Comparison of the signals measured at the pulse and the palm, demonstrating that the measurement at the pulse corresponds to the pulse signal. (F) Capacitance response of a single pulse, featuring the characteristic two peaks. Reproduced from Kwon et al.^727^ © 2016 American Chemical Society.

The porous nature of materials for pressure and strain sensors can be exploited in various ways. A porous network with graphene and PDMS was used for pressure and strain sensing to recognize walking states and measure wrist blood pressure.^728^ Capacitive pressure sensors were developed with a porous pyramid dielectric layer, which was insensitive to strain and temperature.^729^ A similar concept was employed to develop high-performance capacitive pressure sensors using tilted micropillar dielectric arrays.^730^ In yet another example, electromechanical sensors were developed using poly­(lactic acid) (PLA) electret films with a cellular structure.^731^ This cellular structure was essential for increasing the charge stored in the electret film, thereby enhancing pressure sensitivity. The example of pressure sensors made from PLA films highlights the overarching principles that define wearable sensors. Specifically, the sensing design involves careful selection of materials, molecular and film architectures and fabrication conditions. In the case of PLA sensors, the polymer serves as a biodegradable electret that can be engineered with a cellular microstructure. Under optimized corona charging conditions, PLA cellular electrets exhibit enhanced polarization, resulting in high pressure sensitivity. The pressure sensitivity was measured at 1000 pC/kPa within a detection range of 0.03–62.4 kPa. These fully degradable sensors were utilized to monitor body movements and detect subtle physiological signals, as illustrated in Figure 30.

**Figure 30:**
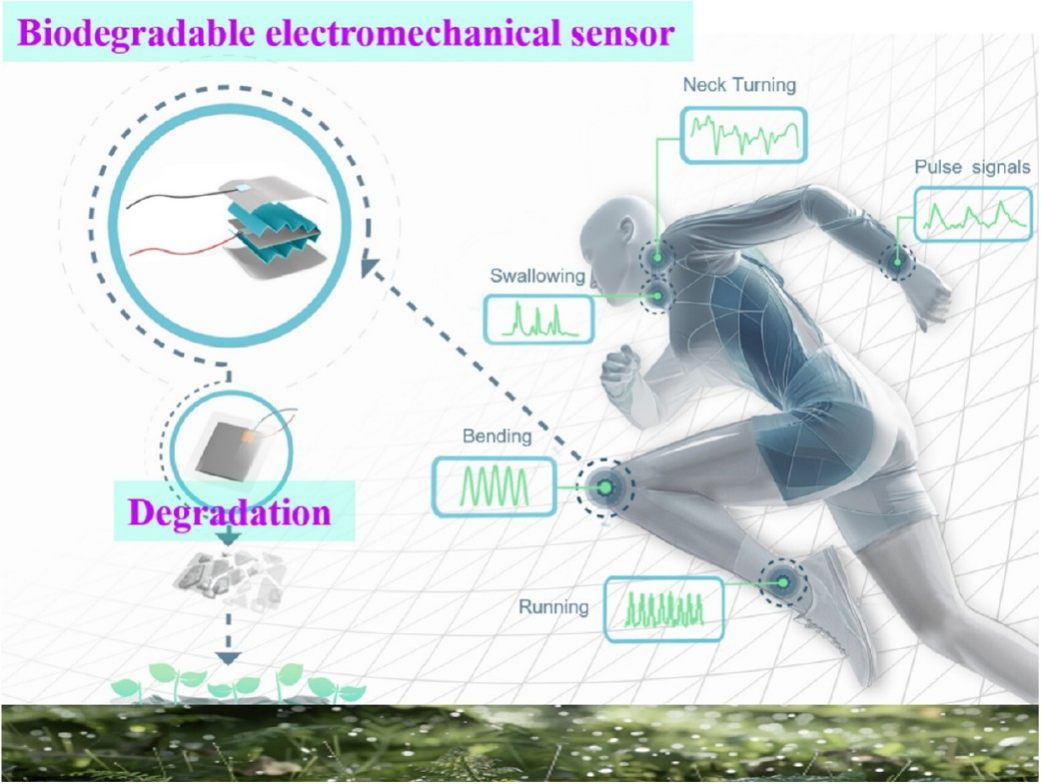
Schematic illustration of the various uses for the PLA pressure sensors, which are fully degradable. The sensors can be used to detect various body movements and pulse signals. Reproduced from Qin et al.^731^ © 2024 American Chemical Society.

The combination of pyramidal and porous structures was also investigated to develop piezoresistive pressure sensors capable of achieving both high sensitivity and linear response.^732^ The unique design of the dual sensor is illustrated in Figure 31. The sensor is described as dual because it integrates a micropatterned (micropyramidal) PDMS component with a porous component made of carbon black and filter paper. These two parts, shown in Figure 31B, face an interdigitated electrode (IDE). The distinct ways the two components respond to pressure are depicted in Figure 31C,D. The complete sensor architecture, shown in Figure 31E, consists of these components sandwiched between two PET films, one of which contains the interdigitated electrodes. At low pressures (<1 kPa), the micropyramidal component provides high sensitivity, while at higher pressures, the conductive filter paper component ensures both high sensitivity and a linear response within the 1–20 kPa range.

**Figure 31:**
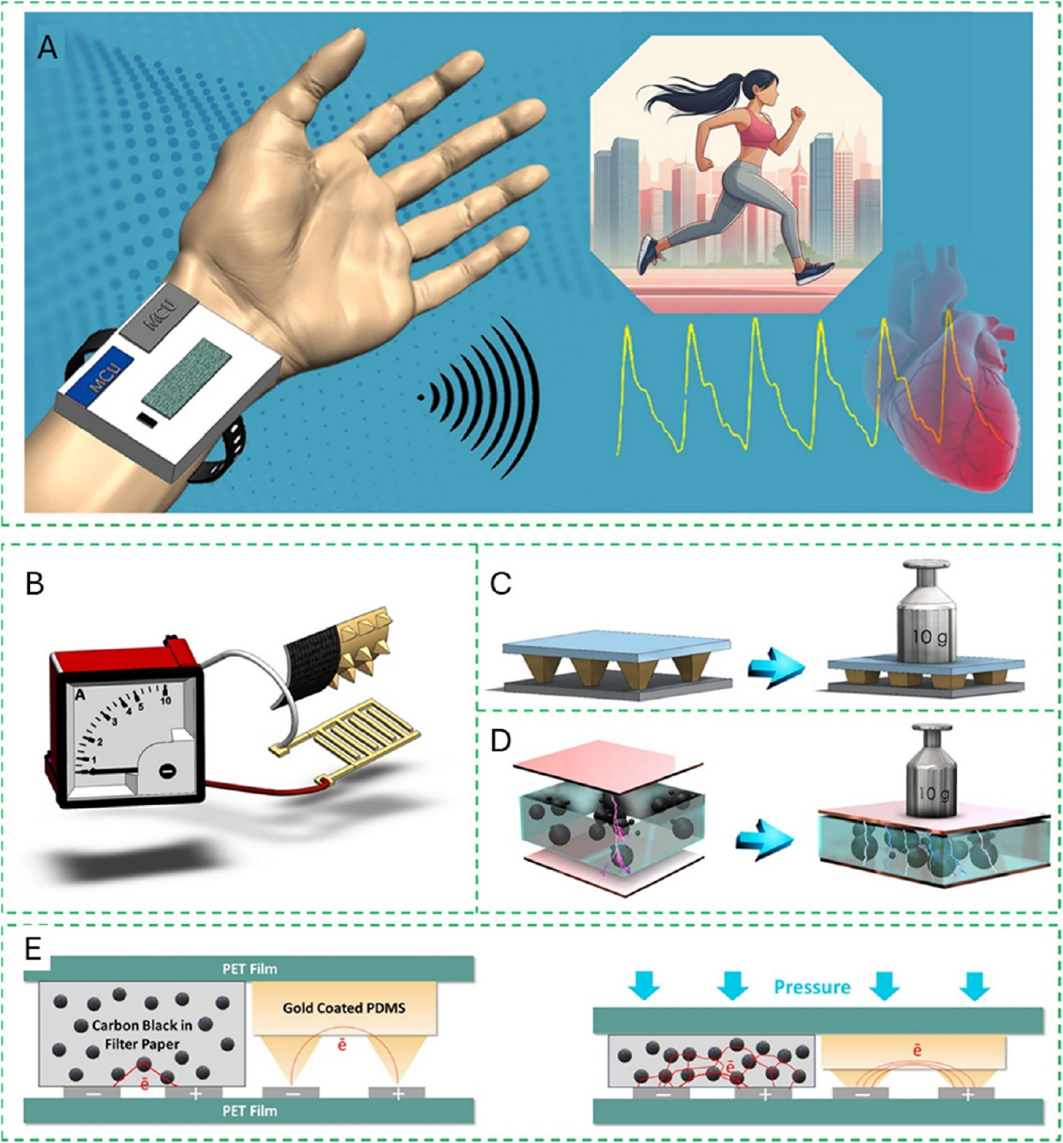
(A) Illustration of the pressure sensor placed on the pulse for monitoring the pulse signal. (B) Two-component sensor deposited on interdigitated electrodes, electrically connected to measure the piezoresistive signal. (C) Diagram showing how the micropyramidal sensor responds to pressure, exhibiting sensitivity to low pressures. (D) Diagram showing the response of the porous sensor component, which provides a linear response and is sensitive to higher pressures. (E) Architecture of the dual sensor, highlighting the distinct ways the two components respond under pressure. Reproduced from Jafarizadeh, B. et al.^732^ © 2024 American Chemical Society.

This section has primarily focused on hydrogel-containing sensors as these are among the most widely used materials for such applications. They not only offer versatility in materials engineering to enhance sensing properties but also provide excellent mechanical and functional adaptability. Another class of widely used materials is nanomaterials, particularly graphene. Because of its exceptional mechanical and electrical properties, graphene has been incorporated into various strain sensors, often in combination with other materials. For instance, wearable sensors created via the layer-by-layer assembly of graphene and yarns from different sources have been employed for human motion monitoring.^733^ Other nanomaterials are also used for similar purposes, primarily to impart electrical conductivity. These include carbon nanotubes,^734^ MXenes,^735^ and silver nanowires.^736,737^ Another approach to creating conductive materials for strain and pressure sensors involves the incorporation of LMs. For example, a microfluidic sensor employing a wave-patterned LM demonstrated superelasticity, sensitivity, and low hysteresis, making it suitable for human activity monitoring.^738^

There are numerous advantages to integrating wearable sensors with textiles, as already discussed in Section 5.6. Examples of textile-containing sensors include a washable piezoresistive sensor made from a composite of AuNW-impregnated knitted cotton fabric.^739^ Another noteworthy example involves strain sensors created from knitted textiles combined with polymers and elastomer yarns.^740^ Additionally, Lou et al.^741^ demonstrated a triboelectric textile sensor produced from core–shell yarns (see Section 10.1 for triboelectric nanogenerators). Figure 32 shows textile pressure sensors incorporating different polymers, with stainless steel yarns used as electrode layers. The two polymers selected, nylon and polytetrafluoroethylene (PTFE), were chosen for their mechanical properties and high abrasion resistance. The textile sensors could be integrated into standard clothing or attached to the human skin. Therefore, human movements could be monitored, including hand, knee, and elbow motions, with the bending speeds and angles. This type of monitoring helps to analyze patients with potential mobility disorders. Another health-monitoring application is for measuring the pulse wave at the carotid artery.

**Figure 32:**
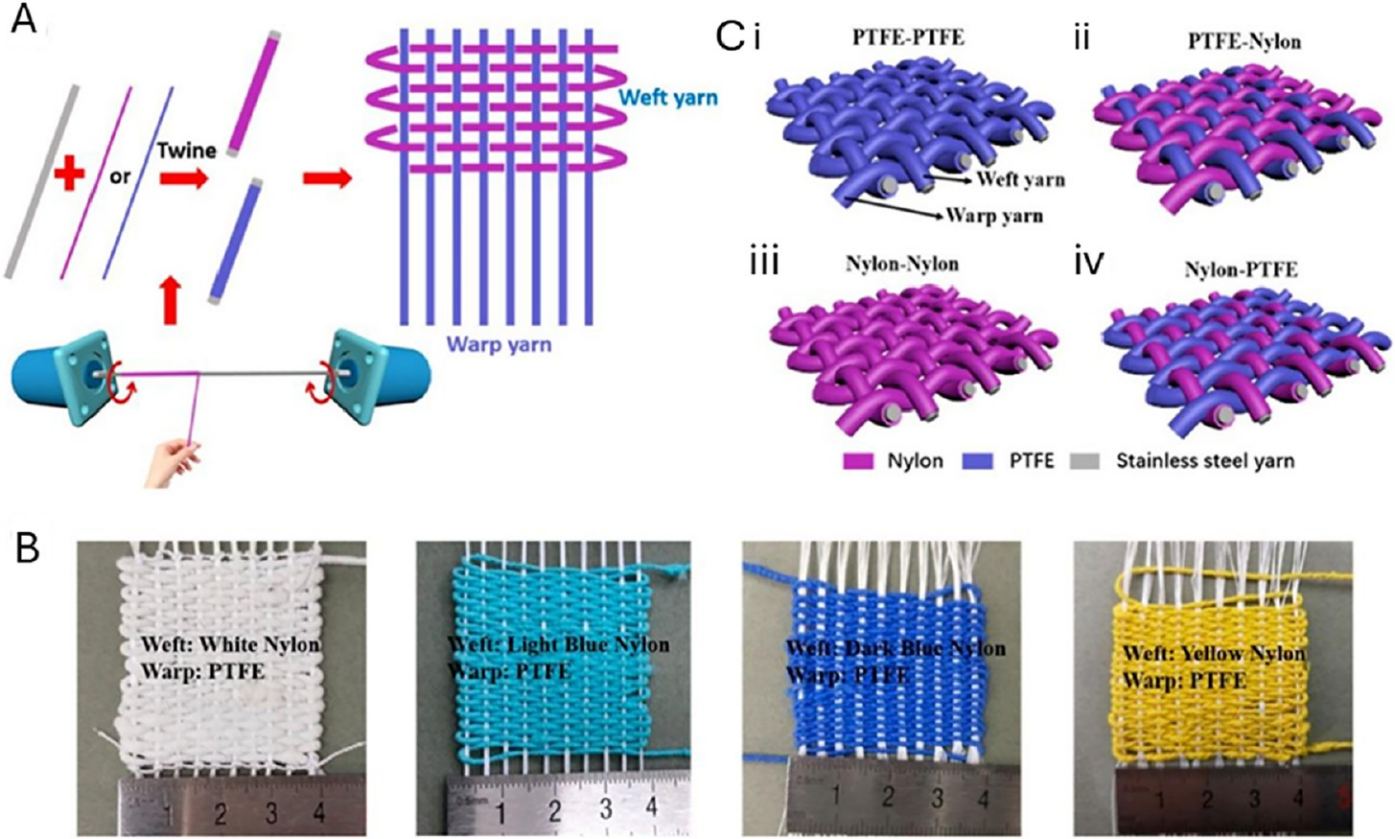
(A) Yarns that were used to produce the sensors. (B) Photographs of different combinations of knitted textiles with different colors. (C) Schematic diagram of knitted textiles including stainless steel yarns. Reproduced from Lou et al.^741^ © 2020 American Chemical Society.

### 6.2 Blood Pressure Sensors

Blood pressure sensors are useful for cardiovascular health management as they can be integrated into daily routines and provide continuous, real-time monitoring.^742^ This section offers a concise overview of the field, highlighting the role of blood pressure monitoring, optimal on-body sensor locations, innovative device strategies, representative applications, and ongoing challenges that require further research and development.

Blood pressure measures the internal pressure exerted by the heart muscles to pump blood throughout the body. Maintaining blood pressure within a specific range is essential for optimal health. Abnormal levels—whether elevated (hypertension) or reduced (hypotension)—can signal dysfunctions in the cardiovascular system. Blood pressure is divided into two types: systolic blood pressure (SBP) and diastolic blood pressure (DBP).^743^ While SBP measures the contracted status of the heart, DBP characterizes the relaxed status.^744^ Wearable blood pressure sensors for long-term monitoring have attracted much attention for the early detection and prevention of heart diseases, such as stroke, hypertension, coronary artery diseases, and myocardial ischemia.^745^ Early detection enables timely interventions, including lifestyle modifications and pharmacological treatments, which can significantly reduce the risk of heart attack, stroke, kidney disease, and other life-threatening complications.

The choice of on-body locations for soft wearable blood pressure sensors is crucial to the accuracy and reliability of the sensing systems. The sensor’s placement affects the quality of the signal captured and impacts the user’s comfort during use. The upper arm, traditionally the standard location for blood pressure measurement, offers proximity to the brachial artery, a major blood vessel that provides a strong and reliable signal.^746^ However, upper arm placement may be less convenient for continuous monitoring due to its potential interference with daily activities. The wrist, a more accessible location, has gained popularity for wearable blood pressure sensors. Wrist-worn devices allow for discrete monitoring and integration with existing smartwatch and fitness tracker designs. However, the radial artery at the wrist is smaller and more superficial than the brachial artery, potentially leading to less accurate readings.^747^ Alternative locations, such as the finger, earlobe, and ankle, are also under investigation. Each location presents advantages and challenges in terms of signal quality, user comfort, and sensor design.

The demand for noninvasive, continuous, and precise blood pressure monitoring has driven the development of soft wearable sensors with various sensing mechanisms. Examples include strain sensors, ultrasonic sensors, and optical sensors. Among these, strain sensors have received the most attention for their simplicity, low cost, and good conformability. Currently, mechanical sensors used in blood pressure monitoring are mainly categorized into four types:

1. Resistive sensors use the change in electrical resistance of a material under applied pressure. These offer a relatively simple and cost-effective approach with good sensitivity.^7^ When external pressure is applied, the geometric shape of the active material changes, causing restructuring or even breakage of the conducting network and hence significantly increasing the resistance.^748,749^ These sensors can be fabricated using a variety of flexible materials, including metal nanoparticles,^750^ metal nanowires,^751^ carbon-based nanomaterials,^749^ conductive polymers,^752^ and MOF compounds.^753,754^ A major challenge of resistive sensors, however, is the repeatability. Recently, cracks have been introduced in the active material to control the conducting pathway, offering an excellent combination of sensitivity, strain range, and repeatability.^748^ Figure 33A shows a set of two silver nanowire-based strain sensors with surface crack design to capture the pulse wave velocity, which can be further translated to blood pressure.^748^
2. Capacitive sensors, which measure the change in capacitance due to pressure-induced deformation, provide excellent linearity and repeatability. These sensors often employ flexible electrodes and dielectric materials, enabling conformal contact with the skin and accurate detection of pressure variations.^755^ Recent advancements, such as the design of a microstructured dielectric layer,^756^ have improved sensitivity. Figure 33B shows a wireless capacitive pressure sensor and the measured pulse pressure waveform.^757^
3. Piezoelectric sensors, which generate electrical charges in response to mechanical stress, offer the advantage of self-powered operation.^758^ This type of sensor excels in capturing rapid pulse waveforms due to its millisecond-level response time.^759^ Materials such as polyvinylidene fluoride (PVDF) and its copolymers are commonly used in flexible piezoelectric sensors, providing a balance of piezoelectric properties, flexibility, and biocompatibility.^760^ Figure 33C shows a 3D-printed PVDF-BaTiO_3_ sensor using direct-ink writing. The sensor was calibrated against a standard cuff-based oscillometric device and measured the blood pressure of a subject by placing the sensor near the radial artery.^761^
4. Triboelectric sensors operate based on triboelectric effects and electrostatic induction principles. When materials come into contact and separate, a potential difference is generated, inducing transient currents that enable highly sensitive pressure detection. Triboelectric sensors are known for ultrahigh sensitivity and extremely low power consumption. They can even achieve fully self-powered monitoring by generating electricity through slight relative motion between the skin and sensor, offering a promising solution for long-term portable heartbeat monitoring. Figure 33D shows a flexible triboelectric nanogenerator-based wearable blood pressure sensor with high sensitivity (resolution of 1–6 kPa) and fast response time (8.5 ms).^762^

**Figure 33:**
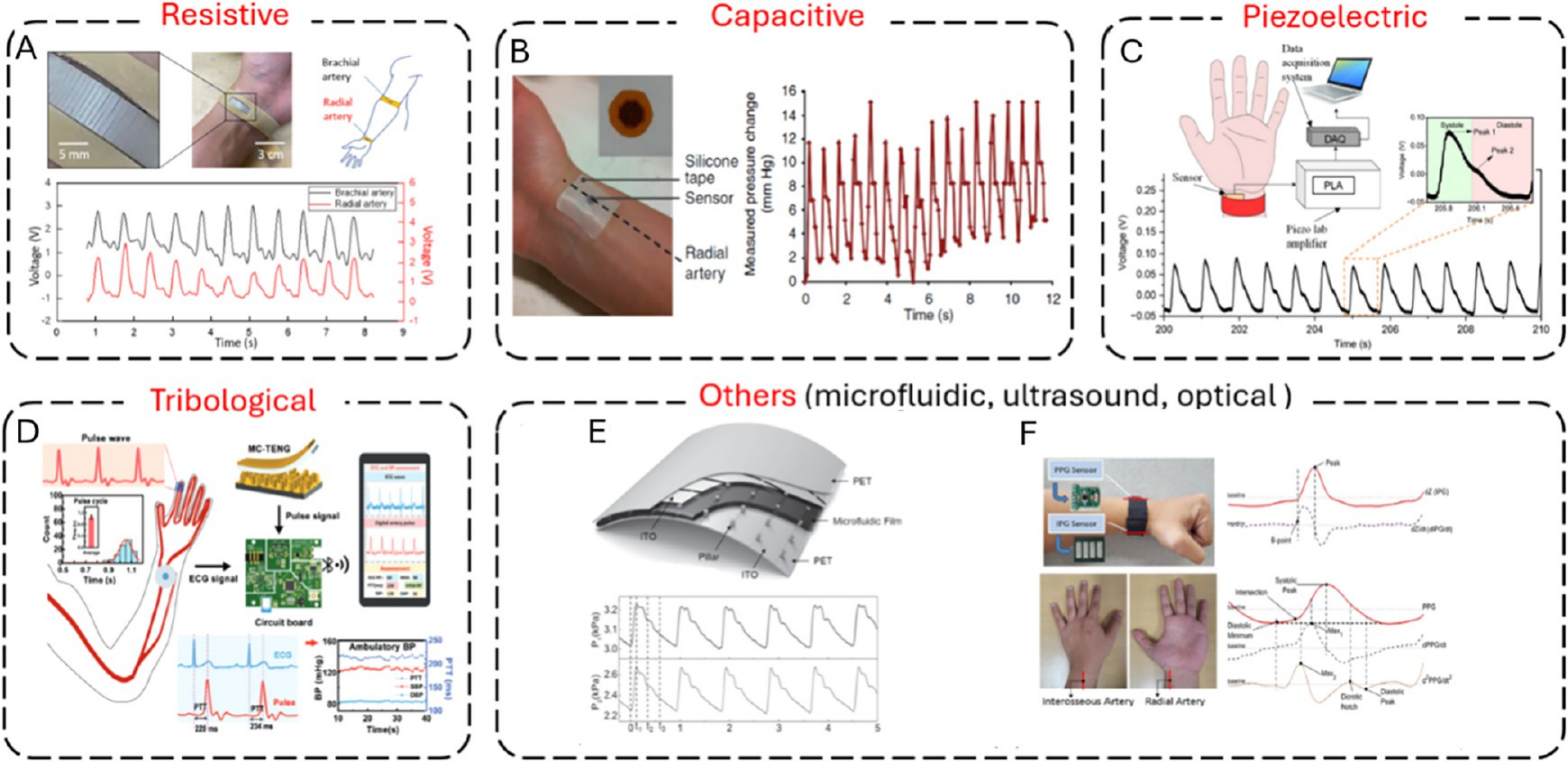
Soft wearable blood pressure sensors. (A) A pair of resistive strain sensors with surface crack design captures the pulse wave velocity. Reproduced from Wu et al.^748^ © 2023 American Chemical Society. (B) A wireless capacitive pressure sensor (left) and the measured pulse pressure waveform of a human subject with a heart rate of 82 bpm (right). Reproduced with permission from Chen et al.^757^ © 2014 Springer Nature. (C) Pulse wave measured by a 3D-printed piezoelectric pressure sensor. The inset figures show the peaks representing systolic pressure and diastolic pressure, respectively. Reproduced with permission from Mandal et al.^761^ © 2024 John Wiley & Sons. (D) A real-time pulse and blood pressure monitoring system developed using triboelectric nanogenerators. Reproduced with permission from Zhang et al.^762^ © 2025 John Wiley & Sons. (E) A microfluidic pressure sensor enabled by fluid-driven impedance changes. Reproduced with permission from Li et al.^763^ © 2014 John Wiley & Sons. (F) A multimodal wrist biosensor for wearable cuff-less blood pressure monitoring. Reproduced from Rachim et al.^765^ Available under a CC BY 4.0 license. © 2019 Springer Nature.

Other sensing mechanisms have been used for detecting blood pressure, including microfluidic channels,^763^ ultrasound technology,^764^ and optical sensors,^765^ together with advanced signal processing algorithms to enhance accuracy and reliability.^766^ Figure 33E shows a microfluidic pressure sensor that measures the impedance change of internal fluid due to external pressure.^763^ Flexible piezocomposite ultrasonic sensors have also been used to monitor continuous blood pressure through ultrasonic motion tracking of the blood vessel wall.^767^ Optical sensors, utilizing PPG, measure changes in light absorption or reflection caused by blood flow variations. This method involves the combination of LEDs with photodetectors. When light passes through the epidermis, it is absorbed and scattered by blood and surrounding tissues, resulting in changes in the reflected light intensity that synchronize with vascular pulsation (Figure 33F). PPG-based sensors are noninvasive and can be integrated into compact, wrist-worn devices. However, their accuracy can be affected by motion artifacts and variations in skin pigmentation.^768^

Soft wearable blood pressure sensors can be used in remote patient monitoring to facilitate timely interventions and reduce the need for frequent clinic visits.^769^ This is beneficial for elderly patients or those residing in rural areas with limited access to healthcare facilities. In sports medicine, wearable blood pressure sensors can monitor athletes’ cardiovascular responses during training and competition.^770,771^ Furthermore, soft wearable blood pressure sensors hold promise for early diagnosis and prevention of preeclampsia in pregnant women, a life-threatening condition characterized by high blood pressure and organ damage.^772^ Wearable blood pressure sensors are also highly beneficial for infants, as they are more vulnerable to the discomfort and risks associated with traditional monitoring devices.^773^

### 6.3 Sensors to Monitor Heart Rate

Heart rate (HR), defined as the frequency of cardiac pulsations, serves as an important biomarker for cardiovascular function. Its value is dynamically regulated by multiple factors, including age, gender, genetics, health status, circadian rhythm, and psychological stress.^774^ Derivative parameters such as normal resting HR and heart rate variability (HRV) can reveal cardiovascular status, with the HR rate reflecting long-term cardiovascular risk.^747,775^ HRV may be related to autonomic system integrity by analyzing fluctuations between adjacent heartbeat intervals. HRV abnormalities are associated with arrhythmias, heart failure, and the risk of sudden cardiac death.^776^ In sports science, maintaining training intensity within 70–85% of maximum heart rate can optimize cardiopulmonary adaptation and reduce the risk of myocardial overload.^777^ Heart rate monitoring is crucial for evaluating pharmaceutical efficacy. For instance, β-receptor blockers require dynamic dose adjustments based on patients’ real-time heart rates.^778^ Thus, long-term, precise heart rate monitoring is key in preventive medicine and chronic disease management.

#### 6.3.1 Electrocardiogram Monitoring

ECG is the gold standard for noninvasive heart rate determination.^779^ Blood pumping is regulated by coordinated contraction and relaxation of myocardial cells, which generate complex electrical patterns through bioelectric potential propagation.^780^ ECG captures these electrical signals via electrodes. After the signal processing step, the heart rate is calculated by identifying cardiac cycles. Although ECG-based heart rate measurement is highly accurate, it is significantly affected by electrode adhesion and skin impedance.^781^ The challenges of ECG signal acquisition are mainly due to motion interference and long-term wearability.^782^ During intense motion, the relative displacement between electrodes and skin and sweat accumulation may lead to motion artifact noise.^783^ Researchers have developed epidermal dry electrodes with a porous structure and ultrathin thickness to address these issues.^784–787^ These types of electrodes have the advantage of improved adhesion, breathability, wearing comfort, and long-term stability.^788^ Centers for ECG analysis obtained using wearable devices are arising, promoting more accessible and frequent healthcare.^789^

#### 6.3.2 Photoplethysmography Monitoring (PPG)

PPG achieves heart rate monitoring by detecting light intensity fluctuations caused by blood volume changes in the blood vessels.^790^ The resulting waveform can be further analyzed for parameters such as systolic amplitude, pulse width, and peak-to-peak intervals, which are used to evaluate vascular disease and autonomic function.^791^ With advancements in DL models, PPG signals can now be used to predict cardiovascular disease risks, expanding their diagnostic potential.^792^ However, the PPG signal quality is easily affected by motion artifacts, ambient light interference, and individual differences in skin reflectance.^793,794^

#### 6.3.3 Mechanical Sensing

When blood is pumped through the blood vessels, it induces mechanical waves and strain, which propagate throughout the skin. Detecting these mechanical waves and strain on the skin enables the capture of arterial pulsation signals or seismographic signals, indirectly inferring heart rate. Strain detection is primarily achieved through various mechanical (strain or pressure) sensors.^795^ Sensitivity and stability are the major concerns for this type of sensor.

#### 6.3.4 Sensor Form Factors and Anatomical Considerations

The effectiveness of heart rate measurement largely depends on balancing the signal quality and wearing comfort. Traditional 12-LED ECG uses electrodes to capture cardiac electrical activity with extreme precision, but the large number of patches limits its practicality for daily applications. Detection methods based on blood flow can monitor arteries and capillary networks.^795^ To collect high-quality heart-rate-related signals, various sensors need to be stably worn on the skin surface. The typical wearable form factors include chest straps, wristbands, earpieces, clothing integration, and patch and electronic skin types:

#### 6.3.5 Chest-Worn

Chest strap heart rate monitors, being close to the heart source, provide optimal signal quality with secure fixation and strong resistance to motion interference (Figure 34a).^796^ They are suitable for high-intensity exercise and precise monitoring.^796,797^ As one of the earliest commercialized heart rate monitoring devices, chest strap monitors use two or more electrodes applied to the chest skin to collect cardiac electrical signals, calculating heart rate after signal processing. These sensors possess high accuracy, high temporal resolution, and strong motion interference resistance, but as they require stable adhesion to the skin surface, they need adhesives or relatively high pressure, potentially causing skin discomfort with long-term wear.^797^ Modern chest-worn heart rate sensors are integrated with signal processing and wireless transmission modules into a compact patch (Figure 34d).^788,798^ The patch is attached to the skin using hypoallergenic adhesive, eliminating the need for preload pressure. This design enhances the long-term wearing comfort and stability.

**Figure 34:**
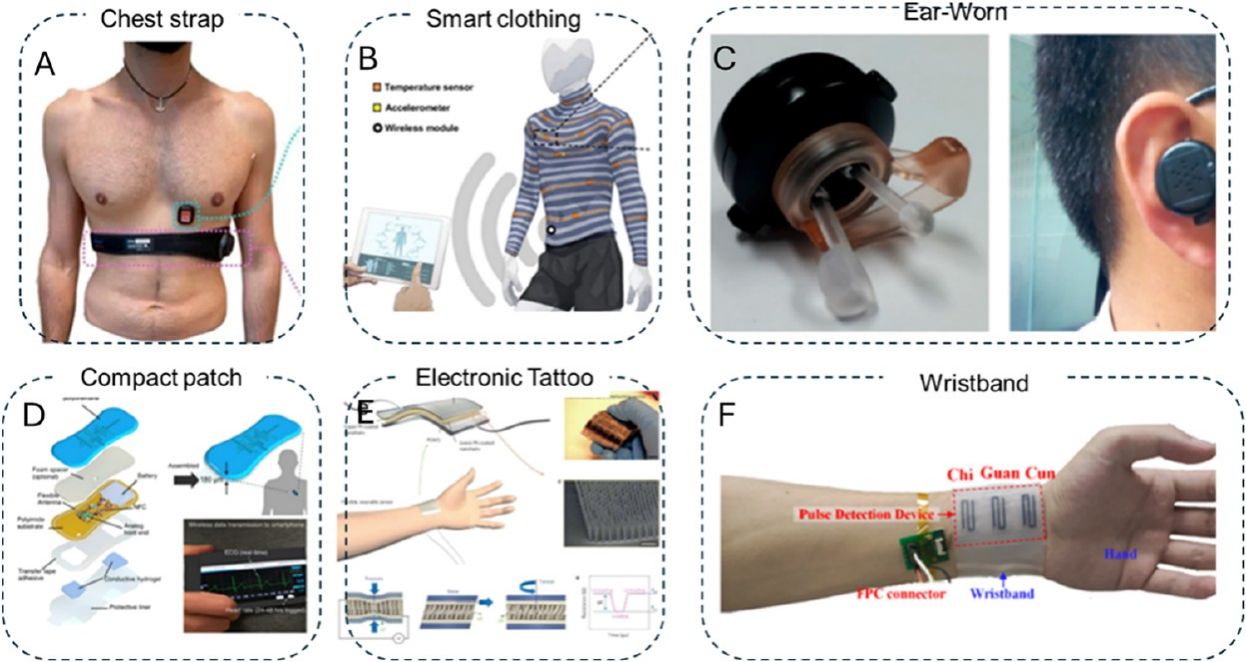
Soft wearable heart rate monitoring devices. (A) Chest strap ECG sensor. Reproduced from Romano et al.^796^ Available under a CC BY 4.0 license. © 2022 MDPI. (B) Smart clothing with PPG sensors. Reproduced from Wicaksono et al.^653^ Available under a CC BY 4.0 license. © 2020 Springer Nature. (C) Ear-Worn Piezoelectric Sensor. Reproduced from Park et al.^803^ Available under a CC BY 4.0 license. © 2015 MDPI. (D) Compact patch integrating ECG sensor, signal processing, and transmission module. Reproduced from Lee et al.^788^ Available under a CC BY 4.0 license. © 2018 Springer Nature. (E) Epidermal electronic heart rate device based on triboelectric Sensors. Reproduced with permission from Pang et al.^749^ © 2012 Springer Nature. (F) Wristband pulse sensor-based piezoresistive technology. Reproduced from Wang et al.^800^ Available under a CC BY 4.0 license. © 2022 MDPI.

#### 6.3.6 Wristbands

Wristband heart rate sensors are known for their excellent portability and daily wearing comfort, with ease of use and high user acceptance.^799^ They are particularly suitable for daily activities and general fitness monitoring (Figure 34f).^800^ As the most common form of heart rate monitoring today, wristband sensors are mainly used in smartwatches and smart bracelets. These devices can employ PPG or pressure sensors. PPG sensors have the advantages of convenience and comfort, while typically being integrated with accelerometers and gyroscopes to compensate and eliminate motion artifacts through algorithms.^801^ Pressure-sensor-based wristbands capture strain produced by arterial pulsation; thus, they need to closely adhere to the wrist pulse area and achieve stable pressure sensing by applying preload pressure. By extracting key features of the pulse pressure waveform and analyzing with preload pressure and skin temperature, additional physiological parameters such as blood pressure can be obtained, also ensuring good monitoring stability.^802^ However, because of its constraining effect on the skin, long-term wearing comfort presents challenges. The latest wristband heart rate sensors have begun to adopt multisensor fusion technology, combining PPG and pressure sensing and processing data using machine learning algorithms, which has improved monitoring accuracy and anti-interference capability.^799^

#### 6.3.7 Ear-Worn

Ear-worn heart rate monitors feature minimal motion interference and excellent signal stability, making them ideal for during-exercise and daily continuous monitoring (Figure 34c).^803^ Ear-worn heart rate monitors are mainly integrated into wireless earphones or specialized ear-hook devices.^804^ Because the ear has rich capillaries and relatively small movement amplitude during daily activities, these devices provide signal stability significantly better than wrist monitoring, performing more reliably, especially during high-intensity exercise. The key advantage lies in seamless integration with daily audio equipment, offering a ’monitoring without awareness’ experience. Additionally, they can simultaneously track other health indicators such as blood oxygen saturation.^805–807^ A major concern of this type of device is long-term comfort, especially the risk of otitis externa after wearing the earphones.^808^

#### 6.3.8 Smart Clothing

Smart clothing with integrated heart rate sensors is designed to seamlessly blend with the skin, offering excellent comfort and wearability for long-term monitoring (Section 5.6).^809^ These garments conform to large skin areas, providing stable monitoring and distributed signal redundancy, making them ideal for all-weather health management and professional sports training (Figure 34b).^653^ By integration of ECG, PPG, and pressure sensors, smart clothing enables multimodal monitoring, delivering comprehensive cardiovascular health data.^810^ The larger sensing area also supports multipoint sensing,^811^ capturing heart rate data from different body parts and additional metrics like blood pressure. However, challenges remain in waterproofing, washability, and limited flexibility. Current solutions include washable electronic textiles such as conductive yarns, printed flexible circuits, and detachable modules.^653^

#### 6.3.9 Electronic Tattoos

Electronic tattoos or epidermal electronic heart rate sensors achieve perfect skin conformity with ultrathin flexible structures, and the ability to be worn continuously for days without detachment (Figure 34e).^749,812–814^ These devices can be used in medical monitoring and continuous health data collection, employing ultrathin flexible ECG electrodes to capture weak ECG signals or miniature pressure sensors to detect pulse waves.^749^ With thicknesses typically less than 10 μm and mechanical properties like or softer than skin (Young’s modulus below 100 kPa), these sensors provide an imperceptible adhesion experience, reducing dynamic noise and providing high-quality signals. These parameters offer practical advantages, including long-term wear (>24 h) without skin discomfort. The ultrathin transparent nature leads to improved user compliance. Additionally, they can be customized in shape and function for different body parts and monitoring needs. Recent research has introduced biodegradable materials, allowing the sensors to decompose after completing their monitoring period without manual removal.^815,816^ Due to direct contact with human skin, epidermal electronics require high levels of biocompatibility and stricter regulation before being applied for commercial use.

### 6.4 Temperature Sensors

Temperature sensing is essential for monitoring physiological states, managing diseases, and ensuring the reliability of integrated sensors. An important parameter is the core body temperature (CBT), as it reflects health conditions and indicates physical, biological, or environmental stress.^817^ An ideal CBT ranges between 36.5 and 37.5 °C, with fever being detected if it rises to more than 37.5 to 38.3 °C.^818^ When infected with a virus or bacteria, the human body heats itself up to increase the immune cell activity.^819^ Inflamed parts or a wound will have its temperature increased, while blood circulation passes this heat to the skin, which is dissipated by sweating. Hence, the skin temperature is also related to CBT. Various body locations can be employed for CBT measurements based on requirements, comfort, or feasibility. Figure 35A shows locations for invasive and noninvasive measurements on the human body. According to medical science guidelines, the temperature of the pulmonary artery is the most accurate location to measure CBT, but it is invasive.^820^ Oral, nasopharynx, esophagus, digestive track, urinary, and rectal are considered alternative invasive CBT locations.^821^ Oral use is the most practiced in daily routine due to its feasibility. In contrast, the noninvasive methods of skin temperature are not accurate for determining CBT, though they are still suitable for detecting major body temperature changes. The skin over the forehead is considered the most accurate, compared to the axilla and other skin locations.^822^ Figure 35B shows body temperature maps for a person, before and after heating with physical exercises. Distinct temperature distributions across the body from 32.5 to 36.5 °C are detected.^515^

**Figure 35:**
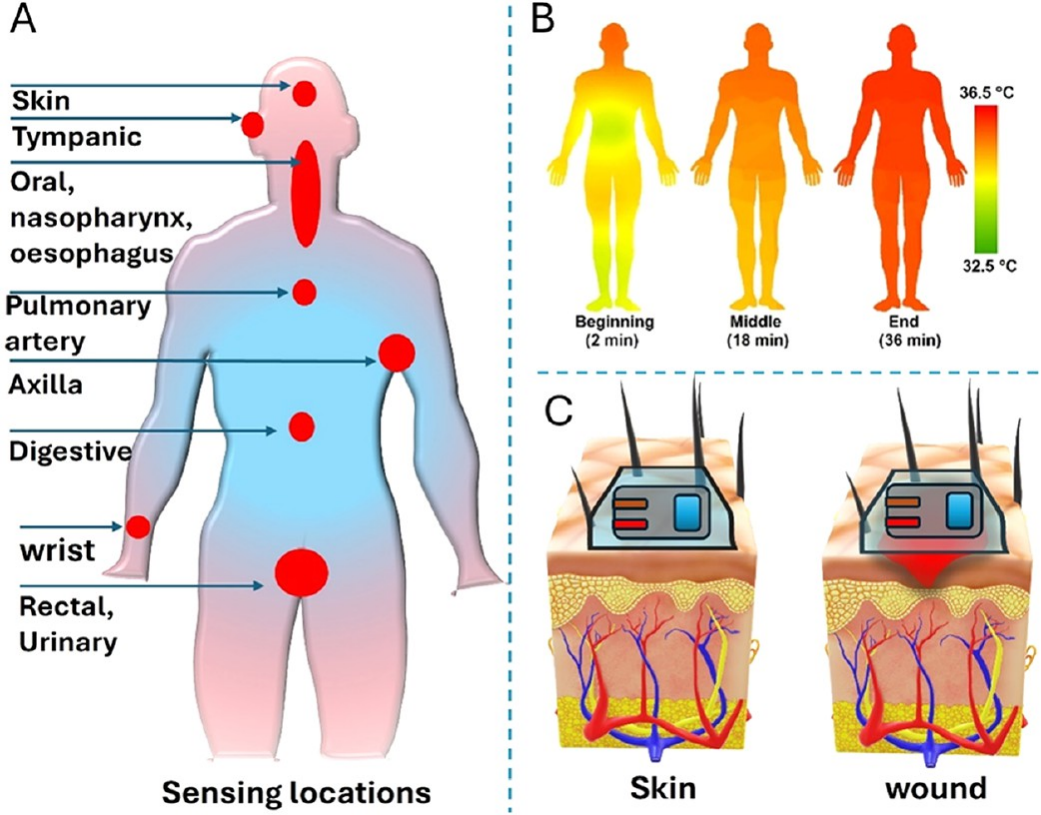
(A) Body locations for temperature sensing. Reproduced/adapted from Yu et al.^821^ Available under a CC BY 4.0 license. © 2024 John Wiley & Sons. (B) Body temporal map before, after, and during the exercise. Reproduced/adapted from Alam et al.^515^ © 2023 American Chemical Society. (C) Demonstration of integrated wearable T-sensor attached to skin and wound for continuous temperature monitoring.

Skin temperature provides a dynamic interface between the internal metabolic activity and external environmental conditions. Unlike CBT, which is relatively stable, the skin temperature fluctuates rapidly, reflecting blood flow, sweat evaporation, and thermal regulation processes. This variability makes skin temperature both a critical health marker and a parameter that must be carefully accounted for in wearable biosensors. Monitoring skin temperature enables the early detection of health issues. For example, fever, heat stress, or inflammation can manifest as changes in the peripheral temperature before other symptoms appear. Skin temperature monitoring is also valuable in chronic disease management, where conditions such as diabetes or vascular disorders influence local thermal profiles. Additionally, skin temperature is important for calibration, particularly for biosensors that are dependent on enzymatic reactions.

Continuous CBT monitoring has been performed for skin and wounds with wearable temperature sensors (T-sensors), as shown in Figure 35C.^823^ Such sensors are integrated with the Internet of Things (IoT) to collect long-term temperature profiles of patients and notifications to physicians for on-time and continuous treatments.^824,825^ To improve the safety standards, attempts were made to integrate sensors with energy storage device patches.^825^ Wearable T-sensors are now commercially available, with the heat response transformed into electronic or optical signals through capacitive, resistive/thermistor, thermoelectric, optical, pyroelectric, and colorimetric processes.^826–831^ Materials used in these sensors include metals and metal-oxide materials, semiconducting polymers, carbon-based materials/graphene, 2D materials, hydrogels, ionogels, and hybrid composites.^832–837^ The sensing units must be flexible, stretchable, and mechanically stable, thus requiring suitable substrates. The latter may be polyimide, PDMS, polyethylene naphthalate, thermoplastic polyurethane, textiles, and cellulose-based substrates.^838–841^

Figure 36 summarizes recent reports of materials engineering to enable flexible wearable T-sensors with different sensing mechanisms. Hu et al. utilized thermotropic composites made with poly­(ethylene oxide) (PEO)/PVDF/H_3_PO_4_ as a dielectric layer in capacitive T-sensors.^842^ PEO acted as a thermotropic dopant to induce ion dissociation and affect the electric double-layer capacitance when the temperature changed, as depicted in Figure 36Ai. This doping strategy leads to highly disordered PEO, thus hampering device performance. Using two-dimensional square holes shown in Figure 36Aii helped to obtain ordered PEO, with which reproducible sensors could be achieved with reduced error performance (<2.2%). Figure 36Aiii-v illustrates the high performance for the flexible sensors shown in Figure 36Avi, which had a resolution of 0.05 °C and response speed (<12 s) within 35–43 °C. Resistive sensors are also widely employed, especially with graphene. Zhang et al. produced a wearable T-sensor with polydopamine/graphene composite (PDA@rGO) with an eggshell membrane (ESM) to ensure flexibility and breathability.^843^ Figure 36Bi shows the heat simulation of ESM with and without PDA@rGO on skin (37 °C), which confirms the high thermal conductivity sensing contribution from the composite. The resistive sensing mechanism and resistance change over 30 to 42 °C are described in Figure 36Bii,iii. Upon introducing PDA and rGO, the temperature coefficient of the resistance increases. The sensing response and stability at various temperatures are depicted in Figure 36Biv,v, with the best T-sensor exhibiting high sensitivity and resolution, good linearity, and rapid response (4–8 s).

**Figure 36:**
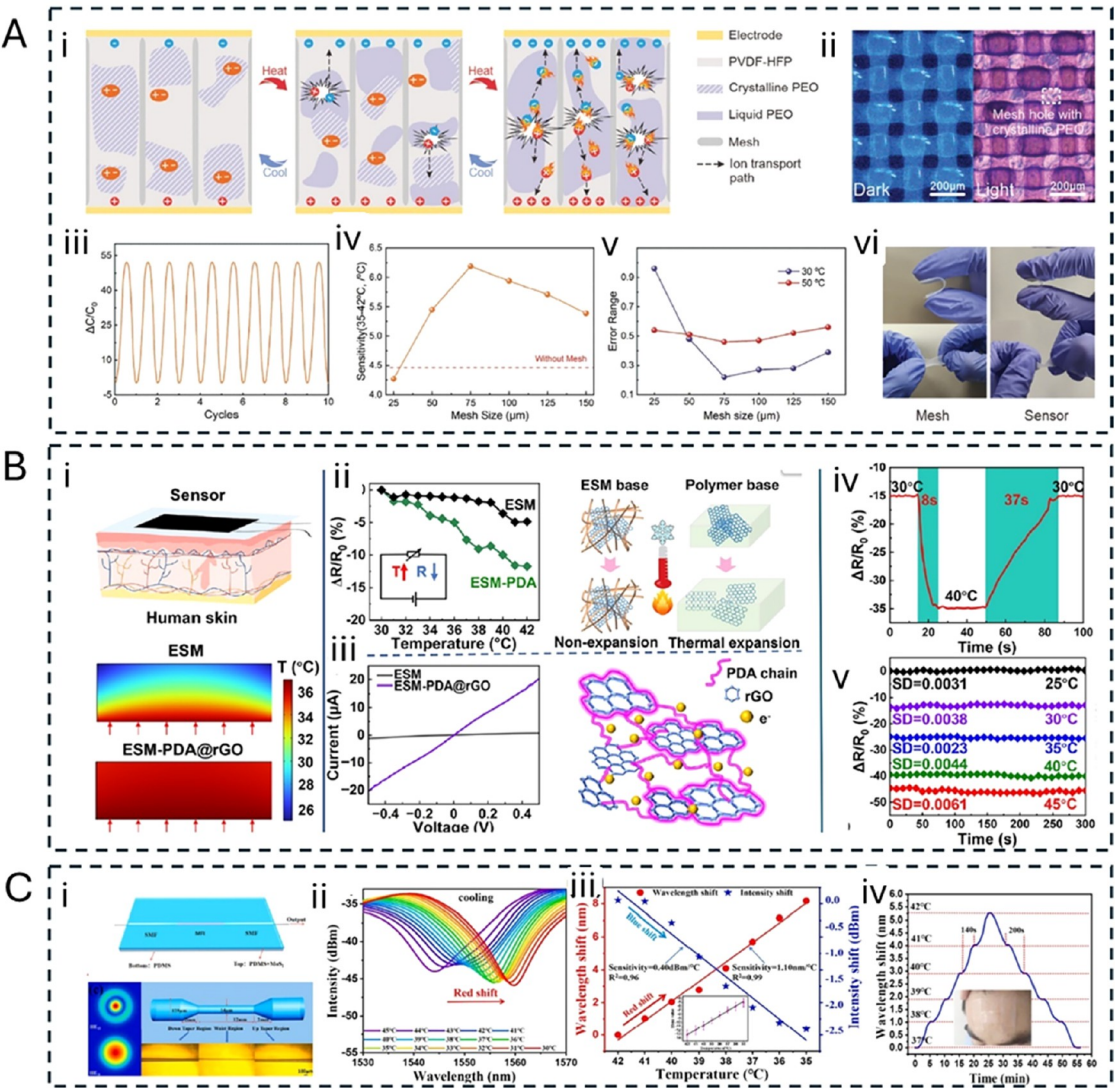
Advances in T-sensing device strategies based on sensing mechanism and materials engineering. (A) (i, ii) Effect of cooling/heating on PEO domains doped in PVDF capacitive layer with two-dimensional square hole structure and corresponding micrograph. (iii) Change in capacitance during cooling/heating cycles. (iv, v) Effect of different mesh sizes on repeatability and sensitivity. (vi) Flexibility of the capacitive layer of T-sensor. Printed with permission from Hu et al.^842^ © 2024 John Wiley & Sons. (B) (i) Schematic illustration of resistive T-sensor attached to the human skin and effect of PDA@rGO in thermal properties of ESM substrate simulation by finite elemental analysis. (ii, iii) Resistance and I–V response of bare ESM and doped ESM and corresponding structural changes in conductive pathways in the presence of graphene and PDA polymer during heat sensing. (iv, v) Response time of T-sensor from 30 to 40 °C and continuous response stability with five different temperature settings for 300 s. Modified from Zhang et al.^843^ © 2023 American Chemical Society. (C) (i) Wearable microfiber T-sensor described with PDMS bottom and PDMS/MoS_2_ layer encapsulation. The detailed structure including optical microscope images. (ii, iii) Recorded spectrum and according shift of spectrum at different temperatures and linearity analysis. (iv) Response of T-sensor at forehead. Printed with permission from Wang et al.^844^ © 2024 Elsevier.

Wearable optical T-sensors allow for continuous monitoring.^830^ These can be achieved, for example, by using optical fibers whose optical properties depend on the temperature. These sensors are immune to electromagnetic interference, which makes them robust and accurate.^845^ Figure 36Ci shows a T-sensor encapsulated in PDMS and MoS_2_ with a smaller diameter in the middle to provide a mismatch to the optical path.^844^ When tested between 30 and 45 °C, the T-sensor spectrum red-shifted with increasing temperature (Figure 36Cii). The sensitivity was 1.10 nm/°C with linearity approaching 0.99. The blue-shift analysis also achieved a linearity of 0.96 in Figure 36Ciii. The demonstration of a wearable sensor on the forehead was made to monitor temperature from 37 to 42 °C, which matches the performance of commercial mercury T-sensors (Figure 36Civ). With its fiber shape, low density, and high strength, this T-sensor can be integrated into wearable patches and facemasks to serve for oral temperature monitoring.

The fabrication process for a strain-insensitive wearable T-sensor is shown in Figure 37Ai.^846^ The sensor was obtained by direct-ink-writing and three-dimensional printing of PEDOT:PSS and graphene ink on poly­(vinyl alcohol). A polygonal design allowed for a strain-insensitive measurement of the temperature, as indicated in Figure 37Aii and Figure 37Aiii. The sensitivity was 0.01 °C^–1^ in the range of 20–50 °C, and 0.001 °C^–1^ in the range of 55–75 °C. Other designs, such as those obtained with micropatterning and kirigami approaches, are efficient in achieving flexible T-sensors.^847^ Furthermore, these sensors can also be based on thermoelectric, piezoelectric, and colorimetric principles of detection.^848,849^

**Figure 37:**
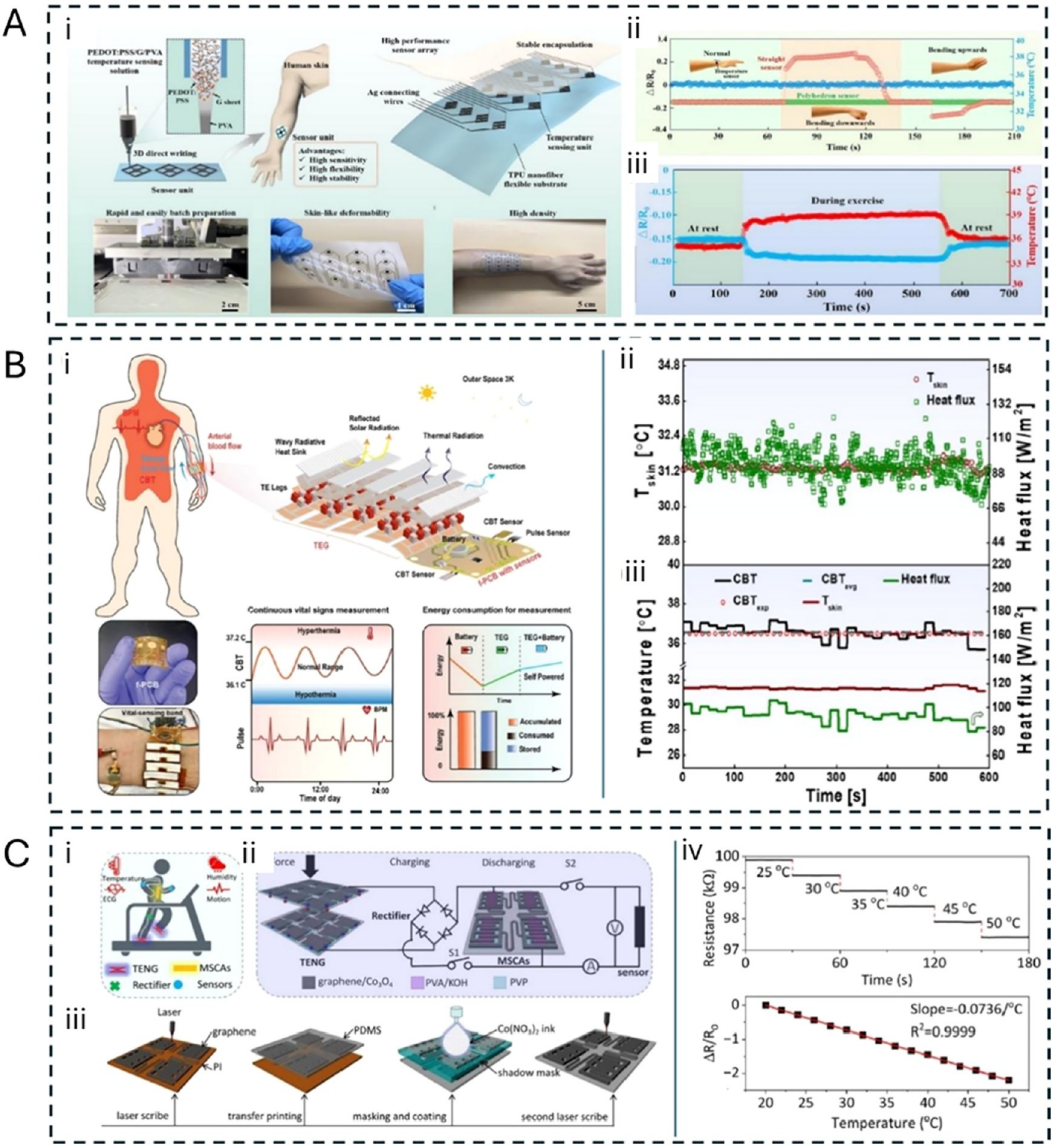
Advanced strategies for flexible designs and integrated self-powered T-sensors. (A) (i) Schematic diagram of the T-sensor unit by the 3D direct writing method, polygonal design, components, and its flexibility, (ii) T-sensing performance at different strain conditions. (iii) T-sensing performance while rest and exercise. Reproduced with permission from Wang et al.^846^ © 2024 Elsevier. (B) (i) Schematic of the compact band for continuous monitoring of temperature on the forearm with a schematic of T-sensor with TEG self-powered unit. The integration components, demonstration of signal measurement and power management. (ii) Sensing performance of T-sensor mounted on forearm. (iii) Calculated and average CBT determination. Modified from Khan et al.^850^ © 2024 American Chemical Society. (C) (i) Concept of integrated health monitoring incorporating T-sensor with self-powered stretchable power unit. (ii) Schematic of sensor unit on laser-induced graphene substrate, TENG with T-sensor and system working mechanism. (iii) Step-by-step nanofabrication of sensor components. (iv) Dynamic sensing response and calibration plot of the T-sensor. Modified from Ding et al.^851^ © 2024 American Chemical Society.

Self-powered T-sensors were produced with the incorporation of a thermoelectric generator (TEG) and a battery, as shown in Figure 37Bi (see Section 10 for more details on self-powered devices).^850^ The powering unit was connected to a commercial T-sensor mounted on a flexible printed circuit board, targeted to monitor CBT on the forearm based on arterial blood heat reaching the skin. In this setup, the human volunteer was stabilized in a temperature-controlled room (25 °C) to prevent thermoregulation. Figure 37Bii,iii shows the heat flux and an average skin temperature of 31.3 °C for the volunteer measured for 600 s. The calculated and expected CBT were 36.55 and 36.50 °C, respectively. Another integrated self-powered T-sensor is shown in Figure 37Ci, made with graphene/Co_3_O_4_ nanocomposite. Figure 37Cii describes the strategy of TENG for power generation, micro supercapacitors for storage and power supply, and the T-sensor for CBT monitoring.^851^ The ink printing process is depicted in Figure 37Ciii, while the sensing response with a sensitivity of 0.074% °C^–1^ and a LOD of 0.042 °C is shown in Figure 37Civ.

Most integrated systems utilize thermosensitive sensors that may be affected by changes in the strain or humidity. This limitation was overcome with the development of a fiber-based flexible T-sensor in a smart textile.^852^ A wireless closed-loop system was employed for decoupled multimodal health monitoring (T-sensing and human pulses) and personalized thermoregulation for precise pulse measurements. The T-sensor made with rGO/MnO_2_/Kevlar/TPU (thermoplastic polyurethane) is illustrated in Figure 38a. The measurements of pulse waves and temperature with the T-sensor of Figure 38b are shown in Figure 38c–e. The application of the system on the leg of two volunteers monitored wirelessly with mobile phones is depicted in Figure 38f–k. This type of T-sensing platform can be extended to multiple healthcare applications such as geriatrics, postpartum care, rehabilitation, and sports, as shown in Figure 38l. Other advances allow for monitoring of wound healing and ongoing treatments such as the diagnosis of kidney necrosis.^853,854^

**Figure 38:**
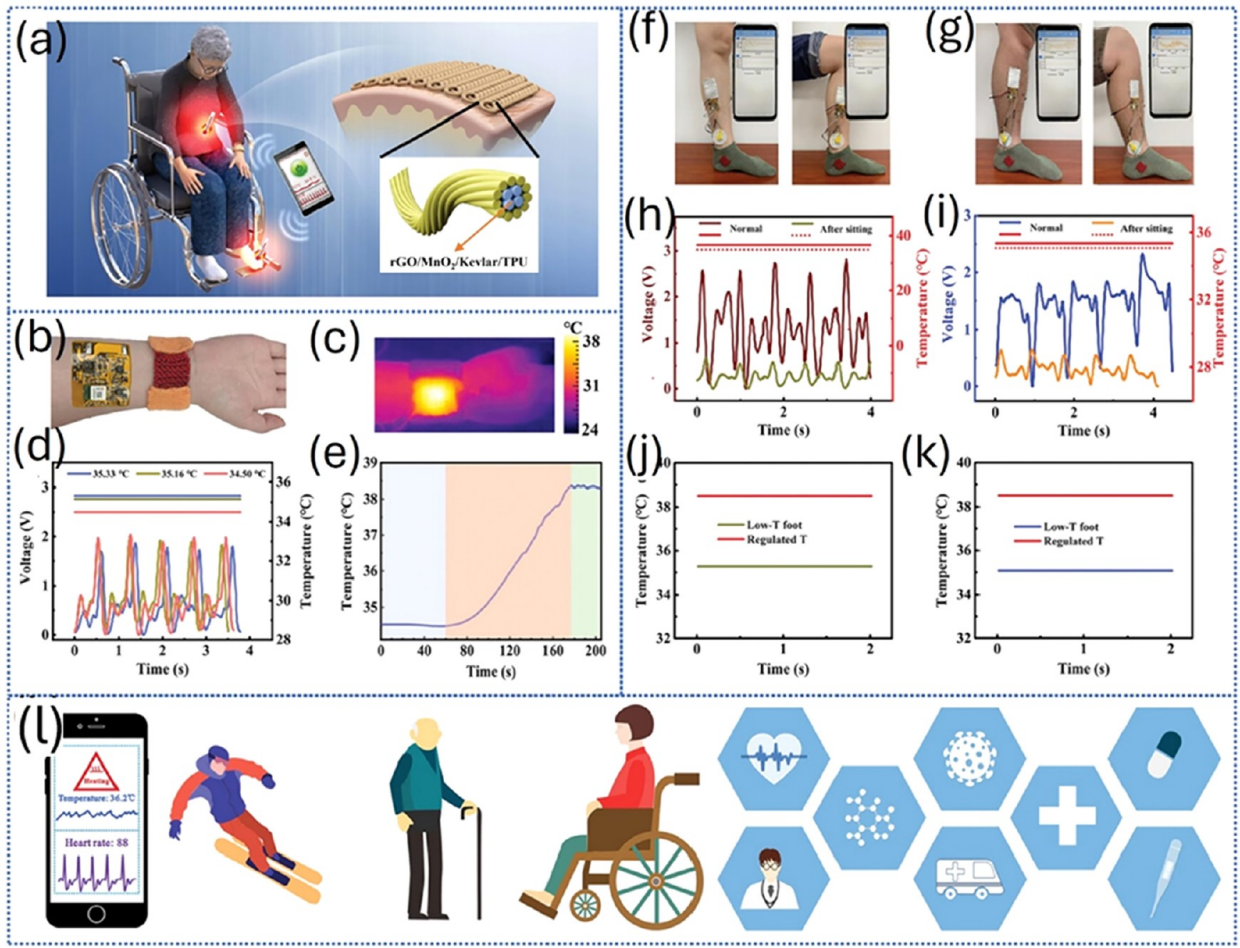
(a) Application of T-sensor integrated in smart textile for handicap patient’s health monitoring and thermal management. (b) Mounted smart textile band and PCB on forehand. (c) Infrared image of thermal regulation by heating textile. (d, e) Measured body temperatures and pulse waveforms simultaneously and their minimized cross effects. (f, g) Validation of closed-loop system on the leg of two volunteers, with measurements of temperature and pulse wave wirelessly on mobile phones. (h, i) The recorded typical body temperature and pulse waveform of person 1 and person 2. (j, k) Thermoregulated temperature by the system. (l) Possible applications of the smart-textile-based wireless closed-loop system. Reproduced/adapted with permission from Zhang et al.^852^ © 2024 John Wiley & Sons.

Wei Gao and collaborators demonstrated how integrating temperature sensing into sweat-based platforms improved the reliability of biomarker detection by accounting for temperature-dependent aptamer sensors.^855^ These wearable devices employ various types of temperature sensors including thermistors, resistive temperature devices (RTDs), and IDEs. Thermistors, with their negative temperature coefficient (NTC) behavior, are widely used because of their simplicity and high sensitivity. However, their nonlinear resistance–temperature relationship can complicate calibration. IDEs leverage capacitance or resistance changes in response to the temperature and are often used in multifunctional sensor platforms. RTDs, such as silver- or graphene-based devices, provide precise temperature measurements with adaptable designs, making them suitable for flexible and wearable applications.

Maroli et al.^856^ proposed the approach depicted in Figure 39 to wearable temperature sensing through an RTD using rGO. These resistors are characterized by their linear negative temperature coefficient, distinguishing them from conventional thermistors that exhibit a nonlinear response. The linearity is a significant advantage for wearable systems, simplifying calibration and ensuring more consistent temperature measurements across a wide operational range. The design optimizes the sensing area while maintaining a compact form factor, enhancing its sensitivity to thermal changes. The linearity of the rGO resistors enables their combination with other materials, such as silver-based resistors, to create composite systems that do not experience changes in their resistance value with temperature. The combination of these materials yielded a resistor whose value exhibited a variation of less than 0.22%, falling within the instrument’s specified error range, across a temperature range of 40 to 100 °C. By leveraging the differing thermal coefficients of these materials, a methodology for compensating for temperature fluctuations was demonstrated, thereby ensuring stable and reliable sensor performance even in dynamic environments. In the latter work, inkjet printing was employed to fabricate the silver-based meander resistors, thereby enabling the precise deposition of conductive materials onto flexible substrates. This method is conducive to scalability and cost-effectiveness, rendering it an optimal choice for wearable technologies. As reported by Scroccarello et al.,^857^ the rGO sensor was fabricated by laser-induced reduction of graphene oxide and then transferred to the substrate using a press. The temperature values obtained with both sensors were found to be in good agreement with those observed with the thermal camera.

**Figure 39:**
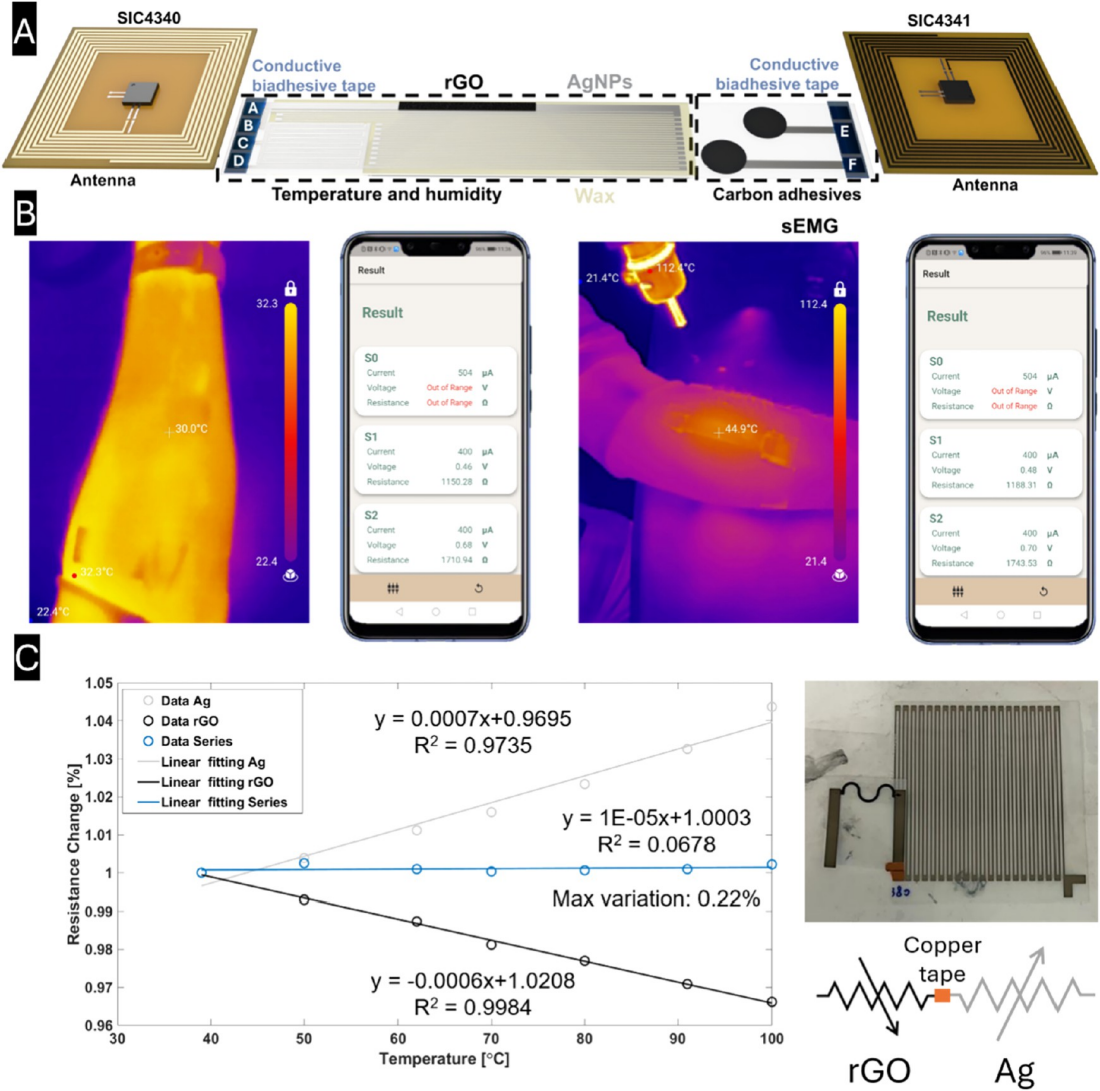
(A) Schematic of the sensor network illustrates the configuration of the rGO temperature sensor, which is connected between pads A and B. As the temperature increases, the resistance value of the sensor decreases. In contrast, the silver sensor between pads B and D exhibits an increase in resistance value with rising temperature. Additionally, a humidity sensor, comprising a silver interdigitated electrodes, is positioned between pads C and D. This sensor assesses the alteration in conductivity of the substrate. Furthermore, the electrodes utilized for motion and seizure detection are situated between pads E and F. (B) Thermal images of the inkjet-printed device applied to the skin, captured at room temperature and during hot air exposure for calibration purposes. (C) The resulting series data for both resistors connected. The calibration curves comparing the response of the silver nanoparticle meander resistor and the rGO serpentine resistor. Reprinted with permission from Maroli et al.^858^ © 2024 Elsevier.

In summary, T-sensors are essential tools for wearable healthcare monitoring, with the area having advanced significantly over the past few years. One of the main potential challenges in developing such devices is the moderate accuracy of measuring CBT on the skin, which is often not as accurate as in invasive body locations. The skin temperature may be impacted by sweating and the outer environment temperature or humidity, which reduce the effectiveness of measured signals. Considering health monitoring, T-sensors also need high-end integration with electronics, IoT, and energy storage devices. This integration results in high costs due to the use of nanofabrication techniques and compact prototyping. Further, battery-utilizing devices bring health or safety hazards, whereas safe and biocompatible aqueous energy storage devices are power-deficient. As demonstrated, self-powered T-sensing modules are quite space-consuming as a unit and very specific in their body location for CBT sensing. The use of electronic components brings electromagnetic radiation interference and radio frequency emission, which are considerable challenges. Hence, further research should focus on developing hazard-free materials, safe energy storage methodologies, low-cost engineering, and devices with low-radiation interference.

## 7 Design of Wearable Devices for Healthcare Applications

### 7.1 Adhesion of Wearable Sensors to Human Skin

The functionality of many wearable sensors depends largely on the stable adhesion between the device and the target surface (e.g., the human skin).^859–862^ Without tailored skin-sensor interfaces, wearable sensors and devices are prone to motion artifacts, compromising reliable signal acquisition and user satisfaction (Figure 40).^863–865^ Secure device fixation is the primary motivation because even minor sensor displacements can introduce significant noise into physiological measurements and continuous monitoring.^866,867^ Achieving stable adhesion requires careful consideration of several key aspects. First, conformability, the ability of the adhesive layer to follow the complex topography of the skin, is essential for maintaining intimate contact and minimizing interfacial impedance. Second, biocompatibility is required to prevent adverse skin reactions, including irritation, allergic responses, or damage during removal. Third, interference must be minimized; the adhesive should not introduce mechanical or electrical disturbances that compromise signal quality. Finally, exposure to sweat, water, temperature fluctuations, desquamation, and dynamic strains can substantially degrade adhesive performance.

**Figure 40:**
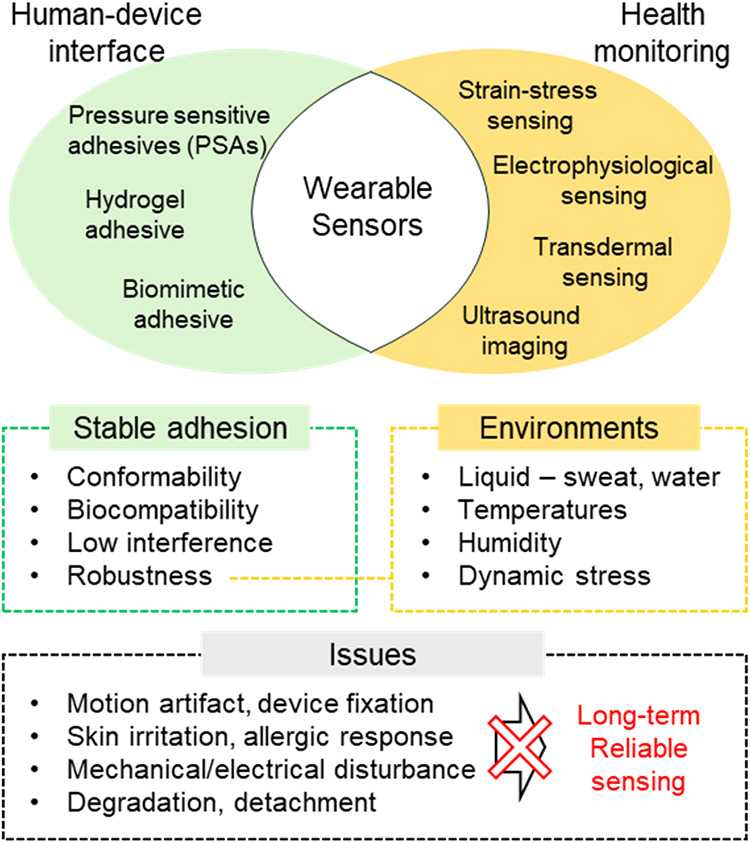
Schematic illustration of the relationship between adhesive types, wearable device functions, and requirements for stable adhesion for reliable sensing.

Various material strategies have been explored to address these requirements. Hydrogel adhesives provide excellent adhesion and biocompatibility, but challenges remain regarding their mechanical durability and degradation under harsh environmental conditions. Dry adhesives, including medical pressure-sensitive adhesives (PSAs), offer reversible adhesion and are particularly suited for applications that require repeated attachment and detachment. Self-adhesive and ultrathin sensors enable intimate skin contact, allowing for seamless skin integration.

### 7.2 Conformal Contact of Wearable Devices on Substrates

Conformal contact refers to the intimate, gap-free interface between a device and a complex, dynamic skin surface.^868^ This attribute is essential for reducing skin-electrode impedance, minimizing motion artifacts, enhancing signal fidelity, and improving the user comfort. A recurring challenge in wearable bioelectronics arises from the mechanical mismatch between soft, curvilinear biological surfaces and the typically planar, rigid, or semiflexible materials used in sensors.^864,868^ Dynamic skin deformation during daily activities, combined with the presence of sweat, hair, and sebaceous secretions, can disrupt electrode-skin interfaces, resulting in increased impedance, baseline drift, and signal degradation. Therefore, strategies to enhance conformal contact are needed for advancing the functionality and reliability of the wearable sensors.

Conventional metal electrodes are an example of devices that exhibit limited stretchability and often fail to conform to dynamic deformations of the skin. This mechanical sensor-skin incompatibility can lead to localized stress concentration, promoting gradual microscale separation that ultimately progresses into permanent detachment under repeated deformations (Figure 41A).^869^ On the other hand, hydrogel materials and interfaces can achieve conformal contact with skin by absorbing moisture and swelling, allowing them to fill microscale gaps (Figure 41B).^870^ This swelling-induced adaptation improves adhesion and reduces interfacial impedance, enhancing the mechanical stability and electrical performance of wearable sensors. Hydrogels with elastic properties matching those of skin offer a soft, compliant interface that maintains intimate contact even on irregular or dynamic skin surfaces. Among recent examples, Shin et al. developed an ultrathin polymeric conductive adhesive (PPd) electrode by incorporating PVA and d-sorbitol into PEDOT:PSS to modulate elasticity and adhesion.^869^ The PPd electrode minimized motion artifacts and connector-induced noise, ensuring stable signal acquisition under dynamic conditions during walking and running (Figure 41C).^869^

**Figure 41:**
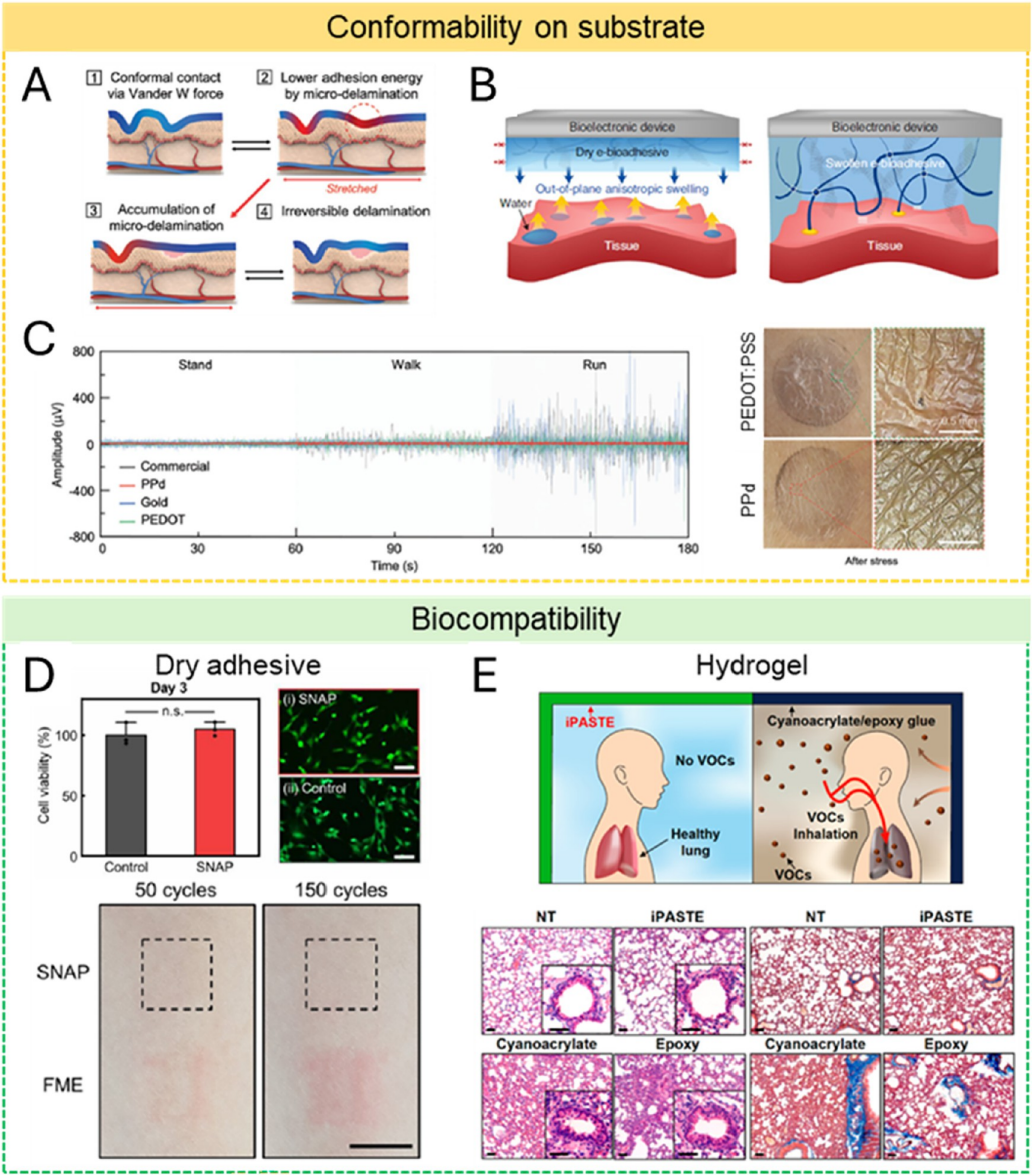
(A) Mechanism of progressive delamination of the electrode from the skin. Reprinted with permission from Shin et al.^869^ © 2024 John Wiley & Sons. (B) Hydrogel adhesive interface capable of providing anisotropic out-of-plane swelling, rapid, and robust adhesion on wet tissues. Reprinted with permission from Deng et al.^870^ © 2020 Springer Nature. (C) Comparison of RMS noise values between commercial (black), PPd (red), Au (blue), and pristine PEDOT:PSS (green) electrodes and EMG signals during standing, walking, and running. Reprinted with permission from Shin et al.^869^ © 2024 John Wiley & Sons. (D) Comparison of the cell viability between the control group and the experimental group. Reproduced from Kim et al.^871^ Available under a CC BY-NC license. © 2024 American Association for the Advancement of Science. (E) (Top) Schematic of surface-coating with/without causing VOCs and (bottom) lung histopathology analysis of mice after 21 days of exposure to adhesives. Hematoxylin/eosin staining and Masson’s trichrome staining. Reproduced from Choi et al.^872^ © 2022 American Chemical Society.

A similar focus on adhesion and mechanical compatibility is reflected in the work by Zhang et al., who developed a ∼10-μm-thick mesh-reinforced hydrogel sensor for continuous wearable bioelectronics.^873^ The ultrathin geometry of the hydrogel minimized interfacial air gaps, thus reducing skin-electrode impedance and improving the signal-to-noise ratio. The role of hydrogel interfaces was further exemplified by Deng et al., who introduced an electrical hydrogel adhesive match by utilizing a graphene nanocomposite hydrogel layer.^870^ The adhesive formed robust, electrically conductive bonding with wet dynamic tissues, overcoming the limitations of van der Waals-based attachment or suturing. Kim et al. addressed conformal contact through multiscale physical adhesion mechanisms by creating an electrostatic-mechanical synergistic elastomer-hydrogel composite.^874^ Inspired by natural suction-based adhesion, these structures combined capillary and hydrogen-bonding interactions to achieve strong adhesion on wet tissues.

Overall, diverse strategies in achieving conformal contact—including ultrathin geometries, adhesive and hydrogel adhesive, and multiscale microstructures—demonstrate converging solutions toward this goal across multiple applications for long-term, high-fidelity health monitoring in a dynamic environment.

### 7.3 Biocompatibility Issues

Interface materials, such as adhesives, influence the physiological interaction with tissues, with biocompatibility being determinant for device safety.^860^ Ensuring biocompatibility in wearable sensors requires careful selection of materials that minimize adverse biological responses.^875^ Adhesive and conductive components must be engineered to maintain skin safety while providing stable mechanical and electrical performance. In addition, the device’s structural design, including penetration depth and interface mechanics, should align with the skin properties. Choi et al. demonstrated that the gel adhesives exhibits excellent biocompatibility by eliminating the release of VOCs, which are common causes of skin, eye, and respiratory irritation (Figure 41E).^872^ These VOC-free materials minimize potential toxicity and irritation during prolonged skin contact. For epidermal electrophysiological monitoring, Kim et al. have developed a stretchable microneedle adhesive patch utilizing an electrically conductive adhesive composed of silver flakes and silicone (Figure 41D).^871^ The materials exhibited no cytotoxic effects, confirming their intrinsic biocompatibility for skin contact applications. Microneedles penetrate only the stratum corneum without reaching pain receptors, minimizing irritation.

In implantable devices, Wu et al. provided evidence that an adhesive interface can prevent fibrous capsule formation by reducing the level of inflammatory cell infiltration.^860^ These adhesive layers achieved conformal integration with diverse organ surfaces, eliminating observable fibrosis over 12 weeks and serving as active mediators of immune tolerance. Overall, designing adhesives for wearable bioelectronics requires a delicate balance of adhesion strength, mechanical compliance, electrical conductivity, and biological tolerance for clinical, daily, and implantable applications.

### 7.4 Robustness under Various Environmental Conditions

Wearable biosensors must withstand a variety of conditions to ensure constant robust and reliable measurements. Biofluid accumulation, temperature changes, the presence of water and applied stress are some of the challenges. Sweat accumulation at the skin–sensor interface is critical, as it increases interfacial impedance, degrades adhesion, and introduces noise and signal drift as well as sweat-induced discomfort and skin irritation.^873,876^ To address these problems, Zhang et al. considered sweat removal by developing a three-dimensional liquid diode structure that actively transports sweat away from the skin through directional liquid transport channels under heavy sweating.^876^ Using a different approach, Wang et al. introduced a Janus membrane-based pH sensor combining a hydrophobic porous substrate with a hydrophilic nanofiber layer to achieve simultaneous sweat absorption, gas permeability, and self-adhesion.^877^ This configuration allowed reliable pH monitoring for over 7 h without detachment or discomfort. Lin et al. incorporated gel-free soft encapsulation and anisotropic conductive films into a wearable ultrasound system to maintain adhesion and coupling in moist environments without relying on traditional coupling gels.^878^ This design supported continuous deep tissue monitoring while minimizing moisture interference.

Regarding water resistance, Yu et al. developed a water-resistant ionogel electrode for underwater ambulatory ECG monitoring, enabling stable physiological signal detection in aquatic environments (Figure 42A). The ionogel achieves underwater adhesion by disrupting the hydration layer through its hydrophobic polymer network, allowing strong interfacial interactions even in wet conditions. Xiang et al. also created water-resistant devices, introducing a multifunctional conductive hydrogel combining dipole–dipole and hydrogen bonding interactions for underwater sensing.^879^ Their hydrogel material maintained stable adhesion, high conductivity, and EEG signal quality even under aquatic immersion, demonstrating robust wet-environment compatibility. As for dry-adhesive materials, Zhou et al. designed a bioinspired adhesive fabric by embedding LM beads within a PLA fiber network to enable reversible adhesion and photothermal responsiveness while maintaining strong bonding and excellent stability in humid or wet environments.^880^

**Figure 42:**
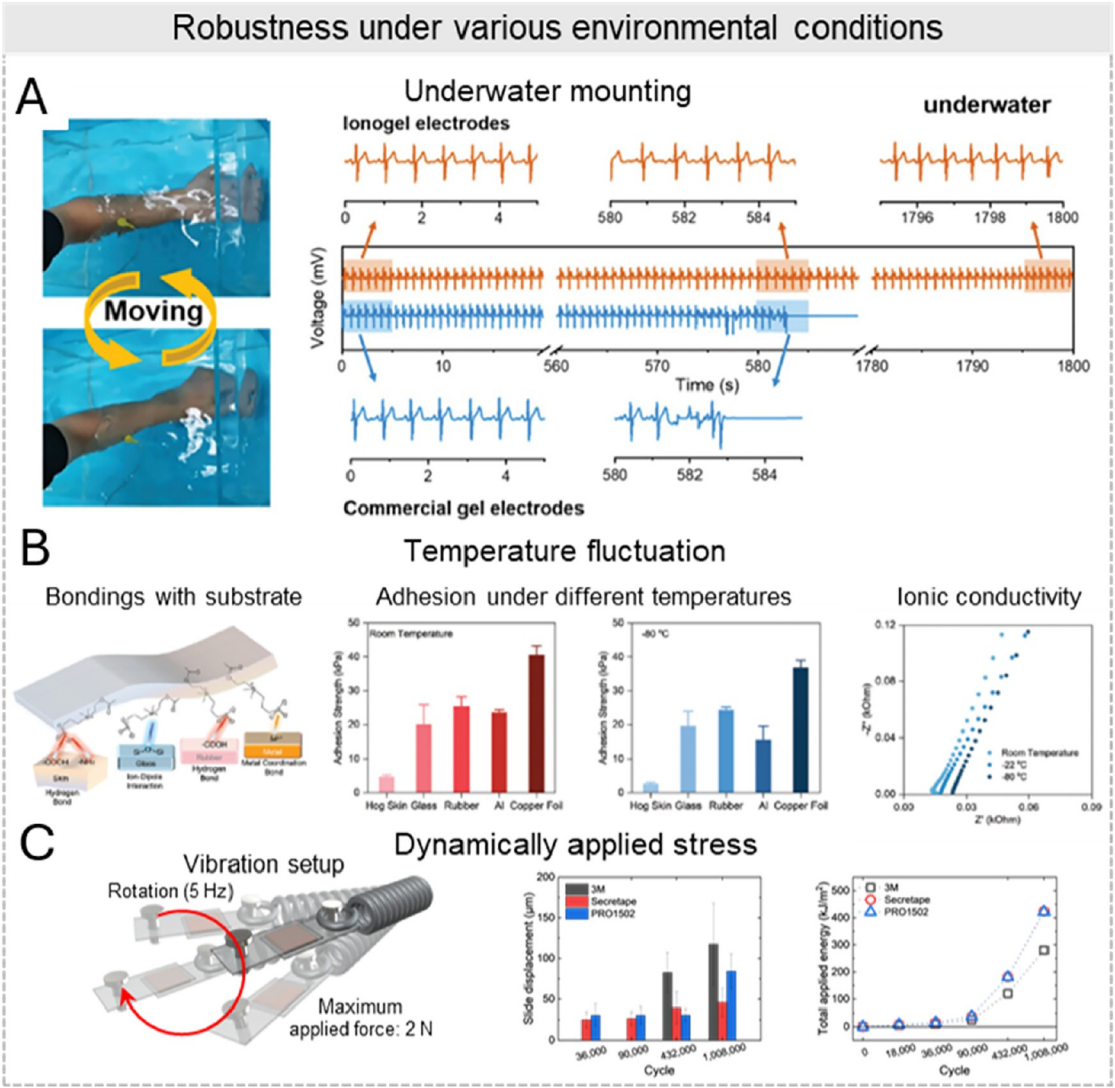
(A) Long-term stability of ECG signal of ionogel electrode in the aquatic environment. Reproduced with permission from Yu et al.^884^ © 2021 John Wiley & Sons. (B) Comparison of hydrogel and organo-hydrogel after 24 h exposure to various environments and relative resistance responses of organo-hydrogel under different temperatures. Reproduced from Han et al.^881^ Available under a CC BY 4.0 license. © 2025 John Wiley & Sons. (C) Stable adhesion of PSAs over million cycles of loading. Reproduced from Jeon et al.^885^ Available under a CC BY 4.0 license. © 2024 American Chemical Society.

Adhesives must maintain conformal contact, adhesion strength, and functional integrity despite environmental extremes such as freezing or heat, to ensure continuous signal acquisition and user comfort. For these applications, Han et al. developed an ionic conductive hydrogel that maintains strong adhesion even at −80 °C, enabled by robust dipole–dipole interactions, hydrogen bonding, and ionic bonding with various substrates (Figure 42B).^881^ This stable adhesion under extreme cold ensures continuous skin contact, preventing signal degradation and device detachment. Shang et al. developed a mussel-inspired organo-hydrogel with robust wet adhesion and environmental tolerance ranging from −40 to 60 °C.^882^ The adhesive layer combined catechol chemistry and hydrophobic interactions to prevent adhesion failure. In another example, Zhou et al. reported glutinous-rice-inspired adhesives for antifreezing and moisture retention for 90 days.^883^

Finally, stable adhesion under dynamically applied stress also must be considered. Jeon et al. evaluated the vibration stability of double-sided PSAs under cyclic circular loading at 5 Hz and 2 N for up to one million cycles to mimic complex human body motions (Figure 42C).^885^ The PSAs exhibited minimal displacement without adhesive failure, maintaining mechanical integrity throughout the test. These findings demonstrate the long-term mechanical durability and reliable adhesion performance of PSAs for wearable devices in dynamic vibrational environments. Furthermore, Wang et al. achieved vibration-resistant adhesion by integrating mushroom-shaped microtips, mechanically isolated adhesive units, and a porous backing layer.^886^ This multiscale design enabled stress decoupling and energy absorption, ensuring stable adhesion under dynamic conditions.

In summary, the adhesive interfaces for wearable sensors are critical for maintaining a stable device performance under practical conditions. For sweat management, permeable structures or ultrathin adhesive layers are employed to enhance water vapor transmission and minimize moisture accumulation at the skin interface. In underwater applications, hydrogel-based adhesives provide reliable adhesion by retaining interfacial bonding even in wet conditions while also offering mechanical compliance. Similarly, hydrogels can maintain adhesion across a wide temperature range, ensuring conformal contact under limited moisture thermal variations, but can suffer from decreased electrical or mechanical performance over time. Dry adhesives may exhibit reduced strength under high humidity or heat, which is critical for maintaining a high sensitivity. Therefore, future strategies must address this trade-off by integrating materials and designs that simultaneously ensure robust adhesion and functional stability across a diverse range of environments.

## 8 Soft Electronics

This topic is essential for wearable sensors since many applications involve embedded electronics, which should preferably be produced with soft electronics for seamless integration across on-body and in-body platforms. The field of soft electronics emerged from the transition from early electronics with inflexible integrated circuits (ICs) fabricated on glass or wafer substrates^887^ to organic semiconductors, plastic substrates, and printing techniques.^888–890^ Softness and flexibility are insufficient, however, for applications that require interfaces with living organisms. In such cases, electronics must also stretch, as defined by elastic responses to large strain deformations associated with non-Gaussian curvatures. These opportunities motivated research on materials and approaches to platforms that can accommodate uniaxial and multiaxial strains often exceeding 50% without compromising electrical performance.^891^ Successful outcomes of these early efforts are in bioinspired, including hemispherical “electronic eye” cameras^892^ and skin-like, or “epidermal”, sensor systems.^893^ These devices retain function under complex deformations, enabling reliable operation on dynamically moving and deforming surfaces.

As shown in Section 7, to achieve accurate signal acquisition in wearable sensors, conformal contact between the electrode and the tissue is required, as signal quality diminishes with increasing distance between them (Figure 43). According to Coulomb’s law, any geometric gap reduces the electric field’s intensity significantly. Thus, minimizing this gap through the selection of appropriate materials and fabrication methods is vital for achieving stable and effective bioelectronic performance. Furthermore, the intimate contact between the electrode and tissue can enhance the capacitance significantly, favoring the resistive equivalent circuit and shifting the phenomena in the interface from capacitive- to ohmic-dominated (simplified equivalent circuit models can be seen in Figure 43). Such an aspect also contributes to lowering the impedance of the system, potentially improving device performance. Electrodes should also conform to soft biological tissues without causing damage. Rigid components can induce inflammatory responses and scarring, leading to the migration and accumulation of astrocytes and microglia around the electrodes. This forms a 50–200 μm thick sheath, separating the recording site from the target tissue.^894–896^

**Figure 43:**
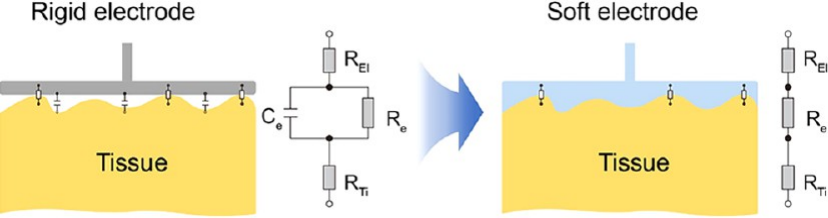
Impedance comparison between rigid and soft electrodes interfacing with tissues, along with their corresponding equivalent circuit models. *R*_El_: Electrode resistance, *R*_Ti_: Tissue resistance, *R*_e_: Interfacial resistance, *C*_e_: Interfacial capacitance.

Soft and stretchable electrodes can be fabricated by using two primary strategies: (1) engineering rigid materials into geometrically deformable structures or (2) utilizing intrinsically soft and stretchable materials combined with conductive fillers. Soft and stretchable electrodes achieve mechanical compliance through structurally deformable designs, allowing conducting materials to stretch while maintaining an electrical performance. In contrast, intrinsically soft and stretchable electrodes leverage elastic polymers mixed with inherently soft conductive fillers, such as metal nanomaterials, LMs, and organic conductive materials. Their mechanical compliance can be further enhanced through additional structural and polymer engineering, as depicted in Figure 44A.

**Figure 44:**
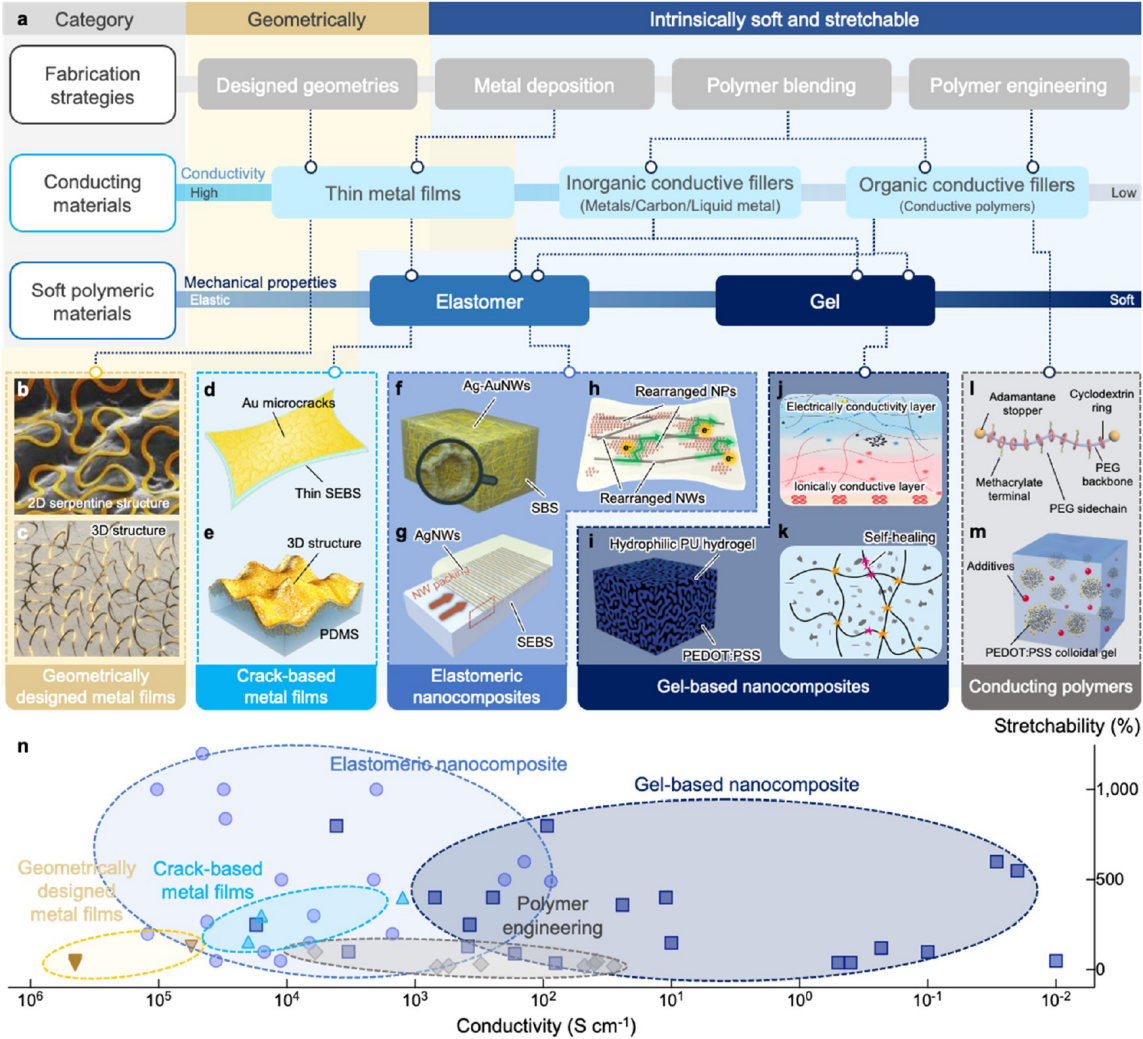
Materials and fabrication strategies for nanomaterials-based stretchable electrodes. (a) Schematic diagram illustrating various materials and fabrication strategies. (b, c) Stretchable electrodes using specially designed geometries. (d, e) Stretchable electrodes using cracked metal thin films. Intrinsically stretchable electrodes by blending conductive fillers and (f–h) elastomer, (i–k) gel, and (l, m) intrinsically stretchable electrodes by engineering polymer chains. (n) Conductivities and stretchabilities of stretchable electrodes fabricated via diverse materials and fabrication strategies.

Conducting materials govern the electrical performance of stretchable devices and can include thin metal films, LMs, inorganic conductive fillers, and organic conductive fillers. Thin metal films offer high conductivity (∼10^6^ S/cm) but are inherently brittle under lateral strain (while remaining flexible under vertical strain), requiring structural engineering to enhance mechanical compliance. Nanoscale inorganic conductive fillers (e.g., nanoparticles (NPs), NWs,^897^ CNTs,^898^ graphene, and nanosheets^899,900^ ), provide tunable conductivity depending on their dispersion and alignment within soft elastic polymer matrices. LMs,^901^ discussed in depth in Section 8.3, exhibit both high conductivity and deformability, and can be integrated with elastic polymers or used to form electrodes within elastic channels.^902^ Organic conductive fillers (e.g., PEDOT:PSS, PANI, and PPy) have lower conductivity (∼10^–2^ to 10 S/cm) than metal films, LMs, and inorganic conductive fillers, but possess both ionic and electronic conductivity, making them particularly useful for bioelectrodes.

Soft polymeric materials include elastomers and gels. Elastomers, such as Ecoflex, PDMS, polyurethane (PU), and styrene-ethylene-butylene-styrene (SEBS), exhibit high elasticity (>300%), robust covalent bonding, and excellent chemical resistance, allowing them to withstand repeated deformation and maintain long-term durability under chemically harsh environments without mechanical failure.^903^ In contrast, gels, including hydrogels^904^ and organogels,^905^ inherently exhibit high porosity and relatively weak mechanical properties, but offer high ionic conductivity, extremely soft mechanical properties, and high cell permeability. In particular, the exceptional softness of hydrogels (∼kPa) allows for conformal contact with biological tissues, minimizing interfacial impedance and ensuring stable charge transfer at bioelectronic interfaces. Additionally, modifying polymer networks allows hydrogels to incorporate additional functionalities such as adhesiveness and self-healing capabilities, improving durability and prolonged operational stability.^906^

A summary of the characteristics of the most used materials for soft electronics is shown in Table 1.

**Table 1:** Comparison of Electrode Materials for Wearable Electronics Applications

Materials		Advantages	Disadvantages	Processing methods
Flexible Materials	Thin solid metal layer	High conductivity	Rigid	Photolithography
		Compatible with existing processes	Limited flexibility	
			Crack formation under strain	
	Graphene	High conductivity	High cost	Photolithography
		Flexibility	Challenging large-scale production	Soft lithography
		Biocompatibility		
	Silver nanowires/nanofibers (AgNW/NF)	High conductivity	Prone to oxidation	Electrospinning
		Excellent flexibility	Reduced durability under deformation	Photolithography
		Transparency		
Soft Materials	Conductive polymers	Biocompatibility	Lower conductivity compared to metals	Photolithography
		Good mechanical properties	Limited durability under deformation	Solution casting
		Stretchability		
	Hydrogels	Stretchability	Sensitive to moisture	Solution processing
		Biocompatibility	Limited fabrication methods;	3D printing
		Wet adhesion	Low durability	
	Liquid Metals	High conductivity	Difficult handling	3D printing
		Extreme softness		Injection molding
		Biocompatibility		

Next, we review the two complementary strategies for achieving stretchability in soft electronic systems: those based mainly on structures and those based mainly on materials. Together, these schemes provide routes to a full range of stretchable components, including electrodes, transistors, batteries, photodetectors, diodes, and LEDs, in some cases with integration densities and levels of performance approaching those of rigid systems.

### 8.1 Structural-Oriented Approaches

Various structural design strategies achieve soft electronics by combining hard, brittle electronic materials in strategic geometrical designs with soft elastomeric substrates and encapsulation layers. These approaches allow the resulting hard/soft constructs to maintain electrical integrity while responding elastically to large strain deformations. Traditional materials such as silicon (Si), germanium (Ge), and gallium arsenide (GaAs) become highly flexible when rendered in thin geometries, simply due to the cubic dependence of the bending rigidity on thickness and an inverse dependence of the maximum strain on thickness for a given bending radius.^907^ Thin-film deposition and patterning methods can yield flexible nanomembranes, nanoribbons, nanowires, and nanomeshes in configurations that allow their transfer printing onto elastomers. In planar geometries, integration in this manner allows strains of only ∼1%, limited by the fracture thresholds of the inorganic materials.^908^ To achieve stretchability, these thin materials can be configured into various geometries before or during the integration process, allowing effective end-to-end strains of the resulting structures of up to 100% or more without mechanical failures. Common designs include wavy, serpentine, helical, polygonal lattice, origami, and kirigami layouts (Figure 44B,C).

The formation of wavy structures follows from ultrathin films fully or selectively bonded to an elastomer substrate in a state of prestretch, followed by release. This buckling procedure imposes compressive forces on the films, thereby causing them to transform spontaneously into well-defined wavy shapes, sometimes assisted by thermal expansion or moisture-induced swelling.^909–911^ In advanced versions, this transformation of the initially planar structures yields well-defined 3D architectures. After full encapsulation, the results of this process can form the basis for soft electronics. In optimized layouts, these structures can sustain, for example, uniaxial stretching of up to 120% with an elastic response and an effective modulus comparable to that of the elastomer with little mechanical loading associated with the hard material component.^912^ Other two-dimensional designs exploit island-bridge architectures, where rigid “islands” support the active components and flexible “bridges” form electrical interconnects to absorb mechanical strain.^913,914^ These structures can be formed by photolithography, transfer printing, multidirectional writing, laser ablation, and/or conformal coating, with the ability to establish robust adhesion to elastomeric substrates.^915,916^

Among these options, serpentine interconnects, composed of periodically repeating half-circular arcs, enable high stretchability through both in-plane rotation and out-of-plane buckling, especially when the traces are narrow, thin, and long.^917,918^ Using such structures, circuits can exhibit less than 0.2% maximum principal strain under an applied strain of 90%.^919^ Through deterministic compressive buckling methods similar to those mentioned previously, these 2D serpentines can transform into 3D helical structures, which more effectively distribute mechanical stress and reduce failure risk compared to their planar counterparts.^920,921^ Polygonal lattice and self-similar structures further support high-density integration of functional components while maintaining mechanical compliance.^922^ For example, a rotationally symmetric cellular lattice with 60% porosity and optimized arc angles can withstand up to 100% tensile strain under an external stress of 50 kPa while maintaining a low elastic modulus (∼0.5 MPa).^923^ Designs inspired by the ancient arts of origami and kirigami can further extend mechanical compliance through folding and cutting techniques. Origami involves the folding of 2D sheets along predefined creases to achieve 3D structures that isolate integrated functional components from strains associated with deformations.^924,925^ Examples of this scheme are in stretchable lithium-ion batteries, soft robotics, and wearable sensors.^926^ Kirigami incorporates periodic cuts to distribute mechanical stress uniformly, enabling out-of-plane buckling motions and in-plane rotations to preserve electrical performance under large strain deformations.^927^ Such systems include GaAs solar trackers that dynamically respond to light direction at the substrate level without the need for costly, cumbersome structural supports, as an example of their potential.^928^

Ultrathin metal films (e.g., Au, Ag, and Pt) deposited on soft substrates such as SEBS and PDMS can form crack-based intrinsically stretchable electrodes (Figure 44D).^929,930^ The inherent mechanical mismatch between the metal films and elastomers induces nanoscale and/or microscale cracks, which help distribute mechanical stress and mitigate localized strain while maintaining electrical percolation networks, even under stretching. To further enhance stretchability, a prestretching strategy can be employed. When a metal thin film is deposited onto a prestretched elastomeric substrate, the release of prestrain generates both wrinkled and cracked microstructures, which improve mechanical compliance (Figure 44E).^931^ In addition, stacking ultrathin elastomeric layers with different thermal expansion coefficients facilitates controlled microcrack formation, enhancing stretchability.^932^ Interfacial engineering can also mitigate the delamination of cracked metal films and prevent fatigue-induced degradation of the electrodes.^933^

### 8.2 Material-Oriented Approaches

A second strategy to achieve soft electronic systems uses materials designed or engineered to exhibit elastic responses, enabling mechanical compliance without compromising the electrical performance. These materials generally fall into two primary categories: (1) intrinsically stretchable materials and (2) composites.

The first category commonly utilizes organic polymers with chemical structures that balance considerations in stretchability and electrical performance, primarily through molecular engineering of the polymer backbone or side chains. Conducting polymers typically exhibit high crystallinity and strong intermolecular interactions, which are essential for charge transport but limit mechanical stretchability. To achieve both electrical conductivity and mechanical compliance, it is necessary to modulate polymer chain interactions, control crystallinity, and introduce stress-dissipative mechanisms within the polymer network. Backbone engineering can reduce rigidity by introducing flexible segments, while tuning the strength of interchain interactions enables relaxation of the crystalline domains, thereby increasing chain mobility (Figure 44L).^934,935^ In addition, modulation of interchain interactions (e.g., π-π stacking)^936–939^ and incorporation of dynamic bonds^940^—such as hydrogen bonds, metal–ligand coordination, or reversible covalent linkages—facilitates stress dissipation by allowing the network to temporarily reorganize under strain, preserving structural integrity and electrical continuity. Furthermore, blending small molecular additives such as plasticizers,^941,942^ ionic liquids,^943^ polar solvents,^944^ or surfactants^945^ into conducting polymers disrupts strong intermolecular forces, enhancing the mobility of polymer chains (Figure 44M). This physical softening approach provides a facile means of improving stretchability and conductivity without requiring significant changes to the polymer backbone structure.

One interesting example of such approaches involves incorporating conjugated comonomer units into high-molecular-weight diketopyrrolopyrrole (DPP)-based polymers to enhance their stretchability.^946^ By inducing controlled multiscale ordering, these materials decouple charge transport pathways from mechanical deformation. As a result, DPP terpolymers designed with modified polymer backbones can achieve reversible strains exceeding 100% while maintaining field-effect mobilities greater than 1 cm^2^ V^–1^ s^–1^ even after 1,000 stretching cycles.^947^ Another strategy involves incorporating hydrogen-bonding sites into semiconducting polymers through a stepwise polymerization and thermal conversion process.^948^ These polymers can restore over 90% of their charge carrier mobility after mechanical damage without sacrificing stretchability. Precise submolecular control over the density and spatial distribution of hydrogen bonds enables the formation of supramolecular networks that dissipate mechanical stress and promote rapid recovery under cyclic strains of up to 50%.^949^ Molecular dynamics simulations and nanoconfinement studies provide further insight into how dynamic cross-links regulate network evolution and facilitate efficient damage repair in these stretchable semiconductors.^950^

Composites are equally important to the development of soft electronic systems, as the basis of the second category of materials. Blending various conductive fillers with elastic polymers can form intrinsically stretchable elastomeric nanocomposites with high conductivity and stretchability. Homogeneously dispersed conductive fillers form a 3D percolation network that maintains continuous electrical pathways even under stretching (Figure 44F).^951,952^ Conductivity and stretchability can be further enhanced with a heterogeneous spatial distribution of fillers. For instance, controlling the sedimentation rate of fillers induces a gradient distribution,^953^ and water-assisted self-assembly of nanowires forms locally bundled structures.^954^ Additionally, Marangoni flow-driven float-assembly enables selective surface embedding of fillers, resulting in nanomembranes with patterned conductivity (Figure 44G).^955^ Integrating conductive fillers with different dimensionalities improves network stability under strain. During stretching, 1D fillers tend to align along the strain direction and bridge conductive pathways, while 0D and 2D fillers occupy gaps, preventing disconnection and maintaining conductivity (Figure 44H).^956,957^ Combining functional fillers—such as low impedimetric materials,^958,959^ magnetic materials,^960,961^ or electrochemically sensitive materials^962^—allows for the fabrication of stretchable functional electrodes with tailored properties.

Gel-based nanocomposites, formed by incorporating conductive fillers into soft hydrogel matrices, are promising for bioelectronic interfaces due to their inherent softness, high permeability, and tissue-like mechanical properties.^963^ Optimized structural designs can simultaneously achieve high conductivity and stretchability in physiological environments for soft, tissue-integrated electronics (Figure 44I).^964^ The gel-based nanocomposites not only provide excellent mechanical compliance but also offer dual conduction pathways by integrating the ionic and electronic transport mechanisms. The hydrated polymer network facilitates efficient ion migration with continuous electronic channels provided by embedded conductive fillers. This synergy reduces interfacial impedance and enhances charge transfer efficiency between biological tissues and electronic devices (Figure 44J).^965^ Moreover, gels can be engineered to incorporate tissue adhesiveness and self-healing capabilities through reversible cross-links or supramolecular interactions. These dynamic features are essential for ensuring long-term mechanical integrity and reliable performance in wearable and implantable bioelectronics (Figure 44K).^966^

Regarding the materials used as fillers, nanomaterials should be highlighted. CNTs are important constituents in such systems as they form percolative conductive networks within elastomeric matrices, enabling reliable performance under large strains.^967,968^ In the liquid phase, CNTs can be dispersed uniformly into copolymer matrices via melt blending or solvent casting to create conductive, stretchable, and durable materials.^969–971^ Other nanostructured materials, such as AgNWs and AgNPs, represent additional options as conductive fillers in elastomeric composites. For instance, AgNWs dispersed in styrene–butadiene-styrene (SBS) matrices—combined with ligand engineering and nanowire welding techniques—offer exceptional electrical conductivity and mechanical resilience, achieving elongation at break exceeding 900%.^972–975^ In addition, careful control over deposition techniques can lead to nanoscale fiber-like structures in the polymer semiconductor poly­(3-hexylthiophene) (P3HT), with phase separation in SEBS composites to optimize charge transport and stretchability.^976^ Rubbery strain, pressure, and temperature sensors that withstand stretch up to 50% can be fabricated while maintaining quality measurement accuracy under deformation.^977^

The conductive components in composites can themselves offer high levels of mechanical compliance. An extreme example is LMs, such as eutectic gallium–indium (EGaIn), integrated by injection molding or by filling into microfluidic channels to achieve desired stretchable composites.^978^ EGaIn-filled microchannels accommodate large mechanical strains, while the surrounding elastomer matrix provides self-resealing capability after deformation.^979,980^ In another example, PEDOT:PSS is of interest in soft electronics as a conductive, biocompatible filler in composites.^870,981,982^ Blending PEDOT:PSS with polar solvents or plasticizers, such as ethylene glycol or d-sorbitol, disrupts hydrogen-bond interactions within the polymer network. For example, mixing PEDOT:PSS with ionic liquids resulted in improved stretchability of >100% and enhanced conductivity of >1000 S/cm.^943,983^

Therefore, many materials and strategies exist for the production of soft electronics that are adequate for wearable sensing. The soft and stretchable devices obtained can exhibit very distinct conductivities and stretchabilities depending on the materials and fabrication strategies, which should be considered when designing a device. A summary of these parameters for different techniques and materials can be found in Figure 44N and Table 2.

**Table 2:** Conductivities and Stretchabilities of Stretchable Electrodes Fabricated via Diverse Materials and Fabrication Strategies

Categories	Conductivity (S/cm)	Stretchability (%)	ref
Geometrically designed metal films	∼56,000	∼130	^984^
	Pure gold conductivity ∼ 450,000	∼50	^985^
		∼40	^986^
		∼30	^987^
		∼50	^988^
		∼30	^893^
Crack-based metal films	∼1250	∼400	^989^
	∼16,000	∼300	^932^
	∼20,000	∼160	^930^
Elastomeric nanocomposites	∼200	∼500	^990^
	∼6700	∼150	^898^
	∼6200	∼300	^957^
	∼100,000	∼1000	^955^
	∼11,000	∼50	^897^
	∼30,000	∼840	^952^
	∼45,000	∼1200	^991^
	∼2000	∼1000	^992^
	∼2100	∼500	^993^
	∼150	∼600	^994^
	∼90	∼500	^995^
	∼42,000	∼270	^957^
	∼31,000	∼1000	^956^
	∼120,000	∼200	^954^
	∼36,000	∼50	^996^
	∼1500	∼200	^900^
	∼15,000	∼100	^953^
	∼11,000	∼500	^958^
Gel-based nanocomposites	∼10	∼400	^964^
	∼4100	∼800	^943^
	∼0.01	∼50	^997^
	∼3300	∼100	^899^
	∼370	∼250	^998^
	∼700	∼400	^905^
	∼0.4	∼40	^999^
	∼0.03	∼600	^1000^
	∼20	∼350	^963^
	∼0.2	∼120	^1001^
	∼80	∼40	^1002^
	∼0.02	∼550	^1003^
	∼10	∼150	^1004^
	∼0.1	∼100	^1005^
	∼0.5	∼40	^966^
	∼90	∼800	^1006^
	∼17,000	∼250	^1007^
	∼170	∼90	^1008^
	∼250	∼400	^1009^
	∼400	∼130	^1010^
Conducting polymers	∼6000	∼100	^1011^
	∼40	∼35	^1012^
	∼50	∼20	^982^
	∼30	∼20	^1013^
	∼300	∼30	^1014^
	∼550	∼20	^1015^
	∼670	∼20	^1016^
	∼40	∼50	^1017^

Because of their relevance to soft electronics applied to healthcare, LMs will be highlighted in the following section.

### 8.3 Liquid Metals for Wearable Healthcare

Liquid metals (LMs) are metals or alloys kept in the liquid state at room temperature, typically possessing high electrical and thermal conductivity, excellent deformability, low toxicity, and intrinsic self-healing capabilities.^1018^ These attributes have positioned LMs, particularly gallium-based alloys, at the forefront of flexible sensor and wearable device innovation for healthcare.^1019^ LMs can be integrated into soft, stretchable, and biocompatible systems, thus allowing for sensors that seamlessly conform to complex biological surfaces, such as human skin, joints, and organs.^1020–1022^ LMs have been utilized in mechanoelectrical sensors for motion analysis and strain detection and in biochemical sensors capable of detecting biomarkers.^4,1023^ They are ideal for electromechanical sensing, including body motion tracking, pressure and strain monitoring, and haptic interaction, where precise and reliable detection is essential.

#### 8.3.1 LM-Based Healthcare Applications

Figure 45 illustrates the various uses of LMs in healthcare monitoring,^1022,1024^ which include sports and human–machine interfaces.^1025,1026^ LM-based sensitive elements can be employed in tactile sensors,^1025,1027,1028^ strain sensors,^1029,1030^ and pressure sensors.^1031,1032^ Highly conductive LMs can be used as electrodes and interconnections for wearable physicochemical sensors, encompassing ultrasound sensors,^1020^ electrophysiological sensors,^1033^ and chemical sensors.^1034,1035^ Unlike rigid materials, LMs maintain mechanical integrity and electrical functionality during stretching and bending.^1036^ Integrated into patches, gloves, or textiles, LM-based sensors enable precise monitoring of joint motion,^1037^ muscle activity,^1035^ and tactile interactions,^1025,1027,1028^ providing real-time biofeedback for early abnormality detection, rehabilitation, and enhanced human–machine interaction.

**Figure 45:**
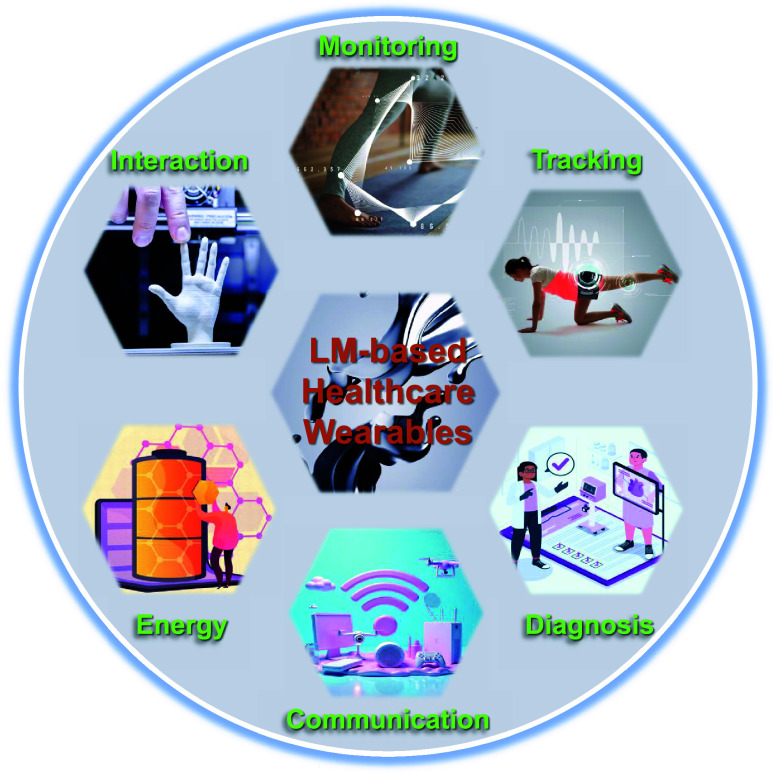
Overview of LMs toward smart healthcare. Image source: Freepik.^1038^

The fluidic properties of LMs are exploited in electromechanical sensors, such as pressure and strain sensors constructed by encapsulating LMs within microchannels. For instance, Yu et al. produced a stretchable tubular elastomeric piezoresistive (STEP) microfiber by embedding LMs into single elastomeric microtubes (Figure 46A).^1029^ These STEP microfibers exhibit such characteristics as softness, thinness, flexibility, stretchability, and washability. By incorporating the STEP microfibers into targeted areas—e.g., ball and heel of socks—accurate measurement of plantar pressure can be performed during locomotion. The electrical signals generated by the STEP microfiber sensors provide real-time monitoring and visualization of the gait cycle, capturing phases such as heel strike, midstance, and toe-off. In addition to piezoresistive-based electromechanical sensors, LM sheath-core microfibers are suitable for electromechanical sensors utilizing triboelectricity. Zheng et al. reported stretchable, highly conductive LM sheath-core microfibers to work as self-powered electromechanical sensors. As shown in Figure 46B, when embedded into fabric gloves worn on a prosthetic hand, bending the fingers generates friction between the LM microfibers and polyurethane fibers in the glove, producing detectable voltage and current. Similarly, when the fiber is adhered to a human wrist, friction between the fiber and the skin produces triboelectric signals.^1039^

**Figure 46:**
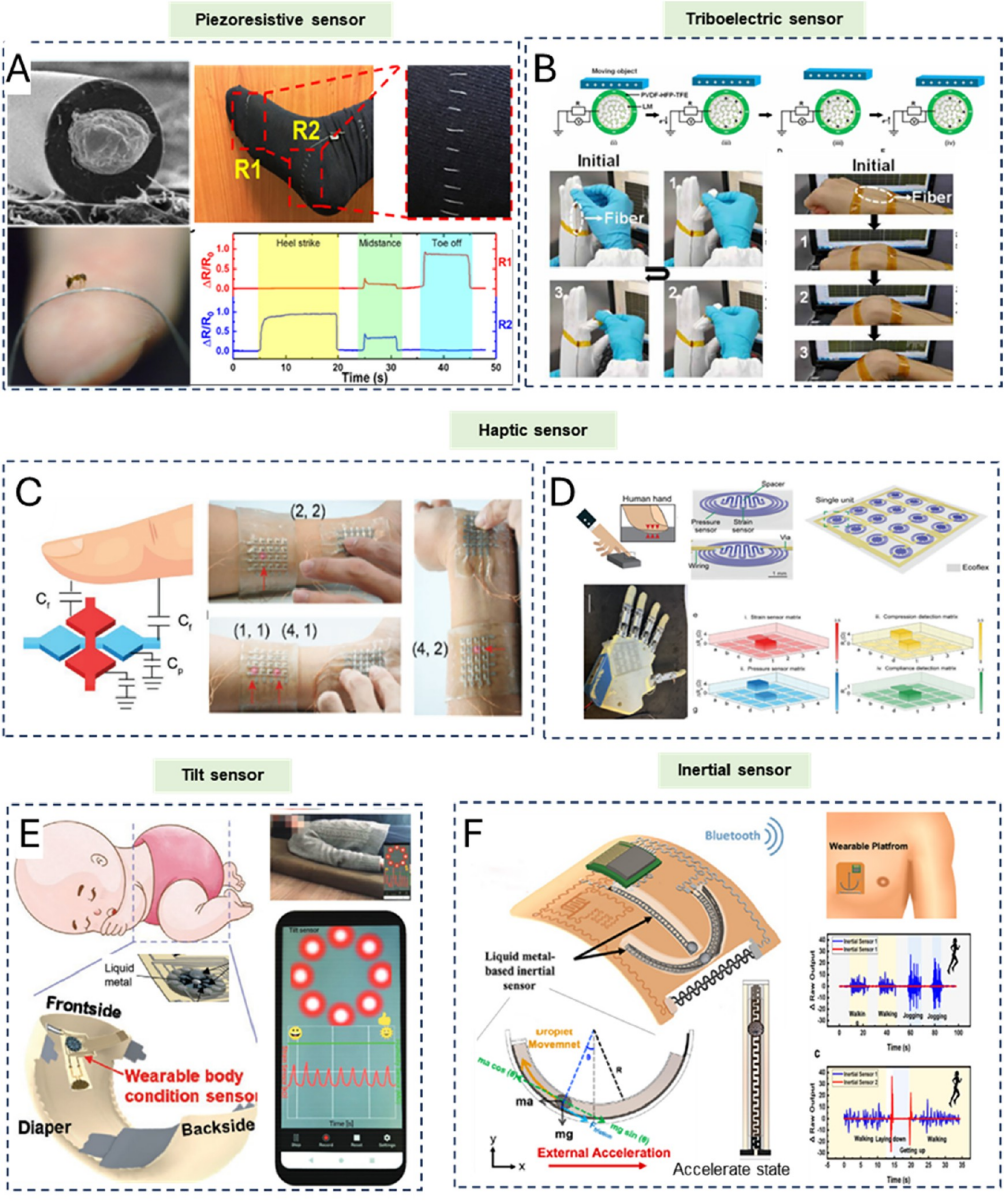
LMs as sensing elements in wearable healthcare applications. (A) LM-based piezoresistance sensor for monitoring plantar pressure during locomotion. Reproduced from Yu et al.^1029^ © 2018 American Chemical Society. (B) LM-based triboelectric sensor for detecting hand and wrist motions. Conductance-stable LM sheath-core microfibers for stretchy smart fabrics and self-powered sensing. Reproduced with permission from Zheng et al.^1039^ © 2021 American Association for the Advancement of Science. (C) LM-based sensor for tactile display interaction. Reproduced with permission from Chen et al.^1025^ © 2023 John Wiley & Sons. (D) LM-based sensor to mimic human-like compliance perception. Reproduced from Chen et al.^1027^ Available under a CC BY-NC license. © 2024 John Wiley & Sons. (E) LM-based title sensor. Reproduced with permission from Xu et al.^1022^ © 2022 John Wiley & Sons. (F) LM-based inertial sensor for posture and body motion monitoring. Reproduced from Babatain et al.^1026^ © 2022 American Chemical Society.

Tactile sensors often utilize LM-based configurations, such as domed or patterned microchannels, which enable the detection of distributed mechanical events, surface roughness, and object stiffness. They can provide precise haptic interfacing, essential for applications in prosthetics, robotics, and virtual reality. For example, Chen et al. fabricated electrode arrays using a TPU and eGaIn-based composite, with a dielectric layer sandwiched between two layers of electrode arrays, forming a capacitive pressure sensor array. As illustrated in Figure 46C, the integrated system demonstrated haptic interactive capabilities. Specifically, the tactile sensor was able to control LEDs through an external controller, enabling functionalities such as one-point or multipoint sliding, pressing, and precise location monitoring.^1025^ A sensor array comprising bimodal sensing units that integrate resistive, strain, and pressure sensors was fabricated using EGaIn LM embedded within elastomer membranes, as shown in Figure 46D.^1027^ To mimic human-like compliance perception, a 4 × 4 sensing matrix was designed to fit the dimensions of a human or robotic palm. This system not only directly captured the responses of strain and pressure sensors but also calculated compression and the corresponding compliance, providing a comprehensive representation of tactile interactions.

LMs are also employed in motion sensors, including tilt and inertial sensors. In these devices, LM droplets are confined within cavities or microchannels, where their movement under gravitational or inertial forces generates detectable changes in electrical properties. For example, Xu et al. reported a wearable body condition sensor system capable of wirelessly monitoring sleeping posture, respiration rate, and diaper moisture, with a built-in feedback alarm notification system. A key component of the system is a tilt sensor that utilizes an LM droplet confined within a cavity, enabling the detection of at least 18 slanting orientations. To continuously track sleep status, the system incorporates a multimodal flexible sensor sheet integrated with a wireless feedback signal processing unit, which is designed to be placed on a disposable diaper. This setup allows real-time multichannel signal detection and transmission to a smartphone interface, providing data on breathing patterns, diaper wetness, and sleeping posture as shown in Figure 46E.^1022^ Similarly, Babatain et al. proposed a fully soft, laser-induced graphene (LIG) and LM-based inertial sensor integrated with additional capabilities for temperature, humidity, and breathing monitoring. The inertial sensor employs a design that confines a graphene-coated LM droplet within a fluidic channel, allowing the droplet to roll over LIG resistive electrodes. This configuration enables the precise detection of motion through resistance variations, which was validated by attaching the sensor to a human subject’s chest during activities such as walking, jogging, lying down, and getting up. Real-time monitoring successfully captured resistance changes corresponding to each activity, demonstrating its capability for motion tracking and multifunctional health monitoring, as shown in Figure 46F.^1026^

#### 8.3.2 Liquid Metals as Conductive Electrodes in Wearable Healthcare Applications

LMs are used in electrophysiological sensors (ECG, EEG, and EMG)^1033^ and flexible energy-harvesting systems.^1040,1041^ LM-based electrodes enhance the performance of wearable electrochemical sensors for real-time biomarker detection and offer improved durability in wireless power transmission and communication systems. By way of illustration, Hu et al. fabricated a wearable ultrasonic device for continuous, real-time, and direct cardiac function assessment (Figure 47A).^1020^ To improve the mechanical coupling between the device and human skin, allowing the left ventricle to be examined from different views during motion, eutectic gallium–indium LM was selected to fabricate electrodes and interconnections. A higher stretchability and fabrication resolution were obtained than with electrodes based on serpentine-shaped copper thin film. Similarly, Ding et al. developed skin-adhesive LM particles (ALMP) for fabricating epidermal electronics designed for wear-resistant electrophysiological monitoring. The ALMP demonstrated self-adhesive and ultraconformal properties, ensuring ultralow skin contact impedance and high-quality electrophysiological signal acquisition. Using this technology, they captured the flexion of the flexor carpi ulnaris with stable and reliable EMG signals (Figure 47B).^1042^

**Figure 47:**
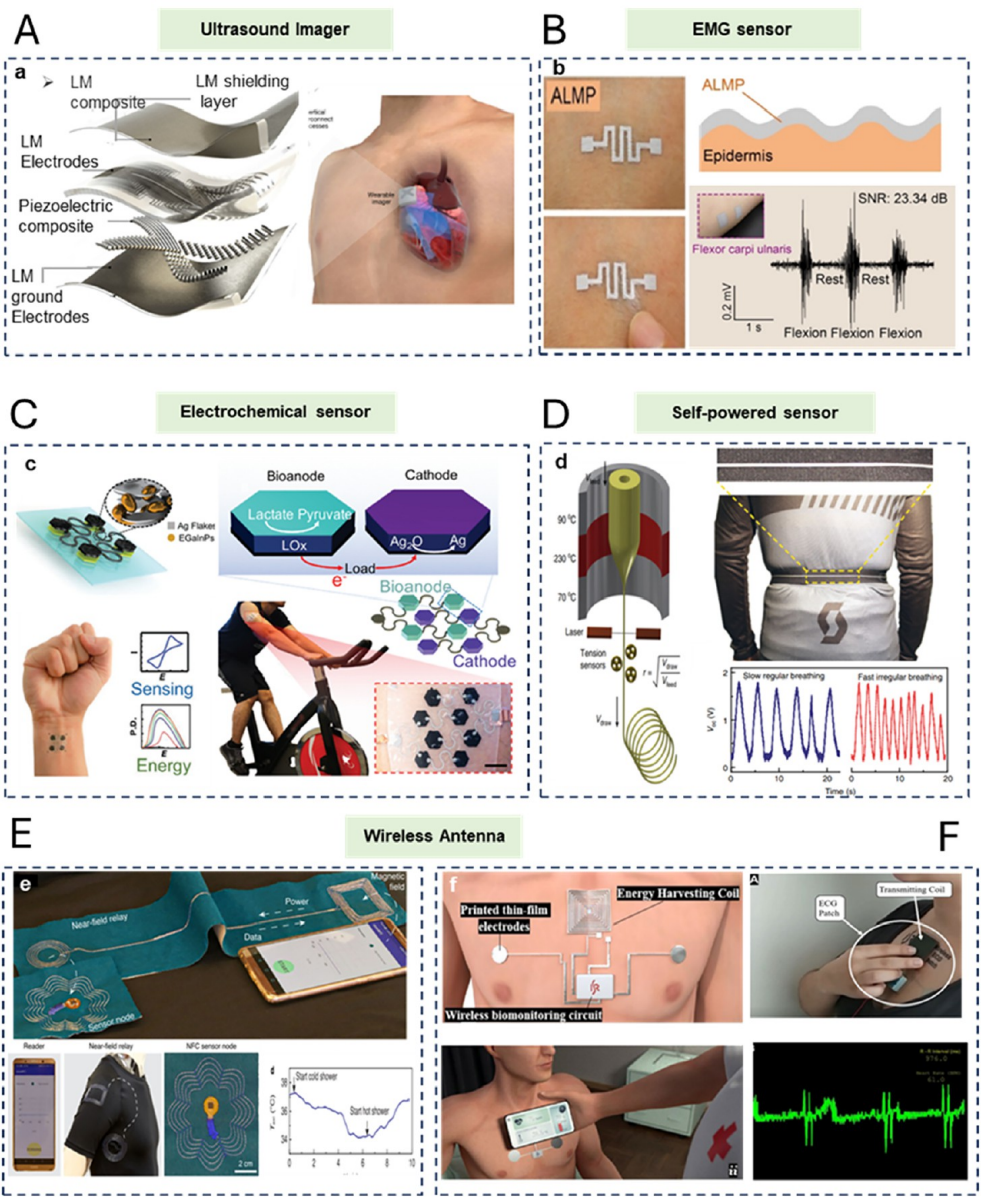
Liquid metals as conductive electrodes in wearable healthcare applications. (A) Wearable cardiac ultrasound imager. Reproduced from Hu et al.^1020^ Available under a CC BY 4.0 license. © 2023 Springer Nature. (B) LM-based epidermal electronics designed for wear-resistant electrophysiological monitoring. Reproduced from Ding et al.^1042^ © 2022 American Chemical Society. (C) LM-based stretchable electrochemical devices as epidermal biofuel cells. Reproduced with permission from Chen et al.^1044^ © 2024 John Wiley & Sons. (D) LM-filled microstructured stretchable TENG fiber-based textile for self-powered breathing monitoring. Reproduced from Dong et al.^1040^ Available under a CC BY 4.0 license. © 2020 Springer Nature. (E) LM-based electronic textile systems with near-field wireless power and communication capabilities. Reproduced from Lin et al.^672^ Available under a CC BY 4.0 license. © 2022 Springer Nature. (F) LM-based electronic tattoo with an energy harvesting coil for wirelessly monitoring ECG signal. Reproduced from Alberto et al.^1048^ Available under a CC BY 4.0 license. © 2020 Springer Nature.

In electrochemical sensing, LMs were used in printed stretchable devices.^1043^ Stress-resistant composite silver (Ag) inks were combined with eutectic gallium–indium particles as dynamic electrical anchors, patterned into serpentine “bridges” connecting “island” electrodes. This architecture enabled high stretchability and stable electrochemical performance under deformation. A key application was epidermal biofuel cells, which harvest bioenergy from sweat metabolites as shown in Figure 47C.^1043^ However, few studies have explored LM-based electrochemical sensors, as challenges like LM’s susceptibility to electrochemical oxidation and its reactivity in acidic or basic environments limit their applicability in broader electrochemical scenarios.^1044^

In addition to conductive properties, LM-based energy-harvesting technologies have been developed to address the need for portable, wearable energy systems capable of storing and harvesting power for prolonged use of personal electronics. Examples include TENGs, piezoelectric nanogenerators, and TEGs, which will be discussed at length later on in this review paper (see Section 10). These systems harness energy from various body motions directly into electricity, enabling efficient and continuous operation of wearable devices. For example, an LM-filled microstructured stretchable TENG fiber-based textile was proposed to exhibit an OCV and a short-circuit transferred charge (Q_sc_) as high as 490 V and 175 nC, respectively, as shown in Figure 47D.^1040^ LMs are also suitable for stretchable antennas in RF communication devices.^672,1045–1047^ By integrating LM-based antennas, healthcare devices can achieve wireless functionality for both data communication and power transfer. Indeed, electronic textile systems with near-field wireless power and communication capabilities were produced by digitally embroidering LM fibers onto clothing. With these textiles, robust and unobtrusive wireless power transfer can be exploited for near-field communication with nearby devices, such as health sensors and smartphones, even during strenuous exercise and daily activities. These wirelessly enabled garments can power bioelectronic implants in patients, detecting diseases in high-risk populations like the elderly. They can also monitor axillary temperature—a key indicator of exhaustion, physical performance, and various health conditions (Figure 47E).^672^ Alberto et al. reported a LM-based electronic tattoo capable of wirelessly harvesting energy through a passive antenna. This system employs a wireless power transfer (WPT) mechanism, integrating an inductive coil antenna with biopotential acquisition electrodes that interface directly with the skin. The design enables efficient wireless power transfer of approximately 300 mW across the skin and up to 100 mW beneath the skin, offering reliable energy support for wearable healthcare devices. This electronic tattoo can be incorporated into monitoring systems for real-time collection and transmission of biopotential signals, such as heartbeat rate, via Bluetooth. By combining energy harvesting, biopotential sensing, and wireless communication, this LM-based electronic tattoo may be used for long-term, remote patient monitoring and management (Figure 47F).^1048^

### 8.4 Active Components in Soft Electronics

Active components in electronics, such as transistors and diodes, as well as LEDs and photodetectors (PDs), can be achieved using the strategies outlined in the previous section for broad classes of soft electronic systems. Stretchable organic field-effect transistors (OFETs) and organic electrochemical transistors (OECTs) play essential roles in low-power, stretchable logic circuits. OFETs are commonly fabricated through solution processing techniques such as inkjet printing, spin coating, and layer-by-layer deposition. The methods described previously support stretchability while preserving charge carrier mobilities in otherwise conventional device designs.^1049–1051^ OECTs operate using different principles, commonly through redox-active PEDOT:PSS channels in a three-electrode configuration. Here, the application of gate voltage modulates the entire channel conductivity via electrochemical doping. This process results in high transconductance and signal amplification at low operating voltages.^1052,1053^ Both OFETs and OECTs have been built into soft electronic platforms and have been integrated into soft electronic devices, including displays, wearable sensors for chemical, physical, and biological monitoring, neural interfaces, electronic skin, and biomimetic systems for artificial perception.^1054–1057^ Due to their importance, thin film transistors (TFT) will be discussed in further detail in the following subsection.

Recent advances in stretchable solar cells, energy storage, and energy harvesting systems provide attractive options for power management (see Section 10). For instance, some of the best types of stretchable organic photovoltaic cells can maintain 80% of their initial power conversion efficiency under tensile strains of greater than 50%.^1058^ Structural designs, such as those that involve weaving functional filaments into fabrics, facilitate conformal, self-powered wearable sensors. In energy storage, printed Ag_2_O–Zn and Ag/AgCl batteries, as well as stretchable lithium-ion and emerging zinc-air chemistries, offer high energy densities while tolerating repeated cycles of mechanical deformation.^1059,1060^ The development of graphene-MXene composites serves as the basis for stretchable supercapacitors, as efficient secondary power sources for wearable systems.^1061^ Furthermore, stretchable energy-harvesting technologies, including TENGs, enzymatic biofuel cells, and wireless power transfer circuits based on inductive, capacitive, or radio-frequency (RF) mechanisms, enable continuous energy delivery.^1062–1064^ These systems support the sustainable, long-term operation of soft electronic platforms without the need for bulky or rigid power components.^1065,1066^

Perovskites, quantum dots, and organic light-emitting conjugated polymers serve as key building blocks of nanocomposites for stretchable LEDs.^1067,1068^ Advanced fabrication techniques, including inkjet printing, transfer printing, and nanoconfinement, enable the integration of these materials into ultrathin mesh layouts on stretchable substrates, preserving both optical performance and mechanical resilience.^1069,1070^ Similarly, stretchable PDs can be constructed with a range of nanostructured materials, including AgNWs, semiconducting P3HT nanofibers, and hybrid systems that combine low-dimensional perovskites with wrinkled graphene. These PDs can sustain tensile strains exceeding 50% with minimal reductions in the photocurrent.^1071^ When embedded in soft, biocompatible substrates, these LEDs and PDs conform intimately to biological tissues, enabling a range of applications including epidermal pulse oximetry, UV dosimetry, and noninvasive biosensing and bioimaging.^1072^

### 8.5 Thin-Film Transistors

Flexible and large-area electronics are important for various applications, from next-generation displays^1073–1075^ and large-area image sensors^1076,1077^ to wearable health monitors,^626,1078–1080^ digital microfluidics,^1081,1082^ and thin-film microprocessors.^1083–1085^ At the heart of these technologies lie TFTs, which enable lightweight, conformable, and scalable electronic systems. Flexible circuits typically use thin-film semiconductors deposited on plastic substrates (e.g., polyimide, PET). Key material platforms include silicon (single crystals, polycrystalline (LTPS), and amorphous), organic semiconductors (e.g., conjugated polymers, small molecules), oxide semiconductors (e.g., amorphous Indium Gallium Zinc Oxide—a-IGZO), and 2D semiconductors (e.g., MoS_2_, WSe_2_).

In this section, we review and compare them, focusing on flexible-device demonstrations and benchmarks. Table 3 compares four semiconductor classes (silicon, crystalline and amorphous, organic polymers, a-IGZO, and 2D materials like MoS_2_) across key metrics for flexible electronics. Amorphous and crystalline Si are brittle: bulk Si typically fractures at ∼1% strain,^1086^ a-Si is similar, whereas organic films can accommodate much larger deformation (organic light-emitting electrochemical cells, OLEC, devices stretch ≈30% without cracking).^1087^ Two-dimensional carbon shows the best stretchability—pristine graphene can sustain ≈ 20–25% strain—but many 2D semiconductors are weaker, e.g., monolayer MoS_2_ fails around 6–11%.^1088,1089^ Electron mobility likewise varies widely. Organic polymers generally have very low mobilities (<10 cm^2^/V·s and often <1 cm^2^/V·s),^1090^ whereas graphene’s carriers are extraordinarily mobile when sandwiched between a proper dielectric like boron nitride, hBN (∼2 × 10^5^ cm^2^/V·s).^1091^ Crystalline Si is intermediate (∼1.35 × 10^3^ cm^2^/V·s for electrons).^1092^ In practice, silicon’s native oxide makes Si devices chemically stable, and graphene’s inert sp^2^ carbon network also resists corrosion, whereas organic films tend to degrade under environmental exposure (O_2_, moisture).

**Table 3:** Properties of Semiconductor Materials for Flexible Electronic Devices

Material		Max Fracture Strain (%)	Electron Mobility (cm^2^/V·s)	Chemical Stability	Biocompatibility	Large-Area Fabrication	Miniaturization	CMOS-Compatibility
Silicon	Crystalline thin-film (Nanomembrane/wires)	∼5	∼1000	High	Medium-High	Low	Very High	Very High
	Amorphous Si (a-Si)	∼1–2	∼0.5–1	High	Medium-High	High	High	Very High
	LTPS	∼0.5–1	∼100	High	Medium-High	Medium	High	High
Organics		∼20–30	∼0.1–1	Low	Very High	Very High	Low–Medium	Low
Oxides		∼2.9	∼10–20	Medium-High	Medium	High	Medium	Medium
MoS_2_		∼6–11	∼10–100	Medium	Medium	Medium	Very High	High

Biocompatibility follows a similar trend: Si-based materials (including silicon oxide surfaces) and many bioengineered organics are known to be well tolerated *in vitro* and *in vivo*.^1093^ In contrast, oxide materials (such as ZnO/IGZO) and some 2D semiconductors can leach ions or produce reactive byproducts; for example, ZnO nanowires show measurable cytotoxicity due to Zn^2+^ release.^1094^ A key concern with a-IGZO materials is the potential biocompatibility and toxicity issues associated with indium, which can pose risks if released or degraded in biological environments. Careful material encapsulation and further biotoxicity studies are essential to ensure safe application in biointegrated devices.

Large-area manufacturability also differs. Organic semiconductors are solution-processable, enabling wafer-scale, low-cost films. In contrast, wafer-scale 2D film growth is still maturing: CVD and epitaxial methods exist, but defects and contamination are still problematic at the production scale.^1095,1096^ Finally, Si leads in CMOS scaling: conventional silicon processes allow aggressive lithography and device downscaling. Amorphous IGZO can be patterned below 1 μm and even integrated back-end-of-line on CMOS wafers.^1097^ By comparison, 2D materials face integration challenges: their interfaces (dielectrics/contacts) and etching processes are not yet mature, making “CMOS+2D” hybrid integration challenging.^1095^ Figure 48 illustrates these properties with a radar graph.

**Figure 48:**
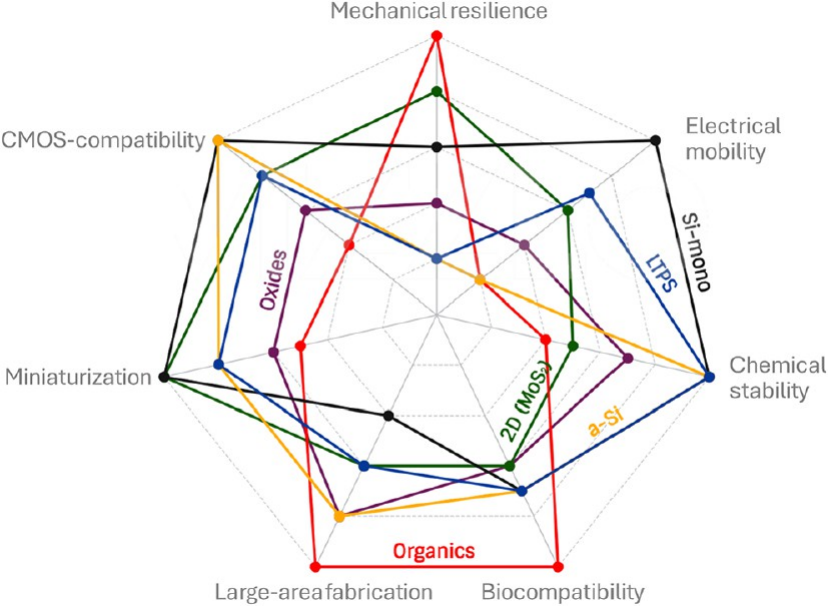
Benchmark of semiconductor materials for flexible electronics.

#### 8.5.1 Silicon-Based TFTs (Si NMs, Si NWs, a-Si, Poly-Si)

##### 8.5.1.1 Silicon Nanomembranes (Si NMs)

Ultrathin single-crystal Si layers (tens of nm) peeled from wafers and bonded onto polymer substrates combine the electronic quality of bulk Si with flexibility.^893,1098^ These Si NMs can be patterned into conventional CMOS devices, with built-in tensile strain (e.g., from Si/SiGe/Si stacks) to boost carrier mobility. In practice, Si NM FETs on plastic show electron mobilities of a few hundred cm^2^/V·s and hole mobilities ∼100–150 cm^2^/V·s (comparable to SOI devices). Gate currents and interfaces behave like rigid Si MOSFETs (low flicker noise, negligible bias drift), with on/off ratios easily >10^6^ (due to Si’s bandgap and doping) and subthreshold slopes around 100 mV/dec. Threshold voltages are similar to bulk Si (∼0.5–1 V). In RF tests, even unstrained Si NM transistors (channel ∼ 0.5 μm) achieved f_T_ ≈ 3.3 GHz. Strain-engineered Si NMs (e.g., Si/SiGe/Si trilayers) showed a ∼47% mobility boost, pushing f_T_ toward 6 GHz.^1099^ Si NMs tolerate bending to millimeter-scale radii: theoretical fracture of a 20 μm device-on-polyimide happens at a radius of about 1 mm, and experiments have bent Si-NM circuits around a 2 mm radius without damage.^1100^ Key trade-offs are cost and process complexity: SI NMs require Si wafer processing, oxide release to liberate the silicon from the SOI wafer, followed by a controlled and reliable transfer process, possibly at large scale. Often, NM circuits are cut into islands or meshes for stretchability (e.g., “nanomesh” Si has 25% stretch and μ50 cm^2^/V·s) at the cost of extra steps.^1101^

Advantages of Si NMs include the performance of bulk Si on plastic, which is comparable with standard CMOS devices. Moreover, the designs and toolkit developed for silicon can be exploited to yield high-speed, low-power circuits on plastic. Very high speed (GHz) and transconductance far beyond a-Si or organics, with static off-currents <10 pA. Excellent analog stability and low power (due to low V_th_ and high μ). Also, large-area manufacturing is conceivable. However, the processing cost can be higher, and the flexibility is moderate since flat NMs fracture below ∼mm-scale bend radii unless patterned (islands/meshes) for stretching.

##### 8.5.1.2 Silicon Nanowires (Si NWs)

Quasi-1D Si channels (diameter <100 nm) offer high performance with extreme deformability. Recent demonstrations grow aligned arrays of crystalline Si NWs via a solid–liquid–solid method, then transfer them onto elastomer (PDMS) or plastic (PET) substrates.^1102,1103^ The resulting Si NW FETs achieve high hole-carrier μ ≈ 70 cm^2^/V·s (similar for electrons in other work) and *I*_on_/*I*_off_ > 10^5^–10^6^. Subthreshold slopes are low (SS ≈ 134–200 mV/dec), reflecting good interface quality. Because the nanowires are built on small “islands” of circuitry, these devices tolerate enormous strain: reported stretchability is up to 50% strain, withstanding >1000 cycles at 20% strain.^1103^ Even under such deformation, μ and *I*_on_/*I*_off_ degrade only modestly. The Si NW FET arrays endure tiny bending radii—e.g., bending to *R* ≈ 0.5 mm for 1000 cycles had negligible performance loss. Si NW devices can also be made into logic inverters on plastic, demonstrating scalability.

Si NWs are advantageous for their outstanding mechanical flexibility, as ultrathin Si wires on elastomer island networks yield stretchability (50%) and extreme bend radius (<1 mm). Performance is much better than a-Si or organics, and batch transfer growth allows wafer-scale integration. Devices have high *I*_on_/*I*_off_ and work stably for >270 days without passivation. The disadvantages include a more complex device architecture and a lower mobility than bulk Si NMs (tens vs hundreds cm^2^/V·s).

##### 8.5.1.3 Thin-Film Silicon (a-Si:H and LTPS) TFTs

Conventional display backplanes use hydrogenated amorphous Si (a-Si:H) or LTPS on glass or plastic. a-Si:H TFTs (PECVD-deposited at <300 °C) have very low carrier mobility (∼0.5–1 cm^2^/V·s for electrons, ∼0.01 cm^2^/V·s holes), limiting speed to tens of kHz. Nevertheless, they reliably deliver *I*_on_/*I*_off_ > 10^6^, threshold <3 V, and SS < 0.5 V/dec. These devices are intrinsically bendable if the substrate is thin enough: early work on 75 μm glass foils achieved a bending radius ∼5 mm without loss of function.^1104^ Amorphous Si is stable and cheap (roll-to-roll PECVD), but has poor mobility and voltage stability under stress.^1105,1106^

By contrast, LTPS (excimer laser annealed poly-Si) on plastic glass has much higher mobility (tens to hundreds cm^2^/V·s). For example, flexible LTPS devices have demonstrated μ up to ∼174 cm^2^/V·s (on a 20 μm PI substrate). On/off ratios exceed 10^6^ (low leakage ∼ 10 pA). Thresholds are typically a few volts; SS can be ∼0.1–0.3 V/dec^1107,1108^ Current LTPS displays (on glass) operate at tens of MHz; on plastic, the best bendable LTPS reached f_T_ similar to Si NM, but generally LTPS is modest (<100 MHz) due to larger dimensions. Mechanically, flexible LTPS has improved: early reports showed *R* = 20 mm, but modern thin-film LTPS (e.g., on 8–20 μm PI) can bend to a few mm.

Large-area processing is a key feature of LTPS, while a-Si offers simpler fabrication, excellent uniformity, and stability for low-cost flexible sensors and displays. Both technologies can integrate with existing fabs and are proven at scale. However, a-Si devices are slow (low μ, limited to ∼kHz–MHz) and require thicker devices to maintain current, limiting ultimate flexibility. LTPS requires costly laser crystallization and often cannot use plastic substrates without substrate lifting or transfer. Both have poorer flexibility than nanostructured Si: even at ∼10 μm thickness, bending is limited to a few mm (much larger than 2*D*/0D Si devices). Device uniformity (for LTPS) and threshold stability (for a-Si under bias) remain concerns.

#### 8.5.2 Organic Semiconductor TFTs

Organic TFTs (OTFTs) use conjugated polymers or small molecules as the active layer. Organic semiconductors are inherently flexible, can be solution-processed, and allow low-cost large-area manufacturing (printing, coating). Charge transport occurs via hopping between disordered molecular orbitals, typically yielding lower μ than inorganic semiconductors.^1109–1111^ Important advantages of OTFTs include device flexibility (bending radius down to a few mm or less), possible biocompatibility and biodegradability. Hence, OTFTs can be integrated with soft substrates and distinct form factors (e-skin, textile integration), also exhibiting low-cost fabrication.^1112–1114^ There are, however, two main disadvantages, namely, the low mobility (often <5 cm^2^/V·s), which limits speed, and the lack of environmental stability as many organics degrade in air or with electrical stress. Furthermore, multilayer encapsulation is often required.^1115,1116^ In summary, organic TFTs trade high flexibility and low-cost processing for inferior raw performance compared to oxide or silicon devices.^1117,1118^

#### 8.5.3 Oxide Semiconductor TFTs

Amorphous metal-oxide semiconductors (e.g., amorphous In–Ga–Zn–O, a-IGZO) emerged in the mid-1990s and have become mainstream in displays. They are typically n-type, transparent, and can be deposited at low temperature through radio frequency sputtering. Electron transport occurs via overlapping metal 5s orbitals, giving high mobility even in amorphous form.^1119–1121^ Oxide TFTs combine high speed and low leakage with simple fabrication. They are inherently transparent and uniform over large areas. Solution-processed strategies have been used, with performance not yet matching that achieved with vacuum systems.^1122^ The low off-current means oxide-based circuits can consume ultralow static power even if the absence of p-type oxide semiconductors makes complementary design currently challenging. They can operate at modest voltages (1–5 V). Device-to-device uniformity is good on rigid and on flexible substrates.^1121^ An important limitation is that most oxide semiconductors are n-type only (lack a robust p-type counterpart), so complementary logic is hard. Another concern is stability, since threshold voltage drifts under bias/illumination/stress, especially on plastic, requiring compensation circuits.^1123^ Oxygen vacancies can cause trapping and noise. Mobility, though much higher than a-Si, remains far below crystalline Si, limiting ultimate *f*_T_ (∼100 MHz). Also, oxide TFTs often require vacuum deposition (sputtering, ALD) of metal oxides, which is more expensive than printing or solution processes. Bending and long-term environmental stability (humidity/temperature) can degrade performance over time.

#### 8.5.4 Two-Dimensional Semiconductors’ TFTs

Atomically thin 2D semiconductors like MoS_2_, WS_2_, and WSe_2_ provide an extreme form of thinness with a sizable bandgap (e.g., MoS_2_ ∼1.8 eV in monolayer) and inherently excellent gate control. 2D layers are prepared by exfoliation or scalable CVD, then transferred onto flexible substrates with high-κ dielectrics.^1124–1126^ In RF testing, 2D MoS_2_ transistors have shown remarkable speed. Recent work reports *intrinsic* current-gain cutoff frequencies up to ∼40–42 GHz for optimized few-layer MoS_2_ devices. For example, MoS_2_ FET with self-aligned contacts achieved *f*_T_ ≈ 42 GHz.^1127^ Even on flexible substrates, MoS_2_ devices have reached ∼13.5 GHz intrinsic *f*_T_.^1128^ The intrinsic current gain (*g*_m_/*g*_ds_) can exceed 30, allowing voltage gain. Noise performance is favorable compared to graphene (because MoS_2_ can pinch off current), and MoS_2_ has excellent current saturation. 2D transistors tend to have very low gate leakage (thin dielectrics) and modest off-currents.^1129^ The main advantage of these transistors arises from the combination of atomic-scale thickness with relatively high speed. They yield large on/off ratios (unlike graphene) and have shown GHz-range amplification. The atomically thin channel gives excellent immunity to short-channel effects, enabling aggressive downscaling on a flexible substrate without losing gate control. They also allow remarkable flexibility: monolayers can withstand extreme bending. Unlike organics, 2D materials are inorganic crystals and are chemically stable (though some require encapsulation). Their electronic properties can be engineered by layer number, stacking, or doping. On the other hand, fabrication uniformity and scalability remain challenges for 2D materials. CVD-grown films still have grain boundaries and defect scattering. Carrier mobility in realistic devices (with contacts and substrates) is often 10–50 cm^2^/V·s, limiting performance below theoretical potential. ALD-grown 2Ds may provide more controllable large-scale uniformity and compatibility with 300 nm CMOS processing. However, this is an emerging technology with initial experiments showing modest mobility of the final layers. Contact resistance and thermal dissipation are concerns for short-channel devices. Integration of 2D devices often requires complex transfer or growth processes, which are currently less mature than thin-film deposition of oxides or organics.

These trade-offs are summarized in Table 4.

**Table 4:** Comparison of Performance Metrics for Thin-Film Flexible Devices

		μ (cm^2^/(V s))	On/Off Ratio	f_T_	Stability	Power Consumption	Advantages	Disadvantages
Oxide (a-IGZO)		>10 (up to 70)	10^7^	∼10–100 MHz	V_th_ drift under bias/light	Low	High μ & very low leakage; transparent; low-T deposition; mature large-area processes; ultralow static power	n-type only (no p-device); threshold shifts; requires vacuum deposition; μ still below Si
Organic		0.1–3 (up to ∼10 SC)	10^4^–10^6^	kHz–few MHz	Poor (moisture, bias stress)	Low	Intrinsically flexible/stretchable; solution-processable; low cost; biocompatible/biodegradable; novel form factors	Low μ & speed; high SS; significant bias-stress drift; environmental sensitivity; higher noise
2D Semiconductors		10–50 (up to 200)	10^6^	∼10–40 GHz	Substrate influence, interface sensitive	Low	Atomic-scale thickness; very high on/off; GHz-range speed; excellent gate control; flexible	Scalability challenges; grain boundaries and defect scattering; contact resistance; complex integration
Silicon	Si NMs	200–500	10^6^	3–6 GHz	Excellent (CMOS-grade)	Very low	Bulk-Si performance on plastic; CMOS-compatible; ultralow noise; high speed and gm; mature Si processes	Complex wafer bonding/release; high cost; limited bend radius without patterning
	Si NWs	∼70	10^5^–10^6^	∼GHz expected	Excellent	Very low	Exceptional stretchability (50%); ultraflexible; high *I*_on_/*I*_off_; wafer-scale transfer; stable over cycles	Complex guided growth and transfer; lower μ vs Si NMs; island-based architecture adds steps
	a-Si	0.5–1	10^6^	kHz–MHz	poor voltage bias stability	Low	Mature, low-cost large-area PECVD; stable; bendable on thin substrates; excellent uniformity	Very low μ & speed; large SS; voltage instability under stress; limited flexibility
	LTPS	20–500	10^6^	∼100 MHz	some bias drift	Low	High μ for display backplanes; low leakage; good uniformity; moderate flexibility with thin PI	Costly laser crystallization; limited bend radius; threshold drift; process complexity

Therefore, thin-film flexible electronics span a broad range of materials, each with its own unique benefits. Oxide semiconductors (e.g., IGZO) offer high mobility and ultralow leakage for fast, low-power circuits but require improved p-type counterparts and stress stability. Organic semiconductors excel in flexibility and manufacturability and have steadily improved (some polymers now exceed amorphous silicon performance) but still lag in speed and stability. 2D semiconductors like MoS_2_ combine the advantages of high mobility, large on/off ratio, and GHz-speed potential, at the cost of emerging fabrication challenges. Silicon nanomembranes bring true silicon performance to flexible form factors, enabling GHz silicon-like circuits, although with more complex processes. Overall, each technology occupies a different niche: organics for ultraconformable, low-cost large-area sensors; oxides for moderate-speed flexible displays and sensors; 2D materials for eventual high-speed flexible logic; and silicon nanomembranes for best-performance flexible ICs. Continued advances in materials (e.g., novel oxides, conjugated polymers, and 2D alloys) and processes (e.g., better encapsulation, printing techniques) are driving the field forward. Flexible electronics now combine years of materials innovation to approach the performance of rigid silicon in many respects while offering new mechanical possibilities.

### 9 Signal Acquisition

We now focus on describing strategies for effective signal acquisition. The data acquisition process normally includes sensing, signal transmission, and display, according to the following steps: first, wearable sensors detect physical or chemical signals from the human body. These, then, undergo processing to minimize noise and enhance quality. As the signals output from sensors are analogical, they need to be converted to digital signals for effective transmission, which can be achieved by a wired interface or by wireless communication modules. Wired electronics hinder user comfort as the wires interconnecting sensors and read-out systems disrupt users’ natural daily activities and pose a risk of disconnection. These limitations have motivated the incorporation of wireless systems to further improve users’ comfort.^1130,1131^ The most used wireless communication technologies are NFC, Bluetooth, and Wireless Fidelity (Wi-Fi).^236,1132,1133^ They differ in many characteristics, such as the transmission distance, frequency, energy consumption, and application scalability. Figure 49 shows an overview of the methodologies for wireless signal acquisition in wearable sensors.

**Figure 49:**
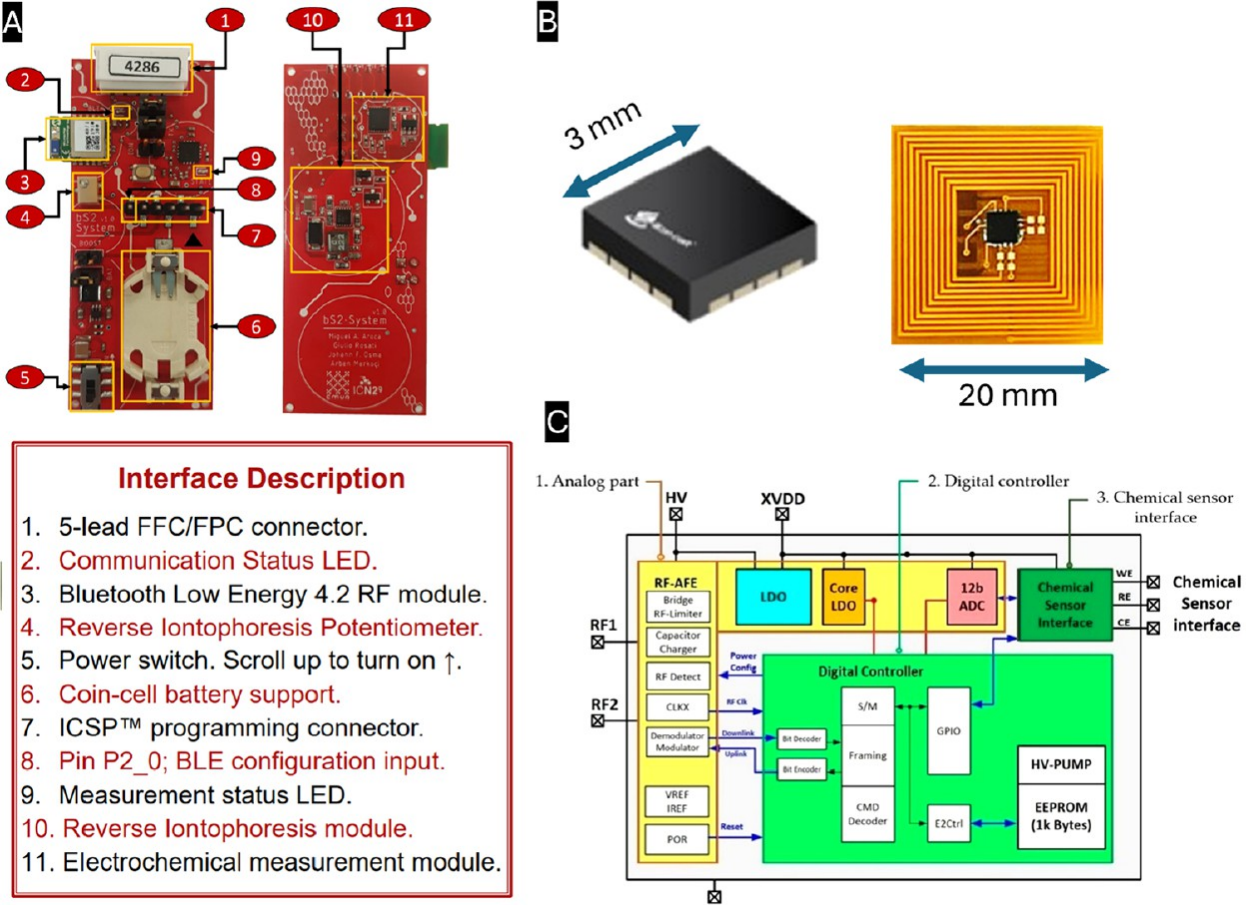
Overview of wireless signal acquisition systems for wearable devices. (A) Hardware configuration of a BLE-enabled potentiostat featuring components such as the BLE module (3), electrochemical measurement module (11), and reverse iontophoresis module (10). (B) Schematic representation of an NFC-enabled electrochemical sensing platform, incorporating the Silicon Craft SIC4341 chip integrated with an NFC antenna for wireless power and data transmission. (C) Block diagram of the SIC4341 chip, highlighting key components such as the RF-AFE for energy harvesting, LDO for voltage regulation, 12-bit ADC for data conversion, and the chemical sensor interface for electrochemical measurements. Reproduced with permission from Yang et al.^531^ Available under a CC BY 4.0 license. © 2022 Elsevier and Uzunçar et al.^1134^ © 2025 John Wiley & Sons.

NFC is a short-range, point-to-point communication method with a transmission distance typically below 10 cm, operating in the high-frequency (HF) band at 13.56 MHz, which is lower than GHz-range wireless technologies such as Bluetooth and Wi-Fi (UHF at 2.4 GHz and SHF at 5–6 GHz). It can operate in passive mode, harvesting energy from the reader and requiring no onboard power source, unlike Bluetooth and Wi-Fi. The NFC system relies on transmitting and receiving coils, where inductive coupling enables data transfer and facilitates battery-free operation. Indeed, Maroli et al. emphasized the battery-free operation and compact design enabled by the NFC,^856^ in contrast with Bluetooth-based systems such as that reported by Yang et al.^1135^ Koh et al. built a small NFC chip on a stretchable antenna for a microfluidic sensor monitoring lactate, chloride, pH, and glucose in sweat and displaying the concentration of each through color-responsive materials.^117^

The Silicon Craft Company developed a potentiostat on a single chip by combining NFC technology with an electrochemical interface. The system can perform electrochemical analyses, including cyclic voltammetry (CV), SWV, and chronoamperometry, directly on a wearable device. Data are transmitted wirelessly via NFC. The unification of potentiostat operations and wireless communication into a single chip allows for the achievement of exceptional miniaturization and simplicity, which, in turn, reduces both cost and complexity. This technology is advantageous for POC applications, where disposable sensors are placed on the skin or in contact with a sample (e.g., sweat or urine) and powered by the reader during the measurement.^1134^ As the transmission distance is quite limited, the safety of the data is at a high level. However, the limited operating range of the NFC reduces its practicality.

For long-distance wireless communication, Bluetooth or Wi-Fi systems have been adopted.^102,1136^ Although these systems require an external power source, they provide a wide communication range and reliable signal acquisition. Bluetooth is used in wearable sensors due to the much longer transmission distance (∼10 m for classic, ∼100 m for industrial use), higher frequency (2.4 GHz), and data transmission speed (∼3 Mbps). Compared with NFC electronics, a Bluetooth module generally needs a more complex design and larger size for the power management circuit and an efficient antenna.^1137^ Martin et al. demonstrated real-time sweat monitoring through a soft microfluidic sensor integrated with a Bluetooth communication module (5.0 × 2.4 cm^2^).^1138^ Qazi et al. demonstrated remotely programmable wireless networks that can control multiple devices simultaneously.^1139^ This wireless system facilitates a large-scale collection of biosignals from various locations.

Wi-Fi possesses the largest transmission distance capabilities (∼30 m indoors and ∼100 m outdoors), frequency (2.4, 5, and 6 GHz), and data transmission speed (up to 1 Gbps). The unique benefit of Wi-Fi is the cloud backup, which is accessible to multiple devices and users. This feature enables collaborative monitoring and analysis of health data. In contrast, NFC is limited to point-to-point communication, and Bluetooth requires pairing or Bluetooth Mesh for mesh networking. Therefore, the energy consumption of Wi-Fi modules is higher than that of NFC and Bluetooth, leading to a higher requirement for efficient power resources and a challenge to the miniaturization of wearable sensors.^1140^ Hybrid communication systems can be developed to meet diverse requirements such as transmission distance, energy consumption, and multiple signal channels. For example, Gaetani et al. stored the hand motion data in a cloud database through Wi-Fi for display and sent EMG data through BLE to control an armband.^1141^ Such hybrid systems can leverage the strengths of different wireless technologies based on application needs, such as using NFC for low-energy, short-range interactions and Wi-Fi for long-range, high-data applications.

In a real scenario of wearable applications, the decision between NFC and BLE methodologies depends on the specific use case. NFC excels in close-proximity, energy-efficient systems, particularly for disposable or semidisposable devices like those developed by Maroli et al.^858^ The integration of the SIC4341 potentiostat ensures compact and robust electrochemical sensing for applications such as POC diagnostics, where portability and simplicity are paramount. In contrast, BLE is more suited for applications requiring long-range, continuous monitoring. The LMP91000 potentiostat paired with BLE, as shown in Yang et al.’s work,^531^ provides greater flexibility for high-bandwidth data transmission and remote monitoring scenarios, such as fitness tracking or hospital-based multisensor networks. A comparison between these two methodologies is given in Table 5.

**Table 5:** Comparison between NFC and BLE with Specific Types of Devices

Feature	SIC4341 (NFC)	BLE with LMP91000
Range	∼10 cm	Up to 100 m
Power Source	Energy harvested from the reader	Battery required
Data Rate	∼424 kbps	Up to 2 Mbps
Integration	Single-chip system for simplicity	Two-chip system (BLE + potentiostat)
Energy Consumption	Extremely low (no battery needed)	Ultralow (long battery life)
Cost and Complexity	Low	Moderate
Security	High (limited range)	Moderate to Low (extended range)
Use Cases	Disposable diagnostics, patch biosensors	Fitness trackers, remote health monitors

In summary, integrating wearable sensors with wireless communication modules such as NFC, Bluetooth, and Wi-Fi presents challenges in miniaturization, power consumption, security, and standardization. The foremost challenge lies in designing ultracompact wearable devices without compromising their functionality. Additionally, the high-power consumption of Bluetooth and Wi-Fi limits the battery life, making it difficult to maintain reliable data transmission under stringent power constraints. To address this, low-power communication protocols, such as BLE, and advanced energy-harvesting technologies should be investigated to prolong the battery life. Furthermore, signal interference in densely populated environments, coupled with environmental factors such as sweat and movement, can degrade the transmission reliability. Implementing interference management strategies, including frequency hopping and dual-band systems, may help mitigate these issues.^1142,1143^ Robust encryption protocols and secure authentication mechanisms are also essential to protect sensitive health data.^1144^ Developing standardized, modular designs and employing precertified modules can enhance compatibility with diverse healthcare systems and streamline adherence to regulatory requirements.

### 10 Energy Harvesting for Self-Powered Wearable Sensors

Harvesting energy from the human body or the environment to power wearable sensors is a promising solution for extending the operational time of the current devices. This approach eliminates the need for frequent battery replacement or charging, enhancing the convenience and willingness to use such devices. More importantly, it enables continuous and long-term collection of human health data, achieving around-the-clock health management. Several energy-harvesting technologies have been developed around the three primary energy sources available from the human body: mechanical energy from the movement of organs or limbs, thermal energy from body heat or breath, and chemical energy, such as from sweat glucose (Figure 50). Each of these technologies has its own unique application scenarios, which will be detailed below.

**Figure 50:**
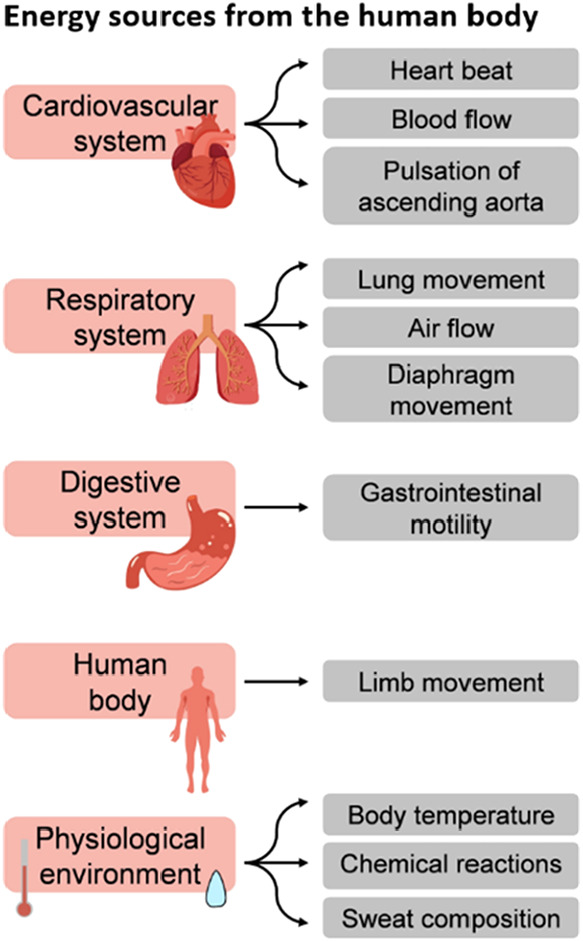
Energy sources from the human body.

#### 10.1 Mechanical Energy Harvesting

##### 10.1.1 Triboelectric Nanogenerators

The TENG is a mechanical-electrical energy conversion device that combines contact electrification with electrostatic induction to achieve high material compatibility, high cost-effectiveness, and the ability to capture ultralow-frequency and atypical mechanical energy.^1145–1151^ Because TENGs are probably the most used devices for energy harvesting for self-powered sensors, a longer description will be provided compared to the other harvesting mechanisms. The working principle of TENGs mainly relies on the coupling of contact electrification and electrostatic induction, as shown in Figure 51. When two materials with different triboelectric polarities come into contact and then separate, electrons will transfer between them due to the difference in their ability to gain or lose electrons. This leads to the generation of an electric potential difference between the electrodes attached to these materials. When an external circuit is connected, electrons will flow through the circuit to balance the potential difference, thus generating an electrical current. For example, in a typical vertical contact-separation mode of a TENG, initially, there is no charge on the uncontacted surfaces of the dielectric materials (such as PMMA and commercial polyimide (Kapton)). Under external force, contact between these surfaces results in the generation of equal amounts of heterogeneous charge. When the external force is withdrawn, a potential difference forms between the two electrodes in the open-circuit state, and the voltage increases with the separation distance until saturation. When the external force is reapplied, and the materials gradually come back into contact, the heterogeneous charges on the surfaces are gradually neutralized, causing the potential difference between the two electrodes to disappear gradually.

**Figure 51:**
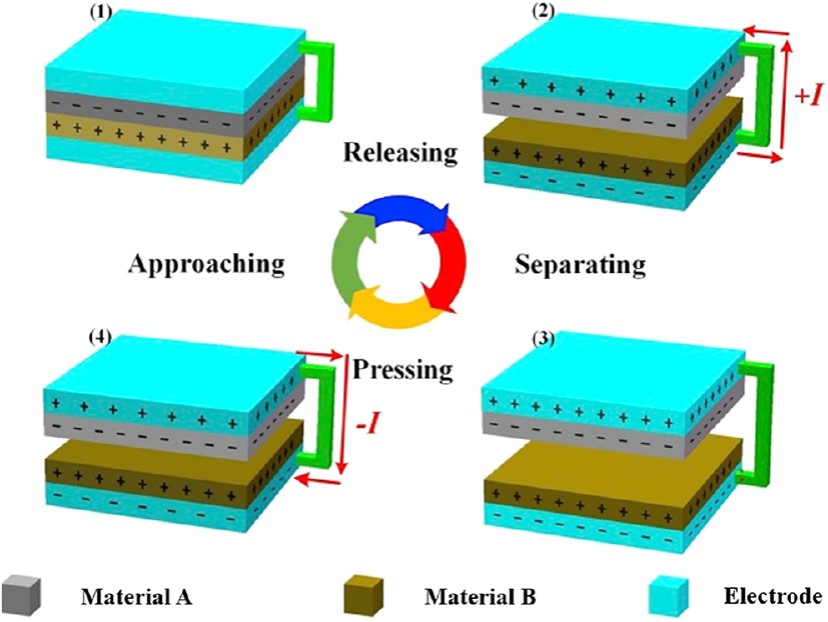
Schematics of the working principle of TENGs. Reproduced with permission from Xia et al.^1152^ © 2019 Elsevier.

There are four main operating modes for TENGs in common use today, as indicated in Figure 52A: vertical contact-separation mode, single-electrode mode, lateral sliding mode, and free-standing mode. TENGs for wearable sensors can have diverse structures depending on the specific application requirements. One common structure is the planar or sandwich structure, where the triboelectric layers are stacked one on top of the other with electrodes on either side to collect generated charges. In a simple pressure-sensing wearable TENG, a flexible polymer layer may act as one triboelectric material and a thin metal film as the other, with the electrodes integrated into the substrate. Another structure is the fiber- or textile-based structure, which is suitable for seamless integration into clothing for wearable applications. This allows for comfortable and unobtrusive wear while being able to detect various body movements and pressures. There are also more complex structures such as multilayer or hybrid structures. Multilayer TENGs can enhance the electrical output by increasing the number of contact-separation interfaces. Hybrid structures may combine TENGs with other functional components such as piezoelectric elements or energy storage units to improve the overall performance and functionality of the wearable sensor. For example, integrating a piezoelectric layer with a TENG layer can enable the sensor to detect both the mechanical pressure and vibration simultaneously.

**Figure 52:**
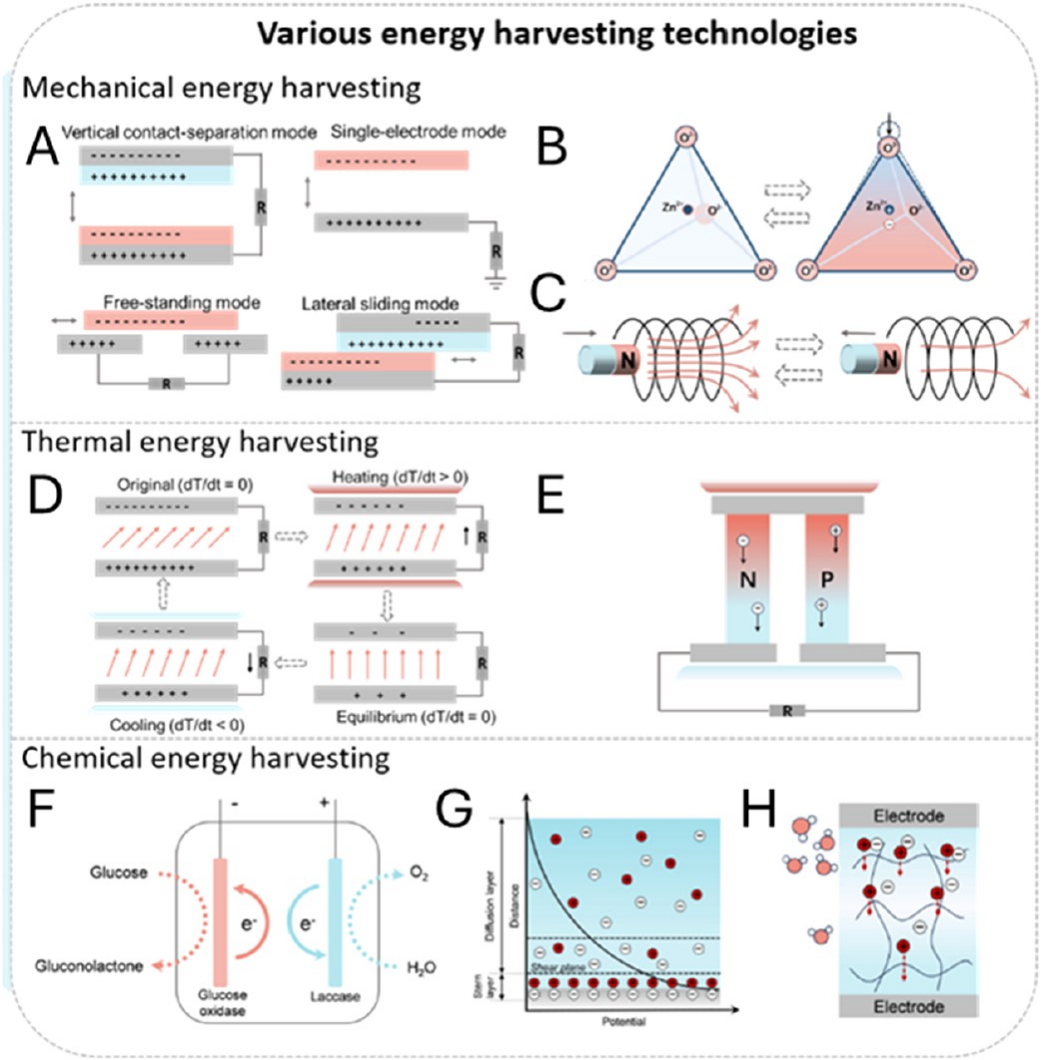
Emerging energy harvesting technologies. (A) Triboelectric nanogenerator. (B) Piezoelectric nanogenerator. (C) Electromagnetic generator. (D) Pyroelectric nanogenerator. (E) Thermoelectric generator. (F) Biofuel. (G) Hydrovoltaic effect generators. (H) Moisture effect generators.

A wide range of materials can be used in TENGs for wearable sensors. Polymer materials are popular due to their flexibility, lightweight nature, and ease of processing. PTFE is a commonly used polymer with excellent triboelectric properties. Other polymers like PET, PI, and PDMS are also frequently employed. Inorganic materials are also used, including metals like aluminum and copper, and nanomaterials such as carbon nanotubes and graphene. They can help with improving the charge transfer efficiency and the overall performance of the sensors. Composite materials have also gained prominence. For instance, a composite of a polymer with embedded inorganic fillers can achieve a balance between flexibility and enhanced triboelectric performance. Furthermore, biobased and biodegradable materials are being explored for more environmentally friendly and biocompatible wearable TENGs, especially in applications with long direct contact with the skin, such as in healthcare monitoring.

##### 10.1.2 Piezoelectric Nanogenerators

The operating principle of piezoelectric nanogenerators (PENGs) is the piezoelectric effect. When an external force acts on a piezoelectric material, the anions and cations in the lattice will be displaced from each other, resulting in the centers of the positive and negative charges no longer coinciding and thus generating an electric dipole moment. The superposition of the electric dipole moment is macroscopically manifested as a difference in the potential distribution of the entire material in the direction of the external force (Figure 52B). Typical piezoelectric materials include inorganic materials such as zinc oxide (ZnO), lead zirconate titanate (PZT), and barium titanate, as well as some organic materials, such as polyvinyl chloride, polyvinylidene fluoride, and its derivatives.^1153^

##### 10.1.3 Electromagnetic Generators

The basic principle of electromagnetic generators (EMGe) is to convert kinetic energy into electrical energy by using the law of electromagnetic induction. Since the invention of the first alternator in 1866, EMGe has been essential for the progress of human society and is a core pillar of modern energy. EMGe is generally composed of a stator, rotor, end-caps, bearings, and other components. Driven by external forces, the rotor rotates in the stator, making the movement of cutting the magnetic inductance (Figure 52C) due to the law of electromagnetic induction to generate an induced electric potential, thus generating a current in the external circuit.^1154^

TENG, PENG, and EMGe are based on different operating principles, but they all share the common characteristic of converting mechanical energy to electrical energy. The output of TENG is characterized by high voltage and low current, while the output of EMGe is high current and low voltage, and the output voltages and currents of PENGs are both moderate. The three have similar application scenarios and complementary advantages, and either individually applied or composite devices have important roles in the field of wearable devices.

#### 10.2 Thermal Energy Harvesting

##### 10.2.1 Pyroelectric Nanogenerators

The pyroelectric nanogenerator (PyNG) is an energy-harvesting device that utilizes materials with a pyroelectric effect to convert thermal energy into electrical energy.^1155,1156^ The pyroelectric effect refers to the change of the spontaneous polarization of certain crystals when they are heated to different temperatures. The working principle of the PyENG may be explained as follows. When the temperature is constant, the crystals have a stable strength of spontaneous polarization; when the temperature increases or decreases, it will lead to a decrease or increase in the strength of spontaneous polarization, which induces a change of charge on the electrodes and causes the generation of a pyroelectric current in the circuit (Figure 52D).

##### 10.2.2 Thermoelectric Generators

Thermoelectric generators (TEGs) are based on the Seebeck effect, which converts the energy present in a temperature gradient into electrical energy. The Seebeck effect comes from the movement of carriers within a conductor under a temperature gradient, and when there is a temperature difference between the two ends of the loop formed by the two conductors, there is a potential difference and a current.^1157,1158^ A commonly used TEG, shown in Figure 52E, consists of an N-type semiconductor, a P-type semiconductor, and electrodes. The temperature difference causes the directional movement of electrons and holes in the two semiconductors, which produces a DC output in the circuit.

Both PyENG and TEG can convert thermal energy into electrical energy, but the difference is that the former utilizes thermal energy that varies over time, and the latter utilizes thermal energy that varies over space. In addition, PyENG has a larger output voltage and smaller current, with its output depending on the rate of temperature change. This is suitable for environments with rapid temperature fluctuations. On the other hand, TEG has a larger current, a relatively high power density, and is easy to output in series, but it requires a stable heat source and heat dissipation conditions. Both technologies can be used to collect human body heat to power wearable devices.

#### 10.3 Chemical Energy Harvesting

##### 10.3.1 Biofuel Cells

A BFC can generate electricity by obtaining biochemical energy from the environment of the living organisms. BFCs generate electricity through redox reaction mechanisms and can be categorized into enzyme fuel cells and microbial fuel cells, characterized by their mild actuation conditions and being environmentally friendly and sustainable. Figure 52F shows a typical glucose biofuel cell using GOx as a catalyst, where glucose is oxidized to gluconolactone at the anode, while oxygen reduction takes place at the cathode.^1159^ In addition to glucose, BFC can utilize substances such as lactic acid, urea, vitamin C, and ethanol, to generate power for wearable devices.^1160^

##### 10.3.2 Hydrovoltaic Effect Generators

The hydrovoltaic effect generator (HEG) was proposed in 2017 after the discovery that the evaporation of water from a carbon black sheet could produce a sustained voltage of up to 1 V.^1161^ The materials used for HEG expanded to include natural materials such as bacterial membranes, wood, and lotus leaves as well as various organic or inorganic materials such as MXene, ZnO, and Si NWs. It is now believed that the principle of the HEG is the kinetic effect. The pressure gradient caused by the evaporation of water in the nanomaterials drives the flow of water in the nanochannels, causing the diffusion layer in the overlapping bilayers to move from upstream to downstream, which in turn creates a potential difference (Figure 52G).

##### 10.3.3 Moisture Effect Generators

The moisture effect generator (MEG) relies on the dissociation of free ions from functional groups of hygroscopic materials after absorbing moisture from the environment.^1162^ The ion concentration gradient prompts the directional diffusion of ions, generating a significant built-in electric field that results in the formation of a potential difference between the two electrodes (Figure 52H). The ionic concentration gradient is an essential principle of power generation. There are three ways to construct an ionic concentration gradient within a material: asymmetry in the chemical structure of the material, asymmetry in the distribution of moisture due to the asymmetry of the electrodes, and a nonhomogeneous structure of the assembled material.^1163^ Compared to HEG, MEG does not require bulk water sources and only utilizes moisture in the air, which may further increase the portability of wearable devices and broaden their application scenarios.

The above three energy-harvesting technologies for chemical energy all have superior DC output characteristics. Moreover, BFC can directly utilize chemical energy from human metabolites, and HEG and MEG can convert water energy in the environment to electrical energy for wearable devices, all three of which have the potential for clean, safe, and sustainable development.

#### 10.4 Self-Powered Sensing Strategies

The characteristics of the energy-harvesting technologies described should be considered in applying them to distinct wearable health-monitoring devices. Factors such as the monitored body part, environmental conditions, and performance must be accounted for. There are two basic self-powered-sensing strategies. The first approach is to construct self-powered sensing systems based on energy harvesting techniques and power management strategies. The second approach involves the direct construction of self-powered sensors using energy conversion devices. The strategy of the self-powered sensing system is to integrate an energy harvester, energy storage unit, and power management unit (PMU) to ensure a stable power supply for the sensors. This method is advantageous for active sensors that require higher or more stable power input. A PMU typically consists of components, such as rectifiers and voltage regulators. These components convert the alternating current generated by energy harvesters (such as TENGs and PENGs) into direct current and then store it in capacitors or batteries. The PMU also ensures that the stored energy is delivered to the sensor as needed, providing a steady power supply. This strategy is especially beneficial when the harvested energy is insufficient, as it helps smooth out energy fluctuations. By integrating energy storage and regulation, the PMU-based approach maintains the balance between energy production and consumption, ensuring efficient sensor operation over time.

A typical case of these strategies in wearable health monitoring is sweat analysis. For instance, Song et al. developed a wearable sweat sensor system powered by a TENG (Figure 53A).^487^ The system incorporates a PMU that stores harvested energy and supplies it to the sensor for the real-time monitoring of biomarkers like pH and sodium. The design of a low-power wireless sensor circuit and flexible PCB makes it suitable for continuous monitoring under comfortable wear. Gai et al. designed a wearable sweat analysis system that uses a hybrid nanogenerator module to harvest energy from human motion (Figure 53B).^1164^ The stored energy is used to power sensors for real-time analysis of biomarkers such as Na^+^ and K^+^. Ding et al. demonstrated a wearable system powered by a biofuel cell that harvests energy from sweat.^1165^ The energy is stored in a microgrid system, which powers a range of sensors for continuous metabolic sensing, efficiently monitoring metabolites such as glucose and vitamin C.

**Figure 53:**
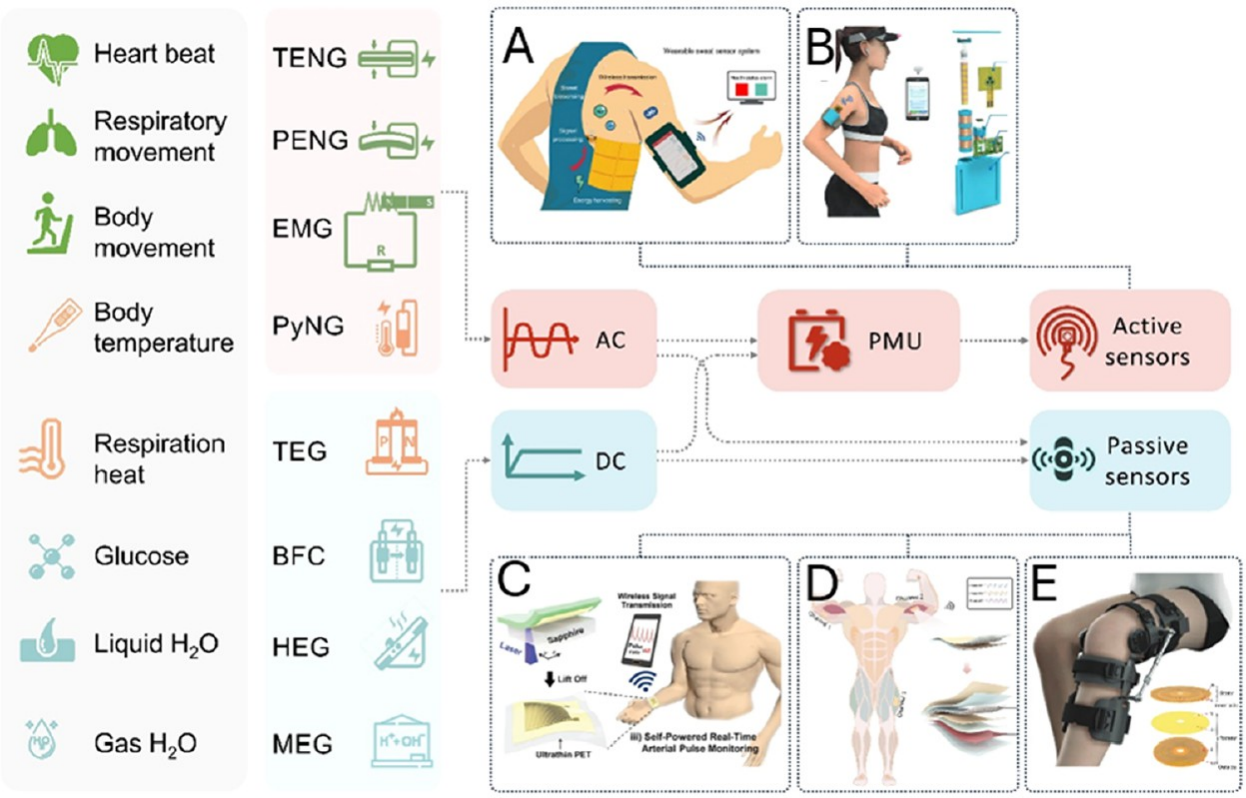
Self-powered wearable devices for health monitoring. (A) Wearable sweat sensor system powered by TENG. Reproduced from Song et al.^487^ Available under a CC BY-NC license. © 2020 American Association for the Advancement of Science. (B) Hybrid nanogenerator-based sweat analysis system. Reproduced with permission from Gai et al.^1164^ © 2022 John Wiley & Sons. (C) Self-powered PENG sensor for measuring arterial pulses. Reproduced with permission from Park et al.^1166^ © 2017 John Wiley & Sons. (D) Self-powered TENG sensor for monitoring muscle activity. Reproduced from Wang et al.^1167^ © 2021 American Chemical Society. (E) TENG-based wearable knee rehabilitation sensor for tracking joint angle. Reproduced from Luo et al.^1168^ Available under a CC BY 4.0 license. © 2022 John Wiley & Sons.

Another strategy is to construct self-powered sensors that convert environmental and biological physical or chemical changes directly into electrical signals, without the need for external power. Most energy harvesters can also serve as self-powered sensors themselves and work in the form of passive sensors, directly converting the human body’s respiration, pulse, body temperature, and sweat components into electrical signals that can be analyzed. The sensitivity of these sensors is positively correlated with the electrical output, which means the higher the output signal, the greater the sensitivity. Unlike other types of sensors, where higher sensitivity requires higher energy consumption, self-powered sensors achieve high sensitivity without additional power consumption. Therefore, the self-powered capability of these sensors is crucial for balancing ultrahigh sensitivity with low power usage, making them ideal for applications that require high performance with minimal energy demands. For example, Park et al. developed a self-powered piezoelectric pulse sensor that converts mechanical energy from arterial pulse waves into electrical energy (Figure 53C).^1166^ This sensor, made from an inorganic piezoelectric material, measures pressure pulses in the radial and carotid arteries and wirelessly transmits the data to a mobile device. Wang et al. proposed a skin-mounted electrostatic sensor that can obtain muscle information from skin deformation by detecting signals from both the biceps and triceps (Figure 53D).^1167^ Luo et al. introduced a wearable self-powered knee rehabilitation sensor embedded in a knee brace to track muscle strength and joint angles in knee replacement patients (Figure 53E).^1168^ Zou et al. reported a stretchable self-powered respiratory sensor inspired by the shark gill with a PENG and a TENG combined in bionic structures.^1169^

Triboelectric nanogenerators offer a promising alternative as they can harvest energy from body movements and convert it into electrical signals for sensing purposes.^1147–1151^ This self-powering capability is crucial for wearable sensors as it eliminates the need for frequent battery replacements and enables seamless integration into clothing or accessories for continuous monitoring.

##### 10.4.1 Pressure-Sensing Applications

TENGs have remarkable potential in wearable pressure-sensing applications because of their ability to convert mechanical pressure *stimuli* into electrical signals. For instance, Ouyang et al. (2017) developed a flexible self-powered ultrasensitive pulse sensor (SUPS) based on a triboelectric active sensor for cardiovascular disease diagnostics (Figure 54).^1170^ The SUPS was designed with nanostructured Kapton and Cu films as triboelectric layers. When pressure was applied, such as when placed on the radial artery, contact electrification and electrostatic induction mechanisms came into play. As the external force was applied and released, electrons flowed back and forth through the external circuit, generating an electrical signal that was modulated by the force. The SUPS could detect the pulse pressure and provide reliable information on the cardiovascular system. It had excellent output performance with an open-circuit voltage of 1.52 V, a high peak signal-to-noise ratio of 45 dB, and a long-term stability over 107 cycles. Another example is the work of Xia and co-workers. (2019),^1152^ where a tea leaf triboelectric nanogenerator using aluminum plastic bags (TAP-TENG) was fabricated (Figure 55). The TAP-TENG was designed with a unique structure that could respond to different pressures *stimuli*. When used in an office environment, it could detect various human touching states like knocking, pressing, and stroking. The different pressure magnitudes and durations during these actions resulted in different electrical output signals. For example, when the honeycomb lantern TAP-TENG structure was integrated into a book, the turning of pages exerted different levels of pressure on the TENG, and it was able to harvest the mechanical energy generated by these actions and convert it to electrical energy. This indicates that TENGs can be applied in scenarios in which the detection of variable pressure inputs is required for interactive or monitoring purposes.

**Figure 54:**
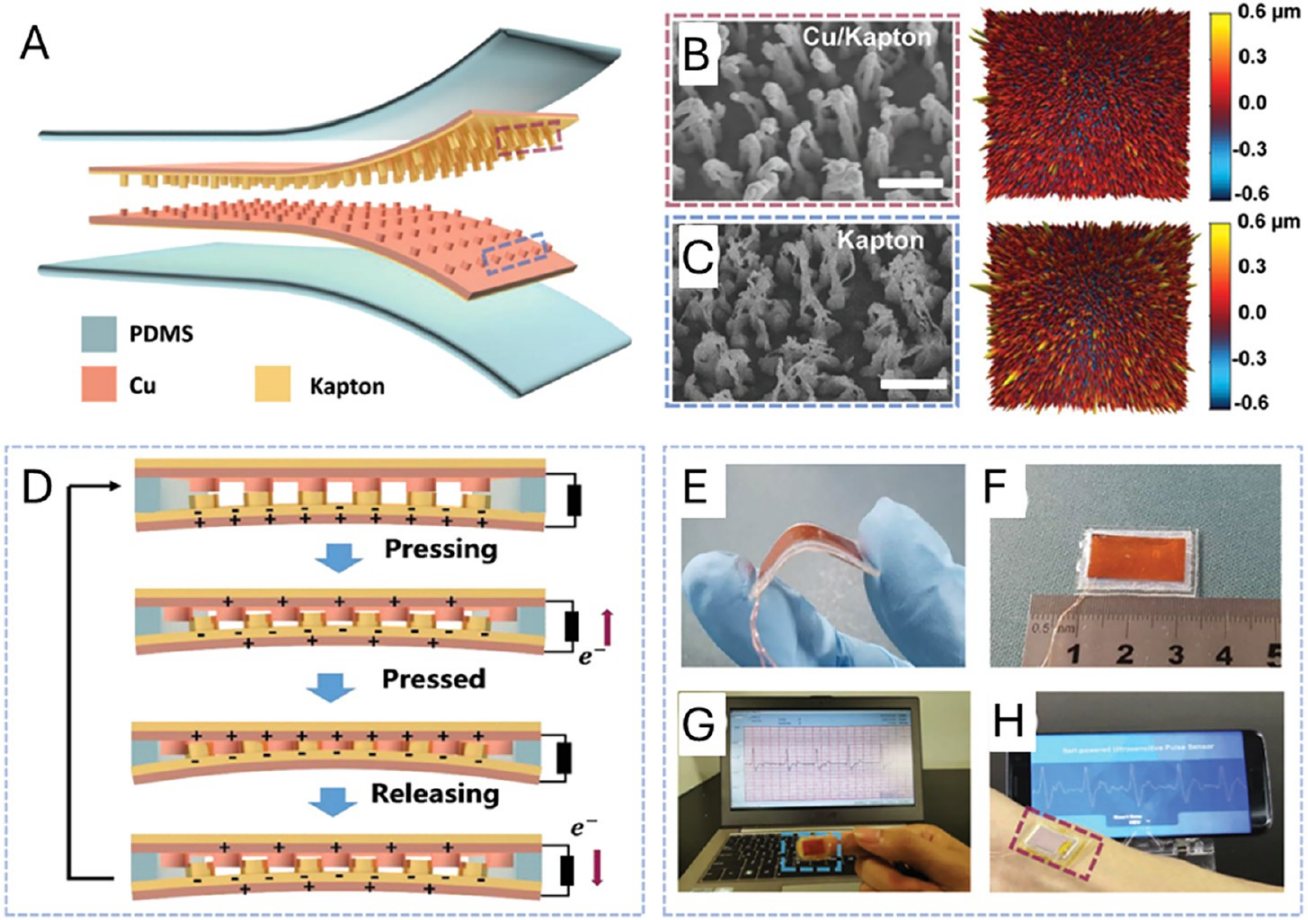
Structure, composition, working principle, characteristics, and signal output of SUPS. (A) Schematic representation of the device, showing each material layer. Scanning elecron microscopy (SEM) and atomic force microscopy (AFM) from (B) Cu and Kapton film and (C) Kapton film. (D) Schematics of the basic working mechanism of the device. (E) Picture showing the flexibility of the device; (F) Picture showing the size of the built device; and a sample signal when the sensor is placed on top of the (G) finger and (H) radial artery of a patient. Reproduced with permission from Ouyang et al.^1170^ © 2017 John Wiley & Sons.

**Figure 55:**
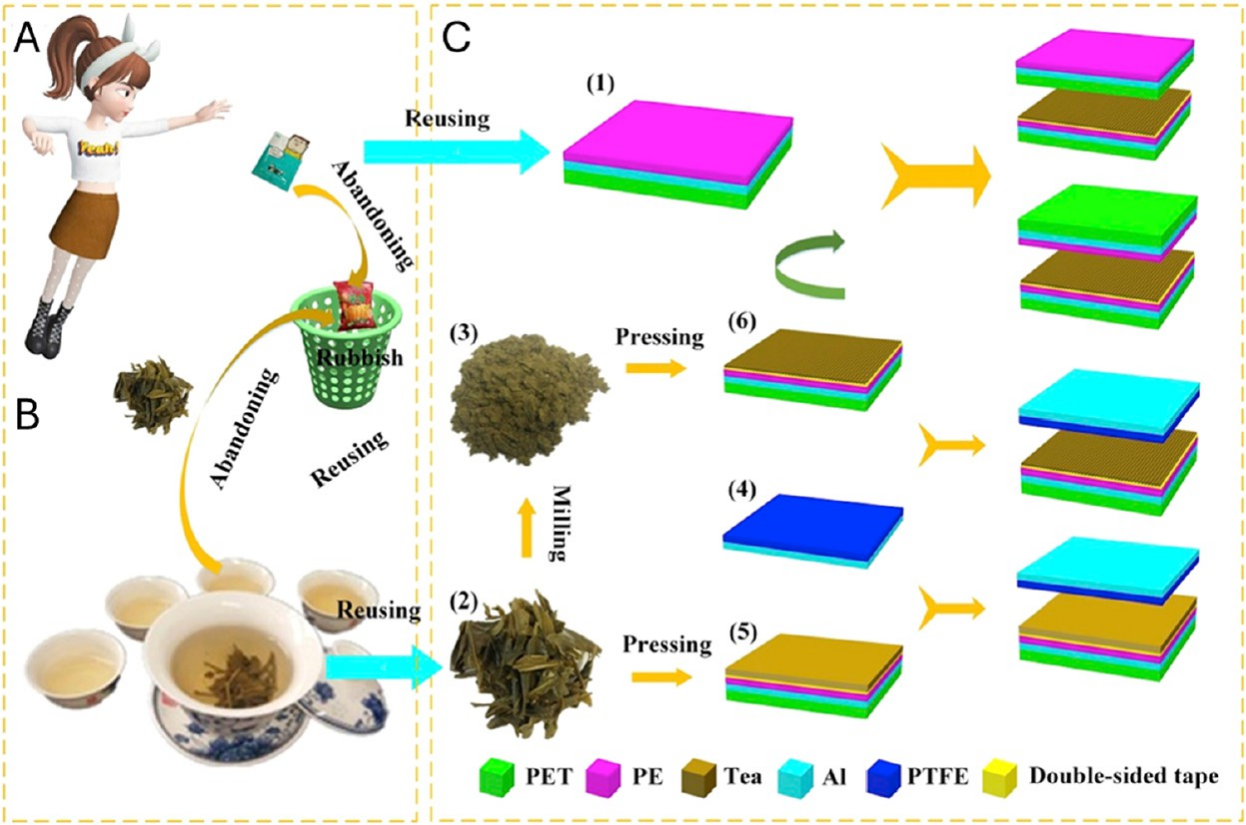
Scheme showing the preparation process of TAP-TENG. Reuse of (A) aluminum plastic bags and (B) tea leaves. (C) Processing of materials. Reproduced with permission from Xia et al.^1152^ © 2019 Elsevier.

Materials and structures have been optimized to enhance the sensitivity and range of pressure detection using TENGs. For example, nanostructuring triboelectric layers can increase the effective contact area during pressure application, thereby enhancing the charge transfer and improving the electrical output signal strength. This enables the detection of even very subtle pressure changes typical of gentle touches in wearable haptic feedback systems or monitoring the light pressure exerted by body movements in smart clothing for health and fitness tracking.

##### 10.4.2 Acoustic Sensing Applications

TENGs are promising for acoustic sensing in wearable devices. In audible sound sensing, TENGs can function as self-powered acoustic sensors by converting acoustic vibrations in the audible frequency range (20–20,000 Hz) into electrical signals.^1171^ The contact-separation mode of TENGs plays a vital role here. When sound waves impinge on the triboelectric layers of the TENG, they cause periodic contact and separation of the layers. For instance, in a simple dielectric-dielectric contact-separation TENG setup, as sound waves exert pressure on the materials, the resulting mechanical deformations lead to the generation of charges due to triboelectrification and subsequent electrostatic induction-driven flow of electrons in the external circuit. This enables the detection of sound signals. TENG-based sensors have been investigated for hearing aids owing to their miniature structure and because they do not require an external power supply. They can capture ambient sounds and convert them into electrical signals to be further processed and enhance the hearing experience for individuals with hearing impairments. Compared with traditional piezoelectric-based acoustic transducers used in some hearing aids, TENGs have the advantages of higher output power density under certain conditions and are less likely to be affected by interference in some frequency ranges. This enables them to provide a more stable and clear sound amplification effect for hearing aid users. In cochlear implants, TENG-based systems can take advantage of the body’s mechanical movements or acoustic vibrations to generate electricity.^1172,1173^ For example, when a person is talking, walking, or in an environment with ambient sound, the TENG integrated into the cochlear implant can convert these mechanical *stimuli* into electrical signals. The miniature structure of TENGs allows them to be implanted in a relatively small space within the ear, and their ability to work without an external power supply makes them more suitable for long-term use. The acoustic energy from the external environment can drive the contact separation or other working modes of the TENG, generating electrical signals that can be further processed and used to stimulate the auditory nerve, thereby helping patients with hearing impairments to perceive sound.

##### 10.4.3 Ultrasonic Sensing Application

TENGs can be used for ultrasonic sensing,^1174^ since frequencies higher than 20,000 Hz have unique characteristics to be harnessed by such technology. They may be used for noninvasive or minimally invasive monitoring, as in the diagnosis of certain internal organ conditions. Ultrasound waves are sent into the body, and the TENG-based sensors can detect the reflected signals. Yang et al. developed a flexible, ultrawideband triboelectric ultrasonic device (FUTUD),^1175^ as shown in Figure 56. The FUTUD was integrated with a flexible electromagnetic coil to transmit electrical signals containing physiological information from *in vivo* to *ex vivo* wirelessly as a passive wireless sensor. The mechanical vibrations caused by the ultrasound on the TENG’s triboelectric layers result in charge generation and electrical signal output. By analyzing these signals, information about the structure and health of internal organs such as the liver, kidneys, or blood vessels can be obtained. This can aid in the early detection of diseases like tumors, cysts, or vascular disorders.

**Figure 56:**
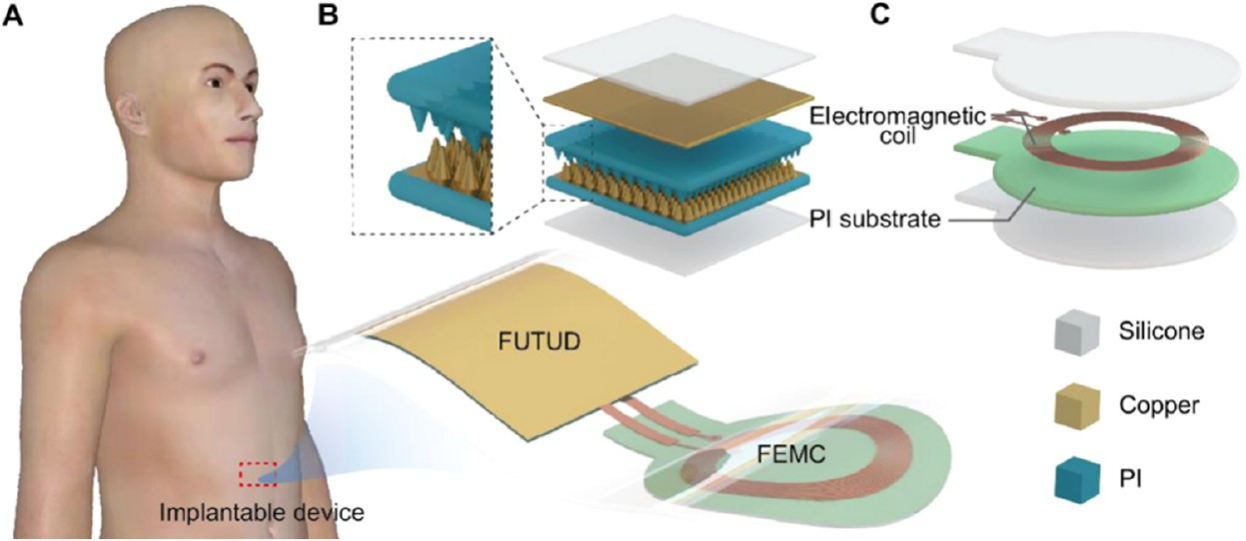
(A) Schematic representation of the acoustic device implanted into the human body. (B) Structure of FUTUD and of (C) FEMC. Reproduced with permission from Sun et al.^1175^ © 2023 Elsevier.

Ultrasonic sensing using TENGs can be integrated into patches or bands to monitor ultrasonic signals related to physiological processes. For instance, they can detect changes in blood flow in deep tissues by sensing ultrasonic echoes. This can provide valuable data on cardiovascular health and help in the long-term management of conditions such as hypertension or atherosclerosis. In addition to medical applications, in industrial and environmental monitoring scenarios, where wearable sensors are required, TENG-based ultrasonic sensors can be used. In industrial settings, they can detect flaws or cracks in materials by analyzing the ultrasonic reflections from the surfaces of the structures. Workers can wear these sensors as part of their personal protective equipment to quickly identify potential safety hazards in machinery and building components. In environmental monitoring, ultrasonic TENG sensors can be used to detect the presence of certain objects or changes in an underwater environment. For example, in marine research, they can help in tracking the movement of marine organisms or monitoring the health of coral reefs by sensing the ultrasonic signals that they emit or interact with. In conclusion, the ultrasonic sensing capabilities of TENGs in wearable sensors offer a wide range of applications that span multiple domains, contributing to improved health monitoring, industrial safety, and environmental understanding.

### 11 The Use of Machine Learning and AI for Data Analysis

Health monitoring is not a new concept. Recording symptoms for diagnosis can be traced back to ancient times, primarily through visual and tactile observations. Over the centuries, various tools and instruments have been introduced, including noninvasive technologies such as ECG, pulse oximeters, and wearable devices. What is remarkable in recent years is the convergence of two technologies that have been revolutionizing health monitoring. As amply discussed in this paper, wearable devices enable continuous, real-time tracking of health conditions, while AI methodologies are applied to process and interpret the vast amounts of data generated by continuous monitoring. In this section, we present several examples of how ML and other AI techniques are employed for health monitoring. Before delving into these examples, we introduce the basic concepts of these AI tools along with related topics in data analytics.

#### 11.1 Data Analytics and AI Tools

The analysis of the literature presented in Section 2 indicates that the use of ML and other AI methods for health monitoring has surged over the last 10 years, with most publications appearing within the past 5 years. However, ML has been employed to analyze sensing data for decades, often under the framework of artificial neural networks (e.g., electronic tongues^1176^). For wearable sensors, in particular, recent review papers have illustrated a variety of applications and challenges.^57^ The integration of ML and AI in sensing and biosensing has been extensively discussed, with many authors emphasizing their potential for transformative advancements in both materials science and AI. For instance, the central role of sensing and biosensing in the technological revolution driven by ML and Big Data has been associated with ubiquitous sensing, which is expected to transform various sectors.^1177^ Additionally, the application of ML in sensor and biosensor data analysis could be extended to encompass knowledge discovery, aiming for a paradigm shift toward machine-generated knowledge.^1178–1180^ This concept is now being realized through tools powered by large-language models. Since the principles and applications of ML and AI in sensing have been reviewed comprehensively in several papers,^57^ we provide only a concise overview of key definitions to help readers understand the descriptions and examples presented herein.

*Data analytics* refers to the systematic computational analysis and interpretation of data, focused on discovering, interpreting, and communicating meaningful patterns in data to guide decision-making. Data analytics directly intersects with several other key disciplines related to Mathematics, Statistics, and Computer Science, particularly ML in AI and Data Visualization.^1181^ ML uses algorithms and statistical models to identify patterns in data and make decisions with minimal human intervention. As such, it gives computers the ability to learn descriptive and predictive models from data without being explicitly programmed. Tom Mitchell, in his seminal book,^1182^ states that “A computer program is said to learn from experience E with respect to some class of tasks T and performance measure P, if its performance at tasks in T, as measured by P, improves with experience E.”

*Information visualization* has been defined as the study of visual representations of abstract data to reinforce human cognition and derive insights.^1183^ It focuses on the creation and optimization of computer-based interactive graphical representations to explore and understand the data. *Multidimensional Data Visualization* specifically refers to techniques and methods for visually representing data described by more than three dimensions or variables (hence referred to as multidimensional, or high-dimensional data), allowing users to explore relationships between multiple attributes simultaneously.^1184,1185^ *Visual analytics* has been defined as the science of analytical reasoning facilitated by interactive visual interfaces. It focuses on combining techniques from data visualization, ML, and statistics to enable users to explore, understand, and interpret complex data through visual representations to support decision-making by allowing users to identify patterns, trends, and insights in large and complex data sets.^1186^

A *dimension reduction* technique is a mathematical method that transforms high-dimensional data into a lower-dimensional form while preserving meaningful structures and relationships in the data, making it more manageable for analysis, processing by algorithms, and visualization.^1187^ A *multidimensional projection* technique is a specific type of dimension reduction method that maps high-dimensional data onto a lower-dimensional (typically 2D or 3D) visual space while attempting to preserve relative distances or relationships between data points, making complex data structures visually interpretable.^1188^ The key relationship with dimension reduction is that multidimensional projection is essentially dimension reduction with a specific focus on creating meaningful visual representations, while dimension reduction can be used for various purposes, such as preprocessing or computation efficiency of ML algorithms.

In ML, *supervised learning* refers to an approach where an algorithm learns to make predictions from a data set containing input-output pairs where the desired outputs (called labels or targets) are known. When these outputs are categorical (e.g., spam/not spam or dog/cat/bird/fish), the task is called classification. When the outputs are continuous numerical values (e.g., temperature and house prices), the task is called regression. In both cases, the algorithm learns to discover patterns in the input features that allow it to map new, unseen inputs to their likely outputs, attempting to generalize from the training examples that it was shown. SVM is a particular algorithm for classification that works by finding an optimal hyperplane in an N-dimensional space to distinctly separate data points into different classes by maximizing the margin between the nearest training points (called support vectors) of any class.^1189^ In *unsupervised learning*, an algorithm learns patterns, structures, or relationships without being given input-output pairs or predefined categories, discovering hidden patterns and groupings independently, directly from the data points.

*Deep learning* is a subset of machine learning that uses artificial neural networks with multiple layers (hence “deep”) to automatically learn representations and patterns from data. While loosely inspired by biological neural networks, modern deep learning architectures and algorithms are primarily mathematical/computational constructs that may work quite differently from the human brain. These models excel at discovering hierarchical features in data with each layer learning increasingly abstract representations. The approach has proved particularly effective for tasks like image and speech recognition, natural language processing, and computer vision components of autonomous driving systems.^1190^ CNNs are a class of deep learning architectures specifically designed for processing structured grid data, such as images. They use convolutional layers to automatically detect features (e.g., edges and textures) at various levels of abstraction, enabling the model to learn spatial hierarchies. CNNs are widely used in image and video recognition, natural language processing, and other computer vision tasks.^1191,1192^

*Explainable Machine Learning* (XAI) is the broad field focused on making AI systems understandable to humans. Explainable Machine Learning, in turn, is a subset of XAI that encompasses methods and techniques aimed at making ML systems’ decisions and behaviors understandable to humans. This includes both developing inherently interpretable models and creating tools to explain existing complex “black-box” models. The field addresses several key aspects: understanding how models arrive at specific decisions, identifying potential biases or failure modes, verifying compliance with regulations, and enabling meaningful human oversight. XAI techniques range from simple approaches like feature importance rankings to sophisticated methods that generate human-friendly explanations of model behavior through examples, rules, or visualizations.^1193,1194^ This is particularly relevant in practical application scenarios that require decisions to comply with fairness and safety principles, as well as with ethical and legal regulations.

Finally, *IoT* refers to the network of physical devices embedded with sensors, software, and other enabling technologies for collecting, exchanging, and processing data over the Internet. These interconnected devices allow for automation, remote monitoring, and improved decision-making across various industries, such as healthcare, transportation, and smart homes.^1195,1196^

The connection between (bio)­sensing and data analytics is crucial, as analytics transform raw sensor data into actionable insights. Sensor data are often unprocessed and require analytics to extract meaningful trends, patterns, and relationships. For example, a glucose sensor produces raw electrical signals that must be calibrated, processed, and interpreted to estimate the blood sugar levels. When large volumes of data need processing, dimensionality reduction techniques are often applied to high-dimensional data, e.g., measurements obtained from multimodal biosensors. Dimension reduction methods such as principal component analysis (PCA) or t-SNE are commonly employed for visualization and analysis, as the resulting visual representations depicted as point cloud similarity views can give insight into the distribution, grouping patterns, and outliers in multidimensional data. In similar scenarios, it is also possible to apply classification and clustering algorithms to the data to obtain explicit categories or groups. Data analytics form the basis for automated or assisted decision-making in sensing systems. For instance, an air quality monitoring system may trigger alerts when pollutant levels exceed predefined thresholds. Analytics also help optimize sensor performance, such as compensating for drift or identifying faulty sensors. Key processes in sensor data analytics include data preparation, which involves aggregating, cleaning, and transforming data for analysis as well as handling missing values or noisy data points during preprocessing. Visualization and statistical methods are employed to explore the data, understand their characteristics, and identify anomalies or trends in sensor readings. Finally, statistical models and machine learning algorithms are applied to analyze the processed sensor data and obtain prediction models such as regression models to predict temperature trends or classification models for biosensors that detect specific biomarkers.

The ability to perform classification tasks, essential for diagnosis and health monitoring, is a significant feature of ML in analyzing sensing data. Additionally, ML methods are highly versatile, capable of handling diverse data types and excelling in scenarios where synergy is achieved through data fusion from multiple sources and modalities. Whether the data signals are electrical, optical, mechanical, or thermal—or whether ML algorithms process inputs like images, text, or other formats—their adaptability remains consistent. For health monitoring, ML can be combined effectively with time-series analysis and pattern recognition techniques, e.g., applied to images. The application of AI methods to process multidimensional data captured by multimodal sensing systems has been reviewed by Mahato et al.^1197^ The authors outline several potential applications, as illustrated in Figure 57, which also includes a flowchart depicting data processing and the development of intelligent systems.

**Figure 57:**
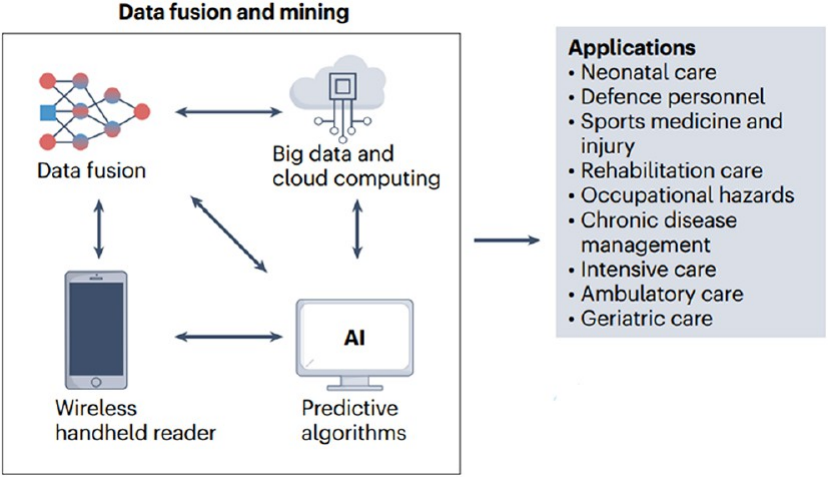
Flowchart illustrating the integration of data fusion with Big Data and cloud computing methodologies in developing intelligent systems capable of making predictions for diagnosis and health monitoring. These systems ideally employ hand-held readers, such as smartphones. A nonexhaustive list of potential applications is displayed in the box on the right. Adapted with permission from Mahato et al.^1197^ © 2024 Springer Nature.

#### 11.2 Uses of AI in Health Monitoring

The use of AI in health monitoring, particularly ML, is growing at such a fast pace that it has become unfeasible to provide an exhaustive account of recent developments. We have thus selected a few representative examples, focusing primarily on ML and pattern recognition as the key subfields of AI. While we may refer to AI and ML interchangeably in this context, we remind readers that ML comprises a subarea of AI.

Various types of physical sensors, particularly wearable strain and pressure sensors, are used to analyze gait patterns, which are relevant for health monitoring as they provide insight into an individual’s physical health, neurological condition, and overall functional ability. Gait patterns can serve as indicators of neurological diseases such as Parkinson’s disease and multiple sclerosis, with changes in stride length, walking speed, or symmetry often signaling neurological impairments. Gait analysis is also instrumental in fall risk assessments and patient rehabilitation monitoring. In many of these applications, AI is essential in data analysis. For example, Xiang et al.^1198^ developed an AI-assisted insole sensing system for real-time gait monitoring. This system integrates silicone rubber with PMMA optical fibers (S-POF), enabling the conversion of pressure measured at key points into changes in light intensity. The insole system and its operating principle are depicted in Figure 58.

**Figure 58:**
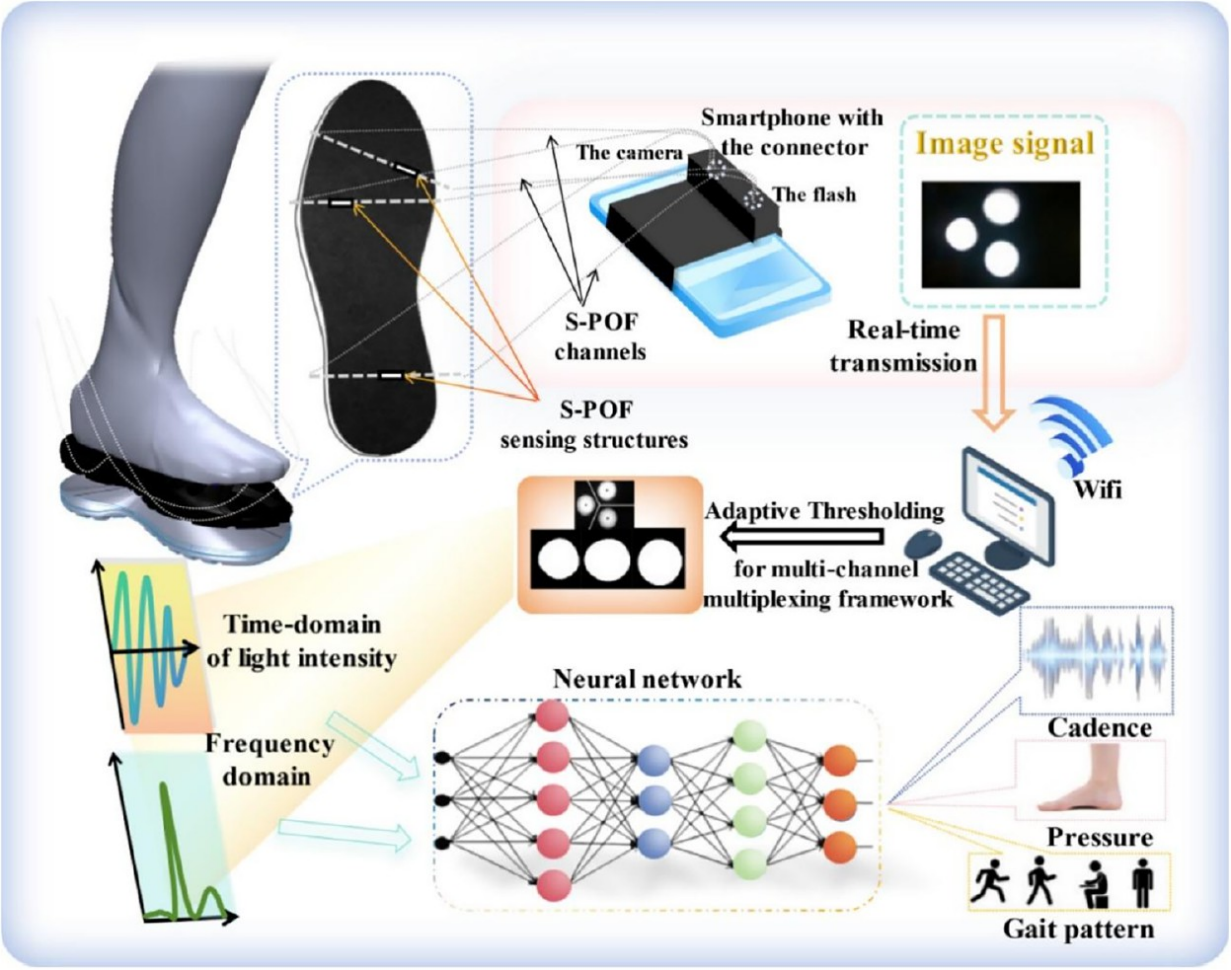
Insole system, positioned beneath the foot as shown in the top left of the figure, features 3 sensing channels (S-POF channels) embedded at strategic locations (heel and metatarsophalangeal joints). Plantar pressures are converted into light signals and transmitted via optical fibers to a smartphone camera, as illustrated in the central top portion of the figure. These image signals are processed in real time on a computer system, with adaptive thresholding applied as a preprocessing step. In the final stage, shown in the lower part of the figure, signals in both time and frequency domains are analyzed using neural networks to determine gait patterns (see further details below). Reproduced from Xiang et al.^1198^ © 2024 American Chemical Society.

One particularly interesting aspect of this example is that the solution developed integrates combined pressure sensing data and image processing. As we will discuss in the Prospects and Outlook section, combining ML with image analysis of sensing units has the potential to transform health monitoring. Such approaches could enable almost instrument-free strategies, requiring only cameras or microscopes that may be embedded into routine devices such as smartphones. In the aforementioned study,^1198^ signal processing and data analysis were performed through several steps. The first module acquires data from the camera, transmits it to the computer, and converts the signal to grayscale. Three spots represent data from the channels corresponding to three positions on the insole. Adaptive thresholds and image segmentation are used within a multiplexing framework for the grayscale summation matrix to be processed. To extract gait information from each of the three channels individually, filtering and power-spectrum transformation are applied. These processed signals are then input into a neural network algorithm, which predicts and differentiates gait patterns.

Details of the identification of gait patterns are provided in Figure 59. Three regimes of walking speeds were defined, as follows: (i) normal walking speed, between 1.33 and 1.67 m/s; (ii) slow walking, below 1.33 m/s; and (iii) fast walking, above 1.67 m/s. For running, the authors also established three regimes: normal running speed, within the range of 2.67 to 3.33 m/s, and slow and fast running speeds, defined as below and above this range, respectively. Figure 59a illustrates the experimental setup, where participants were asked to walk specific distance ranges within a fixed time (30 s, as shown in the figure) for 20 repetitions in each case. This procedure ensured the acquisition of sufficient data for subsequent classification. The data set comprised 1,000 data instances, corresponding to 5 subjects × 10 routines × 20 repetitions. Each data instance included three arrays of frequency values and three arrays of pressure values, derived from time-domain and frequency-domain measurements. The histograms in Figure 59b compare the accuracy of predicting gait patterns using five classification models: decision trees, random forests, K-nearest neighbors (KNN), SVM, and Bayesian neural networks. Neural networks achieved the highest performance, with an average accuracy rate of 90.62%, as reflected in the confusion matrix shown in Figure 59c. These neural networks were subsequently employed as the classification model for predicting gait patterns, as illustrated in Figure 59d.

**Figure 59:**
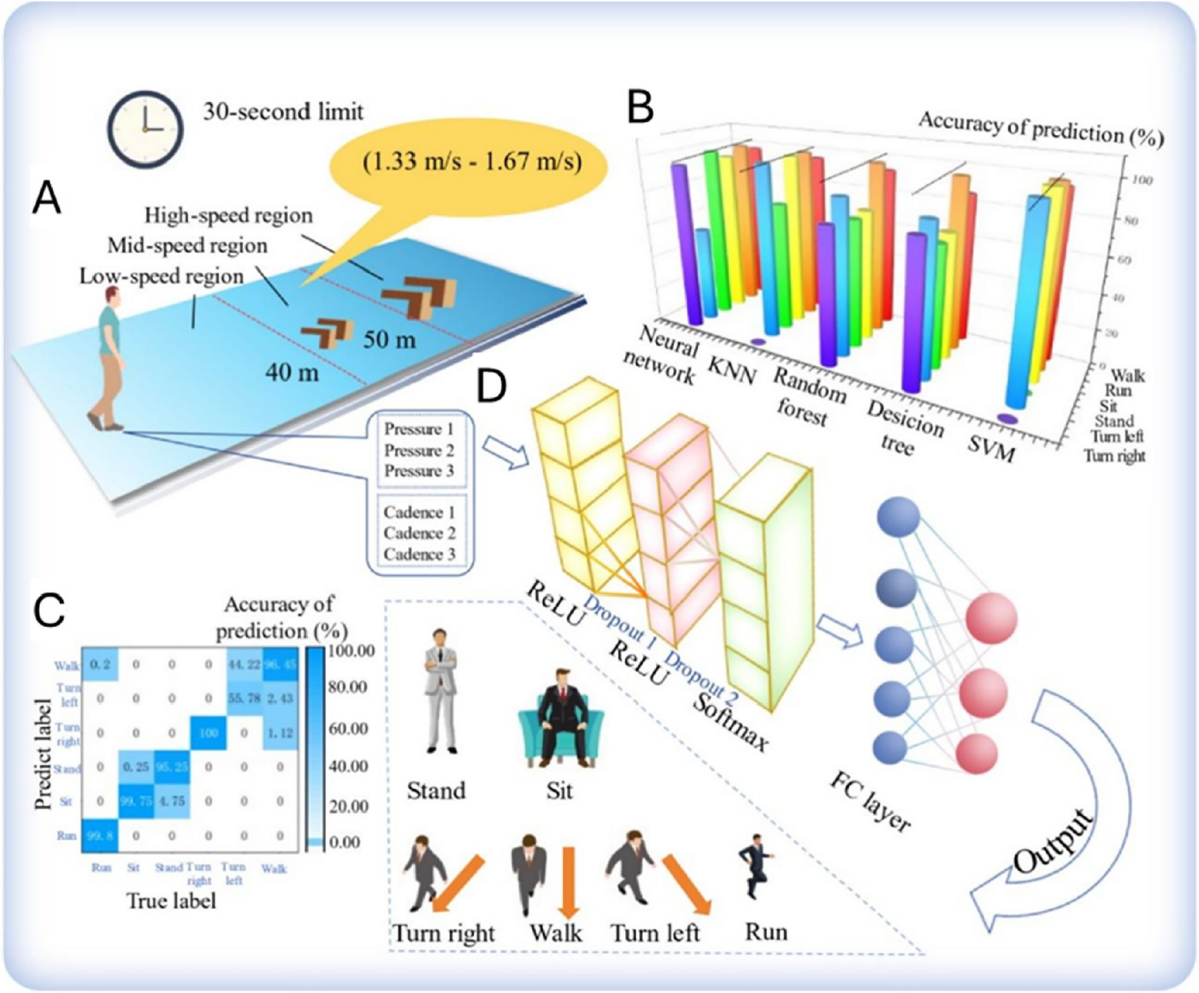
(A) Schematic illustration of the scenario for collecting image data on walking and running. Human subjects were instructed to walk or run varying distances within a fixed time period of 30 s. (B) Histograms showing the prediction accuracy of gait patterns using five ML algorithms. (C) Confusion matrix for gait pattern predictions made by neural networks. (D) Flowchart illustrating the process of gait pattern prediction using neural networks. Reproduced from Xiang et al.^1198^ © 2024 American Chemical Society.

The potential for continuous monitoring enabled by wearable pressure and strain sensors has spurred significant research into processing data and correlating them with various types of motion and health conditions. For instance, screen-printed strain sensors composed of a carbon-based conductive network on flexible TPU films were used to detect EMG and ECG signals.^1199^ Because these sensors are stretchable, they can conformally adhere to human skin, enabling long-term, continuous monitoring. The sensors are also capable of detecting various types of human motions, including joint movements, mouth opening, chewing, swallowing, breathing, and clenching. Figure 60 illustrates the electrical signals corresponding to several movements along with the confusion matrix generated from using an SVM algorithm to predict six types of knee movements.

**Figure 60:**
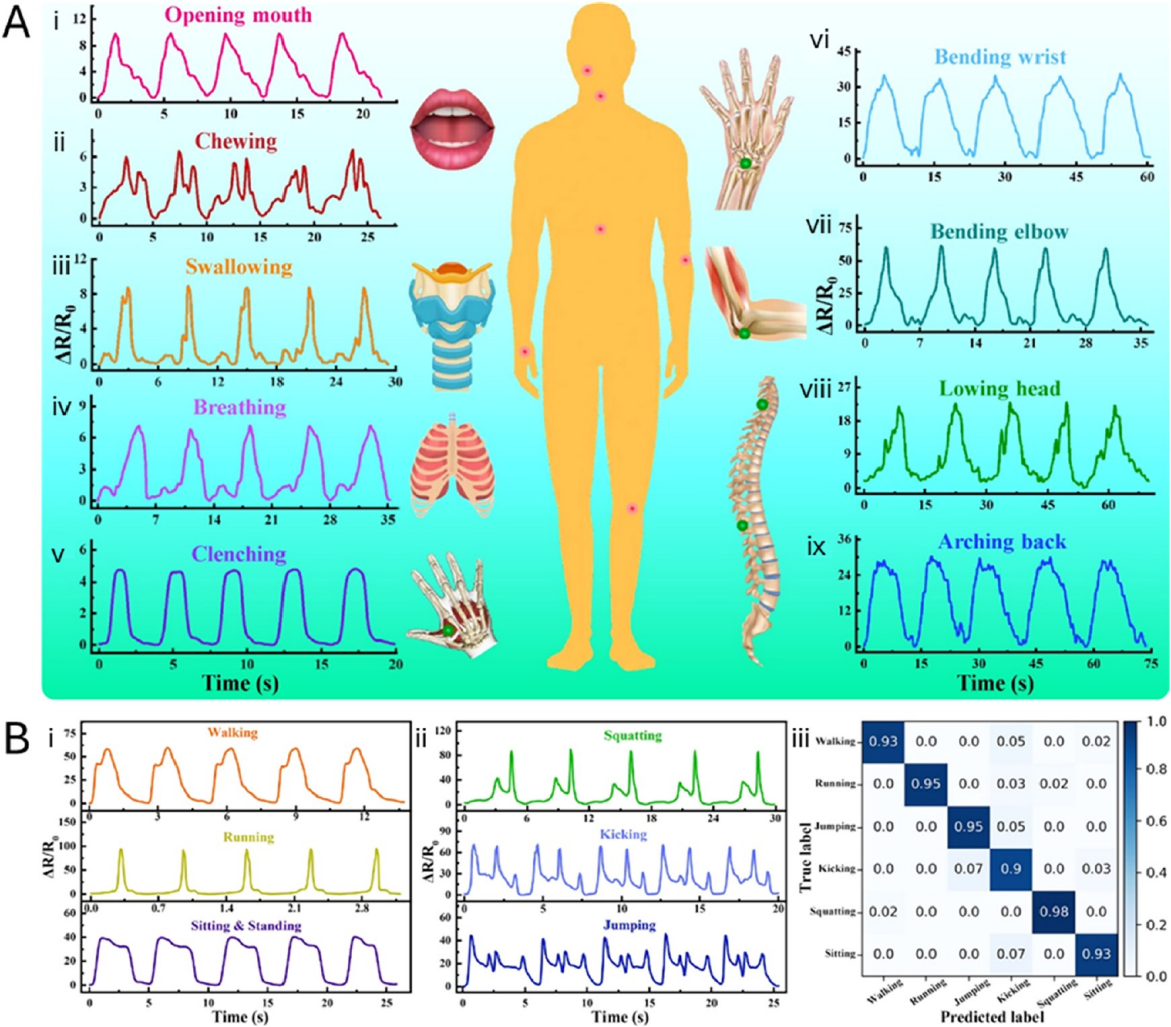
(A) Typical responses from the pressure sensors for various types of human motion (i–ix). (B) Responses related to knee motions shown in (i) and (ii) were classified using an SVM algorithm with a high accuracy, as indicated in the confusion matrix in (iii). Reproduced from Zhu et al.^1199^ © 2024 American Chemical Society.

Monitoring cardiovascular health has been a prominent topic, as highlighted in several of the previous sections. Due to the intrinsic nature of this type of monitoring—which involves multiple types of instruments and requires time-series analysis—ML is an obvious choice for developing next-generation diagnosis and monitoring systems. This concept has been explored by Wang et al.^58^ and is illustrated in their chart (reproduced in Figure 61), where standard monitoring methods are compared to AI-based approaches. Figure 61A depicts the manual collection and processing of patient data in traditional settings, whereas the AI-based counterpart is shown in Figure 61B. Significant differences are noted in data acquisition, as wearable devices afford continuous data collection, and in the data processing and diagnostic phases, where ML algorithms are applied. A variety of algorithms can be employed to classify or quantify samples, as outlined in the AI box in Figure 61, with the potential for expansion to include many other methods. Moreover, these algorithms can process diverse data types and are not restricted to those listed in the biosignal acquisition block. Most importantly, the outcomes from data processing can serve as inputs for intelligent systems, enabling functionalities beyond diagnostics, such as developing treatment plans, as exemplified in the AI-assisted diagnostics and treatment box in Figure 61.

**Figure 61:**
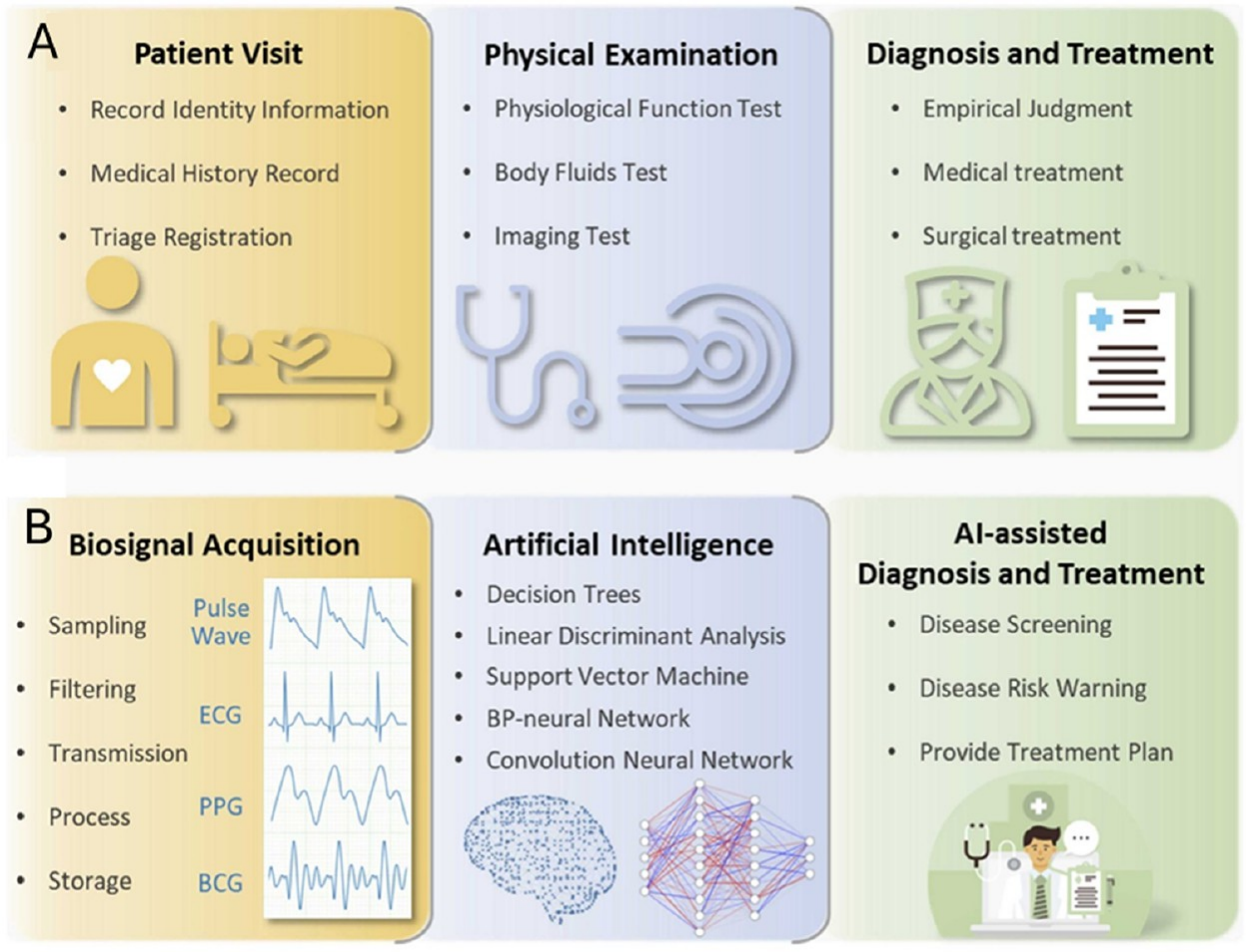
Boxes in the top row (A) represent traditional methods for diagnosis and treatment planning, which rely entirely on human decisions and interventions. Patient visits are followed by examinations, the results of which guide medical doctors in diagnosing conditions and recommending treatments. These decisions are based on patient data and the doctors’ experience. In contrast, the boxes in the bottom row (B) illustrate an AI-assisted approach to diagnosis and treatment planning. Data from biosignal acquisition are processed using AI tools, primarily ML, with the results informing AI systems to develop diagnoses and treatment plans. Reprinted with permission from Wang et al.^58^ © 2024 Elsevier.

In terms of data analysis, ML can be used in classifying data from varied sources, including pulse pressure, ECGs, and ultrasonography images. Figure 62 shows the design and functions of a soft wearable stethoscope (SWS) for cardiopulmonary auscultation.^1200^ The soft device whose photos are shown in Figure 62A comprises an elastomeric enclosure with a conductive hydrogel layer to auscultate cardiac and respiratory signals, as described in detail in Figure 62B. The latter shows the electronic components such as a microelectromechanical system (MEMS) microphone, a rechargeable battery, a Bluetooth for wireless transmission, and an electric circuit, in addition to the multiple layers of soft materials. Some of the stretching and bending characteristics of the SWS are shown in Figure 62C–F. The ML-based diagnosis system is depicted in Figure 62G, which indicates the flow for sound recording, preprocessing with denoising, and ML classification using CNNs.

**Figure 62:**
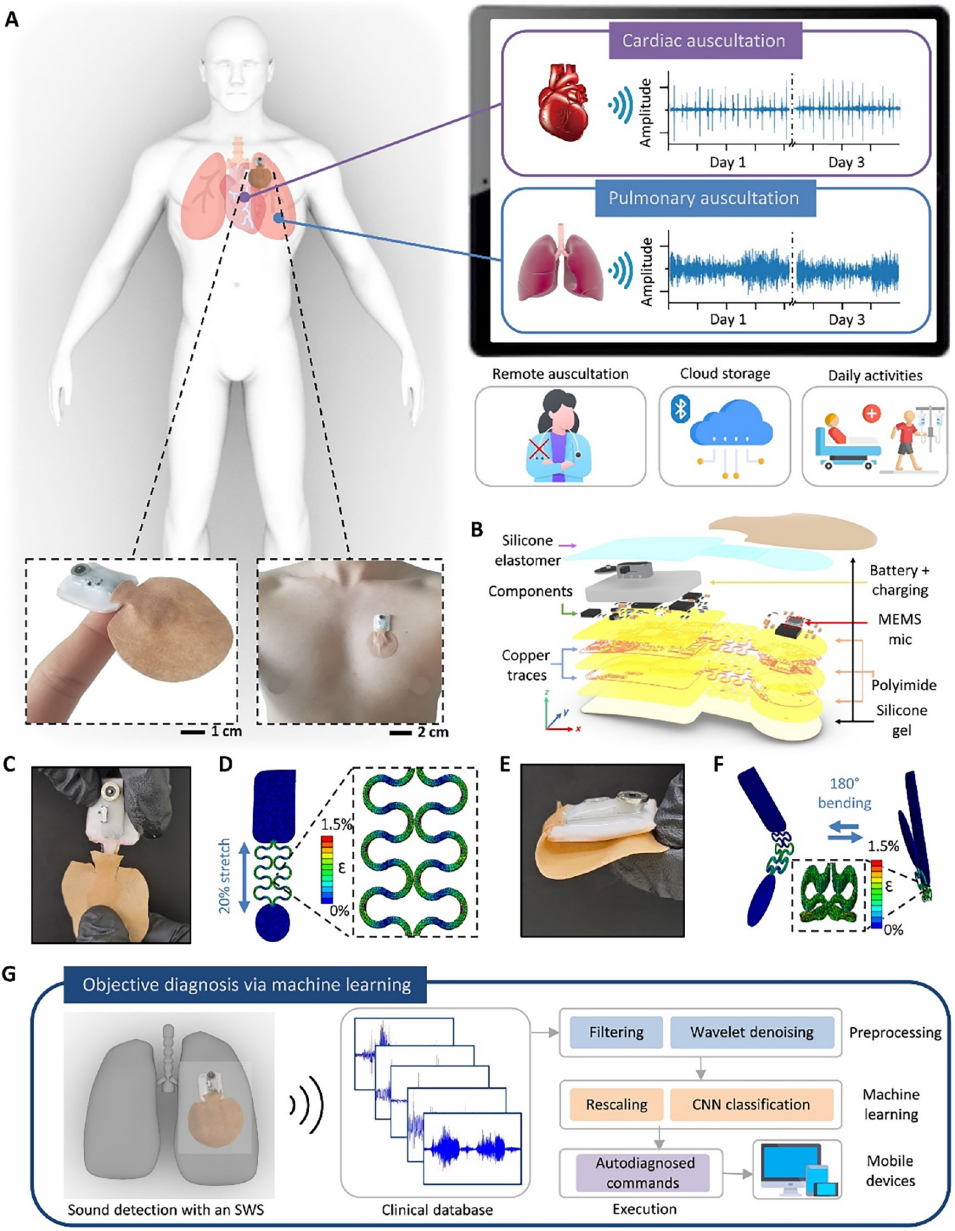
(A) Photos of the SWS and a schematic drawing of its position as attached to the human body. Also shown are the possible types of auscultation monitoring. (B) Representation of the SWS architecture, including the sensing parts and the signal acquisition component. (C) Image of the SWS interconnects when 20% stretched, whose properties can be simulated using finite element analysis (FEA) in (D). (E) Demonstration that the SWS can withstand 180° bending, with bending cycles being reproduced with FEA simulations. (G) Design of the ML-based diagnosis system, in which the sound detected using the SWS is preprocessed and then classified with ML algorithms. Reproduced from Lee et al.^1200^ Available under a CC BY-NC license. © 2022 American Association for the Advancement of Science.

The combination of ML and image analysis is gaining popularity, with classifications not based on images of biological samples but rather on images of the sensing units themselves.^1201^ This approach offers the clear advantage of directly using cameras or optical microscopes for monitoring and diagnosis. A notable example is the integration of mechanoluminescent sensing and biosensing, which facilitated the detection of bacterial infections while simultaneously measuring the orthodontic force.^1202^ The principles of detection are illustrated in Figure 63, featuring a mechanoluminescent elastomer composed of polyvinylidene fluoride-hexafluoropropylene (PVDF-HFP) embedded with SrAl_2_O_4_:Eu^2+^,Dy^3+^ (SAOED) phosphors to measure force. A biosensor consisting of a covalently cross-linked methyl acrylate-phenol red (MA-PR) and PAM film remains in direct contact with the teeth. Biosensing functions through the principle that lactic acid, a metabolic product of cariogenic bacteria, induces a color change upon reaction with MA-PR. The optical signal from this biosensor is captured by using a smartphone camera, and the images are subsequently classified by a deep learning algorithm.

**Figure 63:**
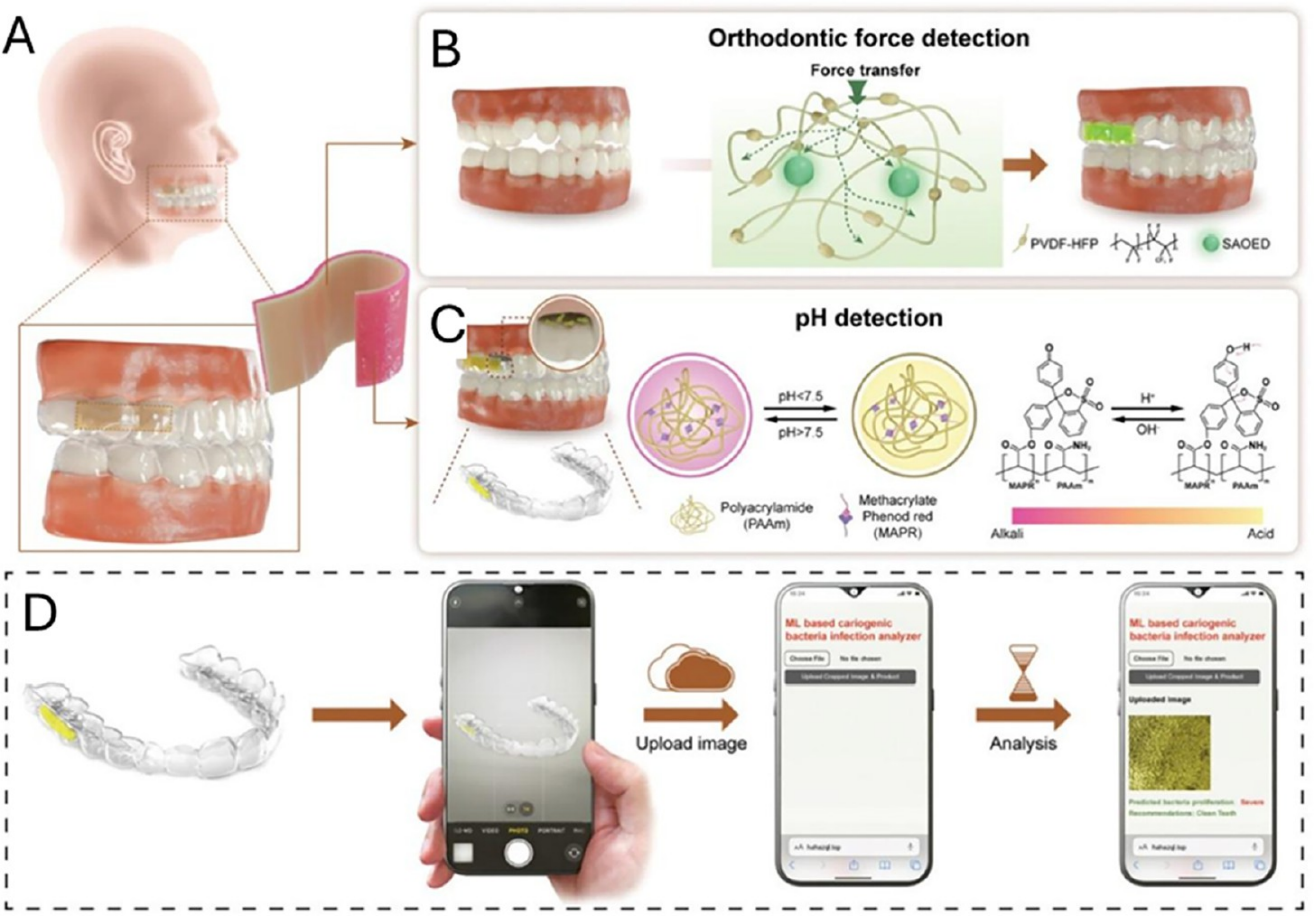
Illustration of the dual sensing system to measure orthodontic force and detect bacteria. (A) Pictorial representation of the dual sensing device, which is used to measure the force as depicted in (B) and detect bacteria infection via pH changes (C). The reaction shown refers to lactic acid and MA-PR. (D) Schematic illustration of image processing using a smartphone. Reproduced with permission from Feng et al.^1202^ © 2024 John Wiley & Sons.

The color change induced in the invisible aligner attachment (IAA) of the orthodontic device is not easily observable with the naked eye. Therefore, the images were processed and classified using the ResNet-50 CNN model.^1203^ Figure 64 illustrates the working principle and key results, with a high accuracy of 97.7% achieved in predicting infections caused by two bacteria, *Streptococcus mutans* and *Streptococcus sanguis*, for concentrations ranging from 10^1^ to 10^5^ CFU mL^–1^ (colony-forming units), as confirmed in the confusion matrix depicted in Figure 64C and further supported by the comparison of training and validation losses shown in Figure 64E. The 2D data visualizations of the image data (feature vectors) obtained with the multidimensional projection technique t-SNE (*t-distributed Stochastic Neighbor Embedding*) illustrate how the model learning capability: as shown in Figure 64F, the initial feature vectors of the images corresponding to distinct bacterial concentrations cannot be distinguished. In contrast, Figure 64G demonstrates clear and accurate clustering of the images after 100 epochs of CNN training, clearly demonstrating the model learns to differentiate the images.

**Figure 64:**
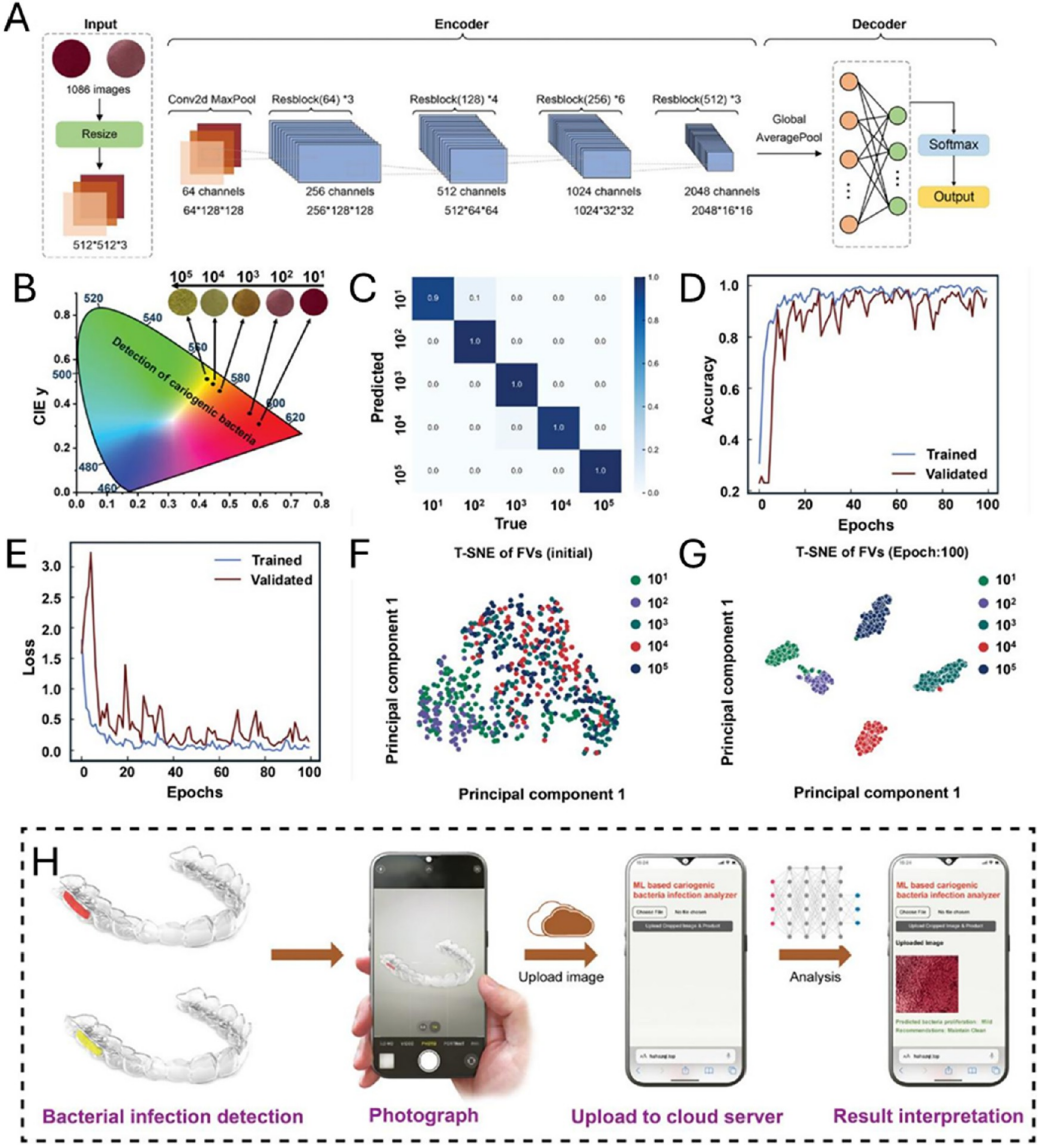
(A) Flowchart depicting the use of the ResNet-50 algorithm for image processing. (B) Color changes induced by different levels of proliferation of cariogenic bacteria. (C) Confusion matrix validating the classification accuracy for bacterial concentrations ranging from 10 to 10^5^ CFU mL^–1^. (D) Accuracy progression as the number of training epochs increases. (E) Comparison of training and validation losses. (F) 2D visualization of image data using t-SNE for different bacterial concentrations before training. (G) Same as (F), but after 100 epochs of CNN training. (H) Illustrative diagram of the biosensing mechanism using the invisible aligner attachment, with images captured by a smartphone and processed using a CNN algorithm. Reproduced with permission from Feng et al.^1202^ © 2024 John Wiley & Sons.

Speech analysis has been employed in diagnosis and health monitoring, traditionally performed by capturing sound with standard microphones. In recent years, it has been demonstrated that wearable pressure and strain sensors can also be utilized for such purposes. These applications require signal processing, followed by the establishment of speech patterns using ML algorithms. Although not specifically aimed at health monitoring, the work by Li et al.^1204^ presented an intelligent system capable of achieving 95% accuracy in speech recognition. This was accomplished using a skin-like piezoresistive sensor that could also detect human pulses and robot clamping. The skin-like flexible pressure sensor (SFPS) featured a laser-induced graphene layer on a PDMS film, whose piezoresistive properties enabled a detection sensitivity of 1348 kPa^–1^ in the 0–2 kPa range, with a wide operational range of up to 200 kPa. Figure 65a presents a schematic diagram of the speech recognition system, featuring a sensor (SFPS) attached to the throats of volunteers to measure the pressure of acoustic vibrations. The figure also depicts the structure of the CNN utilized in the system, consisting of three convolutional layers. The brief list of potential applications listed in the figure includes the development of voice-controlled robots, simultaneous translation, and speech reproduction. Figure 65b shows the current response curves of the SFPS corresponding to seven phrases. These phrases were classified with an accuracy of 95.3%, as demonstrated in the confusion matrix also presented in Figure 65b.

**Figure 65:**
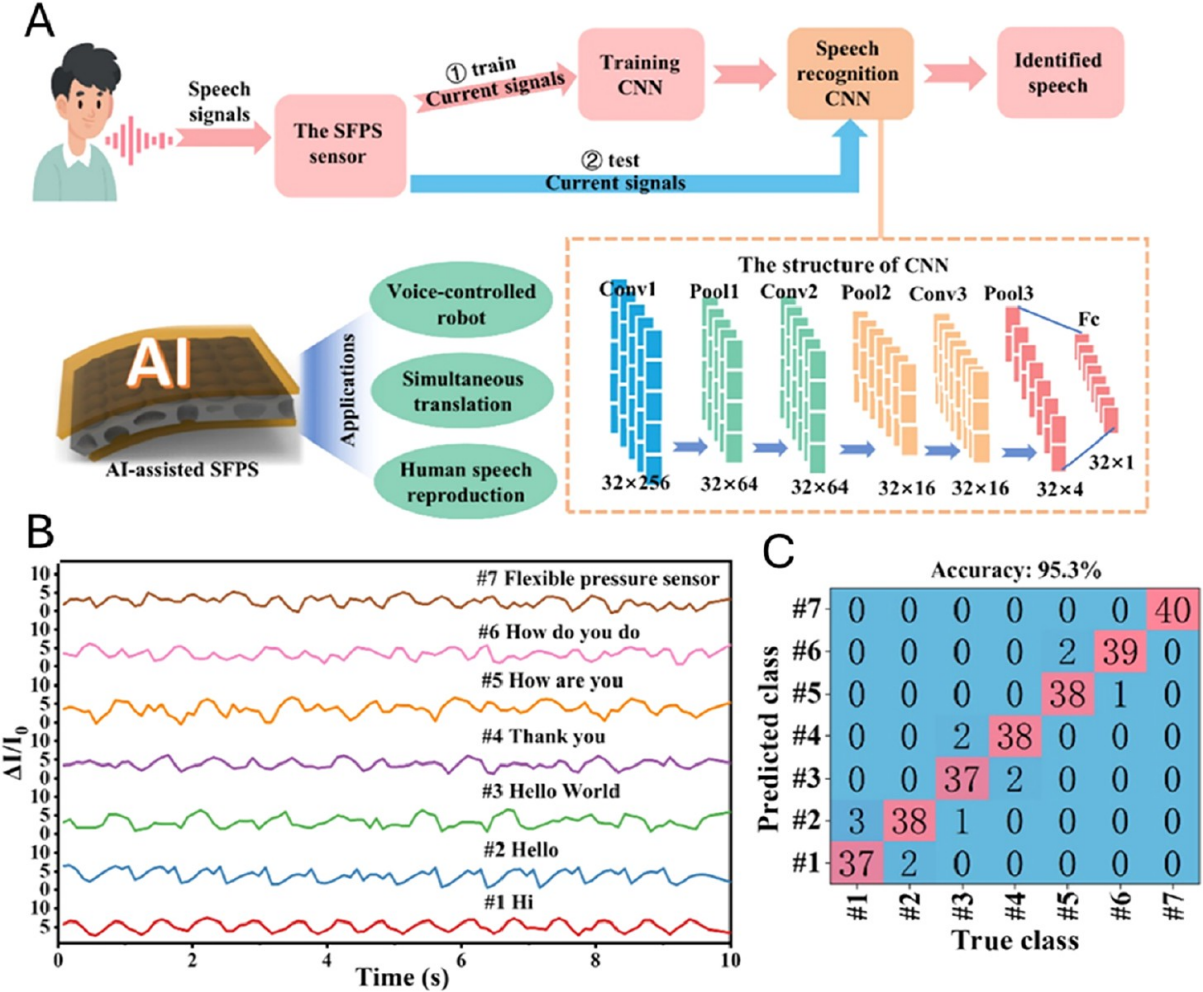
(A) Schematic of the speech analysis system, where signals captured by the skin-like flexible piezoresistive sensor (SFPS) are processed and classified using a three-layer CNN. Additional potential applications of the AI-assisted SFPS are also listed, including voice-controlled robots, speech reproduction, and simultaneous translation. (B) Current responses of the SFPS obtained from volunteers speaking seven phrases and (C) a confusion matrix demonstrate that the intelligent system can classify the phrases with an accuracy of 95.3%. Reproduced from Li et al.^1204^ © 2024 American Chemical Society.

Most examples of ML applied to health monitoring and diagnosis using wearable sensors rely on black-box models (e.g., deep neural networks), i.e., it is not possible to understand which properties of the data contributed to the predictions, and how. The aforementioned examples fall into this category. Models that are highly accurate, but difficult to interpret have limited applicability in many practical scenarios related to health monitoring. However, there is a growing body of research focused on interpretable (or explainable) ML, which aims to design strategies that allow humans to understand the rationale behind a model’s prediction. In the context of sensing data collected from various sources such as wearable devices, IoT systems, and medical instruments, interpretable ML is especially valuable. Sensor data is often complex, high-dimensional, and noisy, making the interpretability of ML models critical for fostering trust, ensuring transparency, and deriving actionable insights. Various strategies exist for achieving interpretable ML (or AI). Some involve the use of standard ML algorithms that incorporate rules, such as decision trees or random forests. This concept underpins the multidimensional calibration space (MCS) framework,^1205^ which enhances the calibration and classification accuracy of sensors and biosensors by accounting for the roles of multiple interdependent variables. MCS enables the construction of interpretable models capable of mapping complex relationships between inputs and sensor outputs. Compared to traditional calibration methods—which typically consider single variables or a limited set and often overlook intricate dependencies in real-world systems—MCS offers a significant advantage.

Another strategy to achieve interpretability is to investigate the importance of individual features for the performance of standard ML algorithms. This approach was followed by Kimura et al.^1206^ They combined lifestyle factors and wearable sensor data to predict high brain amyloid burden as determined by positron emission tomography. A high amyloid burden is associated with Alzheimer’s disease. The researchers used three ML algorithms to predict amyloid positivity: Elastic Net, kernel support vector machine, and logistic regression. Participants wore wristband sensors, and data were collected measuring variables such as conversation time, physical activity, sleep, and heart rate. After adding variables on lifestyle factors, demographic characteristics, and information on chronic diseases, 54 descriptive variables in total were input to the ML algorithms. The average prediction accuracy across the three algorithms was 0.79. In order to enhance model interpretability, the permutation importance estimate method was employed to identify the most relevant variables to distinguish individuals classified as positive or negative for amyloid across the three algorithms.

### 12 Business, Regulatory, and Ethical Considerations

The use of sensors for health monitoring is transitioning from the research stage into clinical trials and the use in humans for real-world applications. Several biosensors are already undergoing clinical testing and commercialization across different medical fields. For instance, GlySens developed implantable continuous glucose monitoring biosensors that offer long-term glucose tracking without frequent sensor replacement.^1207^ For neurological disorders, various companies emerged over the past few decades, developing biosensors for early disease diagnosis, such as depression or seizures. Motif Neurotech, a neurotechnology company, is developing minimally invasive bioelectronics for mental health treatment, specifically to treat depression. Recently, the company has raised US$18.75 million in Series A to support the development of a pea-sized brain implant designed to treat depression.^1208^ Similarly, Amber Therapeutics has developed Picostim, an innovative brain biosensor that has shown promise in reducing seizures in epilepsy patients.^1209^ Another example in the space of brain-machine interface is Neuralink, founded by Elon Musk in 2019. Neuralink’s biosensor platform aims to restore sensory and motor function and treat neurological disorders. The company began its first-in-human clinical trial in early 2024, successfully implanting its device in three patients. The first two recipients demonstrated the ability to control a computer mouse using thought alone. By the end of 2025, Neuralink plans to recruit 20–30 patients to advance commercialization. Additionally, the company has secured approval to commence clinical trials in Canada, targeting individuals with tetraparesis or tetraplegia, reflecting Neuralink’s commitment to international expansion and regulatory compliance.^1210,1211^ Elon Musk is also developing TeslaBot, a humanoid robot designed to perform tasks that are unsafe, repetitive, or mundane for humans. Elon Musk envisions Optimus revolutionizing industries such as manufacturing and logistics by automating various functions.^1212^

From a regulatory perspective, biosensors fall under the broader category of digital health and care which is defined by the USA Food and Drug Administration (FDA) and EU to mean the “tools and services that use information and communication technologies (ICTs) to improve prevention, diagnosis, treatment, monitoring, and management of health and lifestyle”, meaning biosensors are subject to regulations concerning medical devices, data protection, and cybersecurity. Within the medical devices section, biosensors may be considered medical devices if used for diagnosis, treatment, or monitoring, making them subject to strict regulations.^1213^ To comply with these regulations, the biosensor platforms need to be safe and reliable while maintaining privacy and data protection standards. In general, translating biosensors from the laboratory to clinical applications involves navigating a complex landscape of regulatory requirements to ensure their safety, efficacy, and reliability.

The adoption of biosensors for personalized medicine raises ethical, legal, and human rights concerns, particularly regarding access, fairness, and potential misuse. For example, animal use for testing newly developed biosensors is an important ethical aspect to consider. Traditional bioassays often require large amounts of animals or animal samples; on the other hand, biosensors have been presented as an alternative with the potential to reduce the number of animals that are needed to perfect and fully develop assays.^1214^ However, when running clinical trials with the newly developed biosensors, it is required to follow internationally recognized ethical guidelines, ensuring patient autonomy and well-being. These principles trace back to the Hippocratic Oath and have been reinforced by the World Medical Association through the Geneva Declaration (1948) and the Physician’s Pledge (2017). Additionally, key international frameworks, such as the Helsinki Declaration (1964) and the Universal Declaration on Bioethics and Human Rights (2005), provide ethical guidance.^1213^

### 13 Main Challenges and Prospects

This Mega Review provided a comprehensive account of the latest developments in wearable sensors for health monitoring, organized into numerous sections in which several important challenges and perspectives were already discussed. Given the breadth of the topic, it is clear that long-term progress will depend on interdisciplinary collaboration among materials scientists, engineers, data scientists, clinicians, and policymakers. It is essential to incorporate various values^1215^ and preferences, which adds difficulty to the endeavor.^1216^ In particular, the most difficult challenge lies in developing products that can be effectively deployed in society. By integrating innovations in energy harvesting, biocompatible materials, and intelligent algorithms with equitable design and robust regulation, wearable sensors can evolve from prototypes into practical healthcare tools. Wearable sensors must satisfy competing requirements: high sensitivity and accuracy, mechanical flexibility, long-term stability, comfort, and affordable production. They must handle complex biological environments—sweat, temperature fluctuations, and motion—without signal degradation. High interindividual variability in sweat composition, for instance, presents additional challenges in interpreting and standardizing analysis,^1217^ which may require sweat rate normalization^1218^ and active sweat gland induction.^29^ Furthermore, the explosion of data generated by wearables introduces new opportunities for AI and ML in healthcare, but also raises questions of privacy, transparency, and regulation. The challenges presented here focus, therefore, on materials, energy systems, device integration, data management and processing, clinical validation, and societal adoption.

#### 13.1 Materials

A central technical barrier in wearable sensor development lies in identifying materials that are simultaneously flexible, stretchable, durable, and biocompatible. Devices mounted on the skin or integrated into textiles are constantly exposed to mechanical deformation, moisture, temperature changes, and biological fluids. These factors can cause signal drift, degradation of active materials, or even user discomfort over prolonged use. Hydrogels, triboelectric polymers, 2D materials such as graphene, and LMs are promising candidates for soft electronics due to their mechanical adaptability and excellent electrical performance. However, hydrogels are particularly sensitive to humidity and temperature, which may lead to instability and poor reproducibility. Strategies to enhance drying and freezing resistance, or to introduce self-healing properties, are important for long-term stability. Similarly, LM-based sensors offer exceptional conductivity and flexibility but face issues of surface oxidation, electrochemical instability, and poor adhesion to rigid components. Developing robust encapsulation and interface engineering methods is essential for reliable operation in physiological environments. Current sensor designs encounter longevity and stability issues, especially due to the degradation of proteins, enzymes, and lipids in wound environments.^1219^ Also relevant is to employ sustainable materials^1220–1223^ and fabrication methods.^787,1224–1228^ Many current processes rely on nonrecyclable plastics and energy-intensive methods, increasing environmental impact and cost. Future research should prioritize biodegradable and recyclable materials, such as cellulose-based substrates or bioderived polymers, to align wearable technologies with sustainability goals.

#### 13.2 Energy

Energy supply remains one of the most persistent challenges for wearable sensors. Traditional batteries impose limitations due to size, weight, rigidity, and finite lifespan. Frequent recharging or replacement reduces user compliance and practicality, especially for continuous health monitoring. Self-powered technologies—particularly TENGs, PENGs, and TEGs—are being explored to harvest mechanical and thermal energy from the human body or the environment. However, these systems face low and inconsistent energy outputs, as their performance depends on user motion or temperature gradients. Moreover, the generated signals are often noisy, requiring advanced filtering and signal processing algorithms to extract relevant information. Hybrid energy systems that combine multiple harvesting mechanisms, coupled with miniaturized energy storage devices such as micro supercapacitors and flexible batteries, offer a promising path toward continuous, autonomous operation. The integration of energy management circuits within flexible substrates is another engineering frontier. To achieve compact, lightweight, and comfortable designs, all components—including energy harvesters, power management units, sensors, and wireless communication modules—must be embedded seamlessly within soft materials. Achieving this level of miniaturization without compromising durability or comfort requires innovation in both materials and circuit design.

#### 13.3 Device Integration

For wearable sensors to achieve widespread adoption, they must be comfortable, unobtrusive, and easy to use. The ideal wearable device should adapt to body movements, maintain stable contact with the skin, and operate reliably during daily activities such as exercise or sleep. Achieving this involves balancing mechanical compliance and structural integrity, as well as ensuring safe skin interfaces and breathable materials. Emerging solutions include textile-based sensors and soft robotics integration, where sensors are woven directly into fabrics or embedded within elastic substrates. However, the processing of conductive fibers, coatings, and adhesives remains complex, often incompatible with large-scale textile manufacturing. Electronic textiles (e-textiles) still lack a “killer application,” and their commercial success will likely begin with niche markets—such as athletic performance monitoring, medical garments, or military uniforms—before expanding to general use. Comfort and usability also extend to user interaction and data interpretation. Studies show that perceived complexity, especially among older adults, can discourage use. Therefore, wearable systems must provide intuitive interfaces and transparent feedback, potentially through smartphone integration or NFC systems that leverage existing user habits.

#### 13.4 Data Processing and Data Management

Exceptional perspectives—though accompanied by equally tremendous challenges—exist in the use of AI, particularly ML. Wearable sensors can now generate vast quantities of time-series and multimodal data,^802,1229–1231^ whose processing requires sophisticated algorithms capable of distinguishing meaningful physiological changes from noise. For example, ECG provides precise temporal information while PPG supplements hemodynamic parameters.^1232^ Combined with ML algorithms, a more comprehensive cardiovascular function assessment can be achieved.^1233^ AI and ML can detect subtle patterns, predict disease progression, and enable personalized interventions. In addition, multimodal fusion, combining electrical (ECG, EMG), optical (PPG), and mechanical signals, offers a richer understanding of cardiovascular and metabolic health. At the hardware level, advances in flexible electronics and micronano fabrication technologies have made sensors thinner, more flexible, and more integrated.^1234^ At the software level, DL can eliminate various interferences and extract true health information from the multisource data,^1235,1236^ which can assist in precision medicine and personalized health management.^1237^

However, several issues must be addressed for AI-driven insights to be clinically reliable. Data quality, standardization, and interoperability remain fundamental concerns as inconsistent sampling or sensor variability can compromise model accuracy. The lack of standardized biomarkers in nonblood fluids, such as sweat or saliva, further complicates clinical interpretation. Moreover, ensuring data privacy, cybersecurity, and algorithmic transparency is essential to building trust among patients and healthcare providers. A suitable e-health tool is expected to include clinical utility and safety, usability and human-centricity, functionality, and data management.^1238^ A growing movement emphasizes the adoption of FAIR data principles: findable, accessible, interoperable, and reusable within healthcare ecosystems.^1239^ While many industry-based wearable device providers store data streams on data clouds, healthcare institutions have started to prefer data storage on premises that includes full access to raw and processed data.^1240^ Implementing these principles requires collaboration between technologists, regulators, and clinicians to define consistent data formats, ethical frameworks, and secure infrastructures.

#### 13.5 Clinical Validation

Despite impressive laboratory demonstrations, few wearable sensors have achieved clinical-grade validation or regulatory approval.^1241,1242^ The regulatory landscape for wearables remains fragmented: while the FDA and European CE systems have pathways for medical devices, many emerging sensors fall between categories or lack standardized testing protocols. Clinical translation is hindered by variability in biofluids, lack of multisite studies, and high validation costs. For successful clinical integration, wearable technologies must demonstrate not only technical performance but also clinical utility, usability, and safety. In fact, perceived complexity, particularly in interpreting device outputs, deters adoption among seniors.^1243,1244^ Even more difficult is to deploy technology such as POC devices in underserved communities.^1245^ Collaborative frameworks involving healthcare institutions, academic researchers, and industry partners are necessary to conduct large-scale trials and define best practices. Cost is another major barrier—the estimated investment to bring a new medical device to market can exceed hundreds of millions of dollars when accounting for development failures and regulatory requirements. Nevertheless, some notable successes are emerging. Devices such as MC10s BioStamp^1246^ and Sibel Health’s ANNE One have achieved FDA clearance, validating the potential of soft, skin-conformal electronics for continuous monitoring. These examples illustrate how structural design, energy efficiency, and data integration can converge to produce clinically relevant wearables. Also, even consumer health devices can provide valuable sensing capabilities, saving lives in scenarios like out-of-hospital cardiac arrest^1247^ and detecting poor mental health states.^1248^

#### 13.6 Societal Adoption

Beyond technical and regulatory challenges, equity and accessibility are fundamental considerations for the evolution of wearable sensors. Adoption among elderly users and individuals in low-resource settings remains limited because of cost, connectivity barriers, and a lack of digital literacy. Future developments must therefore embrace user-centered and inclusive design, ensuring that devices are adaptable to diverse populations and economic conditions.

#### 13.7 The Future

The evolution of wearable sensors for health monitoring signals more than technological progress; it points to a profound transformation in how knowledge about the human body is generated, interpreted, and applied. As discussed throughout this review, advances in materials science, soft electronics, energy systems, and artificial intelligence are converging to enable continuous, real-time interaction with physiological processes. However, the most significant implication of this convergence may lie beyond improved diagnostics or device performance. It suggests the emergence of what can be described as a fifth paradigm of knowledge generation,^1179,1180^ in which cognition is no longer exclusively human but is increasingly distributed across hybrid human–machine systems.

In this new paradigm, wearable sensors act as interfaces between the biological and digital, continuously translating physiological signals into data streams that can be processed, contextualized, and acted upon by intelligent algorithms. Unlike previous paradigms of knowledge generation, ranging from empirical observation to theory-driven science and data-intensive discovery, this new framework is characterized by autonomous, adaptive, and coevolving systems, where sensing, analysis, and decision-making occur in an integrated and dynamic loop. Wearable technologies thus become not only tools for measurement but also active agents in the production of biomedical knowledge. A key manifestation of this shift is the move toward continuous multimodal physiological mapping, enabling the construction of high-dimensional representations of human health. When coupled with AI methods, these data streams can reveal complex correlations and predictive patterns that are inaccessible through traditional clinical approaches. The role of AI in this context extends beyond data analysis: it becomes a cocreator of knowledge, capable of generating hypotheses, identifying anomalies, and guiding experimental or clinical interventions in real time and autonomously. This fundamentally challenges the classical separation between measurement and interpretation.

The integration of sensing and actuation further amplifies this transformation. Closed-loop wearable systems capable of responding to physiological changes blur the boundary between diagnosis and therapy. In such systems, knowledge is not only descriptive but operational, directly informing and executing interventions.This real-time coupling between perception and action reflects a cybernetic view of healthcare in which the human body is embedded within a responsive technological ecosystem.

## Footnotes

a The 3D visualization at CitationNetwork reveals a dense cluster aggregation due to topic proximity, with some exceptions.

## Abbreviations list

AFM: Atomic Force Microscopy
AI: Artificial intelligence
AIr: Radial artery augmentation index
a-IGZO: Amorphous indium gallium zinc oxide
AgNF: Silver nanofiber
AgNP: Silver nanoparticle
AgNW: Silver nanowire
ALMP: Adhesive liquid metal particles
ALS: Amyotrophic lateral sclerosis
AMR: Antimicrobial resistance
AOx: Alcohol oxidase
BFC: Biofuel cell
BLE: Bluetooth low energy
BP: Burst pressure
CA: Calcium alginate
CBT: Core body temperature
CCP: Conductive carbon paste
CF: Cystic fibrosis
CNC: Computer numerical control
CNCry: Cellulose nanocrystal
CNN: Convolutional neural networks
CNT: Carbon nanotube
COPD: Chronic obstructive pulmonary disease
CQD: Carbon quantum dot
CRP: C-reactive protein
CSF: Cerebrospinal fluid
CV: Cyclic voltammetry
DA: Dopamine
DBP: Diastolic blood pressure
DL: Deep learning
DPV: Differential pulse voltammetry
DPP: Diketopyrrolopyrrole
EAB: Electrochemical aptamer-based
EBA: Exhaled breath aerosols
EBC: Exhaled breath condensate
ECG: Electrocardiogram
EGaIn: Eutectic gallium-indium
E-LIG: Electrografted laser-induced graphene
ELISA: Enzyme-linked immunosorbent assay
EMG: Electromyography
EMGe: Electromagnetic generator
ESM: Eggshell membrane
EVA: Ethylene-vinyl acetate
FDA: Food and Drug Administration
FITC: Fluorescein isothiocyanate
FUTUD: Ultra-wideband triboelectric ultrasonic device
GABA: γ-aminobutyric acid
GI: Gastrointestinal
GFAP: Glial fibrillary acidic protein
GM: Gut microbiota
GO: Graphene oxide
GOx: Glucose oxidase
GSH: Glutathione
HEG: Hydrovoltaic effect generator
HVA: Homovanillic acid
IAA: Invisible aligner attachment
ICT: Information and communication technologies
HR: Heart rate
HRV: Heart rate variability
IC: Integrated circuit
IDE: Interdigitated electrode
IoT: Internet of Things
IR: Infrared
ISE: Ion-selective electrode
ISF: Interstitial fluid
KNN: K-nearest neighbor
LC-MS: Liquid chromatography-mass spectrometry
LED: Light-Emitting Diode
LIG: Laser-induced graphene
LFA: Lateral flow assay
LM: Liquid metal
LOD: Limit of detection
LOx: Lactate oxidase
LP: Lumbar puncture
LTPS: Low temperature polycrystalline silicon
MBA: N,N′-methylene-bis-acrylamide
MCS: Multidimensional calibration space
MEG: Moisture effect generator
MEMs: Microelectromechanical system
MICS: Medical implant communication service
MIP: Molecularly imprinted polymer
ML: Machine Learning
MMP: Metalloproteinases
MN: Microneedle
MNC: Microbial nanocellulose
MTMOS: Methyltrimethoxysilane
NCL: Neuroprosthetic contact lenses
NFC: Near-field communication
NMR: Nuclear magnetic resonance
NP: Nanoparticles
NPL: Natural Language Processing
NTC: Negative temperature coefficient
NW: Nanowire
OCV: Open-circuit voltage
OECT: Organic electrochemical transistors
OFET: Organic field-effect transistors
OTFT: Organic thin film transistor
P3HT: Poly­(3-hexylthiophene)
PAM: Polyacrylamide
PAMD: Polymer-assisted metal deposition
PANI: Polyaniline
PC: Capillary pressure
PCA: Principal component analysis
PCR: Polymerase chain reaction
PD: Photodetectors
PDA: Polydopamine
PdNP: Palladium nanoparticle
PDMS: Polydimethylsiloxane
PECVD: Plasma-enhanced chemical vapor deposition
PEDOT: Poly­(3,4-ethylenedioxythiophene)
PEG: Polyethylene glycol
PENG: Piezoelectric nanogenerators
PEO: Polyethylene oxide
PET: Polyethylene terephthalate
PETG: Polyethylene terephthalate glycol
PIT: Passive inductive telemetry
PH: Hydrostatic pressure
PI: Polyimide
PL: Laplace pressure
PLA: Poly­(lactic acid)
PLGA: Poly­(lactic-co-glycolic acid)
PMEHB: Poly­(MPC-co-EHMA-co-MBP)
PMMA: Polymethyl methacrylate
PMU: Power management unit
POC: Point of care
POU: Point of use
PPG: Photoplethysmography
PPy: Polymeric conductive adhesive
PSA: Pressure sensitive adhesive
PSS: Poly­(styrenesulfonate)
PTFE: Polytetrafluoroethylene
PU: Polyurethane
PVA: Poly­(vinyl alcohol)
PVC: Polyvinyl chloride
PVDF: Polyvinylidene fluoride
P­(VDF-TrFE): Poly­(vinylidene fluoride-co-trifluoroethylene)
PyNG: Pyroelectric nanogenerator
PZT: Lead zirconate titanate
Qsc: Short-circuit transferred charge
Rct: Charge transfer resistance
RIE: Reactive ion etching
RF: Radio frequency
RFID: Radio frequency identification
rGO: Reduced graphene oxide
ROS: Reactive oxygen species
RTD: Resistive temperature device
SBP: Systolic blood pressure
SBS: Styrene-butadiene-styrene
SCFA: Short-chain fatty acid
SEBS: Styrene-ethylene-butylene-styrene
SEM: Scanning Electron Microscopy
SERS: Surface-enhanced Raman spectroscopy
SF: Silk fibroin
SFPS: Skin-like flexible pressure sensor
SNGC: sonogel-carbon
SPCE: Screen-printed carbon electrodes
SSP: Spoof surface plasmon
STEP: Stretchable tubular elastomeric piezoresistive
SUPS: Self-powered ultrasensitive pulse sensor
SVM: Support vector machine
SWASV: Square wave anodic stripping voltammetry
SWS: Soft wearable stethoscope
SWV: Square wave voltammetry
tcPO2: Transcutaneous oxygen partial pressure
TDM: Therapeutic Drug Monitoring
TEG: Thermoelectric generators
TENG: Triboelectric nanogenerator
TFT: Thin film transistors
TPU: Thermoplastic polyurethane
T-sensors: Temperature sensors
t-SNE: t-distributed Stochastic Neighbor Embedding
UV: Ultraviolet
Vis: Visible
VOC: Volatile organic compound
WiFi: Wireless Fidelity
WTF: Wireless power transfer
XAI: Explainable Artificial Intelligence
2-FMA: 2-fluoro-methamphetamine
βCD: β-cyclodextrin
μPAD: Microfluidic Paper-based analytical device
